# Elucidating the cellular response of silver nanoparticles as a potential combinatorial agent for cisplatin chemotherapy

**DOI:** 10.1186/s12951-020-00719-x

**Published:** 2020-11-10

**Authors:** Renata Rank Miranda, Micaella Pereira da Fonseca, Barbara Korzeniowska, Lilian Skytte, Kaare Lund Rasmussen, Frank Kjeldsen

**Affiliations:** 1 Department of Biochemistry and Molecular Biology, University of Southern Denmark, Odense, 5230 Denmark; 2 Department of Physics, Chemistry and Pharmacy, University of Southern Denmark, Odense, 5230 Denmark

**Keywords:** Silver nanoparticles, Combination chemotherapy, Proteomics, Cell viability, Metal uptake

## Abstract

**Background:**

Combination chemotherapy uses drugs that target different cancer hallmarks, resulting in synergistic or additive toxicity. This strategy enhances therapeutic efficacy as well as minimizes drug resistance and side effects. In this study, we investigated whether silver nanoparticles act as a combinatorial partner to cisplatin. In so doing, we compared post-exposure biological endpoints, intracellular drug accumulation, and changes in the proteome profile of tumoral and normal cell lines.

**Results:**

Combinatorial exposure corresponded to cytotoxicity and oxidative stress in both cell lines, yet was substantially more effective against tumoral cells. Proteome analysis revealed that proteins related to energy metabolism pathways were upregulated in both cell lines, suggesting that combinatorial exposure corresponded to energetic modulation. However, proteins and upstream regulators involved in the cell cycle were downregulated, indicating reduced cell proliferation. The response to oxidative stress was markedly different in both cell lines; downregulation of antioxidant proteins in tumoral cells, yet upregulation of the antioxidant defense system in normal cells. These outcomes may have avoided higher cell death rates in normal cells.

**Conclusions:**

Taken together, our results indicate that combining silver nanoparticles with cisplatin increases the biological activity of the latter, and the combination warrants further exploration for future therapies. 
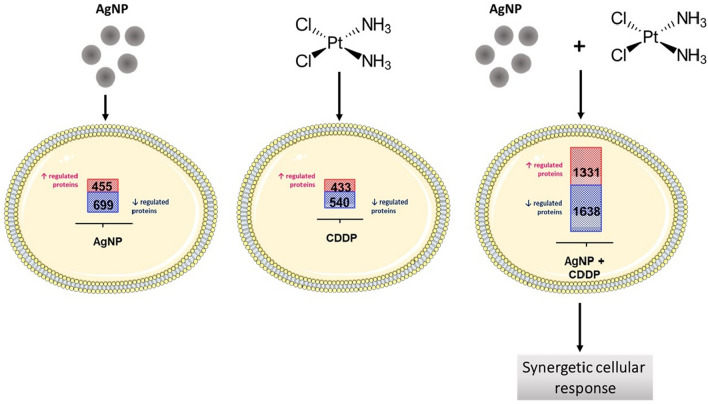

## Background

Nanotechnology arises from unique properties that are attributable to nanoscale structure, and has substantial transformative potential [1, 2]. Disciplines such as physics, chemistry, biology and medicine make use of these properties. In biomedicine, particularly cancer therapy, nano-sized materials have helped researchers improve existing treatments, devise new treatments (e.g., pharmaceutical nanocarriers, photothermal therapy and gene therapy) and diagnostics (e.g., nanosensors and bioimaging agents) [3–6].

Silver nanoparticles (AgNPs) are particularly interesting for cancer therapy due to their potential antitumoral effect, demonstrated by many in vitro studies. It has been shown that AgNPs may hinder cancer cell homeostasis by triggering an increase in reactive oxygen species (ROS), which corresponds to decreased proliferation rates as well as macromolecular damage and cell death [7–10]. Moreover, AgNPs may also disrupt important cancer hallmarks, such as glucose metabolism and drug resistance. Exposure to AgNPs corresponds to decreased lactate production and downregulation of glycolytic enzymes [11, 12], and inhibited efflux activity of multidrug resistance transporters in cancer cells [13, 14]. Researchers have also observed antitumoral effects of AgNPs in solid tumors of animal models, leading to inhibition of lymphosarcoma progression in rats [15], reduced tumor volume in Dalton’s ascites tumors in mice [16] and efficacy against triple-negative breast cancer xenografts in mice [17].

Taken together, these findings underscore the potential of AgNPs as an attractive candidate for the design of antitumoral drugs and treatments. We hypothesize that AgNP toxicity might be useful for enhancing traditional chemotherapy, by synergetic toxicity. In this study, we used AgNPs as a combinatorial agent for cisplatin (CDDP), a widely used antineoplastic drug for several cancers. We conducted mass spectrometry (MS)-based proteomics analyses to reveal molecular events triggered by combined AgNPs/CDDP exposure in a hepatocarcinoma cell line (HepG2) and a normal hepatocyte cell line (THLE2). We also investigated changes in biological endpoints (e.g., levels of viability and ROS) and intracellular metal content after exposure in both cell lines. Our AgNPs/CDDP combination was toxic to both cell lines. However, the exposure was substantially more effective against tumoral cells and therefore warrants further exploration for cancer therapies.

## Methods

### Characterization of AgNPs

We obtained spherical AgNPs (10-nm diameter, in citrate buffer: 1 mg/mL, 2 mM) from Nanocomposix. We characterized the nanoparticles’ size distribution by DLS and zeta potential (Zetasizer Nano ZS, Malvern Instruments, US). Particle shape analysis, kindly provided by Nanocomposix, was accessed with electron transmission microscope (JEOL 1010, Tokyo, Japan).

### Culture of normal and tumoral liver cell lines

We cultured HepG2 cells (Sigma–Aldrich) as a monolayer in high-glucose Dulbecco’s modified Eagle’s medium (DMEM) supplemented with 10% v/v inactivated fetal bovine serum (FBS) and antibiotics (10 U/mL penicillin and 10 µg/mL streptomycin), at 37 °C and 5% CO_2_. Cells at passages 112–120 were utilized to conduct the experiments. We plated normal human liver cell line THLE2 (ATCC) on culture flasks pre-coated with a solution containing 0.01 mg/mL fibronectin, 0.03 mg/mL bovine collagen type I, and 0.01 mg/mL bovine serum albumin dissolved in DMEM medium. These cells were cultured as a monolayer in bronchial epithelium basal medium (Lonza), supplemented with bronchial epithelial growth medium (Lonza) and 10% v/v FBS, at 37 °C and 5% CO_2_. We used cells at passages 3–6 to conduct the experiments.

### Exposure protocol

We seeded cells onto a 96-well microplate for biochemical analyses, a six-well plate for metal quantification and 100-mm petri dishes for proteomics analyses. The seeding density applied for HepG2 and THLE2 cells was 1 × 10^5^ and 2 × 10^4^ cell/mL, respectively.

After 24 h, we replaced the medium with fresh complete medium containing AgNPs, CDDP or a combination of both. We exposed cells for 24 h using appropriate controls.

### Cell viability assay

HepG2 cells were exposed to AgNPs (0–10 µg/mL) or CDDP (0–200 µM) for 24 h, for the initial toxicity screening tests. In order to investigate toxicological interactions between AgNP and CDDP, HepG2 cells were exposed for 24 h to AgNPs (1–5 µg/mL), CDDP (10 or 40 µM) and corresponding combinations. The most suitable concentrations of the co-exposure of AgNP/CDDP (3.5 µg/mL and 10 or 40 µM, respectively) were also applied to THLE-2 cells, during 24 h.

At the end of the exposure period, we investigated 3-(4,5-dimethylthiazol-2-yl)-2,5-diphenyltetrazolium bromide (MTT, Sigma-Aldrich) metabolism in both cell lines after incubating the cells with 0.5 mg/L MTT for 2 h. Subsequently, cells were washed with phosphate-buffered saline (PBS) and formazan was solubilized with 100 µL dimethyl sulfoxide. For the assay, we measured the absorbance at 560 nm using a FLUOstar Omega plate reader (Germany).

### ROS levels

We evaluated cytosolic H_2_O_2_ levels with 2′,7′-dichlorodihydrofluorescein diacetate (H_2_DCF-DA, Sigma-Aldrich) and mitochondrial superoxide levels with MitoSOX Red (Thermo Fisher). After exposure to AgNP (3.5 µg/mL), CDDP (10 and 40 µM), or a combination of both for 24 h, cells were incubated with either 10 µM H_2_DCF-DA or 5 µM MitoSOX in fresh culture medium (15 min, 37 °C, protected from light), washed with PBS, followed by the addition of 250 µL PBS. We measured the fluorescence at 488/530 nm (H_2_DCF-DA) and 514/580 nm (MitoSOX) using a FLUOstar Omega plate reader (Germany).

### Intracellular metal concentration

We quantified intracellular concentrations of silver (Ag) and platinum (Pt) by inductively coupled plasma–mass spectrometry (ICP–MS; Bruker 820-MS + SPS 3 autosampler). Cells were seeded onto six-well plates, cultured for 24 h and exposed to AgNP (3.5 µg/mL), CDDP (10 and 40 µM), or a combination of both, for 4 h. Subsequently, we washed the cells 3 × with PBS (to remove AgNPs and CDDP from the cell surface), trypsinized (0.25% trypsin, 0.02% EDTA, in pH 7.2 PBS), harvested, and pelleted the cells in complete culture medium (500*g* for 5 min). Pellets were further digested with 2% v/v HNO_3_ overnight at room temperature and remained at −20 °C until ICP–MS analysis. We used five-point calibration curves for quantification, and NIST 1486 (for the ICP–MS) for quality control. We performed three independent replicates; the results are expressed in ppm/10^3^ cells.

### Statistical procedures for biochemical and metal quantitation assays

We conducted three independent experiments with three replicates each for biomarkers analyzed in 96-well microplates and metal quantitation analysis. Data distribution was tested and parametric (one-way analysis of variance, ANOVA) tests were performed, followed by Bonferroni’s post-test. We verified the effects of exposures by a comparison of the control versus AgNPs, CDDP or AgNPs/CDDP. Toxicological interaction effects induced by co-exposure with AgNPs/CDDP were identified by a comparison of the co-exposure group versus the single-exposure groups AgNP or CDDP and is represented by the # symbol. We considered *p*-values less than 0.05 to be statistically significant.

### Sample preparation for MS-based proteomics analysis

At the end of the exposure period, we discarded the culture medium and carefully washed the cells 3 × with ice-cold PBS. Next, 1 mL of ice-cold PBS plus protease inhibitor (ProtoSTOP, Roche) was added to the plates and the cells were harvested with the aid of a cell scraper. We centrifuged the cell suspensions for 5 min at 600 *g* and discarded the supernatant. Cell pellets were stored at −80 °C until further analysis.

We resuspended cell pellets in lysis buffer (6 M urea, 2 M thiourea, protease inhibitors, 20 mM triethylammonium bicarbonate, and 10 mM 1,4-dithiothreitol reducing agent) at room temperature for 2 h. Then, the urea concentration was diluted 10 × and the cell lysis was enhanced by tip sonication on ice. We quantified proteins using Qubit fluorometric quantification (LifeTechnologies) and alkylated 50 µg of proteins in 20 mM iodoacetamide for 30 min in the dark. Following incubation, proteins were digested with trypsin (50:1 w/w protein:trypsin) overnight at room temperature. We acidified the peptide solution with 1% v/v formic acid to stop trypsin digestion and dried the peptides prior to desalting.

### Desalting with R2/R3 microcolumns

Samples were resuspended in 0.1% v/v trifluoroacetic acid (TFA) and desalted using self-made P200 columns, made with a C8 plug (Empore, 3 M purification) packed with 1:1 Poros R2 and R3 (Applied Biosystems) resins materials in 100% acetonitrile (ACN). The column was prepared by applying a mild air pressure with a syringe and washing the column 2 × with 0.1% v/v TFA. Subsequently, we loaded the acidified samples to the columns and washed them 2 × with 0.1% v/v TFA. Peptides were eluted with 30% v/v ACN, 0.1% v/v TFA, followed by 70% v/v ACN, 0.1% v/v TFA.

### Peptide labeling

We labeled tryptic peptides (50 µg per sample group) with the isobaric tag for relative and absolute quantitation (iTRAQ) 4-plex, in accordance with the manufacturer’s protocol. For both cell lines, the tags used to label each experimental condition, in triplicate, were as follows: control (114), AgNPs (115), CDDP (116), and AgNPs/CDDP (117). We combined the peptides in a 1:1:1:1 ratio, dried them under vacuum and stored them at −20 °C until further processing.

### Sample fractionation

To reduce complexity and remove unbound iTRAQ reagents, we pre-fractionated samples in an automated manner in reversed-phase at high pH, using a Dionex 3000 system (Thermo Fisher Scientific). Samples were solubilized in buffer A (20 mM ammonium formate, pH 9.2) and the column was loaded. Finally, we eluted the peptides at 100 nL/min by increasing buffer B (80% v/v ACN and 20% v/v buffer A) from 2% to 50%, over 85 min. We sampled 20 fractions, which we concatenated into 10 fractions. These samples including the flow-through were then dried in a vacuum centrifuge.

### Reversed-phase nano-liquid chromatography–tandem MS

We resuspended each high-pH fraction in 0.1% v/v formic acid (FA) and loaded them on an in-house packed trap column (3-cm × 100-µm inner diameter; 5 µm) filled with ReproSil-Pur C18 AQ (Dr. Maisch, Ammerbuch–Entringen, Germany). Peptides were separated on an analytical column (18-cm × 75-µm inner diameter; 3 µm) packed in-house with ReproSil-Pur C18 AQ (Dr. Maisch, Ammerbuch-Entringen, Germany), by reversed-phase chromatography on an EASY-nanoLC system (Thermo Fisher Scientific). The chromatography gradient was as follows: 0% to 3% B for 3 min, 3% to 25% B for 80 min, 25% to 45% B for 15 min, 45% to 100% B for 3 min, followed by 8 min in 100% B (A: 0.1% v/v FA; B: 95% v/v ACN, 0.1% v/v FA) at a constant flow rate of 250 nL/min. We connected the Easy-nanoLC system online to a Q Exactive high-field hybrid quadrupole–orbitrap mass spectrometer (Thermo Fisher Scientific) operating in positive ion mode using data-dependent acquisition. The Orbitrap acquired the full scan with an automatic gain control target value of 3 × 10^6^ and a maximum injection time of 100 ms. We acquired each mass spectrometer scan at a resolution of 60,000 at an *m*/*z* 200 with a mass range of *m*/*z* 400–1600. We subjected the 20 most-intense precursor ions (charge from 2 to 5) to higher-energy collisional dissociation fragmentation. Fragmentation was performed at a normalized collisional energy of 30% using an isolation width of 1.2 Da and a dynamic exclusion duration of 20 s. We acquired tandem mass spectrometry (MS^2^) spectra at 30,000 resolution, *m*/*z* 200, with an automatic gain control of 1 × 10^5^ and a maximum injection time of 200 ms.

### Database search and bioinformatics analyses

We processed raw data using Proteome Discoverer v2.1.1.21 (Thermo Fisher Scientific) and searched against the SwissProt human database using the Mascot search engine. Trypsin was chosen as the enzyme, allowing two missed cleavage sites. We used a precursor mass tolerance of 10 ppm and a product ion mass tolerance of 0.02 Da. Fixed modifications included carbamidomethylation of cysteines and iTRAQ4-plex labeling for lysines and N-termini. Dynamic modifications contained methionine oxidation and N-terminal acetylation. We calculated false discovery rates using the Percolator algorithm (*q*-value filter set to 0.01). Quantification was performed using the Proteome Discoverer workflow node “Reporter Ions quantifier” on the log2-values of the measured normalized peptide abundances. We determined protein regulations using the Limma ranked-product approach [18]. Only proteins with *p*-values ≤ 0.01 were considered to be regulated. We submitted regulated proteins to Ingenuity Pathway Analysis (IPA; Quiagen) to elucidate cellular protein responses induced by exposure to the contaminants, and to the Search Tool for the Retrieval of Interacting Genes/Proteins (STRING) app from Cystoscope to reveal protein–protein interactions [19].

## Results and discussion

### AgNP characterization

**Fig. 1:**
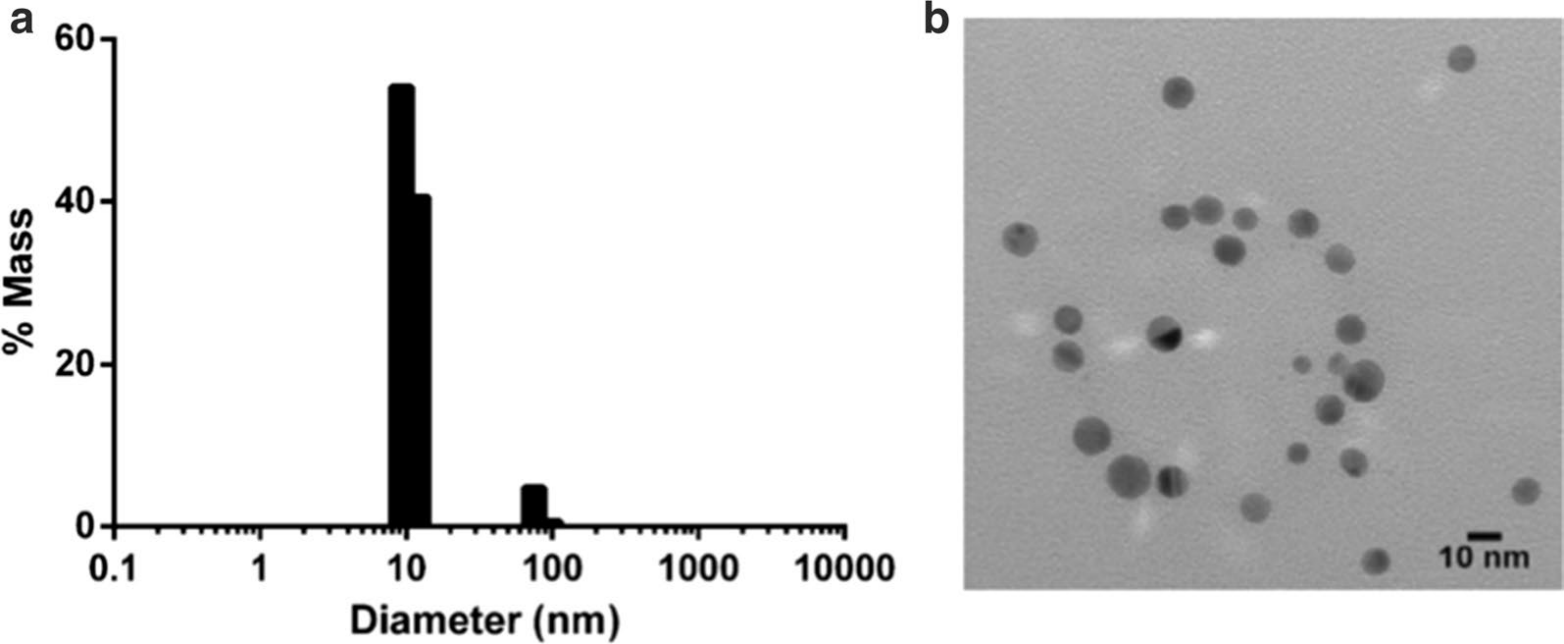
Silver nanoparticle (AgNP) characterization. **a** Particle distribution in accordance with size. **b** Transmission electron microscopy image of the AgNP suspension

Dynamic light scattering measurements indicated that the AgNP suspension had a major peak with a mass distribution of approximately 10 nm (Fig. 1a). The zeta potential of the sample was −38.9 mV ± 1.75 mV, measured for AgNPs dispersed in the citrate buffer with the addition of 1 mM KCl, indicating good colloidal nanoparticle stability (data not shown). Nanocomposix provided the transmission electron microscopy images, which confirmed that the AgNPs were monodisperse and spherical (Fig. 1b).

### AgNPs/CDDP combination induces toxicological interaction in HepG2 cells

**Fig. 2:**
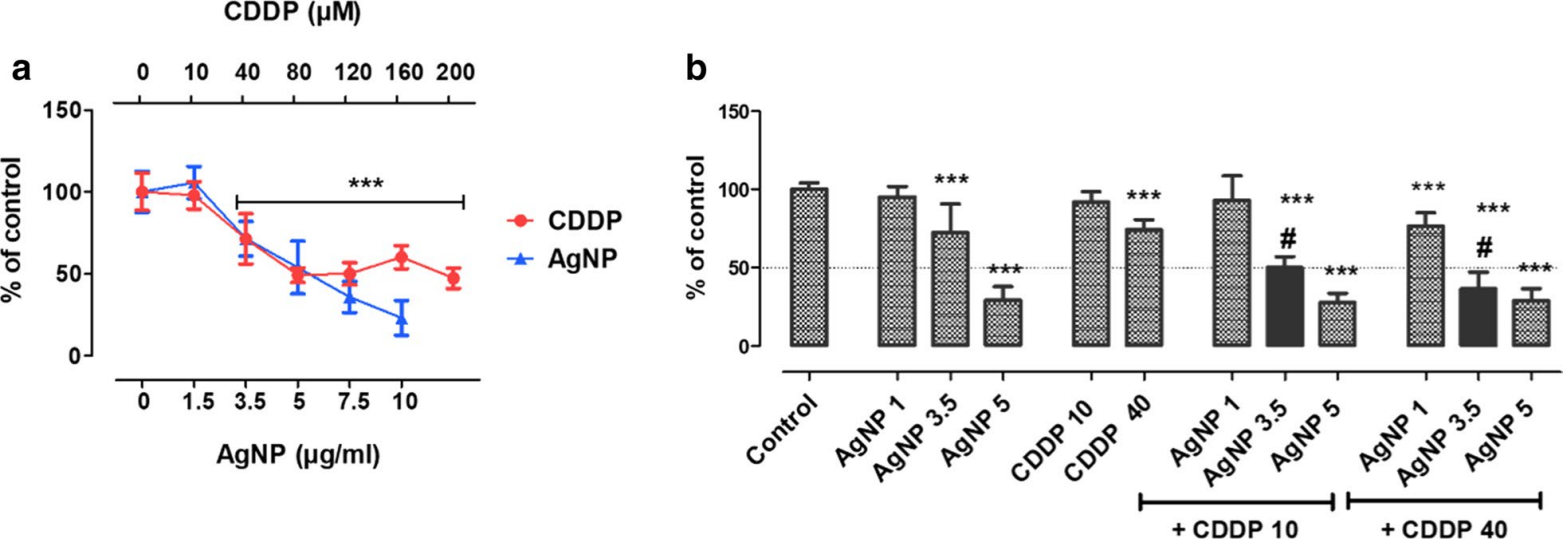
Toxicity screening. **a** MTT assay performed in HepG2 cells after 24 h of exposure with AgNPs (0–10 µg/mL) or CDDP (0–200 µM). **b** MTT assay performed in HepG2 cells after 24 h of exposure with AgNPs (1–5 µg/mL), CDDP (10 or 40 µM) and corresponding combinations. Mean + standard deviation (SD) of three independent experiments in triplicate. Asterisks indicate difference in comparison to the control (***p < 0.001); sharp symbol (#) indicates toxicological interaction

We performed toxicity screening tests based on MTT metabolism in HepG2 cells. We applied various concentrations of AgNPs (0–10 µg/mL) and CDDP (0–200 µM) for 24 h, to select the concentrations to be tested for drug interaction assays (Fig. 2a). Based on these results, we selected three concentrations of AgNPs (1 µg/mL, no toxicity; 3.5 µg/mL, ≈ 30% viability loss; and 5 µg/mL, ≈ 50% viability loss) and two concentrations for CDDP (10 µM, no toxicity; and 40 µM, ≈ 30% viability loss) to study the cytotoxic interaction of AgNPs and CDDP.

These results indicate that 3.5 µg/mL AgNPs in combination with both nontoxic and moderately toxic concentrations of CDDP generate an interaction effect (i.e., higher than the sum of the toxicity induced by single exposures), which significantly lessened the viability of HepG2 cells (Fig. 2b). To date, only a few studies have investigated the ability of AgNPs to enhance the toxicity caused by conventional chemotherapy. Combining AgNPs with doxorubicin is efficient against breast and liver cancer [20, 21], due to increased cytotoxicity and ROS levels. AgNPs together with salinomycin or gemcitabine leads to enhanced apoptosis, oxidative stress, and cytotoxicity in ovarian cancer cells [22, 23]. Kovács and coworkers investigated AgNPs combined with several antineoplastic drugs and found, for all test combinations, synergy against adenocarcinoma cells [14]. These studies indicate the promising potential of AgNPs as a combinatorial agent for chemotherapy. However, researchers do not fully understand how this toxicological interaction occurs in detail and particularly how normal cell lines respond to such combinatorial exposure.

### HepG2 cells are more susceptible to AgNPs/CDDP than THLE2 cells

**Fig. 3:**
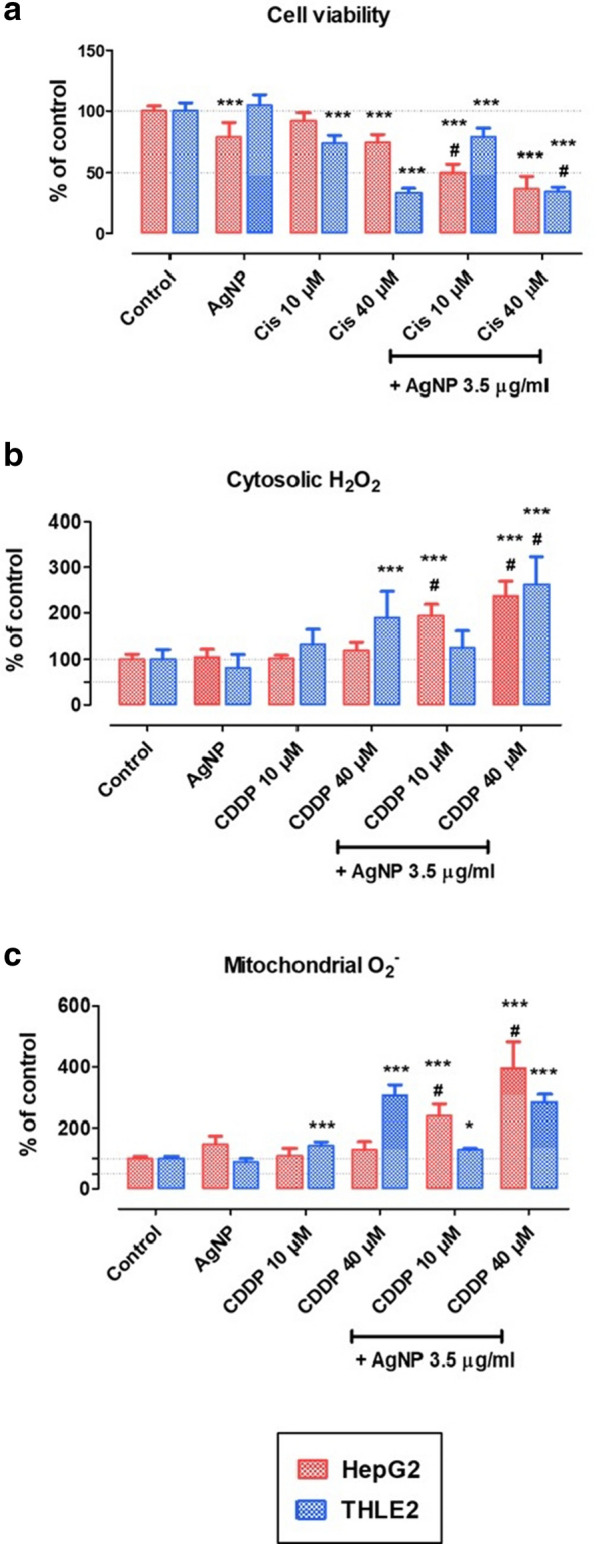
Biological endpoints. **a** Cell viability, **b** cytosolic hydrogen peroxide levels, and **c** mitochondrial superoxide levels in HepG2 and THLE2 cells exposed for 24 h to AgNPs (3.5 µg/mL), CDDP (10 or 40 µM), or AgNPs/CDDP (3.5 µg/mL and 10 or 40 µM CDDP). Mean + SD of three independent experiments in triplicate. Asterisks indicate difference in comparison to the control (*p < 0.05, ***p < 0.001); sharp symbol (#) indicates toxicological interaction

We evaluated the effects of AgNPs, CDDP, and corresponding combinations on cell viability and ROS levels, in both tumoral (HepG2) and normal (THLE2) cell lines after 24 h of exposure (Fig. 3).

We assessed the cytotoxicity of AgNPs, CDDP and AgNPs/CDDP by an MTT metabolism assay (Fig. 3a). Single exposure with AgNPs was more harmful to HepG2 cells (≈ 25% viability loss) than to THLE2 cells (no viability loss). In contrast, CDDP had more of an effect on THLE2 cells than HepG2 cells: 10 µM CDDP led to 10% viability loss in HepG2 cells and ≈ 25% loss in THLE2 cells, while 40 µM CDDP led to ≈ 25% viability loss in HepG2 cells and ≈ 70% viability loss in THLE2 cells. The combinatorial effect of AgNPs/CDDP led to a higher toxicity than single exposures for both cell lines and we observed toxicological interaction in most groups. Particularly interesting was the exposure with AgNPs and the lower concentration of CDDP (10 µM). Whereas the combination of drugs led to a 50% decrease in the viability of HepG2 cells, normal THLE2 cells were notably less hindered, losing ≈ 20% of their viability. Using a higher concentration of CDDP (40 uM) equalized the response to the exposure between the cell lines, thus exceeding the threshold for hindering the cancerous cell line compared to the normal cell line.

We quantitated ROS levels in the cytosol and mitochondria (Fig. 3b, c). Single exposures with AgNPs and 10 µM CDDP increased ROS in neither HepG2 nor THLE2 cells. Exposure with 40 µM CDDP was toxic to THLE2 cells, leading to a 90% increase in cytosolic H_2_O_2_ (Fig. 2b) and a 190% increase in mitochondrial superoxide (Fig. 2c). Combinations of AgNPs and CDDP induced a toxicological interaction in HepG2 cells, whereas normal cells were resistant to the combination with the lowest CDDP concentration. Thus, regarding biological endpoints, cell viability and ROS levels, tumoral HepG2 cells were more sensitive to the combined exposure of AgNPs and 10 µM CDDP than normal THLE2 cells.

Due to their fast metabolism, tumoral cells generate higher ROS levels compared to normal cells. Although this characteristic is protumorigenic, it also renders tumoral cells more susceptible to oxidative stress-induced damage than normal cells, once a tolerance level is reached sooner [24]. Researchers have well-documented that an increased level of ROS is one of the main outcomes after exposure to AgNPs. Therefore, it is possible that co-exposure with AgNPs contributed to the high toxicity observed in tumoral HepG2 cells treated with AgNPs/CDDP. However, normal THLE2 cells maintained ROS at tolerable levels and resisted initiating cell death when exposed to AgNPs and a non-cytotoxic concentration of CDDP (10 mM). In accordance with our findings, a recent study has shown that tumoral HepG2 cells undergo higher levels of apoptosis than normal LO2 cells after exposure with AgNPs and paclitaxel, which was related to oxidative stress [25]. These findings support that normal cells are more resistant to ROS-mediated cell damage than tumoral cells, upon exposure with AgNPs.

### Influence of AgNPs on CDDP cellular uptake

**Fig. 4:**
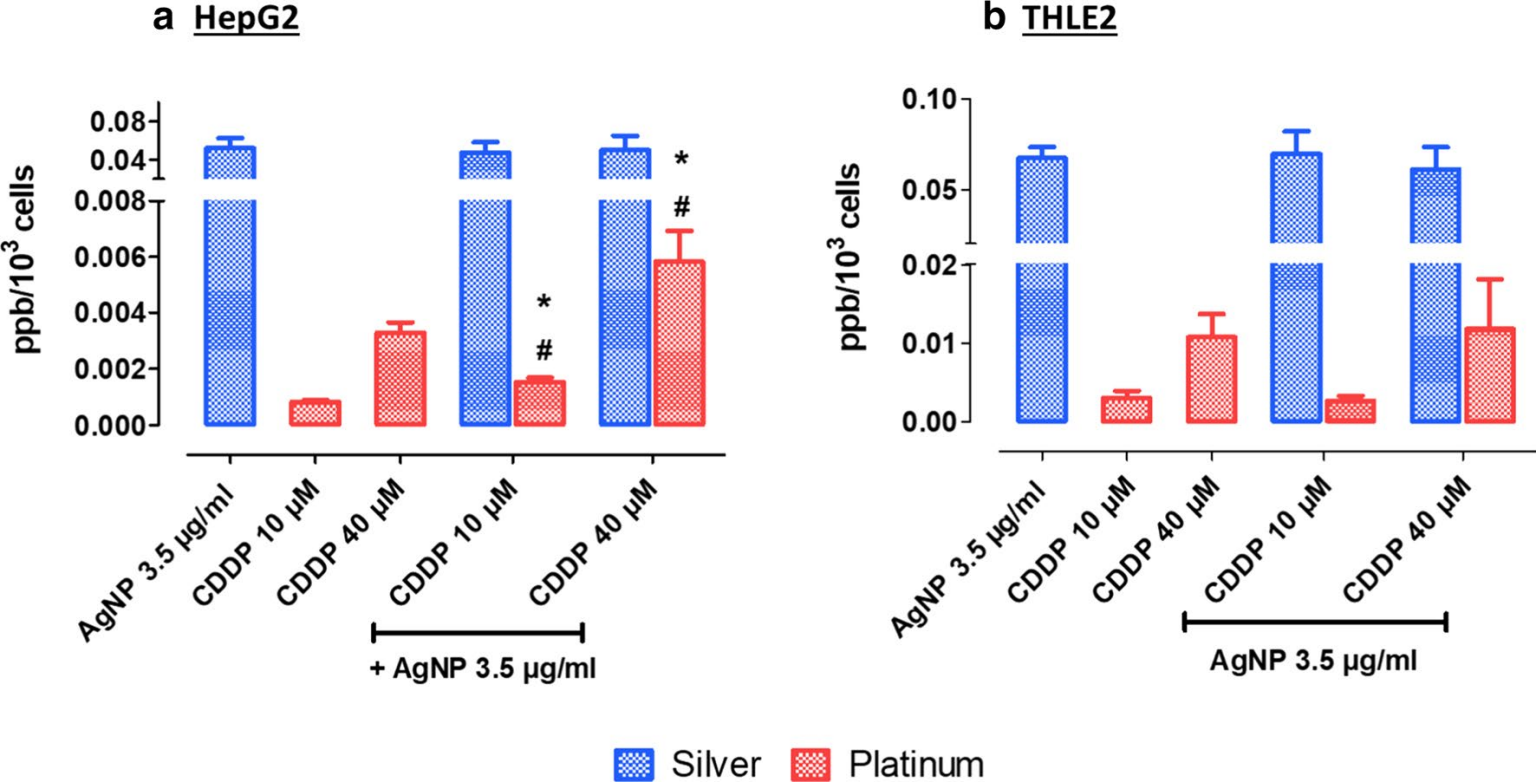
Intracellular concentrations of Ag and Pt in **a** HepG2 cells and **b** THLE2 cells exposed for 4 h. Results are expressed in ppm/10^3^ cells. Mean + SD of three independent experiments. Asterisks indicate difference in comparison to the correspondent metal uptake for single exposures (*p < 0.05); sharp symbol (#) indicates toxicological interaction

We performed ICP–MS analysis to investigate whether the higher toxicity observed in cells exposed to combinations of AgNPs and CDDP correlated to intracellular uptake, by measuring concentrations of Ag and Pt. Analysis in HepG2 cells revealed that the Pt concentration doubled in the presence of AgNPs (Fig. 4a). In contrast, we observed no significant changes in intracellular concentrations in neither Ag nor Pt after the combined exposure of AgNPs/CDDP in THLE2 cells (Fig. 4b).

These results suggest that the toxicological interactions observed after combinatorial exposure in HepG2 may, at least in part, be explained by increased CDDP intracellular accumulation. Based on previous studies, we hypothesize that increased levels of CDDP in HepG2 cells might be related to multidrug resistance transporter activity. Analogously to several antineoplastic drugs, multidrug resistance transporters pump CDDP to the extracellular environment, and thus play an important role in tumor resistance to chemotherapy [26]. AgNPs can interfere with the activity and expression of these proteins [13, 14], which could possibly lead to intracellular accumulation of chemotherapy drugs.

### Proteomics analysis

**Fig. 5:**
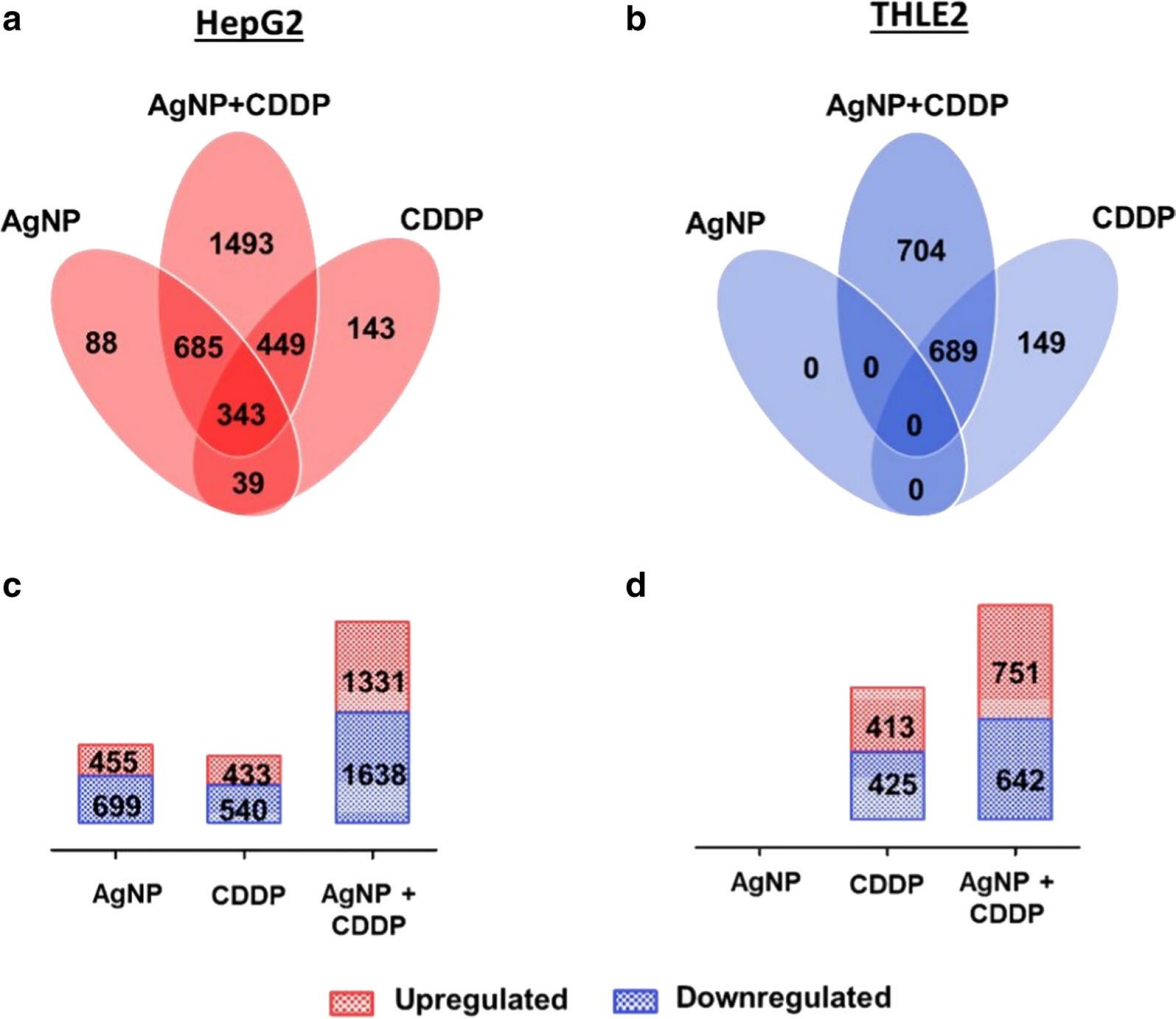
Quantitative data of proteomic analysis of three independent experiments, after both cell lines were exposed for 24 h to AgNPs (3.5 µg/mL), CDDP (10 µM) and AgNPs/CDDP (3.5 µg/mL + 10 µM). We quantitated a total of 5,694 proteins in HepG2 cells and 6,380 proteins in THLE2 cells, in all experimental conditions. We built Venn diagrams to examine the profiles of protein deregulation by overlapping the exposure conditions in **a** HepG2 cells and **b** THLE2 cells. **c** Regulated proteins in HepG2 cells. **d** Regulated proteins in THLE2 cells

To identify molecular pathways involved in the cellular outcomes induced by AgNPs/CDDP (3.5 µg/mL + 10 µM), we conducted quantitative proteomics after 24 h of exposure. For HepG2 and THLE2 cells, we quantitated 5,694 and 6,380 proteins, respectively, with at least three unique peptides based on iTRAQ reporter ion intensities. We considered only proteins with *p*-values less than 0.01 to be regulated (Fig. 5 and Additional file 1: Figure S1).

In this study, upregulated refers to proteins that were more abundant in comparison to the control, whereas downregulated refers to proteins that were less abundant compared to the control. Figure 5 shows the number of deregulated proteins after 24 h of exposure with AgNPs, CDDP and their corresponding combinations in HepG2 and THLE2 cells (for details on protein ID and quantitation see Additional files 2 and 3). Exposure with AgNPs resulted in 1,154 deregulated proteins, of which 455 were upregulated and 699 were downregulated in HepG2 cells, whereas in THLE2 cells no proteins were deregulated. Exposure with CDDP resulted in 433 upregulated and 540 downregulated proteins for HepG2 cells; for THLE2 413 proteins were upregulated and 425 proteins were downregulated. A combination of these two substances resulted in deregulation of 2,969 proteins (≈ 50% of the measured proteome) in HepG2 cells, of which 1,331 were upregulated and 1,638 were downregulated. THLE2 cells also exhibited deregulation of several proteins, accounting for a total of 1,393 proteins (≈ 20% of the proteome), of which 751 were upregulated and 642 were downregulated.

For both cell lines, proteome changes followed a similar trend as that observed in cell viability assays (Fig. 3a). Specifically, the proteome deregulation observed after AgNPs/CDDP exposure was larger than the sum of the two single exposures. However, this effect was substantially enhanced in HepG2 cells compared to THLE2 cells.

### AgNPs/CDDP combination affects similar pathways and upstream regulators in HepG2 and THLE2 cells

To reveal the main molecular pathways disturbed after 24-h exposures for both cell lines, we performed canonical pathway and upstream regulator analyses, using IPA software.

**Fig. 6:**
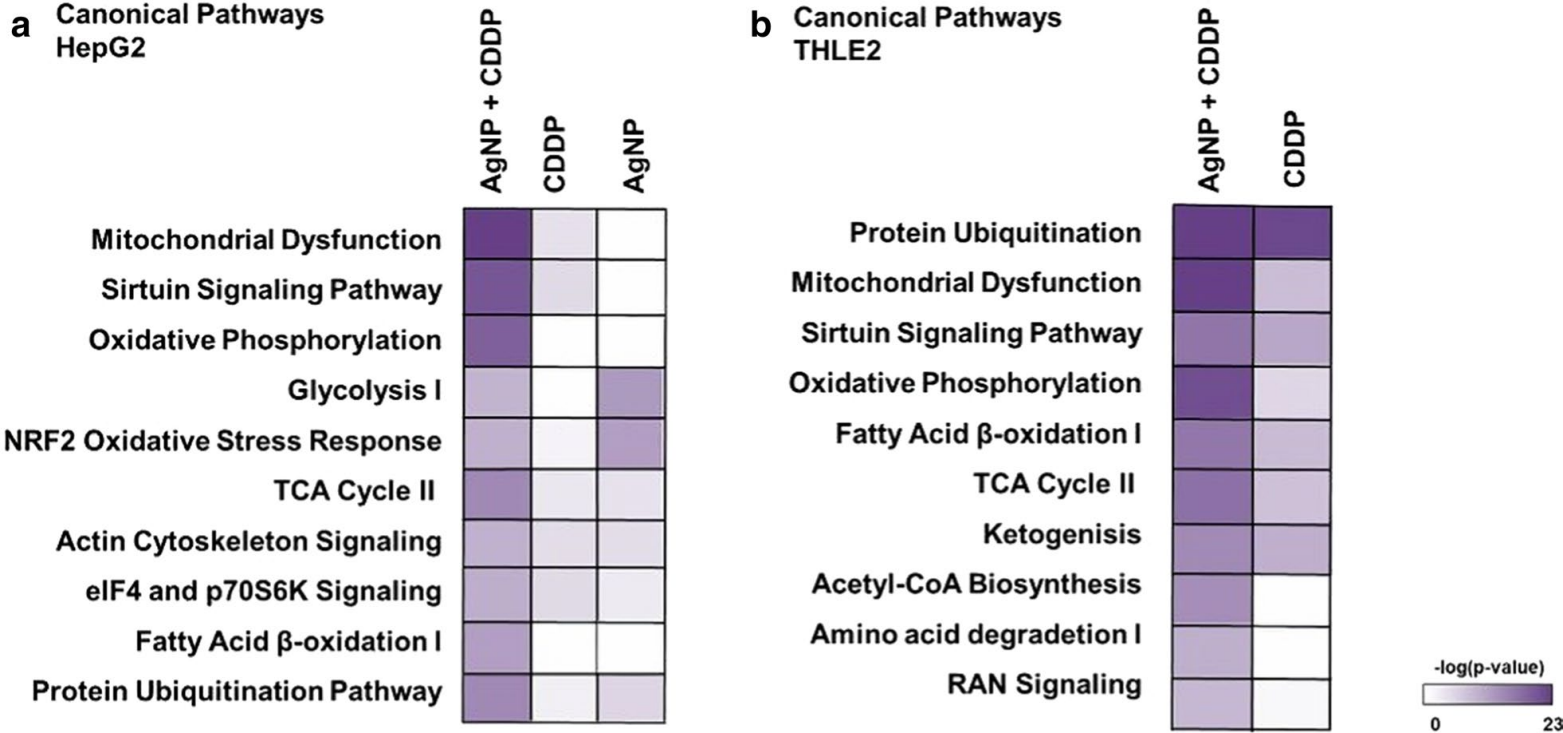
Ingenuity pathway analysis, a canonical pathway analysis. Top 10 significantly affected [− log(*p* value) ≥ 1.3] pathways in **a** HepG2 cells and **b** THLE2 cells after 24 h of exposure to AgNPs/CDDP. The corresponding −log(*p*-value) of these canonical pathways is also represented for single exposures

For HepG2 cells (Fig. 6a), among the top 10 canonical pathways affected by AgNPs/CDDP, half were related to energy metabolism (mitochondrial dysfunction, oxidative phosphorylation, glycolysis, the TCA cycle and fatty acid β-oxidation) and two were related to stress resistance (Nrf-2 regulation and the sirtuin signaling pathway). The actin cytoskeleton, cell signaling and protein ubiquitination canonical pathways were also significantly disturbed. Regarding single exposures, AgNP outcomes were mostly associated with the response to oxidative stress and energy metabolism (glycolysis and the TCA cycle), whereas exposure with CDDP was also associated with energy metabolism (mitochondrial dysfunction and oxidative phosphorylation), as well as the cytoskeleton and stress response (the sirtuin signaling pathway).

For THLE2 cells (Fig. 6b), it is notable that CDDP and AgNPs/CDDP disturbed similar pathways. The majority of the top 10 canonical pathways were associated with energy metabolism (mitochondrial dysfunction, oxidative phosphorylation, the TCA cycle, fatty acid β-oxidation, ketogenesis, acetyl co-A biosynthesis and amino acid degradation); protein ubiquitination, stress resistance and RAN signaling pathways were also affected.

**Fig. 7:**
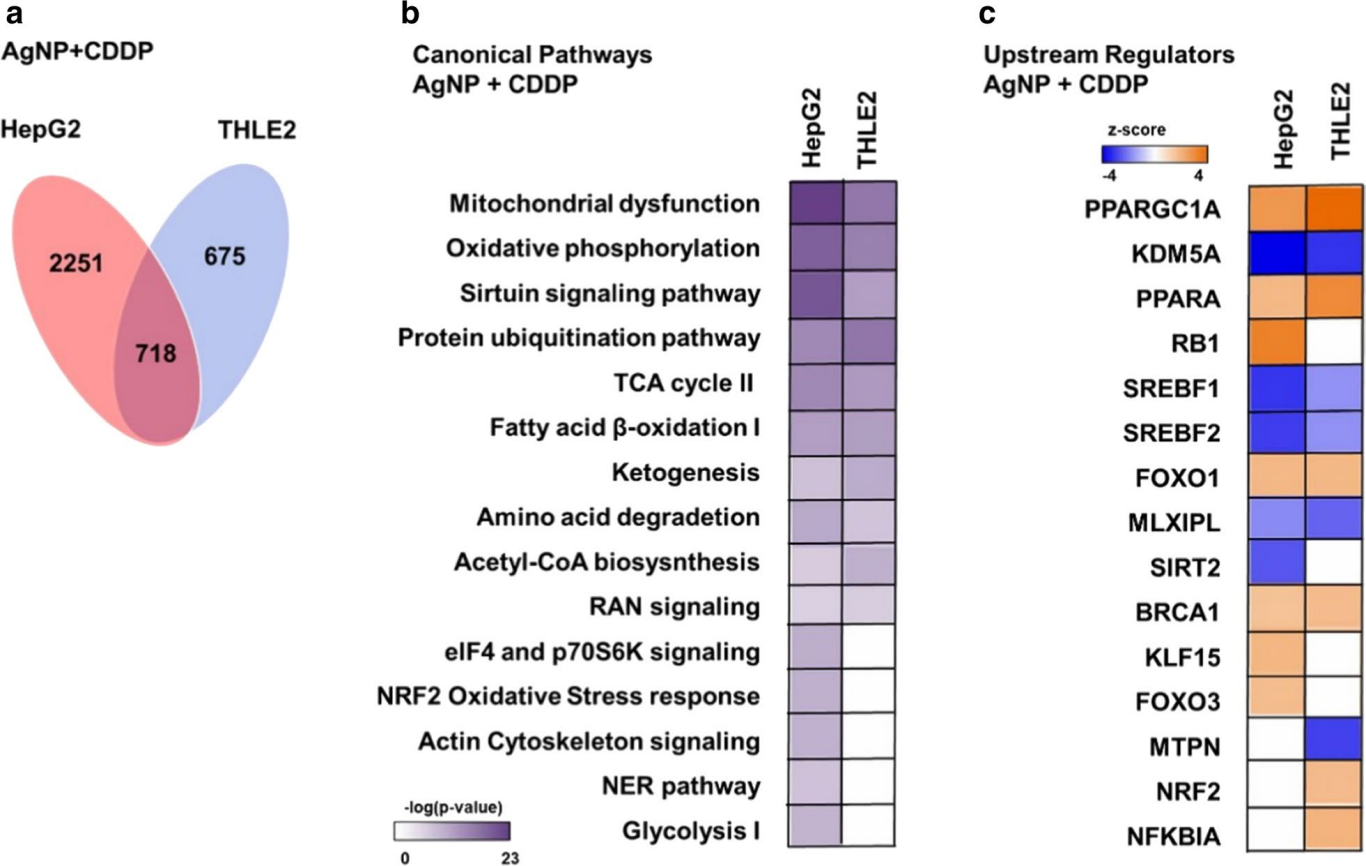
Venn diagram built to show proteins deregulated by AgNPs/CDDP overlap in both cell lines. **a** Number of proteins uniquely deregulated: HepG2 cells, 2251 proteins; THLE2 cells, 675 proteins; 718 proteins were in common deregulated in both cell lines. **b** Comparison of main significantly affected [− log(*p*-value) ≥ 1.3] canonical pathways between HepG2 and THLE2 cell lines, after AgNPs/CDDP exposure, indicated by IPA software. **c** Top active (*z*-score ≥ 2) and inactive (*z*-score ≤ − 2) upstream regulators in HepG2 and THLE2 cells after 24 h of exposure to AgNPs/CDDP, indicated by IPA software

We compared the top canonical pathways of both cell lines exposed to AgNPs/CDDP (Fig. 7b). Similar pathways were affected in both cell lines, except for glycolysis, the nucleotide excision and repair (NER) pathway, the NRF2 stress response pathway, actin and eif4 signaling, which were only significantly disturbed in HepG2 cells.

Figure 7c shows upstream regulators (*z*-score ≥ 2 or ≤ − 2) involved in protein deregulation following exposure to AgNPs/CDDP. Eight upstream regulators followed the same activation pattern in both cell lines after exposure with AgNPs/CDDP. Among these, six are related to energy metabolism (PPARGC1A, PPARA, SRBP1, SRBP2, MLXIP, and FOXO1), whereas others are related to the DNA damage response and cell cycle arrest [breast cancer type 1 susceptibility protein (BRCA1)], and histone demethylase (KDM5A).

Activated upstream regulators upon AgNPs/CDDP exposure that we observed only in HepG2 cells are RB1, which is a key regulator of cell cycle arrest; FOXO3, which is a transcriptional activator involved in apoptosis regulation in response to oxidative stress; and KLF15. SIRT2 was the only inhibited upstream regulator that we observed exclusively in HepG2 cells; this protein has a central role in cell cycle progression and genomic stability.

For THLE2 cells, NRF2 (a key transcriptional activator of genes related to the response to oxidative stress) and NFKBIA (an inhibitor of the activity of the complex NF–Kβ/REL) were indicated as active upstream regulators after AgNPs/CDDP exposure. MTPN (promotes dimerization of NF–Kβ subunits, regulates corresponding transcriptional activity, and has a role in actin filament dynamics) was indicated as an inhibited upstream regulator.

AgNPs/CDDP exposure altered similar pathways and upstream regulators in both cell lines. Energy metabolism, response to oxidative stress and regulation of cell fate were common targets after co-exposure.

### AgNPs/CDDP exposure induces energy metabolism adaptation

**Fig. 8:**
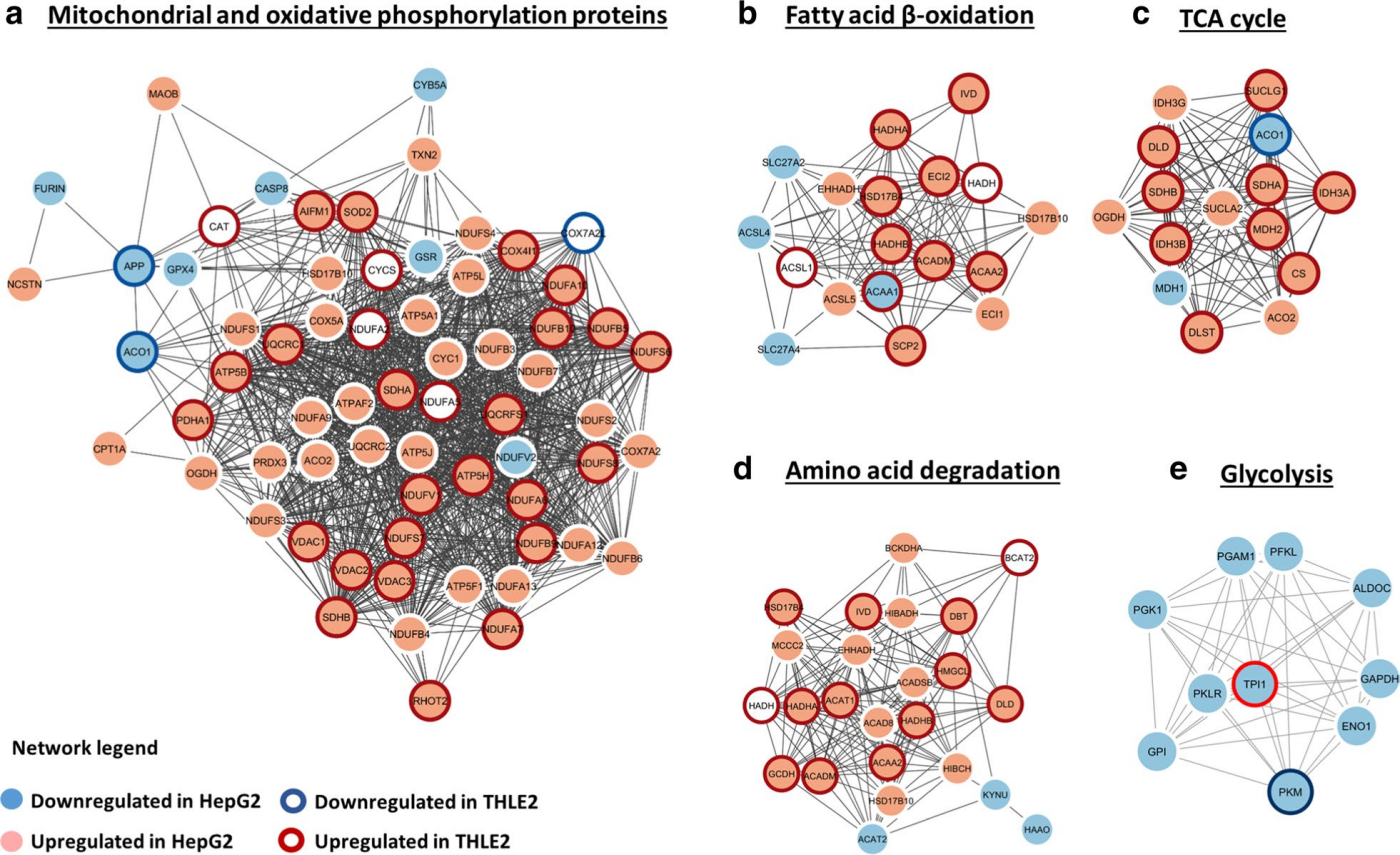
Protein–protein interaction networks of energy metabolism canonical pathways affected by AgNPs/CDDP exposure in HepG2 and THLE2 cells. We built functional interaction networks of deregulated proteins of each pathway with the STRING algorithm. Lines represent interactions and only interactions with medium confidence (score ≥ 0.4) are shown. Upregulated proteins in HepG2 cells are light red, whereas downregulated proteins are light blue. For THLE2 cells, a dark red halo around a protein indicates that the protein is upregulated, whereas a dark blue halo indicates that the protein is downregulated

The metabolism of tumoral cells differs substantially from normal cells because the metabolism of the former is adapted for fast growth in hypoxic and acidic environments. Therefore, glycolysis is the preferred pathway to synthesize ATP, even in the presence of oxygen and functional mitochondria, because glycolysis generates energy more rapidly than oxidative phosphorylation [27, 28]. For this reason, cancer energy metabolism has gained attention in relation to cancer treatment. The glycolysis pathway was significantly enriched in HepG2 cells, with all main proteins involved being downregulated after exposure to AgNPs alone and AgNPs/CDDP (Figs. 6a and 8e, and Additional file 1: Table S1). This information shows that AgNPs impair an important cancer hallmark and that combining them with CDDPs increases this effect somewhat further.

In proliferating normal or tumoral cells, glucose is an essential nutrient for supplying cells with energy [29, 30]. However, under nutrient deprivation, alternative energy sources such as fatty acids, glutamine and proteins may be oxidized through the TCA cycle and fatty acid β-oxidation to generate ATP [31, 32]. NADH and FADH_2_ generated in these processes are used during oxidative phosphorylation to reduce molecular oxygen to water and generate ATP.

The TCA cycle, fatty acid β-oxidation, amino acid degradation and oxidative phosphorylation canonical pathways were significantly affected after AgNPs/CDDP exposure in both cells (Fig. 7b); the majority of the proteins related to these pathways were upregulated (Fig. 8a–d). These outcomes suggest that both cell lines may have used alternative nutrients to supply metabolic demand, such as the lipids and amino acids present in the complete culture medium.

In HepG2 cells, not only did AgNPs/CDDP hinder glycolysis, the combination also led to decreased GLUT 1 levels (see Additional file 2: page 98, accession number P11166), the main glucose carrier in HepG2 [33]. An inability to efficiently take up and metabolize glucose through glycolysis may have induced the cells to adapt and increase other energy-generating pathways. This adaptation, however, was insufficient to avoid cell death. For THLE2 cells, metabolic adaptation may have contributed to a lower percentage of cell death (20%) compared to HepG2 cells (50%), after exposure with AgNPs/CDDP (Fig. 3a).

Peroxisome proliferator-activated receptor alpha (PPARA) and peroxisome proliferator-activated receptor gamma coactivator 1-a (PPARGC1A) are active upstream regulators in both cell lines after AgNPs/CDDP exposure. PPARA is a key regulator of lipid metabolism, in particular, peroxisomal fatty acid β-oxidation [34]; whereas PPARGC1A plays an essential role in metabolic reprogramming in response to nutrient availability, coordinating the expression of genes involved in glucose and fatty acid metabolism [35]. Therefore, activation of these upstream regulators may be related to the upregulation of the TCA cycle and fatty acid β-oxidation pathways.

Key regulators of lipid synthesis, sterol regulatory element-binding protein (SREBP-1 and 2), and carbohydrate-responsive element-binding protein (MLXIPL, also known as ChREBP) were inhibited in both cell lines after AgNPs/CDDP exposure. These transcriptional activators are essential for lipogenesis and its inhibition may be associated with the cellular energetic state [36–38].

Our findings suggest that exposure with AgNPs/CDDP affects energy homeostasis, and the cells respond by increasing alternative nutrient catabolic processes and inhibiting anabolic processes related to energy storage.

### AgNPs/CDDP exposure affects cell proliferation

**Fig. 9:**
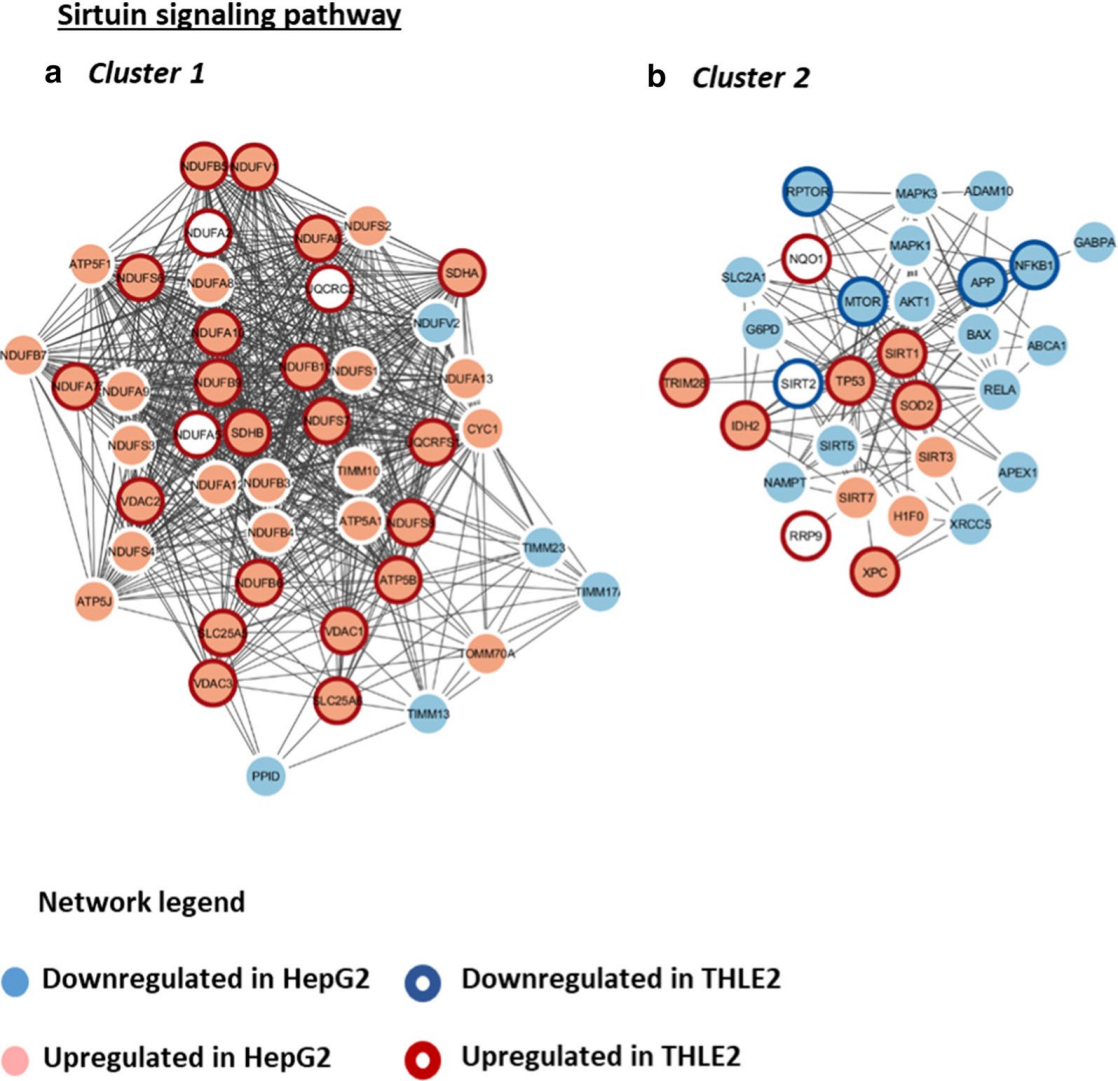
Protein–protein interaction networks of the sirtuin canonical pathway affected by AgNPs/CDDP exposure in HepG2 and THLE2 cells. We built functional interaction networks of deregulated proteins of each pathway with the STRING algorithm. Lines represent interactions and only interactions with medium confidence (score ≥ 0.4) are shown. We used the Markov cluster algorithm (MCL) to identify clusters of tightly connected proteins within the network. Upregulated proteins in HepG2 cells are light red, whereas downregulated proteins are light blue. For THLE2 cells, a dark red halo around a protein indicates that the protein is upregulated, whereas a dark blue halo indicates that the protein is downregulated

The sirtuin signaling pathway regulate many physiological processes, ranging from energy metabolism to epigenetic modifications [39–41]. The pathway was significantly affected in both cell lines after AgNPs/CDDP exposure (Fig. 7b). We grouped proteins associated with this canonical pathway into two main clusters (Fig. 9). Cluster 1 comprises several upregulated proteins associated with mitochondrial energy metabolism, whereas cluster 2 comprises several downregulated proteins related to cell cycle control such as MAPK 1 and 3, AKT1, MTOR, RPTOR (most of which were only deregulated in HepG2 cells) and the tumor suppressor TP53, which is upregulated in both cells.

Two upstream regulators related to cell cycle control exhibited similar activation states in both cell lines after AgNPs/CDDP exposure. BRCA1, an E3 ubiquitin-protein ligase that acts as a tumor suppressor, is involved in DNA damage repair and promotes cell cycle arrest in response to DNA damage [42, 43]. Lysine-specific demethylase 5A (KDM5A, also termed RBP2) is a potential oncogene that is highly expressed in many different cancers [44]. Due to its demethylase activity, this protein activates cell growth by decreasing the expression of cell cycle inhibitors and affecting cell cycle arrest by forming complexes with multiple proteins to regulate transcriptional activation [45, 46]. BRCA1 and KDM5A are, respectively, activated and inactivated in both cell lines after AgNPs/CDDP exposure. This suggests that both cells lines responded to DNA damage and arrested cell proliferation. For HepG2 cells, although BRAC1 is activated and possibly stimulates DNA repair, the NER pathway was significantly disturbed (Fig. 7b). Several proteins belonging to this pathway were downregulated (Additional file 1: Table S2) after co-exposure, thus possibly leading to genomic instability and consequently reduced cell viability.

In addition to BRCA1 and KDM5A, retinoblastoma-associated protein (RB1) was also an active upstream regulator in HepG2 cells after AgNPs/CDDP exposure. The non-phosphorylated active form of this protein acts as a tumor suppressor by limiting the transcription of cell cycle genes, mainly via regulation of the E2F transcription factor [47].

Our data suggests that both cell lines inhibited anabolic processes such as cell proliferation and stimulated catabolic processes dramatically to overcome the energetic stress induced by AgNPs/CDDP exposure.

### Oxidative stress is enhanced in tumoral cells after AgNPs/CDDP exposure

Oxidative stress results from an imbalance between ROS and antioxidant defenses, to detoxify ROS intermediates or repair the resulting damage. Cells respond by increasing the transcription and activity of antioxidant proteins, and by activating pathways to promote cell survival and manage with the stress response [48]. NRF2 is a transcription factor that regulates basal and inducible expression of antioxidant response genes, thus playing a key role in cellular redox homeostasis. The cytoplasmic protein Keap1 interacts with NRF2 and represses its function under homeostatic conditions. When intracellular ROS levels increase, this complex dissociates and NRF2 translocates into the nucleus, promoting transcription of antioxidant genes [49, 50].

**Fig. 10:**
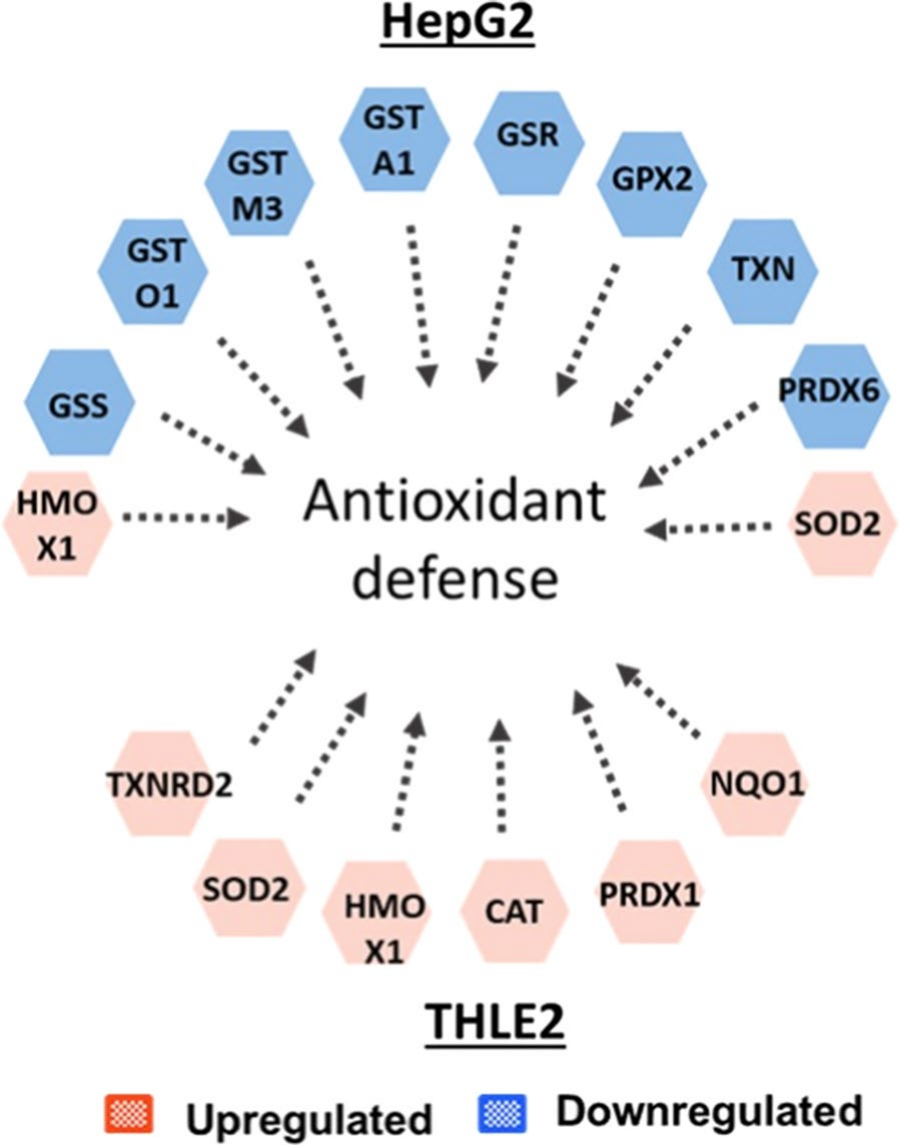
Antioxidants protein deregulated after exposure with AgNPs/CDDP in HepG2 and THLE2 cells

According to our findings, this is how normal THLE2 cells respond to co-exposure to AgNPs/CDDP. Although the NRF2 pathway was not significantly affected by the co-exposure, the transcription factor NRF2 was activated in these cells (Fig. 7). This activation possibly led to upregulation of several antioxidant proteins, such as NAD(P)H:quinone acceptor oxidoreductase 1 (NQO1), heme oxygenase-1 (HMOX1) and thioredoxin reductase (TXNRD2). Catalase, peroxirredoxin (both of which are important enzymes that help reduce hydrogen peroxide) and superoxide dismutase (destroys superoxide anion radicals) were also upregulated (Fig. 10). These outcomes might have resulted in the maintenance of ROS at control levels after exposure with AgNP + 10 µM CDDP (Fig. 3b, c) and the low cytotoxicity (Fig. 3a).

AgNPs and AgNPs/CDDP induced a different response in HepG2 cells. In these cases, the NRF2-mediated stress response was significantly affected (Fig. 6a, and Additional file 1: Table S3). For AgNPs/CDDP exposure, downregulation of several antioxidant proteins occurred (Fig. 10); in particular, the proteins involved in the metabolism of glutathione, one of the main intracellular ROS scavengers. These were glutathione synthetase (GSS), glutathione S-transferase (GSTA1, GSTO1, and GSTM3) and glutathione peroxidase (GPX). Thioredoxin (TXN) was also downregulated after AgNPs/CDDP exposure. This enzyme participates in protein repair after oxidative damage and is responsible for reducing oxidized proteins, including peroxirredoxin, which was also downregulated.

The reduction of the antioxidant pool is possibly related to the increased ROS levels induced by AgNPs alone and together with CDDP (Fig. 3b, c), and may play a key role in decreased cell viability (Fig. 3a).

## Conclusions

Our results demonstrate the efficacy of AgNPs as a combinatorial agent to enhance the biological effect of CDDP, in both tumoral and normal cells. We performed quantitative MS-based proteomic, metal quantification, and biological endpoints assays to achieve deeper understanding on the effect of this combination in HepG2 and THLE2 cells. Biochemical endpoints showed that the toxicological interaction of AgNPs/CDDP occurred in both cell lines; however, the effect was more pronounced in HepG2 cells. This might, at least to some extent, be related to increased CDDP intracellular accumulation after the co-exposure. Proteomics analyses revealed that energy metabolism-related pathways were significantly affected in both cell lines. Upregulation of proteins related to the TCA cycle, fatty acid β-oxidation, amino acid degradation and oxidative phosphorylation indicates that both cells lines possibly underwent energy stress due to AgNPs/CDDP exposure, and alternative nutrient degradation pathways were activated to supply cellular ATP demand. It is possible that energetic demand was higher for HepG2 cells due to downregulation of main glycolytic proteins. Canonical pathways and upstream regulators related to cell proliferation showed protein downregulation and inactivation, respectively, indicating reduced cell proliferation. The oxidative stress response differed in both cell lines. Whereas a downregulation of antioxidant proteins occurred in HepG2 cells, THLE2 cells exhibited upregulation of its antioxidant defense system. Although both cell lines were hindered by AgNPs/CDDP exposure, the response of the normal THLE2 cells was more successful in avoiding cell death than that of HepG2 cells. Taken together, our results indicate that a combination of AgNPs and CDDP could be further explored for future oncotherapies.

## Supplementary information

**Additional file 1: Figure S1.** Volcano plots of the comparisons between control and exposure groups in HepG2 (A–C) and THLE2 (D–F) cells. **Table S1.** Glycolysis related proteins differentially deregulated after 24 h-exposure to AgNP, CDDP or AgNP/CDDP in HepG2 and THLE2 cells. **Table S2.** NER pathway related proteins differentially deregulated after 24 h-exposure to AgNP/CDDP in HepG2 and THLE2 cells. **Table S3.** Oxidative stress response pathway related proteins differentially deregulated after 24 h-exposure to AgNP/CDDP in HepG2 and THLE2 cells.

**Additional file 2.** List of proteins deregulated in HepG2 cells after 24 h exposure to AgNP, CDDP and AgNP/CDDP.

**Addiional file 3.** List of proteins deregulated in THLE2 cells after 24 h exposure to CDDP and AgNP/CDDP.

## Data Availability

The datasets used and/or analysed during the current study are available from the corresponding authors on reasonable request.
