# Supplementary material for: Elucidating the cellular response of silver nanoparticles as a potential combinatorial agent for cisplatin chemotherapy
Source: J Nanobiotechnology. 2020 Nov 10;18:164. doi: 10.1186/s12951-020-00719-x (PMC7654574; doi:10.1186/s12951-020-00719-x)
Supplement: Supplementary file 1 — Additional file 1: Figure S1. Volcano plots of the comparisons between control and exposure groups in HepG2 (A–C) and THLE2 (D–F) cells. Table S1. Glycolysis related proteins differentially deregulated after 24 h-exposure to AgNP, CDDP or AgNP/CDDP in HepG2 and THLE2 cells. Table S2. NER pathway related proteins differentially deregulated after 24 h-exposure to AgNP/CDDP in HepG2 and THLE2 cells. Table S3. Oxidative stress response pathway related proteins differentially deregulated after 24 h-exposure to AgNP/CDDP in HepG2 and THLE2 cells. [file 12951_2020_719_MOESM1_ESM.docx]

**Additional File_1**

**Elucidating the cellular response of silver nanoparticles as a potential combinatorial agent for cisplatin chemotherapy**

Renata Rank Miranda^a^, Micaella Pereira da Fonseca^a^, Barbara Korzeniowska^a^, Lilian Skytte^b^, Kaare Lund Rasmussen^b^ and Frank Kjeldsen^a^

*^a^Department of Biochemistry and Molecular Biology, University of Southern Denmark, Odense, Denmark*

*^b^Department of Physics, Chemistry and Pharmacy, University of Southern Denmark, Odense, Denmark*

**Figure 1.** Volcano plots of the comparisons between control and exposure groups in HepG2 (A-C) and THLE2 (D-F) cells. X-axis: mean log-ratios. Y-axis: statistical significance, p-values on the negative log10 scale. The dashed line represents the p-value cutoff of 0.01. Blue dots represent significantly downregulated proteins in comparison to control, while pink dots represent significantly upregulated proteins in comparison to control.

**Table 1.** Glycolysis related proteins differentially deregulated after 24 h-exposure to AgNP, CDDP or AgNP/CDDP in HepG2 and THLE2 cells. Results express experimental group *vs* control mean log-ratio of three independent experiments. Table extracted from IPA.

| **Symbol** | **Gene name** | **Accession number** | **HepG2** | | | **THLE2** | | |
| --- | --- | --- | --- | --- | --- | --- | --- | --- |
|  |  |  | **AgNP** | **CDDP** | **AGNP + CDDP** | **CDDP** | **AGNP + CDDP** |  |
| **GPI** | Glucose 6 phosphate isomerase | P06744 | -0.18 |  | -0.22 |  |  |  |
| **PFKL** | Phosphofructokinase 1 | P17858 | -0.22 |  | -0.18 |  |  |  |
| **ALDO C** | Fructose-bisphosphate aldolase C | P09972 | -0.18 |  | -0.238 |  |  |  |
| **TPI** | Triphosphate isomerase | P60174 | -0.19 |  | -0.18 | 0.36 | 0.23 |  |
| **GAPDH** | Glyceraldehyde-3-phosphate dehydrogenase | O14556 | -0.17 |  | -0.18 |  |  |  |
| **ENO1** | Alpha enolase | P06733 |  |  | -0.26 |  |  |  |
| **PKLR** | Pyruvate kinase | P30613 | -0.33 | -0.27 | -0.45 |  |  |  |
| **PKM** | pyruvate kinase M1/2 | P14618 |  |  | -0.136 |  | -0.15 |  |
| **PGK1** | phosphoglycerate kinase 1 | P00558 | -0.15 |  | -0.125 | 0.15 |  |  |
| **PGAM1** | phosphoglycerate mutase 1 | P18669 | -0.18 |  | -0.154 |  |  |  |

**Table 2.** NER pathway related proteins differentially deregulated after 24 h-exposure to AgNP/CDDP in HepG2 and THLE2 cells. Results express experimental group *vs* control mean log-ratio of three independent experiments. Table extracted from IPA.

| **Symbol** | **Gene name** | **Accession number** | **AgNP + CDDP** | | |
| --- | --- | --- | --- | --- | --- |
|  |  |  | **HepG2** | **THLE2** | |
| **CCNH** | \| Cyclin H \| \| --- \| | P51946 | 0.34 | |  |
| **CETN2** | Centrin 2 | P41208 | -0.169 | | 0.21 |
| **COPS5** | \| Signalosome subunit 5 \| \| --- \| | Q92905 | -0.176 | |  |
| **COPS8** | Signalosome subunit 8 | Q99627 | -0.126 | |  |
| **CUL4A** | \| Cullin 4ª \| \| --- \| | Q13619 | -0.096 | |  |
| **DDB1** | Damage specific DNA binding protein 1 | Q16531 | -0.172 | |  |
| **DDB2** | Damage specific DNA binding protein 2 | Q92466 | 0.393 | | 0.49 |
| **GTF2H1** | General transcription factor IIH subunit 1 | P32780 |  | | 0.239 |
| **NEDD8** | NEDD8 ubiquitin like modifier | Q15843 | -0.262 | |  |
| **PCNA** | Proliferating cell nuclear antigen | P12004 | 0.126 | |  |
| **POLA2** | \| DNA polymerase alpha 2, accessory subunit \| \| --- \| | Q14181 | -0.173 | |  |
| **POLD2** | DNA polymerase delta 2, accessory subunit | P49005 | -0.341 | |  |
| **POLE** | DNA polymerase epsilon, catalytic subunit | Q07864 | -0.277 | |  |
| **POLE3** | DNA polymerase epsilon 3, accessory subunit | Q9NRF9 | -0.155 | |  |
| **POLR2H** | RNA polymerase II subunit H | P52434 | -0.403 | | -0.346 |
| **POLR2I** | RNA polymerase II subunit I | P36954 |  | | -0.317 |
| **POLR2J** | RNA polymerase II subunit J | P52435 | 0.332 | |  |
| **PRIM1** | DNA primase subunit 1 | P49642 | -0.196 | | -0.184 |
| **RAD23B** | RAD23 homolog B | P54727 | -0.26 | | -0.425 |
| **TOP2A** | DNA topoisomerase II alpha | P11388 | 0.69 | | 0.358 |
| **TOP2B** | DNA topoisomerase II beta | Q02880 | 0.436 | |  |
| **UBE2I** | Ubiquitin conjugating enzyme E2 I | P63279 | -0.189 | |  |
| **UBE2N** | Ubiquitin conjugating enzyme E2 N | P61088 | -0.321 | |  |
| **XPC** | XPC complex subunit | Q01831 | 0.374 | | 0.652 |

**Table 3.** Oxidative stress response pathway related proteins differentially deregulated after 24 h-exposure to AgNP/CDDP in HepG2 and THLE2 cells. Results express experimental group *vs* control mean log-ratio of three independent experiments. Table extracted from IPA.

| **Symbol** | **Gene name** | **Accesion number** | **HepG2** | | | **THLE2** | |
| --- | --- | --- | --- | --- | --- | --- | --- |
|  |  |  | **AgNP** | **CDDP** | **AGNP + CDDP** | **CDDP** | **AGNP + CDDP** |
| **ABCC1** | ATP binding cassette subfamily C member 1 | P33527 | 0.254 |  |  |  |  |
| **AKR1A1** | aldo-keto reductase family 1 member A1 | P14550 | -0.204 |  | -0.203 |  |  |
| **AKR7A2** | aldo-keto reductase family 7 member A2 | O43488 |  |  | -0.204 | 0.182 |  |
| **AKT1** | AKT serine/threonine kinase 1 | P31749 | -0.145 |  | -0.15 |  |  |
| **CAT** | Catalase | P04040 |  |  |  | 0.2 | 0.259 |
| **CBR1** | carbonyl reductase 1 | P16152 |  |  | -0.155 |  |  |
| **CCT7** | chaperonin containing TCP1 subunit 7 | Q99832 |  |  | -0.132 |  |  |
| **CDC34** | cell division cycle 34 | P49427 |  | 0.133 | 0.29 |  |  |
| **CLPP** | caseinolytic mitochondrial matrix peptidase proteolytic subunit | Q16740 | 0.173 |  | 0.28 |  |  |
| **DNAJB1** | DnaJ heat shock protein family (Hsp40) member B1 | P25685 | 0.744 |  | 0.934 | 0.23 | 0.28 |
| **DNAJB11** | DnaJ heat shock protein family (Hsp40) member B11 | Q9UBS4 |  |  | 0.122 |  |  |
| **DNAJB12** | DnaJ heat shock protein family (Hsp40) member B12 | Q9NXW2-2 | 0.278 |  |  |  |  |
| **DNAJB2** | DnaJ heat shock protein family (Hsp40) member B2 | P25686-3 | 0.316 | 0.408 | 0.478 |  |  |
| **DNAJB4** | DnaJ heat shock protein family (Hsp40) member B4 | Q9UDY4 | 0.77 |  | 1.193 |  |  |
| **DNAJB6** | DnaJ heat shock protein family (Hsp40) member B6 | O75190 |  |  |  |  | -0.283 |
| **DNAJB6** | DnaJ heat shock protein family (Hsp40) member B6 | O75190 |  |  |  |  | -0.283 |
| **DNAJC1** | DnaJ heat shock protein family (Hsp40) member C1 | Q96KC8 |  |  |  |  | -0.113 |
| **DNAJC10** | DnaJ heat shock protein family (Hsp40) member C10 | Q8IXB1 |  | -0.118 |  |  |  |
| **DNAJC15** | DnaJ heat shock protein family (Hsp40) member C15 | Q9Y5T4 | 0.309 | -0.695 | -0.583 |  |  |
| **DNAJC19** | DnaJ heat shock protein family (Hsp40) member C19 | Q96DA6 |  |  | 0.398 | 0.3 | 0.384 |
| **DNAJC3** | DnaJ heat shock protein family (Hsp40) member C3 | Q13217 | 0.125 |  |  |  |  |
| **DNAJC7** | DnaJ heat shock protein family (Hsp40) member C7 | Q99615-1 |  |  | 0.117 |  |  |
| **EIF2AK3** | eukaryotic translation initiation factor 2 alpha kinase 3 | Q9NZJ5 |  | -0.222 | -0.305 |  |  |
| **EPHX1** | epoxide hydrolase 1 | P07099 | 0.161 | 0.244 | 0.445 | 0.249 | 0.333 |
| **ERP29** | endoplasmic reticulum protein 29 | P30040 |  |  | 0.186 |  |  |
| **FTH1** | ferritin heavy chain 1 | P02794 |  |  | 0.377 |  | 0.368 |
| **FTL** | ferritin light chain | P02792 | 0.262 | 0.311 | 0.761 | 0.467 | 0.604 |
| **GCLC** | glutamate-cysteine ligase catalytic subunit | P48506 | 0.381 |  | 0.189 |  |  |
| **GCLM** | glutamate-cysteine ligase modifier subunit | P48507 | 0.909 |  | 1.098 |  |  |
| **GPX2** | glutathione peroxidase 2 | P18283 | -0.395 |  | -0.354 |  |  |
| **GSR** | glutathione-disulfide reductase | P00390 |  |  | -0.092 |  |  |
| **GSTA1** | glutathione S-transferase alpha 1 | P08263 | -0.502 |  | -0.468 |  |  |
| **GSTK1** | glutathione S-transferase kappa 1 | Q9Y2Q3 |  |  | 0.246 |  |  |
| **GSTM2** | glutathione S-transferase mu 2 | P28161 | -0.202 |  |  |  |  |
| **GSTM2** | glutathione S-transferase mu 2 | P28161 |  |  |  | 0.198 |  |
| **GSTM3** | glutathione S-transferase mu 3 | P21266 | -0.165 |  | -0.208 |  |  |
| **GSTO1** | glutathione S-transferase omega 1 | P78417 | -0.297 |  | -0.414 | 0.194 |  |
| **GSTP1** | glutathione S-transferase pi 1 | P09211 |  |  |  | 0.222 |  |
| **HACD3** | 3-hydroxyacyl-CoA dehydratase 3 | Q9P035 |  |  | 0.159 |  |  |
| **HERPUD1** | homocysteine inducible ER protein with ubiquitin like domain 1 | Q15011-1 |  | -0.783 | -0.686 |  |  |
| **HMOX1** | heme oxygenase 1 | P09601 | 1.109 |  | 1.422 | -0.177 | 0.236 |
| **HSPB8** | heat shock protein family B (small) member 8 | Q9UJY1 |  |  |  |  | 0.377 |
| **KEAP1** | kelch like ECH associated protein 1 | Q14145 | 0.134 | 0.132 | 0.174 |  |  |
| **MAFF** | MAF bZIP transcription factor F | Q9ULX9 | 0.467 |  | 1.243 | 0.617 | 0.885 |
| **MAFG** | MAF bZIP transcription factor G | O15525 | 0.715 |  | 1.206 |  |  |
| **MAP2K1** | mitogen-activated protein kinase kinase 1 | Q02750 |  |  | -0.321 |  |  |
| **MAP2K2** | mitogen-activated protein kinase kinase 2 | P36507 |  |  | -0.128 |  |  |
| **MAPK1** | mitogen-activated protein kinase 1 | P28482 | -0.272 |  | -0.349 |  |  |
| **MAPK3** | mitogen-activated protein kinase 3 | P27361-1 | -0.233 |  | -0.156 |  |  |
| **MGST1** | microsomal glutathione S-transferase 1 | P10620 |  |  | 0.286 |  |  |
| **MGST2** | microsomal glutathione S-transferase 2 | Q99735 |  |  | 0.09 |  |  |
| **NQO1** | NAD(P)H quinone dehydrogenase 1 | P15559-1 |  |  |  | 0.201 | 0.217 |
| **NQO2** | N-ribosyldihydronicotinamide:quinone reductase 2 | P16083 |  |  |  | 0.225 |  |
| **PMF1/PMF1-BGLAP** | polyamine modulated factor 1 | Q6P1K2-2 |  |  | -0.4 |  |  |
| **PPIB** | peptidylprolyl isomerase B | P23284 |  |  | 0.136 |  | 0.18 |
| **PRDX1** | peroxiredoxin 1 | Q06830 |  |  |  | 0.24 | 0.153 |
| **PRKCA** | protein kinase C alpha | P17252 |  | -0.378 | -0.131 |  | -0.27 |
| **PRKCD** | protein kinase C delta | Q05655 |  |  | 0.1 |  |  |
| **RALA** | RAS like proto-oncogene A | P11233 |  |  | 0.13 |  |  |
| **RALB** | RAS like proto-oncogene B | P11234-2 | 0.208 | 0.246 | 0.355 |  |  |
| **RAP2B** | RAP2B, member of RAS oncogene family | P61225 |  | 0.437 | 0.213 |  |  |
| **RRAS2** | RAS related 2 | P62070-4 | 0.343 |  | 0.363 |  |  |
| **SCARB1** | scavenger receptor class B member 1 | Q8WTV0-2 | -0.167 |  | -0.126 |  |  |
| **SOD2** | superoxide dismutase 2 | P04179 |  | 0.346 | 0.419 |  |  |
| **SOD2** | superoxide dismutase 2 | P04179 |  |  |  | 0.284 | 0.358 |
| **STIP1** |  | P31948-2 |  |  |  |  | -0.17 |
| **TXN** | thioredoxin | P10599-1 |  |  | -0.227 |  |  |
| **TXNRD1** | thioredoxin reductase 1 | Q16881 | 0.246 |  |  |  |  |
| **USP14** | ubiquitin specific peptidase 14 | P54578 |  |  | -0.134 |  |  |
