# Supplementary material for: Elucidating the cellular response of silver nanoparticles as a potential combinatorial agent for cisplatin chemotherapy
Source: J Nanobiotechnology. 2020 Nov 10;18:164. doi: 10.1186/s12951-020-00719-x (PMC7654574; doi:10.1186/s12951-020-00719-x)
Supplement: Supplementary file 2 — Additional file 2. List of proteins deregulated in HepG2 cells after 24 h exposure to AgNP, CDDP and AgNP/CDDP. [file 12951_2020_719_MOESM2_ESM.pdf]

Elucidating the cellular response of silver nanoparticles as a potential combinatorial agent for cisplatin chemotherapy

Additional File 2

List of proteins deregulated in HepG2 cells after 24h exposure to AgNP 3.5 ug/mL………………… pages 1 – 25
List of proteins deregulated in HepG2 cells after 24h exposure to CDDP 10 µM………………… pages 26 – 46
List of proteins deregulated in HepG2 cells after 24h exposure to AgNP 3.5 ug/mL + CDDP 10 µM ………………… pages 47 – 110

List of proteins deregulated in HepG2 cells after 24h exposure to AgNP 3.5 ug/mL

ID	Symbol	Expr Log Ratio	Entrez Gene Name
Q9NQ94	A1CF	-0.202	APOBEC1 complementation factor
P01023	A2M	-0.225	alpha-2-macroglobulin
Q86V21-1	AACS	-0.191	acetoacetyl-CoA synthetase
Q2M2I8	AAK1	0.19	AP2 associated kinase 1
Q9NY61	AATF	0.171	apoptosis antagonizing transcription factor
P33527	ABCC1	0.254	ATP binding cassette subfamily C member 1
Q96IU4	ABHD14B	-0.231	abhydrolase domain containing 14B
Q8TB40	ABHD4	0.417	abhydrolase domain containing 4
P09110-1	ACAA1	-0.182	acetyl-CoA acyltransferase 1
Q13085-4	ACACA	-0.19	acetyl-CoA carboxylase alpha
P49748-3	ACADVL	0.251	acyl-CoA dehydrogenase very long chain
Q9BWD1	ACAT2	-0.344	acetyl-CoA acetyltransferase 2
P21399	ACO1	-0.154	aconitase 1
Q9Y305	ACOT9	0.237	acyl-CoA thioesterase 9
P24666	ACP1	-0.145	acid phosphatase 1
O60488-1	ACSL4	-0.4	acyl-CoA synthetase long chain family member 4
Q9ULC5-3	ACSL5	0.416	acyl-CoA synthetase long chain family member 5
Q9NR19-2	ACSS2	-0.298	acyl-CoA synthetase short chain family member 2
P42025	ACTR1B	-0.393	actin related protein 1B
Q03154-1	ACY1	-0.307	aminoacylase 1
P78536	ADAM17	0.137	ADAM metallopeptidase domain 17
P35611-3	ADD1	-0.247	adducin 1
Q9UEY8-2	ADD3	-0.249	adducin 3
P08319-2	ADH4	-0.355	alcohol dehydrogenase 4 (class II), pi polypeptide
P11766	ADH5	-0.271	alcohol dehydrogenase 5 (class III), chi polypeptide
P28332-2	ADH6	-0.333	alcohol dehydrogenase 6 (class V)
Q9BV57	ADI1	-0.289	acireductone dioxygenase 1
P55263	ADK	-0.28	adenosine kinase
Q9H2P0	ADNP	0.193	activity dependent neuroprotector homeobox
Q16186	ADRM1	0.236	adhesion regulating molecule 1
P30566	ADSL	-0.245	adenylosuccinate lyase
Q9UHB7-1	AFF4	0.172	AF4/FMR2 family member 4
P02771	AFP	-0.245	alpha fetoprotein
O95081	AGFG2	-0.119	ArfGAP with FG repeats 2
Q9UKV8	AGO2	0.163	argonaute RISC catalytic component 2
O00468	AGRN	-0.279	agrin
P01019	AGT	-0.564	angiotensinogen
P23526-1	AHCY	-0.231	adenosylhomocysteinase
P00568	AK1	-0.258	adenylate kinase 1

**Q02952-1**	AKAP12	1.337	A-kinase anchoring protein 12
**Q02040-1**	AKAP17A	0.224	A-kinase anchoring protein 17A
**O43823**	AKAP8	0.182	A-kinase anchoring protein 8
**Q9ULX6**	AKAP8L	0.208	A-kinase anchoring protein 8 like
**P14550**	AKR1A1	-0.204	aldo-keto reductase family 1 member A1
**O60218**	AKR1B10	0.146	aldo-keto reductase family 1 member B10
**P51857**	AKR1D1	-0.229	aldo-keto reductase family 1 member D1
**P31749**	AKT1	-0.145	AKT serine/threonine kinase 1
**P13716-2**	ALAD	-0.293	aminolevulinate dehydratase
**P13196**	ALAS1	0.702	5'-aminolevulinate synthase 1
**P02768**	ALB	-0.414	albumin
**P02769**	ALB	0.709	albumin
**Q8IZ83**	ALDH16A1	-0.165	aldehyde dehydrogenase 16 family member A1
**P00352**	ALDH1A1	-0.274	aldehyde dehydrogenase 1 family member A1
**P09972**	ALDOC	-0.179	aldolase, fructose-bisphosphate C
**Q9Y673**	ALG5	0.142	ALG5 dolichyl-phosphate beta-glucosyltransferase
**Q86V81**	ALYREF	0.209	Aly/REF export factor
**Q9H1A4**	ANAPC1	-0.103	anaphase promoting complex subunit 1
**Q9P2R3-4**	ANKFY1	-0.234	ankyrin repeat and FYVE domain containing 1
**Q9NQW6**	ANLN	0.174	anillin actin binding protein
**Q92688**	ANP32B	-0.174	acidic nuclear phosphoprotein 32 family member B
**P15144**	ANPEP	-0.14	alanyl aminopeptidase, membrane
**P07355-2**	ANXA2	0.479	annexin A2
**P09525**	ANXA4	-0.277	annexin A4
**P08758**	ANXA5	-0.211	annexin A5
**P08133**	ANXA6	-0.304	annexin A6
**Q10567**	AP1B1	-0.135	adaptor related protein complex 1 subunit beta 1
**O43747**	AP1G1	-0.18	adaptor related protein complex 1 subunit gamma 1
**P61966**	AP1S1	-0.139	adaptor related protein complex 1 subunit sigma 1
**P63010-2**	AP2B1	-0.165	adaptor related protein complex 2 subunit beta 1
**O00203**	AP3B1	-0.107	adaptor related protein complex 3 subunit beta 1
**O14617-5**	AP3D1	-0.167	adaptor related protein complex 3 subunit delta 1
**P13798**	APEH	-0.264	acylaminoacyl-peptide hydrolase
**P02647**	APOA1	-0.51	apolipoprotein A1
**P04114**	APOB	-0.435	apolipoprotein B
**P02649**	APOE	-0.248	apolipoprotein E
**O14791-2**	APOL1	-0.318	apolipoprotein L1
**Q6UXV4**	APOOL	0.191	apolipoprotein O like
**P07741**	APRT	-0.221	adenine phosphoribosyltransferase
**O60306**	AQR	0.166	aquarius intron-binding spliceosomal factor
**Q96P48-6**	ARAP1	-0.109	ArfGAP with RhoGAP domain, ankyrin repeat and PH domain 1
**P48444**	ARCN1	-0.099	archain 1
**P84085**	ARF5	-0.175	ADP ribosylation factor 5
**Q9Y6D5**	ARFGEF2	-0.198	ADP ribosylation factor guanine nucleotide exchange factor 2
**P78540**	ARG2	0.22	arginase 2
**Q07960**	ARHGAP1	-0.163	Rho GTPase activating protein 1
**Q8N392**	ARHGAP18	-0.206	Rho GTPase activating protein 18

**Q13017-1**	ARHGAP5	0.252	Rho GTPase activating protein 5
**Q92974**	ARHGEF2	0.374	Rho/Rac guanine nucleotide exchange factor 2
**Q99856**	ARID3A	-0.188	AT-rich interaction domain 3A
**Q8IVW6**	ARID3B	0.209	AT-rich interaction domain 3B
**P40616**	ARL1	-0.133	ADP ribosylation factor like GTPase 1
**P36405**	ARL3	-0.163	ADP ribosylation factor like GTPase 3
**Q6NXE6-1**	ARMC6	-0.26	armadillo repeat containing 6
**Q5T2E6**	ARMH3	-0.172	armadillo like helical domain containing 3
**Q9H993**	ARMT1	-0.207	acidic residue methyltransferase 1
**Q92747**	ARPC1A	0.133	actin related protein 2/3 complex subunit 1A
**O15145**	ARPC3	-0.172	actin related protein 2/3 complex subunit 3
**P15848**	ARSB	-0.237	arylsulfatase B
**Q13510-2**	ASAH1	-0.174	N-acylsphingosine amidohydrolase 1
**P07306**	ASGR1	-0.359	asialoglycoprotein receptor 1
**P07307**	ASGR2	-0.332	asialoglycoprotein receptor 2
**P04424**	ASL	-0.207	argininosuccinate lyase
**P08243**	ASNS	0.181	asparagine synthetase (glutamine-hydrolyzing)
**Q12797**	ASPH	0.289	aspartate beta-hydroxylase
**Q7L266**	ASRGL1	-0.266	asparaginase and isoaspartyl peptidase 1
**P00966**	ASS1	-0.398	argininosuccinate synthase 1
**Q8NBU5**	ATAD1	0.271	ATPase family AAA domain containing 1
**P18847**	ATF3	0.773	activating transcription factor 3
**P31939**	ATIC	-0.177	5-aminoimidazole-4-carboxamide ribonucleotide formyltransferase/IMP cyclohydrolase
**P05023**	ATP1A1	0.1	ATPase Na+/K+ transporting subunit alpha 1
**P05026**	ATP1B1	0.291	ATPase Na+/K+ transporting subunit beta 1
**P54709**	ATP1B3	0.189	ATPase Na+/K+ transporting subunit beta 3
**P25705-1**	ATP5F1A	0.143	ATP synthase F1 subunit alpha
**P48047**	ATP5PO	0.202	ATP synthase peripheral stalk subunit OSCP
**P61421**	ATP6V0D1	0.187	ATPase H+ transporting V0 subunit d1
**Q9UBB4**	ATXN10	-0.177	ataxin 10
**P54252**	ATXN3	0.578	ataxin 3
**Q9Y679-1**	AUP1	0.139	AUP1 lipid droplet regulating VLDL assembly factor
**O14965**	AURKA	0.207	aurora kinase A
**Q96GD4-5**	AURKB	0.283	aurora kinase B
**O43505**	B4GAT1	-0.466	beta-1,4-glucuronyltransferase 1
**O95817**	BAG3	0.653	BCL2 associated athanogene 3
**O95429**	BAG4	-0.246	BCL2 associated athanogene 4
**Q9UHR4**	BAIAP2L1	0.278	BAR/IMD domain containing adaptor protein 2 like 1
**Q07812-2**	BAX	-0.208	BCL2 associated X, apoptosis regulator
**Q9NRL2**	BAZ1A	0.289	bromodomain adjacent to zinc finger domain 1A
**Q9UIF9**	BAZ2A	0.244	bromodomain adjacent to zinc finger domain 2A
**P50895**	BCAM	-0.392	basal cell adhesion molecule (Lutheran blood group)
**P56945-6**	BCAR1	0.328	BCAR1 scaffold protein, Cas family member
**Q9BUT1**	BDH2	-0.322	3-hydroxybutyrate dehydrogenase 2
**Q9NYM9**	BET1L	-0.254	Bet1 golgi vesicular membrane trafficking protein like
**Q13867**	BLMH	-0.178	bleomycin hydrolase
**P53004**	BLVRA	-0.164	biliverdin reductase A

**O95861-2**	BPNT1	-0.241	3'(2'), 5'-bisphosphate nucleotidase 1
**Q7Z569**	BRAP	0.125	BRCA1 associated protein
**Q15059**	BRD3	-0.243	bromodomain containing 3
**Q8TDN6**	BRIX1	0.26	biogenesis of ribosomes BRX1
**Q5VW32**	BROX	-0.191	BRO1 domain and CAAX motif containing
**P35613**	BSG	-0.167	basigin (Ok blood group)
**Q96K17**	BTF3L4	-0.247	basic transcription factor 3 like 4
**Q7KYR7**	BTN2A1	0.28	butyrophilin subfamily 2 member A1
**O43683**	BUB1	0.182	BUB1 mitotic checkpoint serine/threonine kinase
**O60566-3**	BUB1B	0.123	BUB1 mitotic checkpoint serine/threonine kinase B
**P41223-2**	BUD31	0.237	BUD31 homolog
**Q13895**	BYSL	0.187	bystin like
**Q9Y6E2**	BZW2	-0.226	basic leucine zipper and W2 domains 2
**Q9H0W9**	C11orf54	-0.249	chromosome 11 open reading frame 54
**O00193**	C11orf58	-0.322	chromosome 11 open reading frame 58
**Q9H3H3-3**	C11orf68	-0.136	chromosome 11 open reading frame 68
**A6NCS6**	C2orf72	-0.235	chromosome 2 open reading frame 72
**P01024**	C3	-0.218	complement C3
**Q9BRJ6**	C7orf50	-0.24	chromosome 7 open reading frame 50
**Q8IXQ3**	C9orf40	-0.302	chromosome 9 open reading frame 40
**Q5T6V5**	C9orf64	-0.228	chromosome 9 open reading frame 64
**Q9Y376**	CAB39	-0.154	calcium binding protein 39
**Q13557-10**	CAMK2D	-0.158	calcium/calmodulin dependent protein kinase II delta
**Q86VP6-1**	CAND1	-0.179	cullin associated and neddylation dissociated 1
**P07384**	CAPN1	-0.138	calpain 1
**P47755**	CAPZA2	-0.196	capping actin protein of muscle Z-line subunit alpha 2
**Q9Y2V2**	CARHSP1	-0.129	calcium regulated heat stable protein 1
**P35520-2**	CBS/CBSL	-0.345	cystathionine beta-synthase
**Q5JTY5**	CBWD3/CBWD6	-0.133	COBW domain containing 3
**Q13185**	CBX3	-0.144	chromobox 3
**P45973**	CBX5	-0.197	chromobox 5
**Q96BQ5**	CCDC127	0.372	coiled-coil domain containing 127
**O60826**	CCDC22	-0.163	coiled-coil domain containing 22
**Q96A33**	CCDC47	0.193	coiled-coil domain containing 47
**Q16204**	CCDC6	-0.21	coiled-coil domain containing 6
**Q9H6F5**	CCDC86	0.122	coiled-coil domain containing 86
**O60563**	CCNT1	0.377	cyclin T1
**O14618**	CCS	-0.234	copper chaperone for superoxide dismutase
**Q12834**	CDC20	0.23	cell division cycle 20
**Q9H3Q1**	CDC42EP4	-0.342	CDC42 effector protein 4
**Q6P1J9**	CDC73	0.138	cell division cycle 73
**P38936**	CDKN1A	0.673	cyclin dependent kinase inhibitor 1A
**Q9NXV6**	CDKN2AIP	0.226	CDKN2A interacting protein
**P49715-4**	CEBPA	-0.376	CCAAT enhancer binding protein alpha
**Q03701**	CEBPZ	0.186	CCAAT enhancer binding protein zeta
**Q9UHN6**	CEMIP2	0.238	cell migration inducing hyaluronidase 2
**Q9UPN4**	CEP131	-0.131	centrosomal protein 131

**Q9HA82**	CERS4	-0.176	ceramide synthase 4
**O00748**	CES2	-0.111	carboxylesterase 2
**P41208**	CETN2	-0.311	centrin 2
**Q9UEE9-1**	CFDP1	-0.377	craniofacial development protein 1
**P05156**	CFI	-0.388	complement factor I
**Q9Y281-1**	CFL2	-0.306	cofilin 2
**Q9P2M7-1**	CGN	-0.622	cingulin
**Q13111-1**	CHAF1A	-0.155	chromatin assembly factor 1 subunit A
**Q9NX63**	CHCHD3	0.161	coiled-coil-helix-coiled-coil-helix domain containing 3
**O14647**	CHD2	0.297	chromodomain helicase DNA binding protein 2
**Q8IWX8**	CHERP	0.132	calcium homeostasis endoplasmic reticulum protein
**Q9UQN3-1**	CHMP2B	0.128	charged multivesicular body protein 2B
**Q9H444**	CHMP4B	0.138	charged multivesicular body protein 4B
**Q9UHD1**	CHORDC1	0.324	cysteine and histidine rich domain containing 1
**Q99653**	CHP1	0.475	calcineurin like EF-hand protein 1
**P12277**	CKB	-0.228	creatine kinase B
**P56747**	CLDN6	0.275	claudin 6
**Q16740**	CLPP	0.173	caseinolytic mitochondrial matrix peptidase proteolytic subunit
**O76031**	CLPX	0.166	caseinolytic mitochondrial matrix peptidase chaperone subunit
**P09497-2**	CLTB	0.338	clathrin light chain B
**P10909-2**	CLU	0.489	clusterin
**I3L2B0**	CLUH	-0.265	clustered mitochondria homolog
**O75153**	CLUH	-0.214	clustered mitochondria homolog
**P62633**	CNBP	-1.217	CCHC-type zinc finger nucleic acid binding protein
**P62633-6**	CNBP	-0.857	CCHC-type zinc finger nucleic acid binding protein
**Q96KP4**	CNDP2	-0.285	carnosine dipeptidase 2
**A5YKK6**	CNOT1	-0.122	CCR4-NOT transcription complex subunit 1
**Q9UIV1-1**	CNOT7	-0.194	CCR4-NOT transcription complex subunit 7
**Q92600**	CNOT9	-0.198	CCR4-NOT transcription complex subunit 9
**P09543-1**	CNP	-0.133	2',3'-cyclic nucleotide 3' phosphodiesterase
**Q96JB2-1**	COG3	-0.159	component of oligomeric golgi complex 3
**Q9H9E3**	COG4	-0.122	component of oligomeric golgi complex 4
**Q9NX08**	COMMD8	-0.244	COMM domain containing 8
**Q9P000-1**	COMMD9	-0.36	COMM domain containing 9
**P53618**	COPB1	-0.108	coatomer protein complex subunit beta 1
**P35606**	COPB2	-0.117	coatomer protein complex subunit beta 2
**Q9Y678**	COPG1	-0.121	coatomer protein complex subunit gamma 1
**P31146**	CORO1A	-0.261	coronin 1A
**Q14019**	COTL1	-0.28	coactosin like F-actin binding protein 1
**O75976**	CPD	-0.231	carboxypeptidase D
**Q17RY0-1**	CPEB4	0.254	cytoplasmic polyadenylation element binding protein 4
**O75131**	CPNE3	-0.118	copine 3
**Q9BRF8-1**	CPPED1	-0.283	calcineurin like phosphoesterase domain containing 1
**P46108-1**	CRK	-0.202	CRK proto-oncogene, adaptor protein
**P46109**	CRKL	-0.222	CRK like proto-oncogene, adaptor protein
**O75718**	CRTAP	-0.153	cartilage associated protein
**P02511**	CRYAB	2.014	crystallin alpha B

**Q9Y2S2-1**	CRYL1	-0.371	crystallin lambda 1
**Q08257**	CRYZ	0.127	crystallin zeta
**P55060**	CSE1L	-0.226	chromosome segregation 1 like
**P68400**	CSNK2A1	0.164	casein kinase 2 alpha 1
**P19784**	CSNK2A2	0.145	casein kinase 2 alpha 2
**P01034**	CST3	-0.426	cystatin C
**P04080**	CSTB	-0.402	cystatin B
**P32929**	CTH	0.318	cystathionine gamma-lyase
**Q9NSA3**	CTNNBIP1	-0.46	catenin beta interacting protein 1
**Q9NRF8**	CTPS2	-0.224	CTP synthase 2
**Q6PD62**	CTR9	0.229	CTR9 homolog, Paf1/RNA polymerase II complex component
**P53634**	CTSC	-0.172	cathepsin C
**P09668**	CTSH	-0.15	cathepsin H
**P07711**	CTSL	0.38	cathepsin L
**Q13616**	CUL1	-0.137	cullin 1
**Q13619-1**	CUL4A	-0.114	cullin 4A
**P00167-1**	CYB5A	-0.391	cytochrome b5 type A
**P99999**	CYCS	-0.346	cytochrome c, somatic
**Q7L576-1**	CYFIP1	-0.123	cytoplasmic FMR1 interacting protein 1
**Q07973-1**	CYP24A1	0.886	cytochrome P450 family 24 subfamily A member 1
**Q96SQ9-2**	CYP2S1	0.314	cytochrome P450 family 2 subfamily S member 1
**Q8TAV3**	CYP2W1	-0.274	cytochrome P450 family 2 subfamily W member 1
**Q9HBI6**	CYP4F11	0.355	cytochrome P450 family 4 subfamily F member 11
**Q16850-1**	CYP51A1	-0.568	cytochrome P450 family 51 subfamily A member 1
**Q5VWQ8**	DAB2IP	-0.191	DAB2 interacting protein
**Q14118**	DAG1	-0.169	dystroglycan 1
**P51397**	DAP	-0.419	death associated protein
**O43293-1**	DAPK3	0.198	death associated protein kinase 3
**P07108-5**	DBI	-0.743	diazepam binding inhibitor, acyl-CoA binding protein
**P11182**	DBT	0.127	dihydrolipoamide branched chain transacylase E2
**Q9NV06**	DCAF13	0.179	DDB1 and CUL4 associated factor 13
**P61962**	DCAF7	0.115	DDB1 and CUL4 associated factor 7
**Q96C86**	DCPS	-0.231	decapping enzyme, scavenger
**Q9H773**	DCTPP1	-0.244	dCTP pyrophosphatase 1
**O94760**	DDAH1	-0.336	dimethylarginine dimethylaminohydrolase 1
**Q16531**	DDB1	-0.183	damage specific DNA binding protein 1
**Q5TDH0-3**	DDI2	-0.307	DNA damage inducible 1 homolog 2
**Q9NVP1**	DDX18	0.162	DEAD-box helicase 18
**Q9NUU7**	DDX19A	-0.146	DEAD-box helicase 19A
**Q9NR30**	DDX21	0.353	DExD-box helicase 21
**Q9GZR7**	DDX24	0.3	DEAD-box helicase 24
**Q9H0S4**	DDX47	0.183	DEAD-box helicase 47
**O43583**	DENR	-0.303	density regulated re-initiation and release factor
**Q9Y315**	DERA	-0.171	deoxyribose-phosphate aldolase
**Q8WYQ5-1**	DGCR8	0.18	DGCR8 microprocessor complex subunit
**Q9UPY3**	DICER1	-0.154	dicer 1, ribonuclease III
**O60832**	DKC1	0.161	dyskerin pseudouridine synthase 1

**P09622**	DLD	0.206	dihydrolipoamide dehydrogenase
**P11532-6**	DMD	-0.175	dystrophin
**Q08495-1**	DMTN	-0.206	dematin actin binding protein
**Q86Y56**	DNAAF5	-0.241	dynein axonemal assembly factor 5
**P31689**	DNAJA1	0.175	DnaJ heat shock protein family (Hsp40) member A1
**P25685**	DNAJB1	0.744	DnaJ heat shock protein family (Hsp40) member B1
**Q9NXW2-2**	DNAJB12	0.278	DnaJ heat shock protein family (Hsp40) member B12
**P25686-3**	DNAJB2	0.316	DnaJ heat shock protein family (Hsp40) member B2
**Q9UDY4**	DNAJB4	0.77	DnaJ heat shock protein family (Hsp40) member B4
**Q9Y5T4**	DNAJC15	0.309	DnaJ heat shock protein family (Hsp40) member C15
**Q13217**	DNAJC3	0.125	DnaJ heat shock protein family (Hsp40) member C3
**Q9H3Z4**	DNAJC5	-0.427	DnaJ heat shock protein family (Hsp40) member C5
**Q9ULA0**	DNPEP	-0.217	aspartyl aminopeptidase
**O43598-1**	DNPH1	-0.446	2'-deoxynucleoside 5'-phosphate N-hydrolase 1
**Q5QJE6**	DNTTIP2	0.196	deoxynucleotidyltransferase terminal interacting protein 2
**Q9H2P9-5**	DPH5	-0.397	diphthamide biosynthesis 5
**Q9NY33**	DPP3	-0.132	dipeptidyl peptidase 3
**Q16555**	DPYSL2	-0.258	dihydropyrimidinase like 2
**Q8TE96**	DQX1	-0.199	DEAQ-box RNA dependent ATPase 1
**Q03001-7**	DST	0.231	dystonin
**P60981-1**	DSTN	0.149	destrin, actin depolymerizing factor
**P23919**	DTYMK	-0.157	deoxythymidylate kinase
**Q6P1R4**	DUS1L	-0.205	dihydrouridine synthase 1 like
**Q99956**	DUSP9	-0.277	dual specificity phosphatase 9
**P33316-3**	DUT	-0.219	deoxyuridine triphosphatase
**P63167**	DYNLL1	0.384	dynein light chain LC8-type 1
**O95905-3**	ECD	0.313	ecdysoneless cell cycle regulator
**P42892**	ECE1	-0.185	endothelin converting enzyme 1
**O75521**	ECI2	0.096	enoyl-CoA delta isomerase 2
**Q9H8V3-3**	ECT2	0.196	epithelial cell transforming 2
**Q9H223**	EHD4	0.113	EH domain containing 4
**P41567**	EIF1	0.156	eukaryotic translation initiation factor 1
**Q9P2K8-1**	EIF2AK4	-0.172	eukaryotic translation initiation factor 2 alpha kinase 4
**Q14232**	EIF2B1	-0.195	eukaryotic translation initiation factor 2B subunit alpha
**Q13144**	EIF2B5	-0.133	eukaryotic translation initiation factor 2B subunit epsilon
**O75822**	EIF3J	-0.181	eukaryotic translation initiation factor 3 subunit J
**Q9UBQ5**	EIF3K	-0.249	eukaryotic translation initiation factor 3 subunit K
**P55199**	ELL	0.383	elongation factor for RNA polymerase II
**O00472**	ELL2	0.2	elongation factor for RNA polymerase II 2
**Q14241**	ELOA	0.338	elongin A
**O95163**	ELP1	-0.265	elongator complex protein 1
**Q9H9T3**	ELP3	-0.263	elongator acetyltransferase complex subunit 3
**Q15006**	EMC2	0.204	ER membrane protein complex subunit 2
**Q14249**	ENDOG	-0.168	endonuclease G
**P13929-1**	ENO3	-0.415	enolase 3
**Q9UHY7**	ENOPH1	-0.27	enolase-phosphatase 1
**P22413**	ENPP1	-0.179	ectonucleotide pyrophosphatase/phosphodiesterase 1

**O43768-4**	ENSA	-0.311	endosulfine alpha
**P16422**	EPCAM	-0.196	epithelial cell adhesion molecule
**P29317**	EPHA2	0.553	EPH receptor A2
**P07099**	EPHX1	0.161	epoxide hydrolase 1
**P34913**	EPHX2	-0.242	epoxide hydrolase 2
**P58107**	EPPK1	-0.16	epiplakin 1
**Q12929**	EPS8	0.142	epidermal growth factor receptor pathway substrate 8
**Q9H6S3**	EPS8L2	-0.148	EPS8 like 2
**Q9NZ08-2**	ERAP1	-0.126	endoplasmic reticulum aminopeptidase 1
**P04626**	ERBB2	-0.229	erb-b2 receptor tyrosine kinase 2
**P21860**	ERBB3	-0.279	erb-b2 receptor tyrosine kinase 3
**Q96RT1-8**	ERBIN	0.096	erbb2 interacting protein
**P19447**	ERCC3	0.239	ERCC excision repair 3, TFIIH core complex helicase subunit
**Q9Y282-3**	ERGIC3	0.157	ERGIC and golgi 3
**Q96AP7**	ESAM	0.89	endothelial cell adhesion molecule
**B1AK53-1**	ESPN	-0.556	espin
**P62495**	ETF1	-0.224	eukaryotic translation termination factor 1
**Q01780**	EXOSC10	0.178	exosome component 10
**Q9NQT5**	EXOSC3	0.109	exosome component 3
**P07148**	FABP1	-0.658	fatty acid binding protein 1
**O60427**	FADS1	-0.198	fatty acid desaturase 1
**O95864**	FADS2	-0.5	fatty acid desaturase 2
**Q96CS3**	FAF2	0.246	Fas associated factor family member 2
**P16930**	FAH	-0.315	fumarylacetoacetate hydrolase
**Q8IWE2**	FAM114A1	-0.293	family with sequence similarity 114 member A1
**Q9NZB2-6**	FAM120A	-0.136	family with sequence similarity 120A
**Q96C01**	FAM136A	-0.238	family with sequence similarity 136 member A
**Q8N128-2**	FAM177A1	0.185	family with sequence similarity 177 member A1
**Q9GZU8**	FAM192A	-0.181	family with sequence similarity 192 member A
**Q52LJ0-2**	FAM98B	0.108	family with sequence similarity 98 member B
**Q9NVI1**	FANCI	-0.226	FA complementation group I
**Q9Y285**	FARSA	-0.131	phenylalanyl-tRNA synthetase subunit alpha
**Q9NSD9**	FARSB	-0.201	phenylalanyl-tRNA synthetase subunit beta
**P25445**	FAS	0.158	Fas cell surface death receptor
**P49327**	FASN	-0.28	fatty acid synthase
**Q7L8L6**	FASTKD5	0.463	FAST kinase domains 5
**Q14517**	FAT1	0.32	FAT atypical cadherin 1
**P22087**	FBL	0.15	fibrillarin
**Q8TB52**	FBXO30	0.215	F-box protein 30
**Q9Y3I1**	FBXO7	-0.192	F-box protein 7
**P55899**	FCGRT	-0.232	Fc fragment of IgG receptor and transporter
**P37268**	FDFT1	-0.332	farnesyl-diphosphate farnesyltransferase 1
**P14324**	FDPS	-0.244	farnesyl diphosphate synthase
**P02671-2**	FGA	-0.626	fibrinogen alpha chain
**P02675**	FGB	-0.281	fibrinogen beta chain
**P22455-1**	FGFR4	-0.407	fibroblast growth factor receptor 4
**P02679**	FGG	-0.54	fibrinogen gamma chain

**Q96AY3**	FKBP10	-0.137	FKBP prolyl isomerase 10
**Q9NWM8**	FKBP14	-0.214	FKBP prolyl isomerase 14
**P62942**	FKBP1A	-0.298	FKBP prolyl isomerase 1A
**Q00688**	FKBP3	-0.184	FKBP prolyl isomerase 3
**Q02790**	FKBP4	0.235	FKBP prolyl isomerase 4
**Q9Y5Y0**	FLVCR1	-0.294	feline leukemia virus subgroup C cellular receptor 1
**Q96CP2**	FLYWCH2	-0.358	FLYWCH family member 2
**P49326-1**	FMO5	-0.176	flavin containing dimethylaniline monoxygenase 5
**Q9H479**	FN3K	-0.254	fructosamine 3 kinase
**Q9Y2H6**	FNDC3A	0.179	fibronectin type III domain containing 3A
**Q5VW36**	FOCAD	-0.171	focadhesin
**P15407**	FOSL1	1.156	FOS like 1, AP-1 transcription factor subunit
**Q9H334-8**	FOXP1	0.212	forkhead box P1
**Q86XX4-2**	FRAS1	-0.549	Fraser extracellular matrix complex subunit 1
**Q16658**	FSCN1	-0.121	fascin actin-bundling protein 1
**O95954-1**	FTCD	-0.297	formimidoyltransferase cyclodeaminase
**P02792**	FTL	0.262	ferritin light chain
**Q9C0B1-1**	FTO	-0.252	FTO alpha-ketoglutarate dependent dioxygenase
**P51114**	FXR1	0.184	FMR1 autosomal homolog 1
**P51116**	FXR2	0.123	FMR1 autosomal homolog 2
**P11413-2**	G6PD	-0.155	glucose-6-phosphate dehydrogenase
**Q14376**	GALE	-0.345	UDP-galactose-4-epimerase
**P51570-2**	GALK1	-0.225	galactokinase 1
**Q96C23**	GALM	-0.359	galactose mutarotase
**P07902**	GALT	-0.213	galactose-1-phosphate uridylyltransferase
**Q14353**	GAMT	-0.36	guanidinoacetate N-methyltransferase
**P04406**	GAPDH	-0.169	glyceraldehyde-3-phosphate dehydrogenase
**Q9NY12**	GAR1	0.26	GAR1 ribonucleoprotein
**P22102**	GART	-0.121	phosphoribosylglycinamide formyltransferase, phosphoribosylglycinamide synthetase, phosphoribosyl
**O43903**	GAS2	-0.159	growth arrest specific 2
**Q86YP4-3**	GATAD2A	0.142	GATA zinc finger domain containing 2A
**Q8WXI9**	GATAD2B	0.18	GATA zinc finger domain containing 2B
**P30047**	GCHFR	-0.268	GTP cyclohydrolase I feedback regulator
**P48506**	GCLC	0.381	glutamate-cysteine ligase catalytic subunit
**P48507**	GCLM	0.909	glutamate-cysteine ligase modifier subunit
**Q92616**	GCN1	-0.163	GCN1 activator of EIF2AK4
**Q8TB36**	GDAP1	0.368	ganglioside induced differentiation associated protein 1
**Q9NZC3**	GDE1	0.299	glycerophosphodiester phosphodiesterase 1
**Q99988**	GDF15	0.78	growth differentiation factor 15
**P50395-1**	GDI2	-0.233	GDP dissociation inhibitor 2
**Q7L5D6**	GET4	-0.118	guided entry of tail-anchored proteins factor 4
**Q06210**	GFPT1	-0.11	glutamine--fructose-6-phosphate transaminase 1
**Q9H3K2**	GHITM	0.335	growth hormone inducible transmembrane protein
**Q9BRX5**	GINS3	-0.274	GINS complex subunit 3
**Q9BRT9**	GINS4	-0.187	GINS complex subunit 4
**Q8TF65**	GIPC2	-0.137	GIPC PDZ domain containing family member 2
**P08034**	GJB1	-0.663	gap junction protein beta 1

**Q92896-2**	GLG1	-0.332	golgi glycoprotein 1
**Q04760-1**	GLO1	-0.184	glyoxalase I
**Q9HC38**	GLOD4	-0.239	glyoxalase domain containing 4
**O94925**	GLS	0.315	glutaminase
**O94925-3**	GLS	0.323	glutaminase
**Q68CQ7-1**	GLT8D1	-0.213	glycosyltransferase 8 domain containing 1
**P15104**	GLUL	-0.32	glutamate-ammonia ligase
**O60547**	GMDS	-0.255	GDP-mannose 4,6-dehydratase
**Q9Y5P6-2**	GMPPB	-0.293	GDP-mannose pyrophosphorylase B
**P49915**	GMPS	-0.182	guanine monophosphate synthase
**Q14344**	GNA13	0.375	G protein subunit alpha 13
**P08754**	GNAI3	0.145	G protein subunit alpha i3
**P62873**	GNB1	0.166	G protein subunit beta 1
**Q13823**	GNL2	0.252	G protein nucleolar 2
**Q96EK6**	GNPNAT1	-0.192	glucosamine-phosphate N-acetyltransferase 1
**Q7Z5G4**	GOLGA7	0.215	golgin A7
**Q9H4A6**	GOLPH3	-0.182	golgi phosphoprotein 3
**Q9H4A5**	GOLPH3L	-0.178	golgi phosphoprotein 3 like
**Q9Y3E0**	GOLT1B	0.339	golgi transport 1B
**Q9H8Y8**	GORASP2	-0.168	golgi reassembly stacking protein 2
**P00505**	GOT2	0.154	glutamic-oxaloacetic transaminase 2
**Q53EU6**	GPAT3	0.448	glycerol-3-phosphate acyltransferase 3
**P51654-1**	GPC3	-0.297	glypican 3
**Q9Y625**	GPC6	-0.304	glypican 6
**P21695**	GPD1	-0.376	glycerol-3-phosphate dehydrogenase 1
**Q9NQX3-2**	GPHN	-0.242	gephyrin
**P06744**	GPI	-0.188	glucose-6-phosphate isomerase
**Q9NQ84-2**	GPRC5C	0.451	G protein-coupled receptor class C group 5 member C
**P18283**	GPX2	-0.395	glutathione peroxidase 2
**P36969**	GPX4	-0.589	glutathione peroxidase 4
**Q9UBQ7**	GRHPR	-0.181	glyoxylate and hydroxypyruvate reductase
**P25098**	GRK2	-0.204	G protein-coupled receptor kinase 2
**P48637**	GSS	-0.186	glutathione synthetase
**P08263**	GSTA1	-0.502	glutathione S-transferase alpha 1
**P28161**	GSTM2	-0.202	glutathione S-transferase mu 2
**P21266**	GSTM3	-0.165	glutathione S-transferase mu 3
**P78417**	GSTO1	-0.297	glutathione S-transferase omega 1
**P30711**	GSTT1	-0.192	glutathione S-transferase theta 1
**P78347**	GTF2I	-0.141	general transcription factor IIi
**Q8N442**	GUF1	0.196	GUF1 homolog, GTPase
**P0C0S5**	H2AZ1	0.367	H2A.Z variant histone 1
**O95479**	H6PD	-0.135	hexose-6-phosphate dehydrogenase/glucose 1-dehydrogenase
**P46952**	HAAO	-0.222	3-hydroxyanthranilate 3,4-dioxygenase
**O14929**	HAT1	-0.113	histone acetyltransferase 1
**P51858**	HDGF	-0.21	heparin binding growth factor
**Q9H0R4**	HDHD2	-0.221	haloacid dehalogenase like hydrolase domain containing 2
**Q00341**	HDLBP	-0.254	high density lipoprotein binding protein

**Q7Z4Q2**	HEATR3	-0.307	HEAT repeat containing 3
**Q86XA9**	HEATR5A	-0.249	HEAT repeat containing 5A
**Q9P2D3**	HEATR5B	-0.128	HEAT repeat containing 5B
**Q9NRV9**	HEBP1	-0.157	heme binding protein 1
**Q9Y5Z4**	HEBP2	-0.288	heme binding protein 2
**Q93099**	HGD	-0.215	homogentisate 1,2-dioxygenase
**Q9BTY7**	HGH1	-0.108	HGH1 homolog
**O14964**	HGS	0.203	hepatocyte growth factor-regulated tyrosine kinase substrate
**P49773**	HINT1	-0.109	histidine triad nucleotide binding protein 1
**Q9BX68**	HINT2	0.19	histidine triad nucleotide binding protein 2
**Q9BW71-1**	HIRIP3	-0.18	HIRA interacting protein 3
**P08397**	HMBS	-0.23	hydroxymethylbilane synthase
**Q01581**	HMGCS1	-0.385	3-hydroxy-3-methylglutaryl-CoA synthase 1
**P05204**	HMGN2	0.517	high mobility group nucleosomal binding domain 2
**P09601**	HMOX1	1.109	heme oxygenase 1
**P30519**	HMOX2	0.128	heme oxygenase 2
**P20823-7**	HNF1A	-0.182	HNF1 homeobox A
**P41235-4**	HNF4A	-0.27	hepatocyte nuclear factor 4 alpha
**P50135**	HNMT	-0.44	histamine N-methyltransferase
**P14866**	HNRNPL	0.1	heterogeneous nuclear ribonucleoprotein L
**Q00839**	HNRNPU	0.164	heterogeneous nuclear ribonucleoprotein U
**Q1KMD3**	HNRNPUL2	-0.102	heterogeneous nuclear ribonucleoprotein U like 2
**Q9NSC5**	HOMER3	-0.246	homer scaffold protein 3
**P00738**	HP	-0.677	haptoglobin
**P32754-1**	HPD	-0.147	4-hydroxyphenylpyruvate dioxygenase
**P07900**	HSP90AA1	0.213	heat shock protein 90 alpha family class A member 1
**P08238**	HSP90AB1	0.141	heat shock protein 90 alpha family class B member 1
**P48723**	HSPA13	0.195	heat shock protein family A (Hsp70) member 13
**P0DMV9**	HSPA1A/HSPA1B	1.375	heat shock protein family A (Hsp70) member 1A
**P17066**	HSPA6	1.615	heat shock protein family A (Hsp70) member 6
**P38646**	HSPA9	0.149	heat shock protein family A (Hsp70) member 9
**P04792**	HSPB1	0.759	heat shock protein family B (small) member 1
**P10809**	HSPD1	0.202	heat shock protein family D (Hsp60) member 1
**Q92598**	HSPH1	0.662	heat shock protein family H (Hsp110) member 1
**P42858**	HTT	-0.184	huntingtin
**P41252**	IARS1	-0.15	isoleucyl-tRNA synthetase 1
**Q5T440**	IBA57	0.131	iron-sulfur cluster assembly factor IBA57
**O75874**	IDH1	-0.383	isocitrate dehydrogenase (NADP(+)) 1
**P50213**	IDH3A	0.116	isocitrate dehydrogenase (NAD(+)) 3 catalytic subunit alpha
**O43837**	IDH3B	0.169	isocitrate dehydrogenase (NAD(+)) 3 non-catalytic subunit beta
**Q13907**	IDI1	-0.237	isopentenyl-diphosphate delta isomerase 1
**P08833**	IGFBP1	0.492	insulin like growth factor binding protein 1
**Q8N6C5-4**	IGSF1	-0.296	immunoglobulin superfamily member 1
**Q70UQ0-4**	IKBIP	0.158	IKBKB interacting protein
**Q14116**	IL18	0.44	interleukin 18
**Q96G21**	IMP4	0.16	IMP U3 small nucleolar ribonucleoprotein 4
**Q27J81**	INF2	-0.117	inverted formin, FH2 and WH2 domain containing

**Q9UI26-2**	IPO11	-0.127	importin 11
**Q8TEX9-2**	IPO4	-0.351	importin 4
**O00410-3**	IPO5	-0.317	importin 5
**O95373**	IPO7	-0.15	importin 7
**Q96P70**	IPO9	-0.253	importin 9
**Q13576-1**	IQGAP2	-0.168	IQ motif containing GTPase activating protein 2
**Q86U28**	ISCA2	-0.212	iron-sulfur cluster assembly 2
**P05161**	ISG15	-0.144	ISG15 ubiquitin like modifier
**Q9H9L3**	ISG20L2	0.142	interferon stimulated exonuclease gene 20 like 2
**Q96CN7**	ISOC1	-0.194	isochorismatase domain containing 1
**Q9NPH2**	ISYNA1	-0.304	inositol-3-phosphate synthase 1
**P56199**	ITGA1	-0.199	integrin subunit alpha 1
**P17301**	ITGA2	0.473	integrin subunit alpha 2
**P06756**	ITGAV	0.153	integrin subunit alpha V
**P05556**	ITGB1	0.25	integrin subunit beta 1
**P19823**	ITIH2	-0.115	inter-alpha-trypsin inhibitor heavy chain 2
**Q9BY32**	ITPA	-0.195	inosine triphosphatase
**P78504**	JAG1	0.61	jagged canonical Notch ligand 1
**P14923**	JUP	0.106	junction plakoglobin
**Q9Y2K7**	KDM2A	0.24	lysine demethylase 2A
**Q9Y4C1**	KDM3A	0.302	lysine demethylase 3A
**Q7LBC6-1**	KDM3B	-0.147	lysine demethylase 3B
**Q14145**	KEAP1	0.134	kelch like ECH associated protein 1
**P50053-2**	KHK	-0.342	ketohexokinase
**Q9NQT8**	KIF13B	-0.161	kinesin family member 13B
**Q9NS87**	KIF15	-0.149	kinesin family member 15
**Q02241-1**	KIF23	0.191	kinesin family member 23
**Q99661**	KIF2C	0.153	kinesin family member 2C
**P33176**	KIF5B	-0.108	kinesin family member 5B
**Q9NSK0-3**	KLC4	-0.221	kinesin light chain 4
**Q14974**	KPNB1	-0.226	karyopherin subunit beta 1
**Q13601**	KRR1	0.315	KRR1 small subunit processome component homolog
**P08727**	KRT19	0.325	keratin 19
**Q16719**	KYNU	0.265	kynureninase
**P83111-1**	LACTB	0.198	lactamase beta
**O15230**	LAMA5	-0.384	laminin subunit alpha 5
**P55268**	LAMB2	-0.274	laminin subunit beta 2
**P13473-3**	LAMP2	-0.332	lysosomal associated membrane protein 2
**O43813**	LANCL1	-0.293	LanC like 1
**Q71RC2-4**	LARP4	0.138	La ribonucleoprotein domain family member 4
**P00338-3**	LDHA	-0.345	lactate dehydrogenase A
**P01130**	LDLR	0.296	low density lipoprotein receptor
**Q96PV6-2**	LENG8	0.295	leukocyte receptor cluster member 8
**P09382**	LGALS1	0.234	galectin 1
**P17931**	LGALS3	-0.207	galectin 3
**O00214-2**	LGALS8	0.291	galectin 8
**Q3ZCW2**	LGALSL	-0.155	galectin like

**P18858**	LIG1	-0.287	DNA ligase 1
**P20700**	LMNB1	-0.133	lamin B1
**Q7Z4F1**	LRP10	0.493	LDL receptor related protein 10
**Q8N1G4**	LRRC47	-0.206	leucine rich repeat containing 47
**Q96AG4**	LRRC59	0.138	leucine rich repeat containing 59
**Q8IWT6**	LRRC8A	0.17	leucine rich repeat containing 8 VRAC subunit A
**Q32MZ4-3**	LRRFIP1	0.132	LRR binding FLII interacting protein 1
**Q9UFC0**	LRWD1	0.101	leucine rich repeats and WD repeat domain containing 1
**Q9H089**	LSG1	0.27	large 60S subunit nuclear export GTPase 1
**P48449**	LSS	-0.14	lanosterol synthase
**P09960-1**	LTA4H	-0.27	leukotriene A4 hydrolase
**O94822-3**	LTN1	-0.15	listerin E3 ubiquitin protein ligase 1
**Q96GA3**	LTV1	0.226	LTV1 ribosome biogenesis factor
**Q86V48**	LUZP1	0.288	leucine zipper protein 1
**Q8WZA0-2**	LZIC	-0.241	leucine zipper and CTNNBIP1 domain containing
**Q9NQ48**	LZTFL1	-0.296	leucine zipper transcription factor like 1
**Q8WXG6-4**	MADD	0.201	MAP kinase activating death domain
**Q9ULX9**	MAFF	0.467	MAF bZIP transcription factor F
**O15525**	MAFG	0.715	MAF bZIP transcription factor G
**Q9Y5V3-2**	MAGED1	-0.289	MAGE family member D1
**P33908**	MAN1A1	-0.14	mannosidase alpha class 1A member 1
**P55145**	MANF	-0.172	mesencephalic astrocyte derived neurotrophic factor
**P46821**	MAP1B	0.551	microtubule associated protein 1B
**A6NCE7**	MAP1LC3B2	0.826	microtubule associated protein 1 light chain 3 beta 2
**P27816-1**	MAP4	-0.124	microtubule associated protein 4
**Q3KQU3**	MAP7D1	0.27	MAP7 domain containing 1
**P28482**	MAPK1	-0.272	mitogen-activated protein kinase 1
**P27361-1**	MAPK3	-0.233	mitogen-activated protein kinase 3
**Q5VT66-2**	MARC1	-0.224	mitochondrial amidoxime reducing component 1
**Q00266**	MAT1A	-0.316	methionine adenosyltransferase 1A
**Q8NI22**	MCFD2	-0.479	multiple coagulation factor deficiency 2
**P49736**	MCM2	-0.248	minichromosome maintenance complex component 2
**P25205-2**	MCM3	-0.186	minichromosome maintenance complex component 3
**P33991**	MCM4	-0.201	minichromosome maintenance complex component 4
**P33992**	MCM5	-0.179	minichromosome maintenance complex component 5
**Q14566**	MCM6	-0.211	minichromosome maintenance complex component 6
**P33993-1**	MCM7	-0.12	minichromosome maintenance complex component 7
**Q9BTE3-1**	MCMBP	-0.128	minichromosome maintenance complex binding protein
**Q8NE86**	MCU	0.322	mitochondrial calcium uniporter
**P40925-3**	MDH1	-0.296	malate dehydrogenase 1
**Q00987-11**	MDM2	0.424	MDM2 proto-oncogene
**P51608-2**	MECP2	0.201	methyl-CpG binding protein 2
**Q14696**	MESD	-0.223	mesoderm development LRP chaperone
**Q9UBP6**	METTL1	-0.145	methyltransferase like 1
**Q9H8H3**	METTL7A	-0.216	methyltransferase like 7A
**Q9H1A3**	METTL9	0.312	methyltransferase like 9
**Q8IWA4**	MFN1	0.215	mitofusin 1

**P16455**	MGMT	-0.213	O-6-methylguanine-DNA methyltransferase
**Q8N108-12**	MIER1	-0.122	MIER1 transcriptional regulator
**Q9UNW1**	MINPP1	-0.126	multiple inositol-polyphosphate phosphatase 1
**Q8IVT2**	MISP	0.431	mitotic spindle positioning
**Q15773**	MLF2	0.24	myeloid leukemia factor 2
**P51511**	MMP15	-0.337	matrix metallopeptidase 15
**Q96T76-8**	MMS19	-0.211	MMS19 homolog, cytosolic iron-sulfur assembly component
**Q7Z3U7**	MON2	-0.223	MON2 homolog, regulator of endosome-to-Golgi trafficking
**Q15014**	MORF4L2	-0.204	mortality factor 4 like 2
**Q9HCE1**	MOV10	-0.19	Mov10 RISC complex RNA helicase
**Q9Y5U8**	MPC1	0.194	mitochondrial pyruvate carrier 1
**P34949**	MPI	-0.26	mannose phosphate isomerase
**Q5T2T1**	MPP7	-0.14	membrane palmitoylated protein 7
**P25325-2**	MPST	-0.132	mercaptopyruvate sulfurtransferase
**Q9Y605**	MRFAP1	-0.313	Morf4 family associated protein 1
**Q9P015**	MRPL15	0.19	mitochondrial ribosomal protein L15
**Q9P0M9**	MRPL27	0.264	mitochondrial ribosomal protein L27
**P09001**	MRPL3	0.209	mitochondrial ribosomal protein L3
**Q9NYK5**	MRPL39	0.158	mitochondrial ribosomal protein L39
**Q9BRJ2**	MRPL45	0.211	mitochondrial ribosomal protein L45
**Q9BQC6**	MRPL57	0.182	mitochondrial ribosomal protein L57
**Q9Y399**	MRPS2	0.256	mitochondrial ribosomal protein S2
**Q96EL2**	MRPS24	0.436	mitochondrial ribosomal protein S24
**Q9BYN8**	MRPS26	0.162	mitochondrial ribosomal protein S26
**P82673**	MRPS35	0.265	mitochondrial ribosomal protein S35
**P82909**	MRPS36	0.136	mitochondrial ribosomal protein S36
**P82675**	MRPS5	0.261	mitochondrial ribosomal protein S5
**P82932**	MRPS6	0.368	mitochondrial ribosomal protein S6
**Q9Y2R9**	MRPS7	0.204	mitochondrial ribosomal protein S7
**Q9ULH7-5**	MRTFB	-0.166	myocardin related transcription factor B
**P43246**	MSH2	-0.192	mutS homolog 2
**P52701**	MSH6	-0.132	mutS homolog 6
**P07438**	MT1B	3.014	metallothionein 1B
**O94776**	MTA2	0.215	metastasis associated 1 family member 2
**Q96E29**	MTERF3	0.185	mitochondrial transcription termination factor 3
**Q9H019-1**	MTFR1L	-0.165	mitochondrial fission regulator 1 like
**P11586**	MTHFD1	-0.253	methylenetetrahydrofolate dehydrogenase, cyclohydrolase and formyltetrahydrofolate synthetase 1
**P13995**	MTHFD2	0.186	methylenetetrahydrofolate dehydrogenase (NADP+ dependent) 2, methenyltetrahydrofolate cyclohyd
**Q13496**	MTM1	-0.169	myotubularin 1
**Q9C0I1**	MTMR12	-0.195	myotubularin related protein 12
**Q765P7**	MTSS2	-0.371	MTSS I-BAR domain containing 2
**Q5HYI7**	MTX3	0.188	metaxin 3
**P53602**	MVD	-0.215	mevalonate diphosphate decarboxylase
**Q03426**	MVK	-0.302	mevalonate kinase
**Q14764**	MVP	0.168	major vault protein
**P35579**	MYH9	0.319	myosin heavy chain 9
**Q15746**	MYLK	-0.216	myosin light chain kinase

**Q96H55-1**	MYO19	-0.252	myosin XIX
**O00159-3**	MYO1C	0.122	myosin IC
**Q6NSJ0**	MYORG	-0.298	myogenesis regulating glycosidase (putative)
**Q13564-4**	NAE1	-0.166	NEDD8 activating enzyme E1 subunit 1
**Q9UJ70-2**	NAGK	-0.123	N-acetylglucosamine kinase
**Q9NR45**	NANS	-0.231	N-acetylneuraminate synthase
**O43776**	NARS1	-0.145	asparaginyl-tRNA synthetase 1
**P49321-3**	NASP	-0.28	nuclear autoantigenic sperm protein
**A2RRP1**	NBAS	-0.129	NBAS subunit of NRZ tethering complex
**Q15021**	NCAPD2	-0.122	non-SMC condensin I complex subunit D2
**Q6PIU2-2**	NCEH1	0.261	neutral cholesterol ester hydrolase 1
**Q68D85**	NCR3LG1	-0.265	natural killer cell cytotoxicity receptor 3 ligand 1
**Q9UI09**	NDUFA12	0.103	NADH:ubiquinone oxidoreductase subunit A12
**Q9P0J0**	NDUFA13	0.178	NADH:ubiquinone oxidoreductase subunit A13
**O00483**	NDUFA4	-0.294	NDUFA4 mitochondrial complex associated
**P56556**	NDUFA6	0.162	NADH:ubiquinone oxidoreductase subunit A6
**O14561**	NDUFAB1	0.366	NADH:ubiquinone oxidoreductase subunit AB1
**Q8N183**	NDUFAF2	0.166	NADH:ubiquinone oxidoreductase complex assembly factor 2
**P28331-2**	NDUFS1	0.104	NADH:ubiquinone oxidoreductase core subunit S1
**P19404**	NDUFV2	-0.182	NADH:ubiquinone oxidoreductase core subunit V2
**Q15843**	NEDD8	-0.268	NEDD8 ubiquitin like modifier
**Q6NW34**	NEPRO	0.218	nucleolus and neural progenitor protein
**P48681**	NES	0.297	nestin
**Q99519**	NEU1	-0.248	neuraminidase 1
**Q00653**	NFKB2	0.171	nuclear factor kappa B subunit 2
**Q9UMS0**	NFU1	-0.208	NFU1 iron-sulfur cluster scaffold
**Q6ZNB6-1**	NFXL1	-0.546	nuclear transcription factor, X-box binding like 1
**P14543-1**	NID1	-0.31	nidogen 1
**Q9GZT8**	NIF3L1	-0.168	NGG1 interacting factor 3 like 1
**Q9NYR9-1**	NKIRAS2	-0.162	NFKB inhibitor interacting Ras like 2
**O15226-2**	NKRF	0.207	NFKB repressing factor
**Q96D46**	NMD3	0.185	NMD3 ribosome export adaptor
**P22392-2**	NME2	-0.293	NME/NM23 nucleoside diphosphate kinase 2
**P30419**	NMT1	-0.161	N-myristoyltransferase 1
**Q9ULX3**	NOB1	0.191	NIN1 (RPN12) binding protein 1 homolog
**Q9Y3T9**	NOC2L	0.218	NOC2 like nucleolar associated transcriptional repressor
**Q9BSC4**	NOL10	0.135	nucleolar protein 10
**Q9UGY1**	NOL12	0.28	nucleolar protein 12
**Q96MY1**	NOL4L	-0.214	nucleolar protein 4 like
**Q5C9Z4**	NOM1	0.265	nucleolar protein with MIF4G domain 1
**Q9NPE3**	NOP10	0.202	NOP10 ribonucleoprotein
**P46087-4**	NOP2	0.303	NOP2 nucleolar protein
**Q9Y2X3**	NOP58	0.279	NOP58 ribonucleoprotein
**P29474**	NOS3	0.249	nitric oxide synthase 3
**P55786**	NPEPPS	-0.157	aminopeptidase puromycin sensitive
**O43847-2**	NRDC	-0.289	nardilysin convertase
**O95478**	NSA2	0.274	NSA2 ribosome biogenesis factor

**Q8TCD5**	NT5C	-0.232	5', 3'-nucleotidase, cytosolic
**Q5TFE4**	NT5DC1	-0.228	5'-nucleotidase domain containing 1
**Q86UY8**	NT5DC3	0.304	5'-nucleotidase domain containing 3
**Q8TB37**	NUBPL	0.177	nucleotide binding protein like
**Q9H1E3**	NUCKS1	-0.293	nuclear casein kinase and cyclin dependent kinase substrate 1
**Q8WVJ2**	NUDCD2	-0.178	NudC domain containing 2
**Q8IVD9**	NUDCD3	-0.278	NudC domain containing 3
**Q96DE0**	NUDT16	-0.147	nudix hydrolase 16
**P50583**	NUDT2	-0.163	nudix hydrolase 2
**Q9UKK9**	NUDT5	-0.263	nudix hydrolase 5
**P49790**	NUP153	0.349	nucleoporin 153
**P61970**	NUTF2	-0.232	nuclear transport factor 2
**P04181**	OAT	0.199	ornithine aminotransferase
**O75147**	OBSL1	-0.276	obscurin like cytoskeletal adaptor 1
**O60502**	OGA	0.186	O-GlcNAcase
**Q6N063-1**	OGFOD2	-0.214	2-oxoglutarate and iron dependent oxygenase domain containing 2
**Q9NZT2**	OGFR	-0.104	opioid growth factor receptor
**Q9NTK5-1**	OLA1	-0.195	Obg like ATPase 1
**Q9H6K4**	OPA3	0.419	outer mitochondrial membrane lipid metabolism regulator OPA3
**O14841**	OPLAH	-0.162	5-oxoprolinase, ATP-hydrolysing
**Q13415**	ORC1	0.462	origin recognition complex subunit 1
**Q13416**	ORC2	-0.109	origin recognition complex subunit 2
**P19652**	ORM2	-0.421	orosomucoid 2
**Q9BZF1-1**	OSBPL8	0.175	oxysterol binding protein like 8
**Q96G74-1**	OTUD5	0.323	OTU deubiquitinase 5
**Q8N573-1**	OXR1	-0.269	oxidation resistance 1
**Q9NWU1-1**	OXSM	0.151	3-oxoacyl-ACP synthase, mitochondrial
**Q9BRP4**	PAAF1	-0.182	proteasomal ATPase associated factor 1
**Q9UKS6**	PACSIN3	-0.197	protein kinase C and casein kinase substrate in neurons 3
**Q15102**	PAFAH1B3	-0.209	platelet activating factor acetylhydrolase 1b catalytic subunit 3
**P00439**	PAH	-0.501	phenylalanine hydroxylase
**P22234-2**	PAICS	-0.233	phosphoribosylaminoimidazole carboxylase and phosphoribosylaminoimidazolesuccinocarboxamide s[illegible]
**Q9NWT1**	PAK1IP1	0.154	PAK1 interacting protein 1
**Q9NP74**	PALMD	-0.236	palmdelphin
**O43252**	PAPSS1	-0.179	3'-phosphoadenosine 5'-phosphosulfate synthase 1
**Q86W56**	PARG	-0.135	poly(ADP-ribose) glycohydrolase
**Q9NVD7**	PARVA	-0.256	parvin alpha
**P61457**	PCBD1	-0.205	pterin-4 alpha-carbinolamine dehydratase 1
**Q9H4Z3**	PCIF1	-0.208	PDX1 C-terminal inhibiting factor 1
**Q15154-1**	PCM1	-0.159	pericentriolar material 1
**Q8WW12**	PCNP	-0.181	PEST proteolytic signal containing nuclear protein
**Q9UKL6**	PCTP	-0.231	phosphatidylcholine transfer protein
**Q99447**	PCYT2	-0.377	phosphate cytidylyltransferase 2, ethanolamine
**Q53EL6**	PDCD4	-0.273	programmed cell death 4
**O14737**	PDCD5	-0.28	programmed cell death 5
**Q96HC4**	PDLIM5	0.169	PDZ and LIM domain 5
**Q9NR12**	PDLIM7	0.448	PDZ and LIM domain 7

**Q8NCN5**	PDPR	0.269	pyruvate dehydrogenase phosphatase regulatory subunit
**Q6P996-1**	PDXDC1	-0.296	pyridoxal dependent decarboxylase domain containing 1
**Q5T2W1**	PDZK1	-0.319	PDZ domain containing 1
**P30086**	PEBP1	-0.338	phosphatidylethanolamine binding protein 1
**Q9GZU2**	PEG3	-0.154	paternally expressed 3
**Q9BRX2**	PELO	0.203	pelota mRNA surveillance and ribosome rescue factor
**P12955**	PEPD	-0.305	peptidase D
**O15067**	PFAS	-0.211	phosphoribosylformylglycinamidine synthase
**O15212**	PFDN6	-0.147	prefoldin subunit 6
**P17858-1**	PFKL	-0.219	phosphofructokinase, liver type
**P07737**	PFN1	-0.194	profilin 1
**P18669**	PGAM1	-0.185	phosphoglycerate mutase 1
**Q96HS1-1**	PGAM5	0.166	PGAM family member 5, mitochondrial serine/threonine protein phosphatase
**P00558**	PGK1	-0.148	phosphoglycerate kinase 1
**O95336**	PGLS	-0.252	6-phosphogluconolactonase
**P36871-1**	PGM1	-0.387	phosphoglucomutase 1
**Q96G03**	PGM2	-0.176	phosphoglucomutase 2
**A6NDG6**	PGP	-0.33	phosphoglycolate phosphatase
**Q9NXJ5**	PGPEP1	-0.384	pyroglutamyl-peptidase I
**Q8IZ21-2**	PHACTR4	-0.201	phosphatase and actin regulator 4
**Q9H814**	PHAX	-0.195	phosphorylated adaptor for RNA export
**Q99623**	PHB2	0.166	prohibitin 2
**P46019**	PHKA2	-0.122	phosphorylase kinase regulatory subunit alpha 2
**Q8WV24**	PHLDA1	0.194	pleckstrin homology like domain family A member 1
**Q9BTU6**	PI4K2A	0.199	phosphatidylinositol 4-kinase type 2 alpha
**Q9NWS0-1**	PIH1D1	-0.176	PIH1 domain containing 1
**Q9Y237-2**	PIN4	-0.194	peptidylprolyl cis/trans isomerase, NIMA-interacting 4
**Q9P0Z9**	PIPOX	-0.141	pipecolic acid and sarcosine oxidase
**O00625**	PIR	-0.254	pirin
**Q5JRX3-2**	PITRM1	0.316	pitrilysin metallopeptidase 1
**P30613**	PKLR	-0.336	pyruvate kinase L/R
**Q16512-2**	PKN1	-0.23	protein kinase N1
**Q01970**	PLCB3	-0.225	phospholipase C beta 3
**Q99541**	PLIN2	0.314	perilipin 2
**P53350**	PLK1	0.19	polo like kinase 1
**Q02809-2**	PLOD1	-0.169	procollagen-lysine,2-oxoglutarate 5-dioxygenase 1
**O94903**	PLPBP	-0.228	pyridoxal phosphate binding protein
**O43660**	PLRG1	0.11	pleiotropic regulator 1
**P13797**	PLS3	-0.198	plastin 3
**O43157**	PLXNB1	-0.147	plexin B1
**O15305**	PMM2	-0.205	phosphomannomutase 2
**O75439**	PMPCB	0.168	peptidase, mitochondrial processing beta subunit
**Q9NRX1**	PNO1	0.325	partner of NOB1 homolog
**P41247**	PNPLA4	0.246	patatin like phospholipase domain containing 4
**Q9NVS9**	PNPO	-0.238	pyridoxamine 5'-phosphate oxidase
**P09884**	POLA1	-0.279	DNA polymerase alpha 1, catalytic subunit
**P28340**	POLD1	-0.277	DNA polymerase delta 1, catalytic subunit

**P49005**	POLD2	-0.281	DNA polymerase delta 2, accessory subunit
**Q07864**	POLE	-0.225	DNA polymerase epsilon, catalytic subunit
**Q9NRF9**	POLE3	-0.152	DNA polymerase epsilon 3, accessory subunit
**P54098**	POLG	0.173	DNA polymerase gamma, catalytic subunit
**Q06203**	PPAT	-0.308	phosphoribosyl pyrophosphate amidotransferase
**Q9HAB8**	PPCS	-0.184	phosphopantothenoylcysteine synthetase
**P62937**	PPIA	-0.179	peptidylprolyl isomerase A
**P45877**	PPIC	-0.171	peptidylprolyl isomerase C
**Q9H2H8-1**	PPIL3	-0.112	peptidylprolyl isomerase like 3
**Q9ULR3**	PPM1H	-0.216	protein phosphatase, Mg2+/Mn2+ dependent 1H
**Q96C90**	PPP1R14B	-0.344	protein phosphatase 1 regulatory inhibitor subunit 14B
**Q6NYC8**	PPP1R18	0.405	protein phosphatase 1 regulatory subunit 18
**Q6ZMI0-1**	PPP1R21	-0.18	protein phosphatase 1 regulatory subunit 21
**Q15435**	PPP1R7	-0.255	protein phosphatase 1 regulatory subunit 7
**P60510**	PPP4C	-0.153	protein phosphatase 4 catalytic subunit
**Q8TF05**	PPP4R1	-0.229	protein phosphatase 4 regulatory subunit 1
**Q5H9R7-5**	PPP6R3	-0.134	protein phosphatase 6 regulatory subunit 3
**P32119**	PRDX2	-0.195	peroxiredoxin 2
**P30041**	PRDX6	-0.284	peroxiredoxin 6
**P48147**	PREP	-0.193	prolyl endopeptidase
**P49643**	PRIM2	-0.161	DNA primase subunit 2
**P17612**	PRKACA	-0.163	protein kinase cAMP-activated catalytic subunit alpha
**P10644**	PRKAR1A	-0.292	protein kinase cAMP-dependent type I regulatory subunit alpha
**Q99873**	PRMT1	-0.131	protein arginine methyltransferase 1
**O14744**	PRMT5	-0.116	protein arginine methyltransferase 5
**O43395**	PRPF3	0.128	pre-mRNA processing factor 3
**Q8NAV1-1**	PRPF38A	0.142	pre-mRNA processing factor 38A
**O43172**	PRPF4	0.093	pre-mRNA processing factor 4
**Q14558-2**	PRPSAP1	-0.128	phosphoribosyl pyrophosphate synthetase associated protein 1
**Q8TBF2-8**	PRXL2B	-0.219	peroxiredoxin like 2B
**P07602-3**	PSAP	-0.317	prosaposin
**Q9Y617**	PSAT1	-0.263	phosphoserine aminotransferase 1
**Q16401**	PSMD5	-0.203	proteasome 26S subunit, non-ATPase 5
**Q06323**	PSME1	-0.422	proteasome activator subunit 1
**Q9UL46**	PSME2	-0.305	proteasome activator subunit 2
**Q92530**	PSMF1	-0.2	proteasome inhibitor subunit 1
**O95456**	PSMG1	-0.193	proteasome assembly chaperone 1
**P78330**	PSPH	-0.372	phosphoserine phosphatase
**O95758-4**	PTBP3	0.181	polypyrimidine tract binding protein 3
**Q14914**	PTGR1	0.22	prostaglandin reductase 1
**Q13308-6**	PTK7	-0.236	protein tyrosine kinase 7 (inactive)
**Q86YD1-1**	PTOV1	-0.184	PTOV1 extended AT-hook containing adaptor protein
**Q15257**	PTPA	-0.209	protein phosphatase 2 phosphatase activator
**P10586-1**	PTPRF	-0.264	protein tyrosine phosphatase receptor type F
**Q86Y79**	PTRH1	0.277	peptidyl-tRNA hydrolase 1 homolog
**Q9Y3E5**	PTRH2	0.258	peptidyl-tRNA hydrolase 2
**Q15397**	PUM3	0.392	pumilio RNA binding family member 3

**Q3MIT2**	PUS10	-0.311	pseudouridine synthase 10
**Q96C36**	PYCR2	0.158	pyrroline-5-carboxylate reductase 2
**P06737**	PYGL	-0.273	glycogen phosphorylase L
**Q9BRP8**	PYM1	-0.178	PYM homolog 1, exon junction complex associated factor
**P09417**	QDPR	-0.126	quinoid dihydropteridine reductase
**Q15274**	QPRT	-0.279	quinolinate phosphoribosyltransferase
**Q6ZRP7**	QSOX2	-0.117	quiescin sulfhydryl oxidase 2
**Q9BXR0**	QTRT1	-0.118	queuine tRNA-ribosyltransferase catalytic subunit 1
**P61026**	RAB10	0.301	RAB10, member RAS oncogene family
**Q9NP72**	RAB18	0.202	RAB18, member RAS oncogene family
**Q9UL26**	RAB22A	0.211	RAB22A, member RAS oncogene family
**P51159-1**	RAB27A	0.167	RAB27A, member RAS oncogene family
**P61019**	RAB2A	0.147	RAB2A, member RAS oncogene family
**P20338**	RAB4A	-0.202	RAB4A, member RAS oncogene family
**Q9BUV8-5**	RAB5IF	0.231	RAB5 interacting factor
**P51149**	RAB7A	0.31	RAB7A, member RAS oncogene family
**P51151**	RAB9A	0.143	RAB9A, member RAS oncogene family
**Q9UI14**	RABAC1	-0.221	Rab acceptor 1
**Q9Y3P9**	RABGAP1	-0.255	RAB GTPase activating protein 1
**Q3YEC7-1**	RABL6	-0.187	RAB, member RAS oncogene family like 6
**Q9H0H5**	RACGAP1	0.267	Rac GTPase activating protein 1
**P63244**	RACK1	-0.135	receptor for activated C kinase 1
**P78406**	RAE1	0.199	ribonucleic acid export 1
**P11234-2**	RALB	0.208	RAS like proto-oncogene B
**Q9H6Z4**	RANBP3	-0.202	RAN binding protein 3
**P52306-5**	RAP1GDS1	-0.304	Rap1 GTPase-GDP dissociation stimulator 1
**O75884**	RBBP9	-0.254	RB binding protein 9, serine hydrolase
**Q9H477**	RBKS	-0.453	ribokinase
**Q8IXT5**	RBM12B	0.157	RNA binding motif protein 12B
**Q96PK6-1**	RBM14	0.169	RNA binding motif protein 14
**Q96T37**	RBM15	0.162	RNA binding motif protein 15
**Q9Y4C8**	RBM19	0.155	RNA binding motif protein 19
**P52756**	RBM5	0.14	RNA binding motif protein 5
**P02753**	RBP4	-0.5	retinol binding protein 4
**Q9P2K3-3**	RCOR3	-0.204	REST corepressor 3
**Q9HBH5**	RDH14	0.166	retinol dehydrogenase 14
**Q00765**	REEP5	0.187	receptor accessory protein 5
**P78509**	RELN	-0.155	reelin
**Q86VR2**	RETREG3	-0.211	reticulophagy regulator family member 3
**P35250-1**	RFC2	-0.171	replication factor C subunit 2
**Q15382**	RHEB	0.19	Ras homolog, mTORC1 binding
**P84095**	RHOG	0.137	ras homolog family member G
**P52758**	RIDA	-0.24	reactive intermediate imine deaminase A homolog
**Q99496**	RNF2	0.126	ring finger protein 2
**Q63HN8-4**	RNF213	-0.157	ring finger protein 213
**Q9H4A4**	RNPEP	-0.202	arginyl aminopeptidase
**Q9H6T3**	RPAP3	-0.133	RNA polymerase II associated protein 3

**Q07020**	RPL18	0.672	ribosomal protein L18
**P61254**	RPL26	0.268	ribosomal protein L26
**P46776**	RPL27A	0.28	ribosomal protein L27a
**Q96P16-1**	RPRD1A	-0.181	regulation of nuclear pre-mRNA domain containing 1A
**P63220**	RPS21	-0.154	ribosomal protein S21
**P62979**	RPS27A	0.254	ribosomal protein S27a
**P51812**	RPS6KA3	-0.3	ribosomal protein S6 kinase A3
**P55042**	RRAD	1.406	RRAD, Ras related glycolysis inhibitor and calcium channel regulator
**Q7L523**	RRAGA	0.3	Ras related GTP binding A
**Q9HB90**	RRAGC	0.163	Ras related GTP binding C
**P62070-4**	RRAS2	0.343	RAS related 2
**P23921**	RRM1	-0.104	ribonucleotide reductase catalytic subunit M1
**P56182**	RRP1	0.16	ribosomal RNA processing 1
**Q5JTH9**	RRP12	0.265	ribosomal RNA processing 12 homolog
**O43159**	RRP8	0.167	ribosomal RNA processing 8
**Q6NW29**	RWDD4	-0.289	RWD domain containing 4
**P28702-3**	RXRB	-0.153	retinoid X receptor beta
**P23297**	S100A1	-0.494	S100 calcium binding protein A1
**P31949**	S100A11	0.64	S100 calcium binding protein A11
**Q96FQ6**	S100A16	0.236	S100 calcium binding protein A16
**P06703**	S100A6	0.294	S100 calcium binding protein A6
**P25815**	S100P	0.238	S100 calcium binding protein P
**P35542**	SAA4	-0.307	serum amyloid A4, constitutive
**Q9Y3Z3**	SAMHD1	-0.158	SAM and HD domain containing deoxynucleoside triphosphate triphosphohydrolase 1
**O00422**	SAP18	0.123	Sin3A associated protein 18
**Q9Y6B6**	SAR1B	-0.195	secretion associated Ras related GTPase 1B
**Q8WTV0-2**	SCARB1	-0.167	scavenger receptor class B member 1
**O00767**	SCD	-0.287	stearoyl-CoA desaturase
**P18827**	SDC1	-0.343	syndecan 1
**P34741**	SDC2	-0.751	syndecan 2
**O00560**	SDCBP	0.304	syndecan binding protein
**Q15436**	SEC23A	-0.149	Sec23 homolog A, coat complex II component
**Q9Y6Y8**	SEC23IP	-0.142	SEC23 interacting protein
**O95486**	SEC24A	-0.314	SEC24 homolog A, COPII coat complex component
**O94855-2**	SEC24D	-0.183	SEC24 homolog D, COPII coat complex component
**O94979-8**	SEC31A	-0.219	SEC31 homolog A, COPII coat complex component
**Q13228-4**	SELENBP1	-0.296	selenium binding protein 1
**O60613**	SELENOF	-0.361	selenoprotein F
**P49903**	SEPHS1	-0.158	selenophosphate synthetase 1
**Q99611**	SEPHS2	-0.24	selenophosphate synthetase 2
**Q9P0V9-2**	SEPTIN10	0.204	septin 10
**Q9UHD8-1**	SEPTIN9	-0.134	septin 9
**Q8NC51-1**	SERBP1	-0.113	SERPINE1 mRNA binding protein 1
**P01009-1**	SERPINA1	-0.229	serpin family A member 1
**P05154**	SERPINA5	-0.265	serpin family A member 5
**P05543**	SERPINA7	-0.328	serpin family A member 7
**P35237**	SERPINB6	-0.199	serpin family B member 6

**P05546**	SERPIND1	-0.386	serpin family D member 1
**P05121**	SERPINE1	0.565	serpin family E member 1
**P08697-1**	SERPINF2	-0.529	serpin family F member 2
**P50454**	SERPINH1	0.454	serpin family H member 1
**P58004**	SESN2	0.593	sestrin 2
**Q86TU7**	SETD3	-0.239	SET domain containing 3, actin histidine methyltransferase
**Q15428**	SF3A2	0.196	splicing factor 3a subunit 2
**P31947**	SFN	0.368	stratifin
**P51688**	SGSH	-0.261	N-sulfoglucosamine sulfohydrolase
**Q9H299**	SH3BGRL3	0.157	SH3 domain binding glutamate rich protein like 3
**Q9P0V3**	SH3BP4	0.429	SH3 domain binding protein 4
**Q8NEM2**	SHCBP1	0.341	SHC binding and spindle associated 1
**Q9NUL5**	SHFL	-0.132	shiftless antiviral inhibitor of ribosomal frameshifting
**P34896**	SHMT1	-0.364	serine hydroxymethyltransferase 1
**P34897-1**	SHMT2	0.152	serine hydroxymethyltransferase 2
**A0A0B4J2A0**	SHPK	-0.224	sedoheptulokinase
**Q2M3G4-1**	SHROOM1	-0.276	shroom family member 1
**Q9NXA8-1**	SIRT5	-0.135	sirtuin 5
**P53985**	SLC16A1	0.144	solute carrier family 16 member 1
**O15427**	SLC16A3	-0.196	solute carrier family 16 member 3
**O15403**	SLC16A6	0.62	solute carrier family 16 member 6
**Q15758**	SLC1A5	0.231	solute carrier family 1 member 5
**Q8WUM9**	SLC20A1	0.266	solute carrier family 20 member 1
**Q6NUK1**	SLC25A24	0.155	solute carrier family 25 member 24
**P12236**	SLC25A6	0.379	solute carrier family 25 member 6
**O14975**	SLC27A2	-0.237	solute carrier family 27 member 2
**Q9Y6M5**	SLC30A1	0.565	solute carrier family 30 member 1
**Q9HBR0**	SLC38A10	-0.208	solute carrier family 38 member 10
**Q96QD8**	SLC38A2	0.477	solute carrier family 38 member 2
**Q99624**	SLC38A3	-0.26	solute carrier family 38 member 3
**Q9ULF5**	SLC39A10	0.807	solute carrier family 39 member 10
**Q15043**	SLC39A14	-0.291	solute carrier family 39 member 14
**Q6P5W5-2**	SLC39A4	0.174	solute carrier family 39 member 4
**P08195-4**	SLC3A2	0.2	solute carrier family 3 member 2
**Q9Y6M7-7**	SLC4A7	0.226	solute carrier family 4 member 7
**P30825**	SLC7A1	0.296	solute carrier family 7 member 1
**Q9UPY5**	SLC7A11	0.868	solute carrier family 7 member 11
**O14745**	SLC9A3R1	-0.348	SLC9A3 regulator 1
**Q15599**	SLC9A3R2	-0.233	SLC9A3 regulator 2
**Q9NWH9-1**	SLTM	0.153	SAFB like transcription modulator
**Q8IY18**	SMC5	0.144	structural maintenance of chromosomes 5
**Q9H0W8**	SMG9	0.137	SMG9 nonsense mediated mRNA decay factor
**P53814-6**	SMTN	0.409	smoothelin
**Q2TAY7**	SMU1	0.128	SMU1 DNA replication regulator and spliceosomal factor
**Q6GMV2**	SMYD5	-0.269	SMYD family member 5
**Q8TAD8**	SNIP1	0.201	Smad nuclear interacting protein 1
**Q13596**	SNX1	-0.131	sorting nexin 1

**Q9NRS6**	SNX15	-0.144	sorting nexin 15
**O60749**	SNX2	-0.131	sorting nexin 2
**Q9Y5X3**	SNX5	-0.195	sorting nexin 5
**Q00796**	SORD	-0.37	sorbitol dehydrogenase
**Q92673**	SORL1	-0.693	sortilin related receptor 1
**Q99523**	SORT1	-0.371	sortilin 1
**O75391**	SPAG7	-0.377	sperm associated antigen 7
**O60271**	SPAG9	0.119	sperm associated antigen 9
**Q69YQ0-1**	SPECC1L	0.128	sperm antigen with calponin homology and coiled-coil domains 1 like
**Q9H2V7**	SPNS1	0.155	sphingolipid transporter 1 (putative)
**P10451**	SPP1	0.409	secreted phosphoprotein 1
**P35270**	SPR	-0.246	sepiapterin reductase
**Q01082-3**	SPTBN1	0.667	spectrin beta, non-erythrocytic 1
**O15270**	SPTLC2	0.17	serine palmitoyltransferase long chain base subunit 2
**Q68D10**	SPTY2D1	0.457	SPT2 chromatin protein domain containing 1
**Q14534**	SQLE	-0.16	squalene epoxidase
**Q9Y6N5**	SQOR	0.387	sulfide quinone oxidoreductase
**Q13501-1**	SQSTM1	0.29	sequestosome 1
**P19623**	SRM	-0.154	spermidine synthase
**P49458**	SRP9	-0.18	signal recognition particle 9
**Q01130**	SRSF2	-0.411	serine and arginine rich splicing factor 2
**Q9BYN0**	SRXN1	0.908	sulfiredoxin 1
**P15907**	ST6GAL1	-0.263	ST6 beta-galactoside alpha-2,6-sialyltransferase 1
**Q9Y365**	STARD10	-0.209	StAR related lipid transfer domain containing 10
**Q658P3-2**	STEAP3	-0.538	STEAP3 metalloreductase
**O94804**	STK10	0.351	serine/threonine kinase 10
**Q13043-1**	STK4	0.238	serine/threonine kinase 4
**P16949-1**	STMN1	-0.352	stathmin 1
**P27105**	STOM	0.178	stomatin
**Q13277**	STX3	0.379	syntaxin 3
**O15400**	STX7	0.178	syntaxin 7
**P50225**	SULT1A1	-0.248	sulfotransferase family 1A member 1
**P0DMM9**	SULT1A3/SULT1A4	-0.285	sulfotransferase family 1A member 3
**Q06520**	SULT2A1	-0.447	sulfotransferase family 2A member 1
**O94901-9**	SUN1	0.239	Sad1 and UNC84 domain containing 1
**O95425**	SVIL	0.327	supervillin
**Q9UH65**	SWAP70	0.145	switching B cell complex subunit SWAP70
**P37802-2**	TAGLN2	0.22	transgelin 2
**P37837**	TALDO1	0.172	transaldolase 1
**Q86VP1**	TAX1BP1	0.315	Tax1 binding protein 1
**O75347**	TBCA	-0.226	tubulin folding cofactor A
**Q9Y4C2-1**	TCAF1	-0.268	TRPM8 channel associated factor 1
**O75764**	TCEA3	-0.303	transcription elongation factor A3
**Q9UGI8**	TES	0.244	testin LIM domain protein
**Q96BS2**	TESC	-0.283	tescalcin
**P02787**	TF	-0.297	transferrin
**Q00059**	TFAM	0.489	transcription factor A, mitochondrial

**Q9H5Q4**	TFB2M	0.368	transcription factor B2, mitochondrial
**P01137**	TGFB1	0.475	transforming growth factor beta 1
**Q03167**	TGFBR3	-0.129	transforming growth factor beta receptor 3
**Q96EK4**	THAP11	-0.217	THAP domain containing 11
**P07996**	THBS1	0.311	thrombospondin 1
**P52888**	THOP1	-0.206	thimet oligopeptidase 1
**Q9P016**	THYN1	-0.112	thymocyte nuclear protein 1
**Q9Y5L4**	TIMM13	-0.222	translocase of inner mitochondrial membrane 13
**Q9UDY2**	TJP2	-0.172	tight junction protein 2
**Q3LXA3**	TKFC	-0.402	triokinase and FMN cyclase
**Q9UM00**	TMCO1	0.195	transmembrane and coiled-coil domains 1
**Q9H061**	TMEM126A	0.305	transmembrane protein 126A
**P57088**	TMEM33	0.239	transmembrane protein 33
**Q9BUB7**	TMEM70	0.36	transmembrane protein 70
**P42167**	TMPO	0.222	thymopoietin
**P63313**	TMSB10/TMSB4X	0.374	thymosin beta 4 X-linked
**O00220**	TNFRSF10A	0.233	TNF receptor superfamily member 10a
**O14787-2**	TNPO2	-0.223	transportin 2
**Q68CZ2-1**	TNS3	-0.172	tensin 3
**Q8IZW8**	TNS4	0.636	tensin 4
**Q15388**	TOMM20	-0.2	translocase of outer mitochondrial membrane 20
**Q15785**	TOMM34	0.342	translocase of outer mitochondrial membrane 34
**Q96HA7**	TONSL	-0.137	tonsoku like, DNA repair protein
**P11387**	TOP1	0.488	DNA topoisomerase I
**Q5JTV8**	TOR1AIP1	0.254	torsin 1A interacting protein 1
**P60174**	TPI1	-0.194	triosephosphate isomerase 1
**Q9ULW0**	TPX2	0.518	TPX2 microtubule nucleation factor
**Q9Y296**	TRAPPC4	0.114	trafficking protein particle complex 4
**Q9BQ61**	TRIR	-0.279	telomerase RNA component interacting RNase
**Q7L0Y3**	TRMT10C	0.301	tRNA methyltransferase 10C, mitochondrial RNase P subunit
**O95900**	TRUB2	0.173	TruB pseudouridine synthase family member 2
**Q9H0U9**	TSPYL1	0.203	TSPY like 1
**Q9H2G4**	TSPYL2	0.495	TSPY like 2
**Q13630**	TSTA3	-0.139	tissue specific transplantation antigen P35B
**Q5R3I4**	TTC38	-0.409	tetratricopeptide repeat domain 38
**Q14166**	TTLL12	-0.167	tubulin tyrosine ligase like 12
**Q13509**	TUBB3	0.209	tubulin beta 3 class III
**Q9BUF5**	TUBB6	0.177	tubulin beta 6 class V
**Q96CW5**	TUBGCP3	-0.102	tubulin gamma complex associated protein 3
**P49411**	TUFM	0.155	Tu translation elongation factor, mitochondrial
**Q6IBS0**	TWF2	-0.166	twinfilin actin binding protein 2
**P40222**	TXLNA	-0.111	taxilin alpha
**Q9BRA2**	TXNDC17	-0.222	thioredoxin domain containing 17
**P83876**	TXNL4A	-0.296	thioredoxin like 4A
**Q16881**	TXNRD1	0.246	thioredoxin reductase 1
**P19971-2**	TYMP	-0.242	thymidine phosphorylase
**P26368**	U2AF2	-0.136	U2 small nuclear RNA auxiliary factor 2

**Q3KQV9**	UAP1L1	-0.232	UDP-N-acetylglucosamine pyrophosphorylase 1 like 1
**P22314**	UBA1	-0.156	ubiquitin like modifier activating enzyme 1
**Q8TBC4-1**	UBA3	-0.13	ubiquitin like modifier activating enzyme 3
**Q9GZZ9-1**	UBA5	-0.186	ubiquitin like modifier activating enzyme 5
**P63279**	UBE2I	-0.155	ubiquitin conjugating enzyme E2 I
**P68036**	UBE2L3	-0.161	ubiquitin conjugating enzyme E2 L3
**P61081**	UBE2M	-0.144	ubiquitin conjugating enzyme E2 M
**P61088**	UBE2N	-0.167	ubiquitin conjugating enzyme E2 N
**Q9NPD8**	UBE2T	-0.17	ubiquitin conjugating enzyme E2 T
**Q13404-1**	UBE2V1	-0.234	ubiquitin conjugating enzyme E2 V1
**O95155-4**	UBE4B	-0.137	ubiquitination factor E4B
**Q8WVY7**	UBLCP1	-0.153	ubiquitin like domain containing CTD phosphatase 1
**Q8N806**	UBR7	-0.183	ubiquitin protein ligase E3 component n-recognin 7 (putative)
**Q92575**	UBXN4	0.109	UBX domain protein 4
**Q6UWM9**	UGT2A3	-0.122	UDP glucuronosyltransferase family 2 member A3
**Q6BDS2**	UHRF1BP1	-0.142	UHRF1 binding protein 1
**Q9BRT2**	UQCC2	-0.174	ubiquinol-cytochrome c reductase complex assembly factor 2
**Q8TCY9**	URGCP	0.205	upregulator of cell proliferation
**P06132**	UROD	-0.269	uroporphyrinogen decarboxylase
**Q92995**	USP13	-0.223	ubiquitin specific peptidase 13
**Q8TED0**	UTP15	0.192	UTP15 small subunit processome component
**Q9Y5J1**	UTP18	0.146	UTP18 small subunit processome component
**Q9NQZ2**	UTP3	0.239	UTP3 small subunit processome component
**Q969X6**	UTP4	0.259	UTP4 small subunit processome component
**Q9P0L0-2**	VAPA	0.27	VAMP associated protein A
**P26640**	VARS	-0.134	valyl-tRNA synthetase 1
**Q6EMK4**	VASN	-0.294	vasorin
**P50552**	VASP	0.159	vasodilator stimulated phosphoprotein
**Q99536**	VAT1	-0.153	vesicle amine transport 1
**P52735**	VAV2	-0.23	vav guanine nucleotide exchange factor 2
**P61758**	VBP1	-0.15	VHL binding protein 1
**P18206**	VCL	-0.176	vinculin
**P21796**	VDAC1	0.133	voltage dependent anion channel 1
**P45880-1**	VDAC2	0.273	voltage dependent anion channel 2
**P09327**	VIL1	-0.111	villin 1
**Q9H9C1-1**	VIPAS39	-0.277	VPS33B interacting protein, apical-basolateral polarity regulator, spe-39 homolog
**Q9BRG1**	VPS25	-0.168	vacuolar protein sorting 25 homolog
**Q9UBQ0**	VPS29	-0.15	VPS29 retromer complex component
**Q96QK1**	VPS35	-0.191	VPS35 retromer complex component
**Q96JG6**	VPS50	-0.203	VPS50 subunit of EARP/GARPII complex
**P62760**	VSNL1	-0.472	visinin like 1
**Q9UGM6**	WARS2	0.255	tryptophanyl tRNA synthetase 2, mitochondrial
**Q2M389**	WASHC4	-0.188	WASH complex subunit 4
**Q12768**	WASHC5	-0.209	WASH complex subunit 5
**Q9GZL7**	WDR12	-0.16	WD repeat domain 12
**Q9UNX4**	WDR3	0.172	WD repeat domain 3
**Q8NI36**	WDR36	0.152	WD repeat domain 36

**Q6RFH5**	WDR74	0.17	WD repeat domain 74
**Q562E7**	WDR81	-0.12	WD repeat domain 81
**A4D1P6**	WDR91	-0.203	WD repeat domain 91
**Q9Y3S1**	WNK2	-0.646	WNK lysine deficient protein kinase 2
**Q9HCS7**	XAB2	0.145	XPA binding protein 2
**O14980**	XPO1	-0.114	exportin 1
**Q9C0E2**	XPO4	-0.252	exportin 4
**O75191**	XYLB	-0.235	xylulokinase
**P54577**	YARS1	-0.137	tyrosyl-tRNA synthetase 1
**P67809**	YBX1	-0.239	Y-box binding protein 1
**Q5BJH7**	YIF1B	0.198	Yip1 interacting factor homolog B, membrane trafficking protein
**O15498**	YKT6	0.243	YKT6 v-SNARE homolog
**Q86U90**	YRDC	0.242	yrdC N6-threonylcarbamoyltransferase domain containing
**Q96MU7-1**	YTHDC1	0.142	YTH domain containing 1
**P62258**	YWHAE	-0.18	tyrosine 3-monooxygenase/tryptophan 5-monooxygenase activation protein epsilon
**O95625**	ZBTB11	0.191	zinc finger and BTB domain containing 11
**O75152**	ZC3H11A	0.22	zinc finger CCCH-type containing 11A
**Q8WU90**	ZC3H15	-0.154	zinc finger CCCH-type containing 15
**Q7Z2W4**	ZC3HAV1	0.147	zinc finger CCCH-type containing, antiviral 1
**Q9C0B9**	ZCCHC2	-0.231	zinc finger CCHC-type containing 2
**Q8N5A5**	ZGPAT	0.186	zinc finger CCCH-type and G-patch domain containing
**Q14202**	ZMYM3	-0.318	zinc finger MYM-type containing 3
**Q8NC26**	ZNF114	0.394	zinc finger protein 114
**O75362**	ZNF217	-0.113	zinc finger protein 217
**Q9P2Y4**	ZNF219	-0.193	zinc finger protein 219
**Q5BKZ1**	ZNF326	0.112	zinc finger protein 326
**Q969S3**	ZNF622	0.166	zinc finger protein 622
**Q9H900-1**	ZWILCH	-0.141	zwilch kinetochore protein
**Q9C0D3**	ZYG11B	-0.191	zyg-11 family member B, cell cycle regulator
**O43149**	ZZEF1	-0.307	zinc finger ZZ-type and EF-hand domain containing 1
**P00374**		-0.26	
**P00761**		0.251	
**P62158**		-0.273	
**P69905**		1.153	

List of proteins deregulated in HepG2 cells after 24h exposure to CDDP 10 mM

ID	Symbol	Expr Log Ratio	Entrez Gene Name
**Q9NQ94**	A1CF	-0.129	APOBEC1 complementation factor
**Q13685**	AAMP	-0.192	angio associated migratory cell protein
**Q9NRN7**	AASDHPPT	0.193	aminoadipate-semialdehyde dehydrogenase-phosphopantetheinyl transferase
**O95477**	ABCA1	-0.274	ATP binding cassette subfamily A member 1
**Q8TB40**	ABHD4	0.421	abhydrolase domain containing 4
**Q9BV23**	ABHD6	0.18	abhydrolase domain containing 6
**Q9ULW3**	ABT1	-0.141	activator of basal transcription 1
**Q13085-4**	ACACA	-0.274	acetyl-CoA carboxylase alpha
**P45954**	ACADSB	0.144	acyl-CoA dehydrogenase short/branched chain
**Q15057**	ACAP2	-0.229	ArfGAP with coiled-coil, ankyrin repeat and PH domains 2
**P24752**	ACAT1	0.144	acetyl-CoA acetyltransferase 1
**Q15067**	ACOX1	0.226	acyl-CoA oxidase 1
**P11117**	ACP2	0.171	acid phosphatase 2, lysosomal
**O95573**	ACSL3	0.168	acyl-CoA synthetase long chain family member 3
**O60488-1**	ACSL4	-0.247	acyl-CoA synthetase long chain family member 4
**P14621**	ACYP2	-0.575	acylphosphatase 2
**O14672**	ADAM10	-0.165	ADAM metallopeptidase domain 10
**P35611-3**	ADD1	-0.179	adducin 1
**Q9Y653**	ADGRG1	1.224	adhesion G protein-coupled receptor G1
**Q86SQ4-3**	ADGRG6	-0.2	adhesion G protein-coupled receptor G6
**P55263**	ADK	-0.241	adenosine kinase
**Q16186**	ADRM1	0.216	adhesion regulating molecule 1
**P30566**	ADSL	-0.106	adenylosuccinate lyase
**Q9UHB7-1**	AFF4	0.18	AF4/FMR2 family member 4
**Q6ULP2**	AFTPH	-0.354	aftiphilin
**Q96P47-4**	AGAP3	-0.274	ArfGAP with GTPase domain, ankyrin repeat and PH domain 3
**Q9BSE5**	AGMAT	0.112	agmatinase
**O00468**	AGRN	-0.246	agrin
**P01019**	AGT	-0.539	angiotensinogen
**O95831-1**	AIFM1	0.15	apoptosis inducing factor mitochondria associated 1
**Q96IF1**	AJUBA	-0.206	ajuba LIM protein
**P00568**	AK1	0.227	adenylate kinase 1
**Q9UIJ7-1**	AK3	0.248	adenylate kinase 3
**Q12802-2**	AKAP13	-0.268	A-kinase anchoring protein 13
**Q02040-1**	AKAP17A	0.167	A-kinase anchoring protein 17A
**O43823**	AKAP8	0.218	A-kinase anchoring protein 8
**Q9ULX6**	AKAP8L	0.204	A-kinase anchoring protein 8 like
**O60218**	AKR1B10	0.127	aldo-keto reductase family 1 member B10
**P30837**	ALDH1B1	0.161	aldehyde dehydrogenase 1 family member B1
**P05091**	ALDH2	0.163	aldehyde dehydrogenase 2 family member
**P49419-2**	ALDH7A1	0.326	aldehyde dehydrogenase 7 family member A1

**P49189**	ALDH9A1	0.138	aldehyde dehydrogenase 9 family member A1
**P09972**	ALDOC	-0.123	aldolase, fructose-bisphosphate C
**Q8IWZ3-6**	ANKHD1/ANKHD1-EIF4EBP3	-0.18	ankyrin repeat and KH domain containing 1
**Q92625**	ANKS1A	-0.201	ankyrin repeat and sterile alpha motif domain containing 1A
**Q9NQW6**	ANLN	0.541	anillin actin binding protein
**Q9BZZ5-2**	API5	0.118	apoptosis inhibitor 5
**P02647**	APOA1	-0.233	apolipoprotein A1
**P04114**	APOB	-0.692	apolipoprotein B
**Q9NRW3**	APOBEC3C	0.778	apolipoprotein B mRNA editing enzyme catalytic subunit 3C
**P02649**	APOE	-0.172	apolipoprotein E
**O14791-2**	APOL1	-0.442	apolipoprotein L1
**Q96P48-6**	ARAP1	-0.248	ArfGAP with RhoGAP domain, ankyrin repeat and PH domain 1
**P84085**	ARF5	0.172	ADP ribosylation factor 5
**P78540**	ARG2	0.37	arginase 2
**Q8IWW6-1**	ARHGAP12	-0.17	Rho GTPase activating protein 12
**Q8N392**	ARHGAP18	-0.326	Rho GTPase activating protein 18
**Q9NRY4**	ARHGAP35	-0.224	Rho GTPase activating protein 35
**Q92888-3**	ARHGEF1	-0.151	Rho guanine nucleotide exchange factor 1
**Q8N1W1-6**	ARHGEF28	-0.213	Rho guanine nucleotide exchange factor 28
**Q8NFD5-3**	ARID1B	-0.276	AT-rich interaction domain 1B
**Q8IVW6**	ARID3B	0.246	AT-rich interaction domain 3B
**Q6NXE6-1**	ARMC6	-0.141	armadillo repeat containing 6
**Q5T2E6**	ARMH3	-0.257	armadillo like helical domain containing 3
**P15848**	ARSB	-0.227	arylsulfatase B
**O00192**	ARVCF	0.384	ARVCF delta catenin family member
**Q8N9N2-2**	ASCC1	-0.277	activating signal cointegrator 1 complex subunit 1
**Q9H1I8-1**	ASCC2	-0.255	activating signal cointegrator 1 complex subunit 2
**Q8N3C0**	ASCC3	-0.366	activating signal cointegrator 1 complex subunit 3
**P07306**	ASGR1	-0.156	asialoglycoprotein receptor 1
**P07307**	ASGR2	-0.146	asialoglycoprotein receptor 2
**P08243**	ASNS	-0.189	asparagine synthetase (glutamine-hydrolyzing)
**Q9NWL6**	ASNSD1	-0.23	asparagine synthetase domain containing 1
**P00966**	ASS1	-0.188	argininosuccinate synthase 1
**Q8NBU5**	ATAD1	0.171	ATPase family AAA domain containing 1
**Q6PL18-1**	ATAD2	-0.202	ATPase family AAA domain containing 2
**Q5T9A4**	ATAD3B	-0.234	ATPase family AAA domain containing 3B
**P18847**	ATF3	0.857	activating transcription factor 3
**Q9NT62**	ATG3	-0.156	autophagy related 3
**O95352**	ATG7	-0.262	autophagy related 7
**P98196**	ATP11A	-0.267	ATPase phospholipid transporting 11A
**P05023**	ATP1A1	0.107	ATPase Na+/K+ transporting subunit alpha 1
**P05026**	ATP1B1	0.211	ATPase Na+/K+ transporting subunit beta 1
**P54709**	ATP1B3	0.192	ATPase Na+/K+ transporting subunit beta 3
**Q9UII2**	ATP5IF1	0.346	ATP synthase inhibitory factor subunit 1
**O75947-1**	ATP5PD	0.156	ATP synthase peripheral stalk subunit d
**P18859-2**	ATP5PF	0.208	ATP synthase peripheral stalk subunit F6
**P48047**	ATP5PO	0.263	ATP synthase peripheral stalk subunit OSCP

**O75787**	ATP6AP2	0.316	ATPase H+ transporting accessory protein 2
**P61421**	ATP6V0D1	0.218	ATPase H+ transporting V0 subunit d1
**P21283**	ATP6V1C1	0.137	ATPase H+ transporting V1 subunit C1
**Q9Y5K8**	ATP6V1D	0.118	ATPase H+ transporting V1 subunit D
**Q5TC12**	ATPAF1	0.224	ATP synthase mitochondrial F1 complex assembly factor 1
**P0C7T5**	ATXN1L	-0.679	ataxin 1 like
**O14965**	AURKA	0.3	aurora kinase A
**Q96GD4-5**	AURKB	0.329	aurora kinase B
**Q9NQS1**	AVEN	-0.172	apoptosis and caspase activation inhibitor
**Q8NBF6-1**	AVL9	-0.289	AVL9 cell migration associated
**Q9NY97**	B3GNT2	0.223	UDP-GlcNAc:betaGal beta-1,3-N-acetylglucosaminyltransferase 2
**Q9NXR7-1**	BABAM2	-0.286	BRISC and BRCA1 A complex member 2
**P51572-2**	BCAP31	0.291	B cell receptor associated protein 31
**P56945-6**	BCAR1	0.15	BCAR1 scaffold protein, Cas family member
**O75934**	BCAS2	0.161	BCAS2 pre-mRNA processing factor
**Q86UU0**	BCL9L	0.333	BCL9 like
**Q9NYM9**	BET1L	-0.178	Bet1 golgi vesicular membrane trafficking protein like
**Q9NR09**	BIRC6	-0.219	baculoviral IAP repeat containing 6
**Q14692**	BMS1	-0.191	BMS1 ribosome biogenesis factor
**Q53S33**	BOLA3	0.398	bolA family member 3
**Q86WA6**	BPHL	0.234	biphenyl hydrolase like
**Q5VW32**	BROX	-0.14	BRO1 domain and CAAX motif containing
**Q7KYR7**	BTN2A1	0.386	butyrophilin subfamily 2 member A1
**O43709**	BUD23	-0.218	BUD23 rRNA methyltransferase and ribosome maturation factor
**P41223-2**	BUD31	0.224	BUD31 homolog
**Q9Y6E2**	BZW2	-0.18	basic leucine zipper and W2 domains 2
**Q8N5I9**	C12orf45	0.431	chromosome 12 open reading frame 45
**Q8NDD1**	C1orf131	-0.465	chromosome 1 open reading frame 131
**A6NCS6**	C2orf72	-0.223	chromosome 2 open reading frame 72
**P01024**	C3	-0.205	complement C3
**Q9BRJ6**	C7orf50	-0.393	chromosome 7 open reading frame 50
**P27708**	CAD	-0.154	carbamoyl-phosphate synthetase 2, aspartate transcarbamylase, and dihydroorotase
**Q9BY67-3**	CADM1	-0.302	cell adhesion molecule 1
**Q13557-10**	CAMK2D	-0.221	calcium/calmodulin dependent protein kinase II delta
**P40123**	CAP2	-0.239	cyclase associated actin cytoskeleton regulatory protein 2
**P47755**	CAPZA2	-0.152	capping actin protein of muscle Z-line subunit alpha 2
**P49589-3**	CARS1	-0.24	cysteinyl-tRNA synthetase 1
**O15234**	CASC3	-0.238	CASC3 exon junction complex subunit
**O95810**	CAVIN2	0.326	caveolae associated protein 2
**P45973**	CBX5	0.376	chromobox 5
**Q8IX12**	CCAR1	0.195	cell division cycle and apoptosis regulator 1
**Q8IWD4**	CCDC117	0.627	coiled-coil domain containing 117
**Q96CT7**	CCDC124	-0.187	coiled-coil domain containing 124
**Q6PK04**	CCDC137	-0.473	coiled-coil domain containing 137
**O60826**	CCDC22	-0.17	coiled-coil domain containing 22
**Q8IVM0**	CCDC50	-0.226	coiled-coil domain containing 50
**Q9P031**	CCDC59	-0.365	coiled-coil domain containing 59

**Q3V6T2-1**	CCDC88A	-0.184	coiled-coil domain containing 88A
**P20248**	CCNA2	0.542	cyclin A2
**P14635**	CCNB1	0.245	cyclin B1
**O60563**	CCNT1	0.377	cyclin T1
**P49427**	CDC34	0.133	cell division cycle 34
**O60508**	CDC40	0.125	cell division cycle 40
**Q99618**	CDCA3	0.242	cell division cycle associated 3
**P19022**	CDH2	-0.29	cadherin 2
**O14735**	CDIPT	-0.582	CDP-diacylglycerol--inositol 3-phosphatidyltransferase
**P06493**	CDK1	0.249	cyclin dependent kinase 1
**Q00534**	CDK6	-0.269	cyclin dependent kinase 6
**P50750-2**	CDK9	0.224	cyclin dependent kinase 9
**Q5VV42**	CDKAL1	-0.482	CDK5 regulatory subunit associated protein 1 like 1
**P38936**	CDKN1A	1.8	cyclin dependent kinase inhibitor 1A
**Q9NXV6**	CDKN2AIP	0.328	CDKN2A interacting protein
**Q9UHN6**	CEMIP2	0.255	cell migration inducing hyaluronidase 2
**P49454**	CENPF	0.382	centromere protein F
**Q8TAP6**	CEP76	0.649	centrosomal protein 76
**O00748**	CES2	0.297	carboxylesterase 2
**Q9UEE9-1**	CFDP1	-0.22	craniofacial development protein 1
**P05156**	CFI	-0.343	complement factor I
**Q0VF96**	CGNL1	-0.996	cingulin like 1
**Q13112**	CHAF1B	0.239	chromatin assembly factor 1 subunit B
**O14757**	CHEK1	-0.482	checkpoint kinase 1
**Q8IWX8**	CHERP	0.125	calcium homeostasis endoplasmic reticulum protein
**P35790**	CHKA	-0.42	choline kinase alpha
**Q9Y3E7**	CHMP3	0.294	charged multivesicular body protein 3
**Q9H444**	CHMP4B	0.223	charged multivesicular body protein 4B
**Q8NET6**	CHST13	-0.296	carbohydrate sulfotransferase 13
**Q99828-2**	CIB1	0.283	calcium and integrin binding 1
**O14578-4**	CIT	-0.25	citron rho-interacting serine/threonine kinase
**Q8WWK9**	CKAP2	0.652	cytoskeleton associated protein 2
**Q07065**	CKAP4	0.139	cytoskeleton associated protein 4
**P61024**	CKS1B	0.459	CDC28 protein kinase regulatory subunit 1B
**Q7Z460-1**	CLASP1	-0.237	cytoplasmic linker associated protein 1
**O00299**	CLIC1	0.159	chloride intracellular channel 1
**Q9Y696**	CLIC4	-0.169	chloride intracellular channel 4
**Q9H078-1**	CLPB	-0.119	ClpB homolog, mitochondrial AAA ATPase chaperonin
**O76031**	CLPX	0.191	caseinolytic mitochondrial matrix peptidase chaperone subunit
**I3L2B0**	CLUH	-0.342	clustered mitochondria homolog
**O75153**	CLUH	-0.242	clustered mitochondria homolog
**Q96DG6**	CMBL	0.67	carboxymethylenebutenolidase homolog
**Q8IY22**	CMIP	-0.706	c-Maf inducing protein
**Q9BQ75**	CMSS1	-0.388	cms1 ribosomal small subunit homolog
**P62633**	CNBP	-1.428	CCHC-type zinc finger nucleic acid binding protein
**P62633-6**	CNBP	-1.085	CCHC-type zinc finger nucleic acid binding protein
**Q9H9A5-6**	CNOT10	-0.136	CCR4-NOT transcription complex subunit 10

**Q9UIV1-1**	CNOT7	-0.25	CCR4-NOT transcription complex subunit 7
**Q96BR5**	COA7	0.284	cytochrome c oxidase assembly factor 7 (putative)
**P38432**	COIL	0.193	coilin
**Q9P000-1**	COMMD9	-0.412	COMM domain containing 9
**Q9ULV4-3**	CORO1C	0.152	coronin 1C
**A0A0A6YYL4**	CORO7/CORO7-PAM16	-0.118	coronin 7
**P13073**	COX4I1	0.142	cytochrome c oxidase subunit 4I1
**O75976**	CPD	-0.23	carboxypeptidase D
**Q6PUV4**	CPLX2	-0.609	complexin 2
**P23786**	CPT2	0.254	carnitine palmitoyltransferase 2
**P78560**	CRADD	-0.421	CASP2 and RIPK1 domain containing adaptor with death domain
**P52943**	CRIP2	0.307	cysteine rich protein 2
**Q8IUI8**	CRLF3	-0.161	cytokine receptor like factor 3
**O75534-4**	CSDE1	-0.179	cold shock domain containing E1
**Q12996**	CSTF3	0.318	cleavage stimulation factor subunit 3
**P35221**	CTNNA1	-0.236	catenin alpha 1
**Q9UBT7**	CTNNAL1	-0.135	catenin alpha like 1
**O60716-3**	CTNND1	-0.111	catenin delta 1
**Q9NRF8**	CTPS2	-0.168	CTP synthase 2
**Q6PD62**	CTR9	0.12	CTR9 homolog, Paf1/RNA polymerase II complex component
**P07339**	CTSD	0.189	cathepsin D
**P07711**	CTSL	0.216	cathepsin L
**Q2VPK5**	CTU2	-0.156	cytosolic thiouridylase subunit 2
**P39880-3**	CUX1	-0.454	cut like homeobox 1
**Q2TBE0**	CWF19L2	-0.158	CWF19 like cell cycle control factor 2
**P99999**	CYCS	0.247	cytochrome c, somatic
**Q96SQ9-2**	CYP2S1	0.182	cytochrome P450 family 2 subfamily S member 1
**Q8TAV3**	CYP2W1	-0.27	cytochrome P450 family 2 subfamily W member 1
**Q9Y4D1**	DAAM1	-0.24	dishevelled associated activator of morphogenesis 1
**Q5VWQ8**	DAB2IP	-0.218	DAB2 interacting protein
**Q14118**	DAG1	-0.186	dystroglycan 1
**P51397**	DAP	-0.185	death associated protein
**Q9UK59**	DBR1	0.239	debranching RNA lariats 1
**P11182**	DBT	0.192	dihydrolipoamide branched chain transacylase E2
**P61962**	DCAF7	0.22	DDB1 and CUL4 associated factor 7
**Q9H773**	DCTPP1	-0.19	dCTP pyrophosphatase 1
**Q96GG9**	DCUN1D1	0.209	defective in cullin neddylation 1 domain containing 1
**Q92466**	DDB2	0.556	damage specific DNA binding protein 2
**Q13206**	DDX10	-0.249	DEAD-box helicase 10
**Q96GQ7**	DDX27	-0.238	DEAD-box helicase 27
**Q9Y6V7**	DDX49	-0.234	DEAD-box helicase 49
**Q9NY93**	DDX56	-0.524	DEAD-box helicase 56
**P26196**	DDX6	0.165	DEAD-box helicase 6
**Q8WYQ5-1**	DGCR8	0.301	DGCR8 microprocessor complex subunit
**Q5KSL6**	DGKK	-0.346	diacylglycerol kinase kappa
**Q86SQ9-2**	DHDDS	-0.292	dehydrodolichyl diphosphate synthase subunit
**Q8IY37**	DHX37	-0.171	DEAH-box helicase 37

**Q6P158-1**	DHX57	-0.393	DExH-box helicase 57
**O60610-1**	DIAPH1	-0.14	diaphanous related formin 1
**Q9BTC0**	DIDO1	0.254	death inducer-obliterator 1
**Q9UNQ2**	DIMT1	-0.281	DIMT1 rRNA methyltransferase and ribosome maturation factor
**Q9P265**	DIP2B	-0.22	disco interacting protein 2 homolog B
**Q8IYB7**	DIS3L2	-0.349	DIS3 like 3'-5' exoribonuclease 2
**P09622**	DLD	0.228	dihydrolipoamide dehydrogenase
**Q15398**	DLGAP5	0.383	DLG associated protein 5
**P36957**	DLST	0.17	dihydrolipoamide S-succinyltransferase
**P11532-6**	DMD	-0.125	dystrophin
**Q86Y56**	DNAAF5	-0.288	dynein axonemal assembly factor 5
**P31689**	DNAJA1	0.161	DnaJ heat shock protein family (Hsp40) member A1
**P25686-3**	DNAJB2	0.408	DnaJ heat shock protein family (Hsp40) member B2
**Q8IXB1**	DNAJC10	-0.118	DnaJ heat shock protein family (Hsp40) member C10
**Q9Y5T4**	DNAJC15	-0.695	DnaJ heat shock protein family (Hsp40) member C15
**Q9ULA0**	DNPEP	-0.103	aspartyl aminopeptidase
**Q9BU89**	DOHH	0.275	deoxyhypusine hydroxylase
**Q9BQC3**	DPH2	-0.113	diphthamide biosynthesis 2
**Q9H2P9-5**	DPH5	-0.297	diphthamide biosynthesis 5
**Q16555**	DPYSL2	-0.149	dihydropyrimidinase like 2
**Q8TE96**	DQX1	0.276	DEAQ-box RNA dependent ATPase 1
**Q14919-2**	DRAP1	0.205	DR1 associated protein 1
**Q9Y295**	DRG1	-0.279	developmentally regulated GTP binding protein 1
**P60981-1**	DSTN	0.196	destrin, actin depolymerizing factor
**Q99956**	DUSP9	-0.219	dual specificity phosphatase 9
**O14641**	DVL2	-0.364	dishevelled segment polarity protein 2
**Q14204**	DYNC1H1	-0.118	dynein cytoplasmic 1 heavy chain 1
**P63167**	DYNLL1	0.405	dynein light chain LC8-type 1
**Q99848**	EBNA1BP2	-0.277	EBNA1 binding protein 2
**P42892**	ECE1	-0.323	endothelin converting enzyme 1
**Q13011**	ECH1	0.147	enoyl-CoA hydratase 1
**Q9NTX5**	ECHDC1	0.137	ethylmalonyl-CoA decarboxylase 1
**O75521**	ECI2	0.201	enoyl-CoA delta isomerase 2
**Q9H8V3-3**	ECT2	0.333	epithelial cell transforming 2
**P13639**	EEF2	-0.168	eukaryotic translation elongation factor 2
**Q7Z2Z2**	EFL1	-0.177	elongation factor like GTPase 1
**Q8NDI1**	EHBP1	-0.333	EH domain binding protein 1
**Q9H223**	EHD4	-0.222	EH domain containing 4
**P41567**	EIF1	0.25	eukaryotic translation initiation factor 1
**Q9NZJ5**	EIF2AK3	-0.222	eukaryotic translation initiation factor 2 alpha kinase 3
**P41214**	EIF2D	-0.218	eukaryotic translation initiation factor 2D
**Q9UBQ5**	EIF3K	-0.191	eukaryotic translation initiation factor 3 subunit K
**O60573**	EIF4E2	-0.192	eukaryotic translation initiation factor 4E family member 2
**O43432-3**	EIF4G3	-0.3	eukaryotic translation initiation factor 4 gamma 3
**P55199**	ELL	0.295	elongation factor for RNA polymerase II
**O95163**	ELP1	-0.111	elongator complex protein 1
**Q9H9T3**	ELP3	-0.212	elongator acetyltransferase complex subunit 3

**Q14249**	ENDOG	0.186	endonuclease G
**P13929-1**	ENO3	-0.172	enolase 3
**Q5NDL2**	EOGT	-0.18	EGF domain specific O-linked N-acetylglucosamine transferase
**Q96L91**	EP400	-0.155	E1A binding protein p400
**P29317**	EPHA2	0.697	EPH receptor A2
**P54760**	EPHB4	0.379	EPH receptor B4
**P07099**	EPHX1	0.244	epoxide hydrolase 1
**P34913**	EPHX2	-0.194	epoxide hydrolase 2
**A0A087X1U6**	EPPK1	0.333	epiplakin 1
**P58107**	EPPK1	0.292	epiplakin 1
**P42566**	EPS15	-0.14	epidermal growth factor receptor pathway substrate 15
**Q9H6S3**	EPS8L2	0.279	EPS8 like 2
**P04626**	ERBB2	-0.204	erb-b2 receptor tyrosine kinase 2
**Q96RT1-8**	ERBIN	-0.108	erbb2 interacting protein
**Q8IUD2-1**	ERC1	-0.449	ELKS/RAB6-interacting/CAST family member 1
**P18074-1**	ERCC2	-0.142	ERCC excision repair 2, TFIIH core complex helicase subunit
**P19447**	ERCC3	0.21	ERCC excision repair 3, TFIIH core complex helicase subunit
**Q9Y282-3**	ERGIC3	0.184	ERGIC and golgi 3
**P84090**	ERH	0.201	ERH mRNA splicing and mitosis factor
**B1AK53-1**	ESPN	-0.377	espin
**A0FGR8-2**	ESYT2	0.14	extended synaptotagmin 2
**O60645**	EXOC3	-0.375	exocyst complex component 3
**Q96A65**	EXOC4	-0.318	exocyst complex component 4
**Q9Y2D4**	EXOC6B	-0.557	exocyst complex component 6B
**Q9NQT4**	EXOSC5	0.13	exosome component 5
**Q9Y624**	F11R	0.278	F11 receptor
**Q9UNN5**	FAF1	-0.506	Fas associated factor 1
**Q96CS3**	FAF2	0.16	Fas associated factor family member 2
**Q9Y6X4-1**	FAM169A	0.234	family with sequence similarity 169 member A
**Q9NSI2**	FAM207A	-0.386	family with sequence similarity 207 member A
**O75063**	FAM20B	-0.127	FAM20B glycosaminoglycan xylosylkinase
**Q96KR6**	FAM210B	0.547	family with sequence similarity 210 member B
**Q9H4H8**	FAM83D	0.304	family with sequence similarity 83 member D
**Q52LJ0-2**	FAM98B	0.156	family with sequence similarity 98 member B
**P25445**	FAS	0.834	Fas cell surface death receptor
**P49327**	FASN	-0.246	fatty acid synthase
**Q14517**	FAT1	0.154	FAT atypical cadherin 1
**Q8NEZ5**	FBXO22	0.33	F-box protein 22
**O94868**	FCHSD2	-0.293	FCH and double SH3 domains 2
**P22570-7**	FDXR	0.614	ferredoxin reductase
**Q9BQL6**	FERMT1	-0.094	fermitin family member 1
**P02671-2**	FGA	-0.383	fibrinogen alpha chain
**P22455-1**	FGFR4	-0.536	fibroblast growth factor receptor 4
**P02679**	FGG	-0.364	fibrinogen gamma chain
**Q9Y613**	FHOD1	-0.247	formin homology 2 domain containing 1
**O95302-3**	FKBP9	0.213	FKBP prolyl isomerase 9
**Q14254**	FLOT2	0.138	flotillin 2

**Q9Y5Y0**	FLVCR1	-0.271	feline leukemia virus subgroup C cellular receptor 1
**Q96PY5-3**	FMNL2	-0.27	formin like 2
**P02751-15**	FN1	-0.343	fibronectin 1
**Q53EP0**	FNDC3B	-0.353	fibronectin type III domain containing 3B
**Q5VW36**	FOCAD	-0.309	focadhesin
**P15407**	FOSL1	0.937	FOS like 1, AP-1 transcription factor subunit
**Q8IWF2**	FOXRED2	-0.265	FAD dependent oxidoreductase domain containing 2
**Q86XX4-2**	FRAS1	-1.025	Fraser extracellular matrix complex subunit 1
**Q16658**	FSCN1	0.12	fascin actin-bundling protein 1
**P02792**	FTL	0.311	ferritin light chain
**Q9C0B1-1**	FTO	-0.31	FTO alpha-ketoglutarate dependent dioxygenase
**Q9BTY2**	FUCA2	0.188	alpha-L-fucosidase 2
**Q06546**	GABPA	-0.223	GA binding protein transcription factor subunit alpha
**Q14376**	GALE	-0.107	UDP-galactose-4-epimerase
**O43903**	GAS2	-0.2	growth arrest specific 2
**P04062**	GBA	0.125	glucosylceramidase beta
**Q8IWJ2**	GCC2	0.147	GRIP and coiled-coil domain containing 2
**Q99988**	GDF15	0.839	growth differentiation factor 15
**P31150**	GDI1	0.175	GDP dissociation inhibitor 1
**Q06210**	GFPT1	-0.161	glutamine--fructose-6-phosphate transaminase 1
**Q9BRT9**	GINS4	0.149	GINS complex subunit 4
**Q9Y2X7-3**	GIT1	0.257	GIT ArfGAP 1
**P08034**	GJB1	-0.637	gap junction protein beta 1
**O94925**	GLS	0.611	glutaminase
**P15104**	GLUL	-0.227	glutamate-ammonia ligase
**P17900**	GM2A	0.723	GM2 ganglioside activator
**O60547**	GMDS	-0.255	GDP-mannose 4,6-dehydratase
**O75496**	GMNN	0.403	geminin DNA replication inhibitor
**Q9Y5P6-2**	GMPPB	-0.188	GDP-mannose pyrophosphorylase B
**P29992**	GNA11	0.195	G protein subunit alpha 11
**Q13823**	GNL2	-0.409	G protein nucleolar 2
**Q9BVP2**	GNL3	-0.452	G protein nucleolar 3
**Q9NVN8**	GNL3L	-0.283	G protein nucleolar 3 like
**Q9Y3E0**	GOLT1B	0.141	golgi transport 1B
**P17174**	GOT1	0.297	glutamic-oxaloacetic transaminase 1
**P00505**	GOT2	0.207	glutamic-oxaloacetic transaminase 2
**P35052**	GPC1	0.393	glypican 1
**P51654-1**	GPC3	-0.235	glypican 3
**Q9Y625**	GPC6	-0.563	glypican 6
**Q9NQX3-2**	GPHN	-0.445	gephyrin
**Q13889**	GTF2H3	0.294	general transcription factor IIH subunit 3
**Q9BZE4**	GTPBP4	-0.407	GTP binding protein 4
**Q9NYZ3**	GTSE1	0.454	G2 and S-phase expressed 1
**Q4G148**	GXYLT1	0.389	glucoside xylosyltransferase 1
**O14929**	HAT1	0.107	histone acetyltransferase 1
**Q00341**	HDLBP	-0.231	high density lipoprotein binding protein
**Q86XA9**	HEATR5A	-0.194	HEAT repeat containing 5A

**Q9BYK8**	HELZ2	0.292	helicase with zinc finger 2
**Q15011-1**	HERPUD1	-0.783	homocysteine inducible ER protein with ubiquitin like domain 1
**O94992**	HEXIM1	0.431	HEXIM P-TEFb complex subunit 1
**Q9BTY7**	HGH1	-0.187	HGH1 homolog
**P01892**	HLA-A	0.194	major histocompatibility complex, class I, A
**P05534**	HLA-A	0.236	major histocompatibility complex, class I, A
**Q14527**	HLTF	-0.513	helicase like transcription factor
**Q8TCT9**	HM13	-0.131	histocompatibility minor 13
**P09429**	HMGB1	0.209	high mobility group box 1
**P35914**	HMGCL	0.234	3-hydroxy-3-methylglutaryl-CoA lyase
**Q01581**	HMGCS1	-0.339	3-hydroxy-3-methylglutaryl-CoA synthase 1
**P05204**	HMGN2	0.307	high mobility group nucleosomal binding domain 2
**O75330-3**	HMMR	0.462	hyaluronan mediated motility receptor
**P20823-7**	HNF1A	-0.197	HNF1 homeobox A
**P41235-4**	HNF4A	-0.396	hepatocyte nuclear factor 4 alpha
**Q9UJC3**	HOOK1	-0.162	hook microtubule tethering protein 1
**P00738**	HP	-0.366	haptoglobin
**P37235**	HPCAL1	-0.158	hippocalcin like 1
**P37059**	HSD17B2	0.14	hydroxysteroid 17-beta dehydrogenase 2
**Q9H2F3**	HSD3B7	-0.109	hydroxy-delta-5-steroid dehydrogenase, 3 beta- and steroid delta-isomerase 7
**Q6YN16**	HSDL2	0.191	hydroxysteroid dehydrogenase like 2
**O95757**	HSPA4L	0.327	heat shock protein family A (Hsp70) member 4 like
**P04792**	HSPB1	0.373	heat shock protein family B (small) member 1
**P98160**	HSPG2	-0.538	heparan sulfate proteoglycan 2
**Q92598**	HSPH1	0.204	heat shock protein family H (Hsp110) member 1
**P42858**	HTT	-0.176	huntingtin
**Q7Z6Z7**	HUWE1	-0.149	HECT, UBA and WWE domain containing E3 ubiquitin protein ligase 1
**Q9P2D0**	IBTK	-0.287	inhibitor of Bruton tyrosine kinase
**P05362**	ICAM1	0.174	intercellular adhesion molecule 1
**P48735**	IDH2	0.215	isocitrate dehydrogenase (NADP(+)) 2
**P15260**	IFNGR1	-0.338	interferon gamma receptor 1
**Q12894-2**	IFRD2	-0.283	interferon related developmental regulator 2
**P11717**	IGF2R	-0.234	insulin like growth factor 2 receptor
**P08833**	IGFBP1	1.137	insulin like growth factor binding protein 1
**Q8N6C5-4**	IGSF1	-0.26	immunoglobulin superfamily member 1
**Q70UQ0**	IKBIP	0.437	IKBKB interacting protein
**Q70UQ0-4**	IKBIP	0.375	IKBKB interacting protein
**P40189**	IL6ST	-0.365	interleukin 6 signal transducer
**Q9NQS7**	INCENP	0.535	inner centromere protein
**Q27J81**	INF2	-0.197	inverted formin, FH2 and WH2 domain containing
**P49441**	INPP1	0.378	inositol polyphosphate-1-phosphatase
**Q92835**	INPP5D	-0.299	inositol polyphosphate-5-phosphatase D
**P06213**	INSR	-0.319	insulin receptor
**Q5TA45-5**	INTS11	-0.181	integrator complex subunit 11
**Q96HW7**	INTS4	-0.147	integrator complex subunit 4
**Q9UI26-2**	IPO11	-0.148	importin 11
**Q8TEX9-2**	IPO4	-0.16	importin 4

**O00410-3**	IPO5	-0.147	importin 5
**O95373**	IPO7	-0.21	importin 7
**Q13576-1**	IQGAP2	-0.268	IQ motif containing GTPase activating protein 2
**Q6DN90**	IQSEC1	-0.249	IQ motif and Sec7 domain ArfGEF 1
**Q9H1K1**	ISCU	0.343	iron-sulfur cluster assembly enzyme
**P05161**	ISG15	0.26	ISG15 ubiquitin like modifier
**Q9H9L3**	ISG20L2	-0.198	interferon stimulated exonuclease gene 20 like 2
**Q96CN7**	ISOC1	0.147	isochorismatase domain containing 1
**Q9NPH2**	ISYNA1	0.472	inositol-3-phosphate synthase 1
**P23229-1**	ITGA6	-0.279	integrin subunit alpha 6
**P06756**	ITGAV	0.146	integrin subunit alpha V
**Q14571**	ITPR2	-0.194	inositol 1,4,5-trisphosphate receptor type 2
**P23458**	JAK1	-0.428	Janus kinase 1
**Q7L273**	KCTD9	0.383	potassium channel tetramerization domain containing 9
**Q9Y4C1**	KDM3A	0.45	lysine demethylase 3A
**Q14145**	KEAP1	0.132	kelch like ECH associated protein 1
**Q5T5P2-1**	KIAA1217	-0.24	KIAA1217
**Q9P206-2**	KIAA1522	-0.126	KIAA1522
**Q9BY89-1**	KIAA1671	-0.363	KIAA1671
**Q9ULH0-1**	KIDINS220	-0.213	kinase D interacting substrate 220
**P52732**	KIF11	0.161	kinesin family member 11
**Q9NQT8**	KIF13B	-0.219	kinesin family member 13B
**Q9NS87**	KIF15	-0.193	kinesin family member 15
**Q96L93-2**	KIF16B	-0.494	kinesin family member 16B
**Q86Y91-4**	KIF18B	0.353	kinesin family member 18B
**O60333-4**	KIF1B	-0.126	kinesin family member 1B
**O95235**	KIF20A	0.455	kinesin family member 20A
**Q96Q89-2**	KIF20B	0.311	kinesin family member 20B
**Q14807**	KIF22	0.179	kinesin family member 22
**Q99661**	KIF2C	0.263	kinesin family member 2C
**Q9BW19**	KIFC1	0.323	kinesin family member C1
**Q9NSK0-3**	KLC4	-0.138	kinesin light chain 4
**Q9BXK1**	KLF16	-0.432	Kruppel like factor 16
**P52292**	KPNA2	0.162	karyopherin subunit alpha 2
**P83111-1**	LACTB	0.308	lactamase beta
**O15230**	LAMA5	-0.401	laminin subunit alpha 5
**P11279**	LAMP1	0.267	lysosomal associated membrane protein 1
**O43813**	LANCL1	-0.206	LanC like 1
**Q659C4**	LARP1B	-0.377	La ribonucleoprotein domain family member 1B
**P01130**	LDLR	-0.245	low density lipoprotein receptor
**O95202**	LETM1	0.204	leucine zipper and EF-hand containing transmembrane protein 1
**Q99538**	LGMN	0.288	legumain
**P18858**	LIG1	0.127	DNA ligase 1
**Q6P1M3-1**	LLGL2	-0.207	LLGL scribble cell polarity complex component 2
**Q9BRT6**	LLPH	-0.363	LLP homolog, long-term synaptic facilitation factor
**Q9BU23-1**	LMF2	0.134	lipase maturation factor 2
**Q8NF37**	LPCAT1	-0.189	lysophosphatidylcholine acyltransferase 1

**Q93052**	LPP	-0.436	LIM domain containing preferred translocation partner in lipoma
**P50851-2**	LRBA	-0.279	LPS responsive beige-like anchor protein
**Q7Z4F1**	LRP10	0.97	LDL receptor related protein 10
**P30533**	LRPAP1	0.198	LDL receptor related protein associated protein 1
**Q9H9A6**	LRRC40	-0.104	leucine rich repeat containing 40
**Q8IWT6**	LRRC8A	0.158	leucine rich repeat containing 8 VRAC subunit A
**Q9UFC0**	LRWD1	0.128	leucine rich repeats and WD repeat domain containing 1
**O15116**	LSM1	0.187	LSM1 homolog, mRNA degradation associated
**P48449**	LSS	-0.138	lanosterol synthase
**Q96GA3**	LTV1	-0.368	LTV1 ribosome biogenesis factor
**Q9Y383**	LUC7L2	0.211	LUC7 like 2, pre-mRNA splicing factor
**P61626**	LYZ	0.303	lysozyme
**Q9UPN3**	MACF1	-0.27	microtubule actin crosslinking factor 1
**Q9Y6D9**	MAD1L1	-0.286	mitotic arrest deficient 1 like 1
**Q8WXG6-4**	MADD	0.231	MAP kinase activating death domain
**Q9Y5V3-2**	MAGED1	-0.401	MAGE family member D1
**Q96QZ7**	MAGI1	-0.474	membrane associated guanylate kinase, WW and PDZ domain containing 1
**Q9UDY8**	MALT1	-0.141	MALT1 paracaspase
**P33908**	MAN1A1	-0.209	mannosidase alpha class 1A member 1
**Q9NYL2**	MAP3K20	-0.325	mitogen-activated protein kinase kinase kinase 20
**P27816-1**	MAP4	-0.322	microtubule associated protein 4
**Q15691**	MAPRE1	0.187	microtubule associated protein RP/EB family member 1
**P49006**	MARCKSL1	0.203	MARCKS like 1
**P43121**	MCAM	0.496	melanoma cell adhesion molecule
**Q8NI22**	MCFD2	-0.212	multiple coagulation factor deficiency 2
**P25205-2**	MCM3	-0.167	minichromosome maintenance complex component 3
**P33992**	MCM5	-0.196	minichromosome maintenance complex component 5
**Q14676**	MDC1	-0.228	mediator of DNA damage checkpoint 1
**P40926**	MDH2	0.179	malate dehydrogenase 2
**Q00987-11**	MDM2	0.624	MDM2 proto-oncogene
**Q9NU22**	MDN1	-0.137	midasin AAA ATPase 1
**Q9NPJ6**	MED4	0.143	mediator complex subunit 4
**Q9H8H3**	METTL7A	0.226	methyltransferase like 7A
**P26572**	MGAT1	0.137	mannosyl (alpha-1,3-)-glycoprotein beta-1,2-N-acetylglucosaminyltransferase
**Q09328**	MGAT5	-0.437	alpha-1,6-mannosylglycoprotein 6-beta-N-acetylglucosaminyltransferase
**Q9BQP7**	MGME1	0.376	mitochondrial genome maintenance exonuclease 1
**P16455**	MGMT	-0.263	O-6-methylguanine-DNA methyltransferase
**Q8N3F8**	MICALL1	0.45	MICAL like 1
**Q8IYU8**	MICU2	-0.202	mitochondrial calcium uptake 2
**Q9NPA3**	MID1IP1	-0.383	MID1 interacting protein 1
**Q9H000**	MKRN2	-0.191	makorin ring finger protein 2
**Q14165**	MLEC	0.11	malectin
**Q15773**	MLF2	0.342	myeloid leukemia factor 2
**P51511**	MMP15	-0.43	matrix metallopeptidase 15
**Q96EN8**	MOCOS	-0.145	molybdenum cofactor sulfurase
**Q15014**	MORF4L2	-0.386	mortality factor 4 like 2
**Q9Y5U8**	MPC1	0.277	mitochondrial pyruvate carrier 1

**P29372**	MPG	-0.182	N-methylpurine DNA glycosylase
**Q8N3R9-1**	MPP5	-0.207	membrane palmitoylated protein 5
**Q5T2T1**	MPP7	-0.233	membrane palmitoylated protein 7
**Q9Y605**	MRFAP1	-0.303	Morf4 family associated protein 1
**Q9NV56**	MRGBP	0.125	MRG domain binding protein
**Q9NYK5**	MRPL39	0.169	mitochondrial ribosomal protein L39
**Q86TS9**	MRPL52	0.211	mitochondrial ribosomal protein L52
**P82663**	MRPS25	0.164	mitochondrial ribosomal protein S25
**Q9ULH7-5**	MRTFB	-0.225	myocardin related transcription factor B
**O94776**	MTA2	0.189	metastasis associated 1 family member 2
**Q13496**	MTM1	-0.227	myotubularin 1
**P42345**	MTOR	-0.162	mechanistic target of rapamycin kinase
**Q99707**	MTR	-0.196	5-methyltetrahydrofolate-homocysteine methyltransferase
**Q9UBK8-1**	MTRR	-0.222	5-methyltetrahydrofolate-homocysteine methyltransferase reductase
**Q765P7**	MTSS2	-0.132	MTSS I-BAR domain containing 2
**P84157-2**	MXRA7	0.335	matrix remodeling associated 7
**Q969H8**	MYDGF	0.192	myeloid derived growth factor
**Q7Z406-2**	MYH14	-0.165	myosin heavy chain 14
**O14950**	MYL12B	-0.185	myosin light chain 12B
**Q15746**	MYLK	-0.24	myosin light chain kinase
**Q92614-1**	MYO18A	-0.212	myosin XVIIIA
**O94832**	MYO1D	-0.229	myosin ID
**Q12965**	MYO1E	-0.173	myosin IE
**Q9NQX4**	MYO5C	-0.175	myosin VC
**Q6NSJ0**	MYORG	-0.355	myogenesis regulating glycosidase (putative)
**Q14CX7**	NAA25	-0.165	N(alpha)-acetyltransferase 25, NatB auxiliary subunit
**A2RRP1**	NBAS	-0.295	NBAS subunit of NRZ tethering complex
**Q15021**	NCAPD2	-0.13	non-SMC condensin I complex subunit D2
**Q6PIU2-2**	NCEH1	0.182	neutral cholesterol ester hydrolase 1
**Q9NZQ3**	NCKIPSD	0.16	NCK interacting protein with SH3 domain
**Q68D85**	NCR3LG1	-0.347	natural killer cell cytotoxicity receptor 3 ligand 1
**Q92542-1**	NCSTN	0.156	nicastrin
**Q9UGV2**	NDRG3	-0.295	NDRG family member 3
**Q9UI09**	NDUFA12	0.14	NADH:ubiquinone oxidoreductase subunit A12
**P56556**	NDUFA6	0.216	NADH:ubiquinone oxidoreductase subunit A6
**P51970**	NDUFA8	0.394	NADH:ubiquinone oxidoreductase subunit A8
**O14561**	NDUFAB1	0.213	NADH:ubiquinone oxidoreductase subunit AB1
**P28331-2**	NDUFS1	0.104	NADH:ubiquinone oxidoreductase core subunit S1
**O43181**	NDUFS4	0.205	NADH:ubiquinone oxidoreductase subunit S4
**O43920**	NDUFS5	0.237	NADH:ubiquinone oxidoreductase subunit S5
**P46934-4**	NEDD4	-0.23	NEDD4 E3 ubiquitin protein ligase
**P48681**	NES	-0.222	nestin
**Q99519**	NEU1	0.207	neuraminidase 1
**Q8N5V2**	NGEF	-0.551	neuronal guanine nucleotide exchange factor
**Q9H9Q4-2**	NHEJ1	-0.248	non-homologous end joining factor 1
**P14543-1**	NID1	-0.374	nidogen 1
**Q9BYG3**	NIFK	-0.248	nucleolar protein interacting with the FHA domain of MKI67

**Q92982**	NINJ1	0.361	ninjurin 1
**Q969G9**	NKD1	-0.597	NKD inhibitor of WNT signaling pathway 1
**Q9ULX3**	NOB1	-0.175	NIN1 (RPN12) binding protein 1 homolog
**Q9BSC4**	NOL10	-0.132	nucleolar protein 10
**Q9UGY1**	NOL12	-0.182	nucleolar protein 12
**Q9H6R4-1**	NOL6	-0.177	nucleolar protein 6
**Q9UMY1**	NOL7	-0.248	nucleolar protein 7
**Q9Y3C1**	NOP16	-0.366	NOP16 nucleolar protein
**Q9NZM5**	NOP53	-0.407	NOP53 ribosome biogenesis factor
**P29474**	NOS3	-0.31	nitric oxide synthase 3
**P61916**	NPC2	0.264	NPC intracellular cholesterol transporter 2
**O95478**	NSA2	-0.676	NSA2 ribosome biogenesis factor
**P49902**	NT5C2	-0.177	5'-nucleotidase, cytosolic II
**Q9BSD7**	NTPCR	0.259	nucleoside-triphosphatase, cancer-related
**Q8IVD9**	NUDCD3	-0.221	NudC domain containing 3
**P50583**	NUDT2	-0.181	nudix hydrolase 2
**O43809**	NUDT21	0.173	nudix hydrolase 21
**Q7Z417**	NUFIP2	0.111	nuclear FMR1 interacting protein 2
**P49757**	NUMB	-0.133	NUMB endocytic adaptor protein
**P49790**	NUP153	0.126	nucleoporin 153
**Q8NFH4**	NUP37	0.182	nucleoporin 37
**Q7Z3B4**	NUP54	0.156	nucleoporin 54
**Q9UBU9-1**	NXF1	0.125	nuclear RNA export factor 1
**P04181**	OAT	0.323	ornithine aminotransferase
**Q9NX40**	OCIAD1	0.137	OCIA domain containing 1
**Q16625**	OCLN	-0.136	occludin
**O60502**	OGA	0.172	O-GlcNAcase
**Q8N543**	OGFOD1	0.199	2-oxoglutarate and iron dependent oxygenase domain containing 1
**O15294**	OGT	-0.144	O-linked N-acetylglucosamine (GlcNAc) transferase
**Q9H6K4**	OPA3	0.24	outer mitochondrial membrane lipid metabolism regulator OPA3
**O14841**	OPLAH	-0.146	5-oxoprolinase, ATP-hydrolysing
**Q6GQQ9-1**	OTUD7B	0.248	OTU deubiquitinase 7B
**O15460**	P4HA2	0.405	prolyl 4-hydroxylase subunit alpha 2
**Q6VY07-2**	PACS1	-0.334	phosphofurin acidic cluster sorting protein 1
**P00439**	PAH	-0.252	phenylalanine hydroxylase
**P22234-2**	PAICS	-0.124	phosphoribosylaminoimidazole carboxylase and phosphoribosylaminoimidazolesuccinocar
**O96013**	PAK4	-0.339	p21 (RAC1) activated kinase 4
**O95453**	PARN	-0.128	poly(A)-specific ribonuclease
**Q9UKK3**	PARP4	-0.172	poly(ADP-ribose) polymerase family member 4
**Q9NVD7**	PARVA	-0.248	parvin alpha
**P05166-2**	PCCB	-0.208	propionyl-CoA carboxylase subunit beta
**P12004**	PCNA	0.42	proliferating cell nuclear antigen
**Q99447**	PCYT2	-0.163	phosphate cytidylyltransferase 2, ethanolamine
**Q9GZU2**	PEG3	-0.15	paternally expressed 3
**O15067**	PFAS	-0.209	phosphoribosylformylglycinamidine synthase
**Q01813**	PFKP	-0.299	phosphofructokinase, platelet
**Q7RTV0**	PHF5A	0.22	PHD finger protein 5A

**Q6NSJ2-1**	PHLDB3	0.319	pleckstrin homology like domain family B member 3
**Q9P1Y6**	PHRF1	-0.19	PHD and ring finger domains 1
**Q13492**	PICALM	0.159	phosphatidylinositol binding clathrin assembly protein
**Q92643**	PIGK	0.143	phosphatidylinositol glycan anchor biosynthesis class K
**Q8TEQ8-1**	PIGO	-0.229	phosphatidylinositol glycan anchor biosynthesis class O
**Q9H490**	PIGU	-0.275	phosphatidylinositol glycan anchor biosynthesis class U
**Q9Y237-2**	PIN4	-0.135	peptidylprolyl cis/trans isomerase, NIMA-interacting 4
**Q96BK5**	PINX1	-0.236	PIN2 (TERF1) interacting telomerase inhibitor 1
**Q9UG56-3**	PISD	-0.114	phosphatidylserine decarboxylase
**P30613**	PKLR	-0.268	pyruvate kinase L/R
**Q16513**	PKN2	-0.215	protein kinase N2
**Q6IQ23-2**	PLEKHA7	-0.506	pleckstrin homology domain containing A7
**Q99541**	PLIN2	0.384	perilipin 2
**O43660**	PLRG1	0.109	pleiotropic regulator 1
**Q14651**	PLS1	-0.175	plastin 1
**O43157**	PLXNB1	-0.14	plexin B1
**O15031**	PLXNB2	0.24	plexin B2
**P29590**	PML	0.226	promyelocytic leukemia
**O15305**	PMM2	-0.162	phosphomannomutase 2
**Q8N490**	PNKD	-0.602	PNKD metallo-beta-lactamase domain containing
**Q9NRX1**	PNO1	-0.359	partner of NOB1 homolog
**P09884**	POLA1	-0.286	DNA polymerase alpha 1, catalytic subunit
**P06746**	POLB	0.307	DNA polymerase beta
**P28340**	POLD1	-0.163	DNA polymerase delta 1, catalytic subunit
**P49005**	POLD2	-0.184	DNA polymerase delta 2, accessory subunit
**P52435**	POLR2J	0.314	RNA polymerase II subunit J
**Q9BUI4**	POLR3C	0.118	RNA polymerase III subunit C
**O95707**	POP4	-0.112	POP4 homolog, ribonuclease P/MRP subunit
**Q9HAB8**	PPCS	0.151	phosphopantothenoylcysteine synthetase
**P45877**	PPIC	0.184	peptidylprolyl isomerase C
**P30405**	PPIF	0.177	peptidylprolyl isomerase F
**Q9H2H8-1**	PPIL3	-0.132	peptidylprolyl isomerase like 3
**P35813-3**	PPM1A	0.319	protein phosphatase, Mg2+/Mn2+ dependent 1A
**O15297**	PPM1D	0.487	protein phosphatase, Mg2+/Mn2+ dependent 1D
**Q9ULR3**	PPM1H	-0.333	protein phosphatase, Mg2+/Mn2+ dependent 1H
**Q96QC0**	PPP1R10	0.262	protein phosphatase 1 regulatory subunit 10
**Q6ZMI0-1**	PPP1R21	-0.191	protein phosphatase 1 regulatory subunit 21
**Q12972**	PPP1R8	0.148	protein phosphatase 1 regulatory subunit 8
**Q9ULJ8-3**	PPP1R9A	-0.335	protein phosphatase 1 regulatory subunit 9A
**Q6IN85**	PPP4R3A	0.152	protein phosphatase 4 regulatory subunit 3A
**O43663**	PRC1	0.347	protein regulator of cytokinesis 1
**P48147**	PREP	-0.157	prolyl endopeptidase
**Q4J6C6**	PREPL	0.266	prolyl endopeptidase like
**P49643**	PRIM2	-0.417	DNA primase subunit 2
**P31321**	PRKAR1B	-0.584	protein kinase cAMP-dependent type I regulatory subunit beta
**P17252**	PRKCA	-0.378	protein kinase C alpha
**O75569**	PRKRA	0.228	protein activator of interferon induced protein kinase EIF2AK2

**Q9H875**	PRKRIP1	0.266	PRKR interacting protein 1
**Q9UNN8**	PROCR	0.845	protein C receptor
**Q9UMS4**	PRPF19	0.126	pre-mRNA processing factor 19
**Q8NAV1-1**	PRPF38A	0.122	pre-mRNA processing factor 38A
**Q13523**	PRPF4B	0.165	pre-mRNA processing factor 4B
**P61289-2**	PSME3	0.131	proteasome activator subunit 3
**Q92530**	PSMF1	-0.217	proteasome inhibitor subunit 1
**O95456**	PSMG1	-0.151	proteasome assembly chaperone 1
**O95758-4**	PTBP3	0.176	polypyrimidine tract binding protein 3
**Q9BVG9**	PTDSS2	-0.415	phosphatidylserine synthase 2
**Q86YD1-1**	PTOV1	-0.222	PTOV1 extended AT-hook containing adaptor protein
**Q05209**	PTPN12	-0.223	protein tyrosine phosphatase non-receptor type 12
**P17706-1**	PTPN2	-0.24	protein tyrosine phosphatase non-receptor type 2
**P10586-1**	PTPRF	-0.397	protein tyrosine phosphatase receptor type F
**Q15262-3**	PTPRK	-0.531	protein tyrosine phosphatase receptor type K
**Q15397**	PUM3	-0.48	pumilio RNA binding family member 3
**Q96C36**	PYCR2	0.248	pyrroline-5-carboxylate reductase 2
**Q6WKZ4**	RAB11FIP1	-0.174	RAB11 family interacting protein 1
**P61106**	RAB14	0.131	RAB14, member RAS oncogene family
**Q9UL25**	RAB21	0.167	RAB21, member RAS oncogene family
**Q15286**	RAB35	0.144	RAB35, member RAS oncogene family
**O95716**	RAB3D	-0.181	RAB3D, member RAS oncogene family
**Q9Y3P9**	RABGAP1	-0.272	RAB GTPase activating protein 1
**P63000-2**	RAC1	0.18	Rac family small GTPase 1
**Q9H0H5**	RACGAP1	0.302	Rac GTPase activating protein 1
**P63244**	RACK1	-0.181	receptor for activated C kinase 1
**P78406**	RAE1	0.15	ribonucleic acid export 1
**P11234-2**	RALB	0.246	RAS like proto-oncogene B
**P62826**	RAN	0.104	RAN, member RAS oncogene family
**Q96S59-1**	RANBP9	0.133	RAN binding protein 9
**P52306-5**	RAP1GDS1	-0.203	Rap1 GTPase-GDP dissociation stimulator 1
**P61225**	RAP2B	0.437	RAP2B, member of RAS oncogene family
**Q7Z6E9**	RBBP6	0.438	RB binding protein 6, ubiquitin ligase
**O43251-8**	RBFOX2	-0.573	RNA binding fox-1 homolog 2
**Q8N0T1**	RBIS	-0.77	ribosomal biogenesis factor
**Q96T37**	RBM15	0.156	RNA binding motif protein 15
**Q9Y4C8**	RBM19	-0.221	RNA binding motif protein 19
**Q9NW64**	RBM22	0.214	RNA binding motif protein 22
**P49756-1**	RBM25	0.106	RNA binding motif protein 25
**Q9P2N5**	RBM27	0.142	RNA binding motif protein 27
**P42696**	RBM34	-0.24	RNA binding motif protein 34
**P52756**	RBM5	0.193	RNA binding motif protein 5
**P09455-1**	RBP1	-0.168	retinol binding protein 1
**Q8IZV5**	RDH10	0.347	retinol dehydrogenase 10
**P35241-5**	RDX	0.212	radixin
**Q9H6H4**	REEP4	0.283	receptor accessory protein 4
**Q00765**	REEP5	0.236	receptor accessory protein 5

**P78509**	RELN	-0.47	reelin
**Q6NUM9-1**	RETSAT	0.631	retinol saturase
**P35250-1**	RFC2	-0.119	replication factor C subunit 2
**Q969G6**	RFK	0.371	riboflavin kinase
**Q13671**	RIN1	0.885	Ras and Rab interactor 1
**Q9BRS2**	RIOK1	-0.287	RIO kinase 1
**Q96DB5**	RMDN1	0.163	regulator of microtubule dynamics 1
**Q9Y508**	RNF114	0.367	ring finger protein 114
**O75150**	RNF40	0.116	ring finger protein 40
**Q9Y6N7-2**	ROBO1	-0.477	roundabout guidance receptor 1
**Q9H9Y2**	RPF1	-0.524	ribosome production factor 1 homolog
**P30050**	RPL12	-0.179	ribosomal protein L12
**P18621-3**	RPL17	-0.304	ribosomal protein L17
**P84098**	RPL19	-0.351	ribosomal protein L19
**P35268**	RPL22	-0.152	ribosomal protein L22
**P62829**	RPL23	-0.364	ribosomal protein L23
**P62750**	RPL23A	-0.206	ribosomal protein L23a
**P62888**	RPL30	-0.246	ribosomal protein L30
**P62899**	RPL31	-0.168	ribosomal protein L31
**Q9Y3U8**	RPL36	-0.249	ribosomal protein L36
**P46777**	RPL5	-0.14	ribosomal protein L5
**Q6DKI1**	RPL7L1	-0.376	ribosomal protein L7 like 1
**P05388**	RPLP0	-0.169	ribosomal protein lateral stalk subunit P0
**P46783**	RPS10	-0.166	ribosomal protein S10
**P62263**	RPS14	-0.222	ribosomal protein S14
**P08708**	RPS17	-0.124	ribosomal protein S17
**P39019**	RPS19	-0.105	ribosomal protein S19
**P15880**	RPS2	-0.285	ribosomal protein S2
**P60866**	RPS20	-0.17	ribosomal protein S20
**P63220**	RPS21	-0.252	ribosomal protein S21
**P62854**	RPS26	-0.189	ribosomal protein S26
**P42677**	RPS27	-0.285	ribosomal protein S27
**P23396**	RPS3	-0.183	ribosomal protein S3
**P46782**	RPS5	-0.185	ribosomal protein S5
**Q15418-2**	RPS6KA1	0.201	ribosomal protein S6 kinase A1
**P51812**	RPS6KA3	-0.211	ribosomal protein S6 kinase A3
**O75676**	RPS6KA4	-0.239	ribosomal protein S6 kinase A4
**Q8N122**	RPTOR	-0.426	regulatory associated protein of MTOR complex 1
**P55042**	RRAD	1.701	RRAD, Ras related glycolysis inhibitor and calcium channel regulator
**Q7L523**	RRAGA	0.31	Ras related GTP binding A
**Q92766-2**	RREB1	-0.275	ras responsive element binding protein 1
**P23921**	RRM1	0.164	ribonucleotide reductase catalytic subunit M1
**P31350-2**	RRM2	0.44	ribonucleotide reductase regulatory subunit M2
**Q7LG56**	RRM2B	0.666	ribonucleotide reductase regulatory TP53 inducible subunit M2B
**Q7LG56-6**	RRM2B	0.922	ribonucleotide reductase regulatory TP53 inducible subunit M2B
**Q96EU6**	RRP36	-0.376	ribosomal RNA processing 36
**Q15050**	RRS1	-0.313	ribosome biogenesis regulator 1 homolog

**Q9UHA3**	RSL24D1	-0.317	ribosomal L24 domain containing 1
**Q15404**	RSU1	-0.146	Ras suppressor protein 1
**P19793**	RXRA	-0.57	retinoid X receptor alpha
**P28702-3**	RXRB	-0.224	retinoid X receptor beta
**P31949**	S100A11	0.346	S100 calcium binding protein A11
**Q99584**	S100A13	0.216	S100 calcium binding protein A13
**Q96FQ6**	S100A16	0.195	S100 calcium binding protein A16
**P06703**	S100A6	0.308	S100 calcium binding protein A6
**P25815**	S100P	0.156	S100 calcium binding protein P
**P35542**	SAA4	0.741	serum amyloid A4, constitutive
**O00422**	SAP18	0.14	Sin3A associated protein 18
**Q9Y6B6**	SAR1B	-0.14	secretion associated Ras related GTPase 1B
**P82979**	SARNP	0.212	SAP domain containing ribonucleoprotein
**O00767**	SCD	-0.357	stearoyl-CoA desaturase
**Q8WU76-1**	SCFD2	-0.402	sec1 family domain containing 2
**P18827**	SDC1	0.356	syndecan 1
**P34741**	SDC2	-0.521	syndecan 2
**P31431-1**	SDC4	0.501	syndecan 4
**O00560**	SDCBP	0.262	syndecan binding protein
**Q99470**	SDF2	0.238	stromal cell derived factor 2
**P31040**	SDHA	0.228	succinate dehydrogenase complex flavoprotein subunit A
**Q15437**	SEC23B	-0.186	SEC23 homolog B, coat complex II component
**O95486**	SEC24A	-0.176	SEC24 homolog A, COPII coat complex component
**O94855-2**	SEC24D	-0.188	SEC24 homolog D, COPII coat complex component
**Q16181**	SEPTIN7	0.123	septin 7
**Q9UHD8-1**	SEPTIN9	-0.179	septin 9
**Q8NC51-1**	SERBP1	-0.154	SERPINE1 mRNA binding protein 1
**P05543**	SERPINA7	0.511	serpin family A member 7
**P30740**	SERPINB1	0.155	serpin family B member 1
**P05121**	SERPINE1	1.153	serpin family E member 1
**P58004**	SESN2	0.594	sestrin 2
**Q15428**	SF3A2	0.218	splicing factor 3a subunit 2
**Q12874**	SF3A3	0.184	splicing factor 3a subunit 3
**Q9BWJ5**	SF3B5	0.125	splicing factor 3b subunit 5
**P31947**	SFN	0.415	stratifin
**P51688**	SGSH	0.176	N-sulfoglucosamine sulfohydrolase
**Q9H299**	SH3BGRL3	0.238	SH3 domain binding glutamate rich protein like 3
**Q8NEM2**	SHCBP1	0.63	SHC binding and spindle associated 1
**A0A0B4J2A0**	SHPK	-0.117	sedoheptulokinase
**Q9UP95**	SLC12A4	0.359	solute carrier family 12 member 4
**P53985**	SLC16A1	0.153	solute carrier family 16 member 1
**P43007**	SLC1A4	-0.248	solute carrier family 1 member 4
**Q8WUM9**	SLC20A1	-0.253	solute carrier family 20 member 1
**Q9Y619**	SLC25A15	0.308	solute carrier family 25 member 15
**O14975**	SLC27A2	-0.123	solute carrier family 27 member 2
**P11166**	SLC2A1	-0.303	solute carrier family 2 member 1
**P14672**	SLC2A4	-0.34	solute carrier family 2 member 4

**Q8NEW0**	SLC30A7	-0.204	solute carrier family 30 member 7
**Q9HBR0**	SLC38A10	-0.158	solute carrier family 38 member 10
**Q99624**	SLC38A3	-0.172	solute carrier family 38 member 3
**Q15043**	SLC39A14	-0.393	solute carrier family 39 member 14
**Q8WWI5**	SLC44A1	-0.266	solute carrier family 44 member 1
**Q9BWU0**	SLC4A1AP	0.232	solute carrier family 4 member 1 adaptor protein
**P04920**	SLC4A2	-0.133	solute carrier family 4 member 2
**Q9UPY5**	SLC7A11	-0.312	solute carrier family 7 member 11
**Q15599**	SLC9A3R2	-0.276	SLC9A3 regulator 2
**Q14BN4**	SLMAP	-0.17	sarcolemma associated protein
**Q92925**	SMARCD2	-0.257	SWI/SNF related, matrix associated, actin dependent regulator of chromatin, subfamily d,
**Q86US8-1**	SMG6	-0.47	SMG6 nonsense mediated mRNA decay factor
**P53814-6**	SMTN	0.953	smoothelin
**Q2TAY7**	SMU1	0.106	SMU1 DNA replication regulator and spliceosomal factor
**Q7KZF4**	SND1	-0.248	staphylococcal nuclease and tudor domain containing 1
**Q8WVK2**	SNRNP27	0.364	small nuclear ribonucleoprotein U4/U6.U5 subunit 27
**Q13884**	SNTB1	-0.138	syntrophin beta 1
**P55769**	SNU13	-0.193	small nuclear ribonucleoprotein 13
**Q9NRS6**	SNX15	-0.286	sorting nexin 15
**Q15036-1**	SNX17	-0.28	sorting nexin 17
**Q5VWJ9**	SNX30	-0.235	sorting nexin family member 30
**Q9Y5X2**	SNX8	-0.261	sorting nexin 8
**P04179**	SOD2	0.346	superoxide dismutase 2
**Q92673**	SORL1	-0.495	sortilin related receptor 1
**Q99523**	SORT1	-0.164	sortilin 1
**Q96R06**	SPAG5	0.303	sperm associated antigen 5
**O75391**	SPAG7	-0.303	sperm associated antigen 7
**Q96EA4**	SPDL1	0.48	spindle apparatus coiled-coil protein 1
**P10451**	SPP1	-0.514	secreted phosphoprotein 1
**P35270**	SPR	0.163	sepiapterin reductase
**Q5W111**	SPRYD7	0.213	SPRY domain containing 7
**Q9NUV7**	SPTLC3	-0.622	serine palmitoyltransferase long chain base subunit 3
**Q14534**	SQLE	-0.31	squalene epoxidase
**Q8NEF9**	SRFBP1	-0.212	serum response factor binding protein 1
**O75044**	SRGAP2	-0.217	SLIT-ROBO Rho GTPase activating protein 2
**Q96SB4**	SRPK1	-0.147	SRSF protein kinase 1
**Q9BXP5**	SRRT	0.23	serrate, RNA effector molecule
**Q16629**	SRSF7	0.199	serine and arginine rich splicing factor 7
**Q08945**	SSRP1	0.148	structure specific recognition protein 1
**Q9NP77**	SSU72	-0.143	SSU72 homolog, RNA polymerase II CTD phosphatase
**P15907**	ST6GAL1	-0.345	ST6 beta-galactoside alpha-2,6-sialyltransferase 1
**P42224-1**	STAT1	0.108	signal transducer and activator of transcription 1
**P40763**	STAT3	0.433	signal transducer and activator of transcription 3
**Q9NUL3**	STAU2	-0.322	staufen double-stranded RNA binding protein 2
**Q658P3-2**	STEAP3	-0.519	STEAP3 metalloreductase
**Q13586**	STIM1	-0.22	stromal interaction molecule 1
**O94804**	STK10	-0.33	serine/threonine kinase 10

**Q13188-2**	STK3	-0.812	serine/threonine kinase 3
**P27105**	STOM	0.706	stomatin
**Q86Y82**	STX12	0.21	syntaxin 12
**Q13277**	STX3	0.161	syntaxin 3
**Q12846**	STX4	0.109	syntaxin 4
**O43752**	STX6	0.437	syntaxin 6
**O00186**	STXBP3	0.214	syntaxin binding protein 3
**P53999**	SUB1	0.374	SUB1 regulator of transcription
**O75683**	SURF6	-0.542	surfeit 6
**Q9UH65**	SWAP70	0.146	switching B cell complex subunit SWAP70
**O95359**	TACC2	-0.203	transforming acidic coiled-coil containing protein 2
**Q9Y6A5**	TACC3	0.235	transforming acidic coiled-coil containing protein 3
**P37837**	TALDO1	0.245	transaldolase 1
**Q03518**	TAP1	0.462	transporter 1, ATP binding cassette subfamily B member
**Q86VP1**	TAX1BP1	-0.25	Tax1 binding protein 1
**O14907**	TAX1BP3	0.339	Tax1 binding protein 3
**O60343-1**	TBC1D4	-0.174	TBC1 domain family member 4
**Q92609-2**	TBC1D5	-0.347	TBC1 domain family member 5
**Q9Y4C2-1**	TCAF1	-0.182	TRPM8 channel associated factor 1
**Q9BQ70**	TCF25	-0.24	transcription factor 25
**Q9NYB0**	TERF2IP	0.371	TERF2 interacting protein
**Q96BS2**	TESC	-0.326	tescalcin
**Q8IWB9-2**	TEX2	-0.229	testis expressed 2
**P02787**	TF	-0.328	transferrin
**P01137**	TGFB1	0.312	transforming growth factor beta 1
**P37173-2**	TGFBR2	-0.361	transforming growth factor beta receptor 2
**Q03167**	TGFBR3	-0.276	transforming growth factor beta receptor 3
**P21980**	TGM2	-0.16	transglutaminase 2
**Q96EK4**	THAP11	-0.181	THAP domain containing 11
**P07996**	THBS1	-0.351	thrombospondin 1
**Q9NXG2**	THUMPD1	0.267	THUMP domain containing 1
**Q9BV44**	THUMPD3	-0.141	THUMP domain containing 3
**Q9NQ88**	TIGAR	0.756	TP53 induced glycolysis regulatory phosphatase
**Q9Y5J6**	TIMM10B	0.116	translocase of inner mitochondrial membrane 10B
**Q9UDY2**	TJP2	-0.176	tight junction protein 2
**P04183**	TK1	0.494	thymidine kinase 1
**Q9Y3A6**	TMED5	0.257	transmembrane p24 trafficking protein 5
**Q9P0S9**	TMEM14C	0.187	transmembrane protein 14C
**Q9HC07**	TMEM165	0.118	transmembrane protein 165
**Q8N511**	TMEM199	0.23	transmembrane protein 199
**Q9H1E5**	TMX4	0.304	thioredoxin related transmembrane protein 4
**O00220**	TNFRSF10A	0.294	TNF receptor superfamily member 10a
**Q68CZ2-1**	TNS3	-0.335	tensin 3
**Q96GM8**	TOE1	0.122	target of EGR1, exonuclease
**O60784**	TOM1	-0.213	target of myb1 membrane trafficking protein
**Q15785**	TOMM34	0.11	translocase of outer mitochondrial membrane 34
**Q8N4H5**	TOMM5	0.21	translocase of outer mitochondrial membrane 5

**O14656**	TOR1A	0.212	torsin family 1 member A
**O94842**	TOX4	0.232	TOX high mobility group box family member 4
**P04637**	TP53	1.13	tumor protein p53
**Q53FA7**	TP53I3	1.179	tumor protein p53 inducible protein 3
**Q9ULW0**	TPX2	0.647	TPX2 microtubule nucleation factor
**Q9Y296**	TRAPPC4	0.16	trafficking protein particle complex 4
**O15164**	TRIM24	-0.198	tripartite motif containing 24
**Q14258**	TRIM25	-0.195	tripartite motif containing 25
**Q13263**	TRIM28	0.202	tripartite motif containing 28
**Q15643**	TRIP11	0.153	thyroid hormone receptor interactor 11
**Q15650**	TRIP4	-0.405	thyroid hormone receptor interactor 4
**Q8TEL6**	TRPC4AP	-0.233	transient receptor potential cation channel subfamily C member 4 associated protein
**Q9BSV6**	TSEN34	-0.219	tRNA splicing endonuclease subunit 34
**Q9H2G4**	TSPYL2	0.331	TSPY like 2
**Q2NL82**	TSR1	-0.117	TSR1 ribosome maturation factor
**Q16762**	TST	0.266	thiosulfate sulfurtransferase
**Q6P3X3**	TTC27	-0.298	tetratricopeptide repeat domain 27
**Q13509**	TUBB3	0.166	tubulin beta 3 class III
**Q9BUF5**	TUBB6	0.398	tubulin beta 6 class V
**Q96CW5**	TUBGCP3	-0.157	tubulin gamma complex associated protein 3
**Q5TAX3**	TUT4	-0.246	terminal uridylyl transferase 4
**Q6IBS0**	TWF2	0.526	twinfilin actin binding protein 2
**P26368**	U2AF2	0.136	U2 small nuclear RNA auxiliary factor 2
**Q9BSL1**	UBAC1	-0.335	UBA domain containing 1
**P62256**	UBE2H	0.144	ubiquitin conjugating enzyme E2 H
**O95155-4**	UBE4B	-0.209	ubiquitination factor E4B
**O95071**	UBR5	0.114	ubiquitin protein ligase E3 component n-recognin 5
**Q04323-1**	UBXN1	-0.164	UBX domain protein 1
**Q9BZX2-1**	UCK2	-0.208	uridine-cytidine kinase 2
**P16662**	UGT2B7	0.494	UDP glucuronosyltransferase family 2 member B7
**Q96T88-2**	UHRF1	-0.634	ubiquitin like with PHD and ring finger domains 1
**Q70J99-3**	UNC13D	-0.203	unc-13 homolog D
**P13051**	UNG	-0.259	uracil DNA glycosylase
**Q9BZI7-1**	UPF3B	0.196	UPF3B regulator of nonsense mediated mRNA decay
**P51784**	USP11	0.227	ubiquitin specific peptidase 11
**Q92995**	USP13	-0.23	ubiquitin specific peptidase 13
**Q8NFA0**	USP32	-0.323	ubiquitin specific peptidase 32
**Q9P275-2**	USP36	-0.11	ubiquitin specific peptidase 36
**Q93008**	USP9X	-0.144	ubiquitin specific peptidase 9 X-linked
**Q9BVJ6-1**	UTP14A	-0.25	UTP14A small subunit processome component
**Q9Y5J1**	UTP18	-0.105	UTP18 small subunit processome component
**P46939**	UTRN	-0.259	utrophin
**Q9BV40**	VAMP8	0.32	vesicle associated membrane protein 8
**Q6EMK4**	VASN	-0.329	vasorin
**P50552**	VASP	0.126	vasodilator stimulated phosphoprotein
**P52735**	VAV2	-0.331	vav guanine nucleotide exchange factor 2
**P18206**	VCL	-0.121	vinculin

**P21796**	VDAC1	0.163	voltage dependent anion channel 1
**P45880-1**	VDAC2	0.298	voltage dependent anion channel 2
**Q9Y277-2**	VDAC3	0.256	voltage dependent anion channel 3
**Q9H9C1-1**	VIPAS39	-0.218	VPS33B interacting protein, apical-basolateral polarity regulator, spe-39 homolog
**Q69YN4**	VIRMA	0.168	vir like m6A methyltransferase associated
**Q96GC9**	VMP1	-0.139	vacuole membrane protein 1
**Q96JG6**	VPS50	-0.222	VPS50 subunit of EARP/GARPII complex
**Q9UID3-1**	VPS51	-0.191	VPS51 subunit of GARP complex
**Q5VIR6-4**	VPS53	-0.355	VPS53 subunit of GARP complex
**P62760**	VSNL1	-0.24	visinin like 1
**Q96AJ9**	VTI1A	-0.273	vesicle transport through interaction with t-SNAREs 1A
**Q9Y6W5**	WASF2	0.115	WASP family member 2
**Q12768**	WASHC5	-0.172	WASH complex subunit 5
**O00401**	WASL	-0.132	WASP like actin nucleation promoting factor
**Q9H6R7**	WDCP	-0.21	WD repeat and coiled coil containing
**P61964**	WDR5	0.167	WD repeat domain 5
**Q6RFH5**	WDR74	-0.232	WD repeat domain 74
**O00308**	WWP2	-0.697	WW domain containing E3 ubiquitin protein ligase 2
**Q9C0E2**	XPO4	-0.281	exportin 4
**O75191**	XYLB	-0.18	xylulokinase
**P54577**	YARS1	-0.147	tyrosyl-tRNA synthetase 1
**P07947**	YES1	0.108	YES proto-oncogene 1, Src family tyrosine kinase
**O15498**	YKT6	0.289	YKT6 v-SNARE homolog
**Q9H6S0**	YTHDC2	-0.233	YTH domain containing 2
**Q04917**	YWHAH	0.124	tyrosine 3-monooxygenase/tryptophan 5-monooxygenase activation protein eta
**Q8N4Q0**	ZADH2	0.24	zinc binding alcohol dehydrogenase domain containing 2
**Q8WU90**	ZC3H15	-0.307	zinc finger CCCH-type containing 15
**Q7Z2W4**	ZC3HAV1	0.148	zinc finger CCCH-type containing, antiviral 1
**Q9C0B9**	ZCCHC2	-0.371	zinc finger CCHC-type containing 2
**Q8N5A5**	ZGPAT	0.193	zinc finger CCCH-type and G-patch domain containing
**Q9Y6X8**	ZHX2	-0.367	zinc fingers and homeoboxes 2
**Q14202**	ZMYM3	-0.213	zinc finger MYM-type containing 3
**Q5BKZ1**	ZNF326	0.272	zinc finger protein 326
**Q86UK7**	ZNF598	-0.197	zinc finger protein 598
**Q9H582**	ZNF644	-0.313	zinc finger protein 644
**Q9P2E3**	ZNFX1	0.267	zinc finger NFX1-type containing 1
**O43149**	ZZEF1	-0.265	zinc finger ZZ-type and EF-hand domain containing 1
**Q16637**		0.342	
**Q96JG8-4**		-0.216	

List of proteins deregulated in HepG2 cells after 24h exposure to AgNP 3.5 ug/mL + CDDP 10 mM

ID	Symbol	Expr Log Ratio	Entrez Gene Name
**Q9NQ94**	A1CF	-0.214	APOBEC1 complementation factor
**P01023**	A2M	-0.4	alpha-2-macroglobulin
**Q86V21-1**	AACS	-0.181	acetoacetyl-CoA synthetase
**Q9H7C9-1**	AAMDC	-0.387	adipogenesis associated Mth938 domain containing
**Q9Y312**	AAR2	0.301	AAR2 splicing factor
**P49588**	AARS1	-0.2	alanyl-tRNA synthetase 1
**Q5JTZ9**	AARS2	0.233	alanyl-tRNA synthetase 2, mitochondrial
**Q9NRN7**	AASDHPPT	0.161	aminoadipate-semialdehyde dehydrogenase-phosphopantetheinyl transferase
**Q9UDR5**	AASS	0.301	aminoadipate-semialdehyde synthase
**Q9NY61**	AATF	0.184	apoptosis antagonizing transcription factor
**P80404**	ABAT	0.207	4-aminobutyrate aminotransferase
**O95477**	ABCA1	-0.363	ATP binding cassette subfamily A member 1
**Q9NRK6**	ABCB10	0.317	ATP binding cassette subfamily B member 10
**O75027-2**	ABCB7	0.275	ATP binding cassette subfamily B member 7
**Q9NUT2-1**	ABCB8	0.19	ATP binding cassette subfamily B member 8
**P33897**	ABCD1	0.239	ATP binding cassette subfamily D member 1
**P28288**	ABCD3	0.309	ATP binding cassette subfamily D member 3
**P61221**	ABCE1	-0.079	ATP binding cassette subfamily E member 1
**Q9NUQ8**	ABCF3	0.134	ATP binding cassette subfamily F member 3
**Q8NFV4**	ABHD11	0.172	abhydrolase domain containing 11
**Q8N2K0-2**	ABHD12	0.119	abhydrolase domain containing 12
**Q96IU4**	ABHD14B	-0.259	abhydrolase domain containing 14B
**Q8TB40**	ABHD4	1.016	abhydrolase domain containing 4
**Q8IZP0**	ABI1	-0.254	abl interactor 1
**Q9ULW3**	ABT1	-0.113	activator of basal transcription 1
**P09110-1**	ACAA1	-0.118	acetyl-CoA acyltransferase 1
**P42765**	ACAA2	0.309	acetyl-CoA acyltransferase 2
**Q13085-4**	ACACA	-0.233	acetyl-CoA carboxylase alpha
**Q9UKU7**	ACAD8	0.549	acyl-CoA dehydrogenase family member 8
**P11310-2**	ACADM	0.231	acyl-CoA dehydrogenase medium chain
**P16219**	ACADS	0.345	acyl-CoA dehydrogenase short chain
**P45954**	ACADSB	0.151	acyl-CoA dehydrogenase short/branched chain
**P49748-3**	ACADVL	0.363	acyl-CoA dehydrogenase very long chain
**Q15057**	ACAP2	-0.186	ArfGAP with coiled-coil, ankyrin repeat and PH domains 2
**P24752**	ACAT1	0.338	acetyl-CoA acetyltransferase 1
**Q9BWD1**	ACAT2	-0.355	acetyl-CoA acetyltransferase 2
**Q5T8D3-3**	ACBD5	0.268	acyl-CoA binding domain containing 5
**Q9UKV3-5**	ACIN1	0.384	apoptotic chromatin condensation inducer 1
**P21399**	ACO1	-0.17	aconitase 1
**Q99798**	ACO2	0.282	aconitase 2
**Q86TX2**	ACOT1	0.243	acyl-CoA thioesterase 1

**Q9NPJ3**	ACOT13	0.195	acyl-CoA thioesterase 13
**O00154**	ACOT7	-0.185	acyl-CoA thioesterase 7
**O14734**	ACOT8	0.378	acyl-CoA thioesterase 8
**Q9Y305**	ACOT9	0.259	acyl-CoA thioesterase 9
**Q15067**	ACOX1	0.212	acyl-CoA oxidase 1
**Q99424**	ACOX2	0.187	acyl-CoA oxidase 2
**O15254**	ACOX3	0.175	acyl-CoA oxidase 3, pristanoyl
**P24666**	ACP1	-0.218	acid phosphatase 1
**P11117**	ACP2	0.096	acid phosphatase 2, lysosomal
**Q96CM8-2**	ACSF2	0.268	acyl-CoA synthetase family member 2
**Q4G176**	ACSF3	0.234	acyl-CoA synthetase family member 3
**O60488-1**	ACSL4	-0.573	acyl-CoA synthetase long chain family member 4
**Q9ULC5-3**	ACSL5	0.241	acyl-CoA synthetase long chain family member 5
**Q68CK6**	ACSM2B	0.334	acyl-CoA synthetase medium chain family member 2B
**Q9NUB1**	ACSS1	0.28	acyl-CoA synthetase short chain family member 1
**Q9NR19-2**	ACSS2	-0.257	acyl-CoA synthetase short chain family member 2
**Q9H6R3**	ACSS3	0.346	acyl-CoA synthetase short chain family member 3
**P12814-3**	ACTN1	-0.216	actinin alpha 1
**O43707**	ACTN4	-0.267	actinin alpha 4
**P61163**	ACTR1A	-0.134	actin related protein 1A
**P42025**	ACTR1B	-0.456	actin related protein 1B
**P61160-1**	ACTR2	-0.106	actin related protein 2
**Q03154-1**	ACY1	-0.322	aminoacylase 1
**P14621**	ACYP2	-0.486	acylphosphatase 2
**O14672**	ADAM10	-0.26	ADAM metallopeptidase domain 10
**Q13443**	ADAM9	-0.313	ADAM metallopeptidase domain 9
**P55265-4**	ADAR	0.121	adenosine deaminase RNA specific
**O60503**	ADCY9	-0.79	adenylate cyclase 9
**P35611-3**	ADD1	-0.331	adducin 1
**Q9UEY8-2**	ADD3	-0.318	adducin 3
**Q9Y653**	ADGRG1	0.597	adhesion G protein-coupled receptor G1
**Q86SQ4-3**	ADGRG6	-0.405	adhesion G protein-coupled receptor G6
**P08319-2**	ADH4	-0.285	alcohol dehydrogenase 4 (class II), pi polypeptide
**P11766**	ADH5	-0.251	alcohol dehydrogenase 5 (class III), chi polypeptide
**P28332-2**	ADH6	-0.312	alcohol dehydrogenase 6 (class V)
**Q9BV57**	ADI1	-0.268	acireductone dioxygenase 1
**P55263**	ADK	-0.396	adenosine kinase
**Q9H2P0**	ADNP	0.29	activity dependent neuroprotector homeobox
**Q9BRR6-1**	ADPGK	0.158	ADP dependent glucokinase
**Q16186**	ADRM1	0.27	adhesion regulating molecule 1
**P30566**	ADSL	-0.348	adenylosuccinate lyase
**P30520**	ADSS2	-0.185	adenylosuccinate synthase 2
**P55196-5**	AFDN	-0.121	afadin, adherens junction formation factor
**Q9UHB7-1**	AFF4	0.467	AF4/FMR2 family member 4
**Q9Y4W6**	AFG3L2	0.323	AFG3 like matrix AAA peptidase subunit 2
**P02771**	AFP	-0.343	alpha fetoprotein
**Q6ULP2**	AFTPH	-0.29	aftiphilin

**P52594-4**	AGFG1	-0.167	ArfGAP with FG repeats 1
**O95081**	AGFG2	-0.275	ArfGAP with FG repeats 2
**Q9BSE5**	AGMAT	0.286	agmatinase
**Q9NUQ2**	AGPAT5	0.146	1-acylglycerol-3-phosphate O-acyltransferase 5
**O95994**	AGR2	0.397	anterior gradient 2, protein disulphide isomerase family member
**O00468**	AGRN	-0.298	agrin
**P01019**	AGT	-0.657	angiotensinogen
**Q8WYP5-2**	AHCTF1	0.382	AT-hook containing transcription factor 1
**P23526-1**	AHCY	-0.321	adenosylhomocysteinase
**O43865**	AHCYL1	-0.12	adenosylhomocysteinase like 1
**Q09666-1**	AHNAK	-0.23	AHNAK nucleoprotein
**P02765**	AHSG	-0.474	alpha 2-HS glycoprotein
**O95831-1**	AIFM1	0.17	apoptosis inducing factor mitochondria associated 1
**Q9BRQ8**	AIFM2	0.132	apoptosis inducing factor mitochondria associated 2
**Q96IF1**	AJUBA	-0.117	ajuba LIM protein
**P00568**	AK1	-0.186	adenylate kinase 1
**Q9UIJ7-1**	AK3	0.28	adenylate kinase 3
**P27144**	AK4	0.198	adenylate kinase 4
**Q92667**	AKAP1	0.146	A-kinase anchoring protein 1
**Q12802-2**	AKAP13	-0.365	A-kinase anchoring protein 13
**Q02040-1**	AKAP17A	0.328	A-kinase anchoring protein 17A
**O43823**	AKAP8	0.296	A-kinase anchoring protein 8
**Q9ULX6**	AKAP8L	0.301	A-kinase anchoring protein 8 like
**P14550**	AKR1A1	-0.203	aldo-keto reductase family 1 member A1
**O60218**	AKR1B10	0.192	aldo-keto reductase family 1 member B10
**P52895**	AKR1C1/AKR1C2	0.172	aldo-keto reductase family 1 member C2
**Q04828**	AKR1C1/AKR1C2	0.156	aldo-keto reductase family 1 member C2
**P51857**	AKR1D1	-0.171	aldo-keto reductase family 1 member D1
**O43488**	AKR7A2	-0.204	aldo-keto reductase family 7 member A2
**P31749**	AKT1	-0.15	AKT serine/threonine kinase 1
**P13716-2**	ALAD	-0.384	aminolevulinate dehydratase
**P13196**	ALAS1	0.65	5'-aminolevulinate synthase 1
**P02768**	ALB	-0.497	albumin
**P02769**	ALB	1.177	albumin
**Q8IZ83**	ALDH16A1	-0.229	aldehyde dehydrogenase 16 family member A1
**P00352**	ALDH1A1	-0.388	aldehyde dehydrogenase 1 family member A1
**P30837**	ALDH1B1	0.318	aldehyde dehydrogenase 1 family member B1
**P05091**	ALDH2	0.318	aldehyde dehydrogenase 2 family member
**P51648-2**	ALDH3A2	0.118	aldehyde dehydrogenase 3 family member A2
**P30038**	ALDH4A1	0.266	aldehyde dehydrogenase 4 family member A1
**P51649-2**	ALDH5A1	0.352	aldehyde dehydrogenase 5 family member A1
**Q02252**	ALDH6A1	0.144	aldehyde dehydrogenase 6 family member A1
**P49419-2**	ALDH7A1	0.131	aldehyde dehydrogenase 7 family member A1
**P09972**	ALDOC	-0.238	aldolase, fructose-bisphosphate C
**Q9H553**	ALG2	0.229	ALG2 alpha-1,3/1,6-mannosyltransferase
**Q9Y673**	ALG5	0.334	ALG5 dolichyl-phosphate beta-glucosyltransferase
**Q9H6U8-3**	ALG9	0.243	ALG9 alpha-1,2-mannosyltransferase

**Q9BT30**	ALKBH7	-0.354	alkB homolog 7
**Q86V81**	ALYREF	0.178	Aly/REF export factor
**Q9UHK6-5**	AMACR	0.283	alpha-methylacyl-CoA racemase
**Q9Y2J4-4**	AMOTL2	0.682	angiomotin like 2
**Q01433-1**	AMPD2	-0.268	adenosine monophosphate deaminase 2
**P48728**	AMT	0.298	aminomethyltransferase
**Q9H1A4**	ANAPC1	-0.161	anaphase promoting complex subunit 1
**Q9UJX6**	ANAPC2	-0.201	anaphase promoting complex subunit 2
**Q9P2R3-4**	ANKFY1	-0.254	ankyrin repeat and FYVE domain containing 1
**Q8IWZ3-6**	ANKHD1/ANKHD1-EIF4EBP3	-0.205	ankyrin repeat and KH domain containing 1
**Q15327**	ANKRD1	0.826	ankyrin repeat domain 1
**O75179**	ANKRD17	-0.114	ankyrin repeat domain 17
**Q8NB46**	ANKRD52	-0.493	ankyrin repeat domain 52
**Q92625**	ANKS1A	-0.239	ankyrin repeat and sterile alpha motif domain containing 1A
**Q9NQW6**	ANLN	0.411	anillin actin binding protein
**P39687**	ANP32A	-0.178	acidic nuclear phosphoprotein 32 family member A
**Q92688**	ANP32B	-0.307	acidic nuclear phosphoprotein 32 family member B
**Q9BTT0**	ANP32E	-0.162	acidic nuclear phosphoprotein 32 family member E
**P15144**	ANPEP	-0.124	alanyl aminopeptidase, membrane
**P50995**	ANXA11	-0.17	annexin A11
**P09525**	ANXA4	-0.216	annexin A4
**P08758**	ANXA5	-0.194	annexin A5
**P08133**	ANXA6	-0.344	annexin A6
**P20073**	ANXA7	-0.167	annexin A7
**Q10567**	AP1B1	-0.129	adaptor related protein complex 1 subunit beta 1
**O43747**	AP1G1	-0.317	adaptor related protein complex 1 subunit gamma 1
**Q9BXS5-1**	AP1M1	-0.079	adaptor related protein complex 1 subunit mu 1
**P61966**	AP1S1	-0.167	adaptor related protein complex 1 subunit sigma 1
**O94973-2**	AP2A2	-0.135	adaptor related protein complex 2 subunit alpha 2
**P63010-2**	AP2B1	-0.256	adaptor related protein complex 2 subunit beta 1
**Q96CW1**	AP2M1	0.111	adaptor related protein complex 2 subunit mu 1
**O00203**	AP3B1	-0.161	adaptor related protein complex 3 subunit beta 1
**O14617-5**	AP3D1	-0.282	adaptor related protein complex 3 subunit delta 1
**P13798**	APEH	-0.329	acylaminoacyl-peptide hydrolase
**P27695**	APEX1	-0.231	apurinic/apyrimidinic endodeoxyribonuclease 1
**Q96GX9-1**	APIP	-0.23	APAF1 interacting protein
**Q06481**	APLP2	-0.208	amyloid beta precursor like protein 2
**Q9HDC9**	APMAP	0.229	adipocyte plasma membrane associated protein
**P02647**	APOA1	-0.697	apolipoprotein A1
**P02652**	APOA2	-0.337	apolipoprotein A2
**P04114**	APOB	-0.946	apolipoprotein B
**Q9UH17**	APOBEC3B	0.479	apolipoprotein B mRNA editing enzyme catalytic subunit 3B
**Q9NRW3**	APOBEC3C	0.535	apolipoprotein B mRNA editing enzyme catalytic subunit 3C
**P02655**	APOC2	-0.619	apolipoprotein C2
**P02649**	APOE	-0.35	apolipoprotein E
**O14791-2**	APOL1	-0.572	apolipoprotein L1
**Q9BUR5-1**	APOO	0.193	apolipoprotein O

**Q6UXV4**	APOOL	0.296	apolipoprotein O like
**P05067**	APP	-0.7	amyloid beta precursor protein
**P07741**	APRT	-0.276	adenine phosphoribosyltransferase
**Q7Z2E3-1**	APTX	0.171	aprataxin
**O60306**	AQR	0.215	aquarius intron-binding spliceosomal factor
**Q96P48-6**	ARAP1	-0.228	ArfGAP with RhoGAP domain, ankyrin repeat and PH domain 1
**P48444**	ARCN1	-0.116	archain 1
**P18085**	ARF4	-0.134	ADP ribosylation factor 4
**Q8N6T3-1**	ARFGAP1	-0.151	ADP ribosylation factor GTPase activating protein 1
**Q8N6H7**	ARFGAP2	-0.117	ADP ribosylation factor GTPase activating protein 2
**Q9NP61**	ARFGAP3	-0.216	ADP ribosylation factor GTPase activating protein 3
**Q9Y6D6**	ARFGEF1	-0.107	ADP ribosylation factor guanine nucleotide exchange factor 1
**Q9Y6D5**	ARFGEF2	-0.245	ADP ribosylation factor guanine nucleotide exchange factor 2
**P53365**	ARFIP2	-0.216	ADP ribosylation factor interacting protein 2
**P78540**	ARG2	0.3	arginase 2
**Q07960**	ARHGAP1	-0.172	Rho GTPase activating protein 1
**Q8IWW6-1**	ARHGAP12	-0.174	Rho GTPase activating protein 12
**Q68EM7**	ARHGAP17	-0.186	Rho GTPase activating protein 17
**Q8N392**	ARHGAP18	-0.486	Rho GTPase activating protein 18
**Q9NRY4**	ARHGAP35	-0.284	Rho GTPase activating protein 35
**P52565**	ARHGDIA	-0.209	Rho GDP dissociation inhibitor alpha
**Q92888-3**	ARHGEF1	-0.156	Rho guanine nucleotide exchange factor 1
**Q9HCE6**	ARHGEF10L	-0.296	Rho guanine nucleotide exchange factor 10 like
**Q9NZN5**	ARHGEF12	-0.401	Rho guanine nucleotide exchange factor 12
**Q5VV41**	ARHGEF16	-0.126	Rho guanine nucleotide exchange factor 16
**Q92974**	ARHGEF2	0.378	Rho/Rac guanine nucleotide exchange factor 2
**Q8N1W1-6**	ARHGEF28	-0.179	Rho guanine nucleotide exchange factor 28
**Q14155-5**	ARHGEF7	-0.277	Rho guanine nucleotide exchange factor 7
**Q8NFD5-3**	ARID1B	-0.18	AT-rich interaction domain 1B
**Q99856**	ARID3A	-0.21	AT-rich interaction domain 3A
**Q8IVW6**	ARID3B	0.186	AT-rich interaction domain 3B
**Q9Y4X5**	ARIH1	0.141	ariadne RBR E3 ubiquitin protein ligase 1
**P40616**	ARL1	-0.09	ADP ribosylation factor like GTPase 1
**P36405**	ARL3	-0.177	ADP ribosylation factor like GTPase 3
**Q15041**	ARL6IP1	0.391	ADP ribosylation factor like GTPase 6 interacting protein 1
**Q96C12**	ARMC5	0.61	armadillo repeat containing 5
**Q6NXE6-1**	ARMC6	-0.433	armadillo repeat containing 6
**Q9UH62**	ARMCX3	0.115	armadillo repeat containing X-linked 3
**Q5T2E6**	ARMH3	-0.343	armadillo like helical domain containing 3
**Q9H993**	ARMT1	-0.343	acidic residue methyltransferase 1
**O15143**	ARPC1B	-0.178	actin related protein 2/3 complex subunit 1B
**O15144**	ARPC2	-0.15	actin related protein 2/3 complex subunit 2
**O15145**	ARPC3	-0.339	actin related protein 2/3 complex subunit 3
**P59998-3**	ARPC4	-0.093	actin related protein 2/3 complex subunit 4
**O15511**	ARPC5	-0.179	actin related protein 2/3 complex subunit 5
**Q7Z6K5**	ARPIN/ARPIN-AP3S2	-0.394	ARPIN-AP3S2 readthrough
**P56211**	ARPP19	-0.228	cAMP regulated phosphoprotein 19

**P49407**	ARRB1	-0.312	arrestin beta 1
**P49407-2**	ARRB1	-0.179	arrestin beta 1
**P15848**	ARSB	-0.322	arylsulfatase B
**P51690**	ARSL	-0.721	arylsulfatase L
**O00192**	ARVCF	0.256	ARVCF delta catenin family member
**Q8TDY4-1**	ASAP3	-0.419	ArfGAP with SH3 domain, ankyrin repeat and PH domain 3
**Q8N9N2-2**	ASCC1	-0.452	activating signal cointegrator 1 complex subunit 1
**Q9H1I8-1**	ASCC2	-0.155	activating signal cointegrator 1 complex subunit 2
**Q8N3C0**	ASCC3	-0.355	activating signal cointegrator 1 complex subunit 3
**Q9NVP2**	ASF1B	0.443	anti-silencing function 1B histone chaperone
**P07306**	ASGR1	-0.279	asialoglycoprotein receptor 1
**P07307**	ASGR2	-0.329	asialoglycoprotein receptor 2
**P04424**	ASL	-0.173	argininosuccinate lyase
**O95671**	ASMTL	-0.158	acetylserotonin O-methyltransferase like
**P08243**	ASNS	0.183	asparagine synthetase (glutamine-hydrolyzing)
**Q9NWL6**	ASNSD1	-0.121	asparagine synthetase domain containing 1
**Q12797**	ASPH	0.322	aspartate beta-hydroxylase
**Q9BZE9-2**	ASPSCR1	-0.208	ASPSCR1 tether for SLC2A4, UBX domain containing
**Q7L266**	ASRGL1	-0.225	asparaginase and isoaspartyl peptidase 1
**P00966**	ASS1	-0.428	argininosuccinate synthase 1
**Q8NBU5**	ATAD1	0.441	ATPase family AAA domain containing 1
**Q6PL18-1**	ATAD2	0.117	ATPase family AAA domain containing 2
**Q9NVI7-2**	ATAD3A	0.257	ATPase family AAA domain containing 3A
**Q5T9A4**	ATAD3B	0.237	ATPase family AAA domain containing 3B
**Q5SQI0-1**	ATAT1	0.427	alpha tubulin acetyltransferase 1
**P15336-1**	ATF2	-0.406	activating transcription factor 2
**P18847**	ATF3	2.039	activating transcription factor 3
**Q9BSB4**	ATG101	0.638	autophagy related 101
**Q676U5**	ATG16L1	-0.12	autophagy related 16 like 1
**Q9Y4P1-2**	ATG4B	-0.115	autophagy related 4B cysteine peptidase
**O95352**	ATG7	-0.295	autophagy related 7
**P31939**	ATIC	-0.276	5-aminoimidazole-4-carboxamide ribonucleotide formyltransferase/IMP cyclohydrolase
**O00244**	ATOX1	-0.469	antioxidant 1 copper chaperone
**P98196**	ATP11A	-0.2	ATPase phospholipid transporting 11A
**P05023**	ATP1A1	0.123	ATPase Na+/K+ transporting subunit alpha 1
**P05026**	ATP1B1	0.331	ATPase Na+/K+ transporting subunit beta 1
**P54709**	ATP1B3	0.472	ATPase Na+/K+ transporting subunit beta 3
**P16615**	ATP2A2	0.156	ATPase sarcoplasmic/endoplasmic reticulum Ca2+ transporting 2
**P20020**	ATP2B1	0.122	ATPase plasma membrane Ca2+ transporting 1
**P25705-1**	ATP5F1A	0.348	ATP synthase F1 subunit alpha
**P06576**	ATP5F1B	0.231	ATP synthase F1 subunit beta
**P56381**	ATP5F1E	0.455	ATP synthase F1 subunit epsilon
**Q9UII2**	ATP5IF1	0.207	ATP synthase inhibitory factor subunit 1
**P56385**	ATP5ME	0.162	ATP synthase membrane subunit e
**O75964**	ATP5MG	0.3	ATP synthase membrane subunit g
**P24539**	ATP5PB	0.538	ATP synthase peripheral stalk-membrane subunit b
**O75947-1**	ATP5PD	0.183	ATP synthase peripheral stalk subunit d

**P18859-2**	ATP5PF	0.232	ATP synthase peripheral stalk subunit F6
**P48047**	ATP5PO	0.48	ATP synthase peripheral stalk subunit OSCP
**O75787**	ATP6AP2	0.26	ATPase H+ transporting accessory protein 2
**P61421**	ATP6V0D1	0.391	ATPase H+ transporting V0 subunit d1
**P38606**	ATP6V1A	-0.106	ATPase H+ transporting V1 subunit A
**P21283**	ATP6V1C1	0.115	ATPase H+ transporting V1 subunit C1
**Q9Y5K8**	ATP6V1D	0.23	ATPase H+ transporting V1 subunit D
**Q5TC12**	ATPAF1	0.358	ATP synthase mitochondrial F1 complex assembly factor 1
**Q8N5M1**	ATPAF2	0.262	ATP synthase mitochondrial F1 complex assembly factor 2
**O75882**	ATRN	-0.447	attractin
**Q9UBB4**	ATXN10	-0.188	ataxin 10
**Q8WWM7-1**	ATXN2L	-0.114	ataxin 2 like
**P54252**	ATXN3	0.48	ataxin 3
**Q9Y679-1**	AUP1	0.236	AUP1 lipid droplet regulating VLDL assembly factor
**O14965**	AURKA	0.269	aurora kinase A
**Q96GD4-5**	AURKB	0.58	aurora kinase B
**Q9NQS1**	AVEN	-0.193	apoptosis and caspase activation inhibitor
**Q8NBF6-1**	AVL9	-0.237	AVL9 cell migration associated
**O15169**	AXIN1	-0.326	axin 1
**O94766**	B3GAT3	0.201	beta-1,3-glucuronyltransferase 3
**Q9NY97**	B3GNT2	0.249	UDP-GlcNAc:betaGal beta-1,3-N-acetylglucosaminyltransferase 2
**O60513**	B4GALT4	-0.251	beta-1,4-galactosyltransferase 4
**O43505**	B4GAT1	-0.346	beta-1,4-glucuronyltransferase 1
**Q9NWV8**	BABAM1	-0.22	BRISC and BRCA1 A complex member 1
**Q9NXR7-1**	BABAM2	-0.326	BRISC and BRCA1 A complex member 2
**O95816**	BAG2	0.329	BCL2 associated athanogene 2
**O95817**	BAG3	0.791	BCL2 associated athanogene 3
**O95429**	BAG4	-0.346	BCL2 associated athanogene 4
**P46379-3**	BAG6	-0.096	BCL2 associated athanogene 6
**O75531**	BANF1	0.142	barrier to autointegration factor 1
**Q07812-2**	BAX	-0.088	BCL2 associated X, apoptosis regulator
**Q9NRL2**	BAZ1A	0.459	bromodomain adjacent to zinc finger domain 1A
**Q9UIG0**	BAZ1B	0.426	bromodomain adjacent to zinc finger domain 1B
**Q9UIF9**	BAZ2A	0.232	bromodomain adjacent to zinc finger domain 2A
**Q9BXH1**	BBC3	0.308	BCL2 binding component 3
**P50895**	BCAM	-0.35	basal cell adhesion molecule (Lutheran blood group)
**P51572-2**	BCAP31	0.417	B cell receptor associated protein 31
**P56945-6**	BCAR1	0.233	BCAR1 scaffold protein, Cas family member
**O75934**	BCAS2	0.167	BCAS2 pre-mRNA processing factor
**P54687-5**	BCAT1	-0.345	branched chain amino acid transaminase 1
**Q9P287-2**	BCCIP	0.285	BRCA2 and CDKN1A interacting protein
**P12694**	BCKDHA	0.371	branched chain keto acid dehydrogenase E1 subunit alpha
**O14874**	BCKDK	0.306	branched chain keto acid dehydrogenase kinase
**O95999**	BCL10	-0.199	BCL10 immune signaling adaptor
**Q86UU0**	BCL9L	0.191	BCL9 like
**Q9Y276**	BCS1L	0.337	BCS1 homolog, ubiquinol-cytochrome c reductase complex chaperone
**Q9BUT1**	BDH2	-0.237	3-hydroxybutyrate dehydrogenase 2

**Q5T5X7**	BEND3	0.129	BEN domain containing 3
**O15155**	BET1	0.331	Bet1 golgi vesicular membrane trafficking protein
**Q9NYM9**	BET1L	-0.395	Bet1 golgi vesicular membrane trafficking protein like
**O15392-2**	BIRC5	0.112	baculoviral IAP repeat containing 5
**Q9NR09**	BIRC6	-0.354	baculoviral IAP repeat containing 6
**Q13867**	BLMH	-0.193	bleomycin hydrolase
**P78537**	BLOC1S1	-0.227	biogenesis of lysosomal organelles complex 1 subunit 1
**P53004**	BLVRA	-0.174	biliverdin reductase A
**Q14692**	BMS1	-0.26	BMS1 ribosome biogenesis factor
**Q12983**	BNIP3	0.6	BCL2 interacting protein 3
**Q53S33**	BOLA3	0.283	bolA family member 3
**Q96GS4**	BORCS6	-0.413	BLOC-1 related complex subunit 6
**Q86WA6**	BPHL	0.385	biphenyl hydrolase like
**O95861-1**	BPNT1	-0.549	3'(2'), 5'-bisphosphate nucleotidase 1
**O95861-2**	BPNT1	-0.333	3'(2'), 5'-bisphosphate nucleotidase 1
**Q7Z569**	BRAP	0.117	BRCA1 associated protein
**P46736**	BRCC3	-0.213	BRCA1/BRCA2-containing complex subunit 3
**O95696-2**	BRD1	0.201	bromodomain containing 1
**P25440-2**	BRD2	0.356	bromodomain containing 2
**Q15059**	BRD3	-0.315	bromodomain containing 3
**Q5VW32**	BROX	-0.307	BRO1 domain and CAAX motif containing
**P55201-2**	BRPF1	0.304	bromodomain and PHD finger containing 1
**Q96K17**	BTF3L4	-0.249	basic transcription factor 3 like 4
**Q7KYR7**	BTN2A1	0.693	butyrophilin subfamily 2 member A1
**O43683**	BUB1	0.247	BUB1 mitotic checkpoint serine/threonine kinase
**O43684**	BUB3	0.178	BUB3 mitotic checkpoint protein
**P41223-2**	BUD31	0.403	BUD31 homolog
**Q9Y6E2**	BZW2	-0.361	basic leucine zipper and W2 domains 2
**Q9H0W9**	C11orf54	-0.221	chromosome 11 open reading frame 54
**O00193**	C11orf58	-0.373	chromosome 11 open reading frame 58
**E9PRG8**	C11orf98	-0.564	chromosome 11 open reading frame 98
**Q9HB07**	C12orf10	-0.152	chromosome 12 open reading frame 10
**Q8N5I9**	C12orf45	0.333	chromosome 12 open reading frame 45
**Q4G0I0**	C16orf91	0.31	chromosome 16 open reading frame 91
**Q9HAS0**	C17orf75	-0.261	chromosome 17 open reading frame 75
**Q9UNZ5**	C19orf53	0.566	chromosome 19 open reading frame 53
**Q6ZSJ8**	C1orf122	0.091	chromosome 1 open reading frame 122
**Q8NDD1**	C1orf131	-0.334	chromosome 1 open reading frame 131
**Q8N6N3**	C1orf52	-0.367	chromosome 1 open reading frame 52
**Q9GZN8-2**	C20orf27	-0.149	chromosome 20 open reading frame 27
**A6NCS6**	C2orf72	-0.175	chromosome 2 open reading frame 72
**P01024**	C3	-0.468	complement C3
**P01031**	C5	-0.298	complement C5
**Q8NC54**	C5orf15	-0.235	chromosome 5 open reading frame 15
**A6NDU8**	C5orf51	-0.419	chromosome 5 open reading frame 51
**Q9BRJ6**	C7orf50	-0.545	chromosome 7 open reading frame 50
**Q8IXQ3**	C9orf40	-0.347	chromosome 9 open reading frame 40

**Q5T6V5**	C9orf64	-0.315	chromosome 9 open reading frame 64
**Q9H8G2-1**	CAAP1	-0.359	caspase activity and apoptosis inhibitor 1
**Q9Y376**	CAB39	-0.432	calcium binding protein 39
**Q8WUQ7-2**	CACTIN	-0.111	cactin, spliceosome C complex subunit
**Q9HB71**	CACYBP	0.112	calcyclin binding protein
**P27708**	CAD	-0.158	carbamoyl-phosphate synthetase 2, aspartate transcarbamylase, and dihydroorotase
**Q9BY67-3**	CADM1	-0.243	cell adhesion molecule 1
**Q13137-4**	CALCOCO2	-0.22	calcium binding and coiled-coil domain 2
**O43852-3**	CALU	-0.148	calumenin
**O43852-4**	CALU	-0.315	calumenin
**Q14012**	CAMK1	-0.388	calcium/calmodulin dependent protein kinase I
**Q13557-10**	CAMK2D	-0.205	calcium/calmodulin dependent protein kinase II delta
**Q86VP6-1**	CAND1	-0.256	cullin associated and neddylation dissociated 1
**P27824-2**	CANX	0.094	calnexin
**P40123**	CAP2	-0.192	cyclase associated actin cytoskeleton regulatory protein 2
**P40121**	CAPG	-0.228	capping actin protein, gelsolin like
**P07384**	CAPN1	-0.172	calpain 1
**P04632**	CAPNS1	-0.21	calpain small subunit 1
**Q14444**	CAPRIN1	-0.215	cell cycle associated protein 1
**P52907**	CAPZA1	-0.292	capping actin protein of muscle Z-line subunit alpha 1
**P47755**	CAPZA2	-0.288	capping actin protein of muscle Z-line subunit alpha 2
**P47756-2**	CAPZB	-0.202	capping actin protein of muscle Z-line subunit beta
**Q96LW7-2**	CARD19	0.436	caspase recruitment domain family member 19
**Q9Y2V2**	CARHSP1	-0.092	calcium regulated heat stable protein 1
**Q86X55**	CARM1	-0.091	coactivator associated arginine methyltransferase 1
**P49589-3**	CARS1	-0.282	cysteinyl-tRNA synthetase 1
**Q9HA77**	CARS2	0.235	cysteinyl-tRNA synthetase 2, mitochondrial
**O15234**	CASC3	-0.231	CASC3 exon junction complex subunit
**O14936**	CASK	0.135	calcium/calmodulin dependent serine protein kinase
**P49662**	CASP4	0.301	caspase 4
**P55212**	CASP6	-0.094	caspase 6
**Q14790-9**	CASP8	-0.106	caspase 8
**P20810-6**	CAST	-0.269	calpastatin
**A6NHX0**	CASTOR2	-0.501	cytosolic arginine sensor for mTORC1 subunit 2
**O95810**	CAVIN2	0.145	caveolae associated protein 2
**P22681**	CBL	-0.214	Cbl proto-oncogene
**P16152**	CBR1	-0.155	carbonyl reductase 1
**Q8N4T8**	CBR4	0.22	carbonyl reductase 4
**P35520-2**	CBS/CBSL	-0.386	cystathionine beta-synthase
**Q5JTY5**	CBWD3/CBWD6	-0.157	COBW domain containing 3
**O00257**	CBX4	0.2	chromobox 4
**P45973**	CBX5	0.197	chromobox 5
**Q9HC52**	CBX8	0.433	chromobox 8
**Q8IWD4**	CCDC117	0.52	coiled-coil domain containing 117
**Q8WUD4**	CCDC12	-0.293	coiled-coil domain containing 12
**Q96CT7**	CCDC124	-0.12	coiled-coil domain containing 124
**Q96BQ5**	CCDC127	0.627	coiled-coil domain containing 127

**Q9H6E4**	CCDC134	0.206	coiled-coil domain containing 134
**Q6PK04**	CCDC137	-0.36	coiled-coil domain containing 137
**O60826**	CCDC22	-0.262	coiled-coil domain containing 22
**Q96MW1**	CCDC43	-0.4	coiled-coil domain containing 43
**Q96A33**	CCDC47	0.279	coiled-coil domain containing 47
**Q8IVM0**	CCDC50	-0.328	coiled-coil domain containing 50
**Q8IVM0-2**	CCDC50	-0.305	coiled-coil domain containing 50
**Q96ER9**	CCDC51	0.162	coiled-coil domain containing 51
**Q16204**	CCDC6	-0.332	coiled-coil domain containing 6
**A6NKD9**	CCDC85C	-0.398	coiled-coil domain containing 85C
**Q3V6T2-1**	CCDC88A	-0.189	coiled-coil domain containing 88A
**Q9P219**	CCDC88C	-0.632	coiled-coil domain containing 88C
**P20248**	CCNA2	0.218	cyclin A2
**O95067**	CCNB2	0.359	cyclin B2
**P51946**	CCNH	0.34	cyclin H
**O60563**	CCNT1	0.668	cyclin T1
**O14618**	CCS	-0.267	copper chaperone for superoxide dismutase
**P49368-1**	CCT3	-0.104	chaperonin containing TCP1 subunit 3
**P50991**	CCT4	-0.193	chaperonin containing TCP1 subunit 4
**P48643**	CCT5	-0.142	chaperonin containing TCP1 subunit 5
**Q99832**	CCT7	-0.132	chaperonin containing TCP1 subunit 7
**P50990**	CCT8	-0.188	chaperonin containing TCP1 subunit 8
**Q6YHK3**	CD109	0.316	CD109 molecule
**Q9Y5K6**	CD2AP	-0.31	CD2 associated protein
**Q13042**	CDC16	-0.254	cell division cycle 16
**Q12834**	CDC20	0.211	cell division cycle 20
**P49427**	CDC34	0.29	cell division cycle 34
**Q99459**	CDC5L	0.168	cell division cycle 5 like
**Q6P1J9**	CDC73	0.105	cell division cycle 73
**Q69YH5-1**	CDCA2	0.409	cell division cycle associated 2
**Q96FF9**	CDCA5	0.532	cell division cycle associated 5
**Q53HL2**	CDCA8	0.215	cell division cycle associated 8
**P19022**	CDH2	-0.233	cadherin 2
**O14735**	CDIPT	-0.975	CDP-diacylglycerol--inositol 3-phosphatidyltransferase
**P50750-2**	CDK9	0.408	cyclin dependent kinase 9
**Q5VV42**	CDKAL1	-0.205	CDK5 regulatory subunit associated protein 1 like 1
**P38936**	CDKN1A	1.182	cyclin dependent kinase inhibitor 1A
**Q9NXV6**	CDKN2AIP	0.338	CDKN2A interacting protein
**Q96HQ2-1**	CDKN2AIPNL	-0.501	CDKN2A interacting protein N-terminal like
**O95674-1**	CDS2	-0.115	CDP-diacylglycerol synthase 2
**Q9UKY7**	CDV3	-0.218	CDV3 homolog
**P13688**	CEACAM1	0.32	CEA cell adhesion molecule 1
**P49715-4**	CEBPA	-0.396	CCAAT enhancer binding protein alpha
**Q03701**	CEBPZ	0.315	CCAAT enhancer binding protein zeta
**Q9UHN6**	CEMIP2	0.249	cell migration inducing hyaluronidase 2
**P49454**	CENPF	0.229	centromere protein F
**Q7Z7K6-1**	CENPV	0.497	centromere protein V

**Q9UPN4**	CEP131	-0.134	centrosomal protein 131
**O15078**	CEP290	-0.159	centrosomal protein 290
**Q8TAP6**	CEP76	0.545	centrosomal protein 76
**Q96G23**	CERS2	0.205	ceramide synthase 2
**Q9HA82**	CERS4	-0.129	ceramide synthase 4
**Q9Y5P4-3**	CERT1	-0.382	ceramide transporter 1
**O00748**	CES2	0.129	carboxylesterase 2
**P41208**	CETN2	-0.169	centrin 2
**Q9Y6A4**	CFAP20	0.177	cilia and flagella associated protein 20
**Q9UEE9-1**	CFDP1	-0.631	craniofacial development protein 1
**P05156**	CFI	-0.36	complement factor I
**P23528**	CFL1	-0.121	cofilin 1
**Q9Y281-1**	CFL2	-0.338	cofilin 2
**Q9P2M7-1**	CGN	-0.788	cingulin
**Q0VF96**	CGNL1	-0.861	cingulin like 1
**Q13111-1**	CHAF1A	-0.221	chromatin assembly factor 1 subunit A
**Q13112**	CHAF1B	0.125	chromatin assembly factor 1 subunit B
**Q9NX63**	CHCHD3	0.17	coiled-coil-helix-coiled-coil-helix domain containing 3
**O14646**	CHD1	0.283	chromodomain helicase DNA binding protein 1
**O14647**	CHD2	0.3	chromodomain helicase DNA binding protein 2
**Q14839-2**	CHD4	0.266	chromodomain helicase DNA binding protein 4
**Q9HCK8-1**	CHD8	0.192	chromodomain helicase DNA binding protein 8
**Q8NE62**	CHDH	0.131	choline dehydrogenase
**O14757**	CHEK1	-0.53	checkpoint kinase 1
**O96017-9**	CHEK2	-0.199	checkpoint kinase 2
**Q8IWX8**	CHERP	0.243	calcium homeostasis endoplasmic reticulum protein
**Q9BWS9-1**	CHID1	-0.18	chitinase domain containing 1
**P35790**	CHKA	-0.227	choline kinase alpha
**P24386-1**	CHM	-0.181	CHM Rab escort protein
**Q9HD42**	CHMP1A	-0.231	charged multivesicular body protein 1A
**Q9UQN3-1**	CHMP2B	0.098	charged multivesicular body protein 2B
**Q9BY43-2**	CHMP4A	-0.29	charged multivesicular body protein 4A
**Q9H444**	CHMP4B	0.14	charged multivesicular body protein 4B
**Q9NZZ3**	CHMP5	0.162	charged multivesicular body protein 5
**Q96FZ7**	CHMP6	0.095	charged multivesicular body protein 6
**Q9UHD1**	CHORDC1	0.47	cysteine and histidine rich domain containing 1
**Q99653**	CHP1	0.501	calcineurin like EF-hand protein 1
**Q8NET6**	CHST13	-0.418	carbohydrate sulfotransferase 13
**Q8WVB6-2**	CHTF18	-0.346	chromosome transmission fidelity factor 18
**O76071**	CIAO1	-0.199	cytosolic iron-sulfur assembly component 1
**Q9Y3D0**	CIAO2B	-0.273	cytosolic iron-sulfur assembly component 2B
**Q6FI81**	CIAPIN1	-0.125	cytokine induced apoptosis inhibitor 1
**Q99828-2**	CIB1	0.283	calcium and integrin binding 1
**Q8TCG1**	CIP2A	-0.134	cell proliferation regulating inhibitor of protein phosphatase 2A
**Q86X95**	CIR1	0.155	corepressor interacting with RBPJ, 1
**Q14011**	CIRBP	-0.27	cold inducible RNA binding protein
**Q9NZ45**	CISD1	0.112	CDGSH iron sulfur domain 1

**O14578-4**	CIT	-0.328	citron rho-interacting serine/threonine kinase
**Q8WWK9**	CKAP2	0.914	cytoskeleton associated protein 2
**Q07065**	CKAP4	0.316	cytoskeleton associated protein 4
**P12277**	CKB	-0.167	creatine kinase B
**Q7Z460-1**	CLASP1	-0.19	cytoplasmic linker associated protein 1
**O75122-3**	CLASP2	-0.222	cytoplasmic linker associated protein 2
**P56747**	CLDN6	0.299	claudin 6
**Q9Y696**	CLIC4	-0.25	chloride intracellular channel 4
**Q14677**	CLINT1	-0.375	clathrin interactor 1
**Q14677-3**	CLINT1	-0.361	clathrin interactor 1
**P49761**	CLK3	0.686	CDC like kinase 3
**Q9NWW5**	CLN6	0.103	CLN6 transmembrane ER protein
**Q16740**	CLPP	0.28	caseinolytic mitochondrial matrix peptidase proteolytic subunit
**O76031**	CLPX	0.223	caseinolytic mitochondrial matrix peptidase chaperone subunit
**Q9HAW4**	CLSPN	0.312	claspin
**P09496-2**	CLTA	-0.152	clathrin light chain A
**P09497-2**	CLTB	0.462	clathrin light chain B
**P10909-2**	CLU	0.877	clusterin
**I3L2B0**	CLUH	-0.367	clustered mitochondria homolog
**O75153**	CLUH	-0.354	clustered mitochondria homolog
**Q8N0X4**	CLYBL	0.126	citrate lyase beta like
**Q96DG6**	CMBL	0.267	carboxymethylenebutenolidase homolog
**Q8IY22**	CMIP	-0.694	c-Maf inducing protein
**Q9BQ75**	CMSS1	-0.185	cms1 ribosomal small subunit homolog
**P62633**	CNBP	-1.238	CCHC-type zinc finger nucleic acid binding protein
**P62633-6**	CNBP	-0.982	CCHC-type zinc finger nucleic acid binding protein
**Q96KP4**	CNDP2	-0.335	carnosine dipeptidase 2
**Q99439**	CNN2	-0.118	calponin 2
**Q15417**	CNN3	0.103	calponin 3
**A5YKK6**	CNOT1	-0.207	CCR4-NOT transcription complex subunit 1
**Q9H9A5-6**	CNOT10	-0.27	CCR4-NOT transcription complex subunit 10
**Q9NZN8**	CNOT2	-0.18	CCR4-NOT transcription complex subunit 2
**Q96LI5**	CNOT6L	-0.452	CCR4-NOT transcription complex subunit 6 like
**Q9UIV1-1**	CNOT7	-0.521	CCR4-NOT transcription complex subunit 7
**Q92600**	CNOT9	-0.331	CCR4-NOT transcription complex subunit 9
**Q9Y2R0**	COA3	0.127	cytochrome c oxidase assembly factor 3
**Q9NYJ1-2**	COA4	-0.45	cytochrome c oxidase assembly factor 4 homolog
**Q96JB2-1**	COG3	-0.194	component of oligomeric golgi complex 3
**Q9H9E3**	COG4	-0.161	component of oligomeric golgi complex 4
**Q9Y2V7-1**	COG6	-0.129	component of oligomeric golgi complex 6
**Q96MW5**	COG8	-0.12	component of oligomeric golgi complex 8
**P38432**	COIL	0.272	coilin
**P39060-3**	COL18A1	-0.296	collagen type XVIII alpha 1 chain
**Q86X83**	COMMD2	-0.126	COMM domain containing 2
**Q7Z4G1-2**	COMMD6	-0.704	COMM domain containing 6
**Q9NX08**	COMMD8	-0.489	COMM domain containing 8
**Q9P000-1**	COMMD9	-0.535	COMM domain containing 9

**P53621-2**	COPA	-0.145	coatomer protein complex subunit alpha
**P53618**	COPB1	-0.135	coatomer protein complex subunit beta 1
**P35606**	COPB2	-0.17	coatomer protein complex subunit beta 2
**O14579-1**	COPE	-0.194	coatomer protein complex subunit epsilon
**Q9Y678**	COPG1	-0.103	coatomer protein complex subunit gamma 1
**Q9UBF2**	COPG2	-0.23	coatomer protein complex subunit gamma 2
**P61201-1**	COPS2	-0.173	COP9 signalosome subunit 2
**Q9UNS2**	COPS3	-0.182	COP9 signalosome subunit 3
**Q9BT78**	COPS4	-0.21	COP9 signalosome subunit 4
**Q92905**	COPS5	-0.176	COP9 signalosome subunit 5
**Q9UBW8**	COPS7A	-0.151	COP9 signalosome subunit 7A
**Q99627**	COPS8	-0.126	COP9 signalosome subunit 8
**P61923-4**	COPZ1	-0.138	coatomer protein complex subunit zeta 1
**Q5HYK3-1**	COQ5	-0.394	coenzyme Q5, methyltransferase
**P31146**	CORO1A	-0.187	coronin 1A
**Q9ULV4-3**	CORO1C	0.154	coronin 1C
**Q14019**	COTL1	-0.414	coactosin like F-actin binding protein 1
**Q7KZN9**	COX15	-0.092	cytochrome c oxidase assembly homolog COX15
**P13073**	COX4I1	0.289	cytochrome c oxidase subunit 4I1
**P20674**	COX5A	0.142	cytochrome c oxidase subunit 5A
**P09669**	COX6C	0.198	cytochrome c oxidase subunit 6C
**P14406**	COX7A2	0.16	cytochrome c oxidase subunit 7A2
**O75976**	CPD	-0.405	carboxypeptidase D
**Q17RY0-1**	CPEB4	0.255	cytoplasmic polyadenylation element binding protein 4
**Q6PUV4**	CPLX2	-0.569	complexin 2
**Q9BRF8-1**	CPPED1	-0.392	calcineurin like phosphoesterase domain containing 1
**P31327-3**	CPS1	0.149	carbamoyl-phosphate synthase 1
**Q16630-2**	CPSF6	0.141	cleavage and polyadenylation specific factor 6
**P50416**	CPT1A	0.116	carnitine palmitoyltransferase 1A
**P23786**	CPT2	0.403	carnitine palmitoyltransferase 2
**P78560**	CRADD	-0.389	CASP2 and RIPK1 domain containing adaptor with death domain
**P43155**	CRAT	0.441	carnitine O-acetyltransferase
**Q92793-1**	CREBBP	-0.26	CREB binding protein
**P52943**	CRIP2	0.219	cysteine rich protein 2
**P46108-1**	CRK	-0.275	CRK proto-oncogene, adaptor protein
**P46109**	CRKL	-0.245	CRK like proto-oncogene, adaptor protein
**Q8IUI8**	CRLF3	-0.163	cytokine receptor like factor 3
**Q9BZJ0**	CRNKL1	0.219	crooked neck pre-mRNA splicing factor 1
**P02511**	CRYAB	1.788	crystallin alpha B
**Q9Y2S2-1**	CRYL1	-0.381	crystallin lambda 1
**Q08257**	CRYZ	0.16	crystallin zeta
**O75390**	CS	0.31	citrate synthase
**P55060**	CSE1L	-0.361	chromosome segregation 1 like
**P41240**	CSK	-0.189	C-terminal Src kinase
**P48729**	CSNK1A1	0.136	casein kinase 1 alpha 1
**P68400**	CSNK2A1	0.226	casein kinase 2 alpha 1
**P19784**	CSNK2A2	0.301	casein kinase 2 alpha 2

**P67870**	CSNK2B	0.361	casein kinase 2 beta
**P01034**	CST3	-0.296	cystatin C
**Q12996**	CSTF3	0.275	cleavage stimulation factor subunit 3
**Q13363**	CTBP1	-0.158	C-terminal binding protein 1
**P49711-1**	CTCF	0.251	CCCTC-binding factor
**Q9Y5B0**	CTDP1	-0.208	CTD phosphatase subunit 1
**P32929**	CTH	0.252	cystathionine gamma-lyase
**P35221**	CTNNA1	-0.273	catenin alpha 1
**Q9NSA3**	CTNNBIP1	-0.505	catenin beta interacting protein 1
**Q8WYA6**	CTNNBL1	-0.118	catenin beta like 1
**O60716-3**	CTNND1	-0.146	catenin delta 1
**P17812**	CTPS1	-0.158	CTP synthase 1
**Q9NRF8**	CTPS2	-0.31	CTP synthase 2
**Q6PD62**	CTR9	0.328	CTR9 homolog, Paf1/RNA polymerase II complex component
**P07858**	CTSB	0.186	cathepsin B
**P07711**	CTSL	0.529	cathepsin L
**Q14247-1**	CTTN	-0.33	cortactin
**Q2VPK5**	CTU2	-0.183	cytosolic thiouridylase subunit 2
**Q13616**	CUL1	-0.176	cullin 1
**Q13617-2**	CUL2	-0.1	cullin 2
**Q13619-1**	CUL4A	-0.096	cullin 4A
**Q93034**	CUL5	-0.174	cullin 5
**O60888-2**	CUTA	-0.168	cutA divalent cation tolerance homolog
**P39880-3**	CUX1	-0.354	cut like homeobox 1
**Q6UX04**	CWC27	-0.216	CWC27 spliceosome associated cyclophilin
**Q2TBE0**	CWF19L2	-0.111	CWF19 like cell cycle control factor 2
**P78310-1**	CXADR	-0.108	CXADR Ig-like cell adhesion molecule
**Q9P0U4-2**	CXXC1	0.199	CXXC finger protein 1
**P00167-1**	CYB5A	-0.414	cytochrome b5 type A
**Q9UHQ9**	CYB5R1	0.362	cytochrome b5 reductase 1
**P00387-3**	CYB5R3	0.205	cytochrome b5 reductase 3
**Q7L1T6**	CYB5R4	-0.142	cytochrome b5 reductase 4
**P08574**	CYC1	0.302	cytochrome c1
**Q7L576-1**	CYFIP1	-0.241	cytoplasmic FMR1 interacting protein 1
**Q07973-1**	CYP24A1	0.262	cytochrome P450 family 24 subfamily A member 1
**Q96SQ9-2**	CYP2S1	0.436	cytochrome P450 family 2 subfamily S member 1
**Q8TAV3**	CYP2W1	-0.229	cytochrome P450 family 2 subfamily W member 1
**Q9HBI6**	CYP4F11	0.333	cytochrome P450 family 4 subfamily F member 11
**Q16850-1**	CYP51A1	-0.564	cytochrome P450 family 51 subfamily A member 1
**Q9Y4D1**	DAAM1	-0.182	dishevelled associated activator of morphogenesis 1
**Q5VWQ8**	DAB2IP	-0.418	DAB2 interacting protein
**Q14118**	DAG1	-0.287	dystroglycan 1
**P51397**	DAP	-0.539	death associated protein
**O43293-1**	DAPK3	0.274	death associated protein kinase 3
**Q6PI48**	DARS2	0.256	aspartyl-tRNA synthetase 2, mitochondrial
**P07108-5**	DBI	-0.948	diazepam binding inhibitor, acyl-CoA binding protein
**Q9UK59**	DBR1	0.207	debranching RNA lariats 1

**P11182**	DBT	0.328	dihydrolipoamide branched chain transacylase E2
**Q9Y4B6**	DCAF1	-0.321	DDB1 and CUL4 associated factor 1
**Q8TEB1**	DCAF11	-0.11	DDB1 and CUL4 associated factor 11
**Q96JK2**	DCAF5	0.154	DDB1 and CUL4 associated factor 5
**P61962**	DCAF7	0.379	DDB1 and CUL4 associated factor 7
**Q8WVC6-1**	DCAKD	0.152	dephospho-CoA kinase domain containing
**Q9NPI6**	DCP1A	0.197	decapping mRNA 1A
**Q8IZD4**	DCP1B	0.205	decapping mRNA 1B
**Q96C86**	DCPS	-0.404	decapping enzyme, scavenger
**Q14203-4**	DCTN1	-0.215	dynactin subunit 1
**Q13561-2**	DCTN2	-0.245	dynactin subunit 2
**O75935**	DCTN3	-0.219	dynactin subunit 3
**Q9UJW0-3**	DCTN4	0.312	dynactin subunit 4
**O00399**	DCTN6	0.362	dynactin subunit 6
**Q9H773**	DCTPP1	-0.434	dCTP pyrophosphatase 1
**Q8IWE4**	DCUN1D3	0.353	defective in cullin neddylation 1 domain containing 3
**Q7Z4W1**	DCXR	0.173	dicarbonyl and L-xylulose reductase
**Q9BW61**	DDA1	-0.175	DET1 and DDB1 associated 1
**O94760**	DDAH1	-0.401	dimethylarginine dimethylaminohydrolase 1
**Q16531**	DDB1	-0.172	damage specific DNA binding protein 1
**Q92466**	DDB2	0.393	damage specific DNA binding protein 2
**Q5TDH0-3**	DDI2	-0.302	DNA damage inducible 1 homolog 2
**P39656**	DDOST	0.198	dolichyl-diphosphooligosaccharide--protein glycosyltransferase non-catalytic subunit
**P30046**	DDT	-0.396	D-dopachrome tautomerase
**Q92841**	DDX17	-0.128	DEAD-box helicase 17
**Q9NUU7**	DDX19A	-0.164	DEAD-box helicase 19A
**Q9NR30**	DDX21	0.507	DExD-box helicase 21
**Q9BUQ8**	DDX23	0.125	DEAD-box helicase 23
**Q9GZR7**	DDX24	0.292	DEAD-box helicase 24
**Q96GQ7**	DDX27	-0.214	DEAD-box helicase 27
**O00148**	DDX39A	-0.221	DExD-box helicase 39A
**Q13838-2**	DDX39B	-0.176	DExD-box helicase 39B
**O15523**	DDX3Y	0.182	DEAD-box helicase 3 Y-linked
**Q86XP3**	DDX42	-0.166	DEAD-box helicase 42
**Q9H0S4**	DDX47	0.224	DEAD-box helicase 47
**P17844**	DDX5	-0.121	DEAD-box helicase 5
**Q9Y2R4**	DDX52	0.266	DExD-box helicase 52
**Q9NY93**	DDX56	-0.566	DEAD-box helicase 56
**P26196**	DDX6	0.115	DEAD-box helicase 6
**Q16698**	DECR1	0.368	2,4-dienoyl-CoA reductase 1
**Q9NUI1**	DECR2	0.275	2,4-dienoyl-CoA reductase 2
**Q9H4E7**	DEF6	-0.145	DEF6 guanine nucleotide exchange factor
**Q8TCE6-1**	DENND10	-0.275	DENN domain containing 10
**O43583**	DENR	-0.418	density regulated re-initiation and release factor
**Q9Y315**	DERA	-0.194	deoxyribose-phosphate aldolase
**Q8WYQ5-1**	DGCR8	0.416	DGCR8 microprocessor complex subunit
**Q5KSL6**	DGKK	-0.44	diacylglycerol kinase kappa

**Q16854**	DGUOK	0.216	deoxyguanosine kinase
**Q02127**	DHODH	0.146	dihydroorotate dehydrogenase (quinone)
**Q13268**	DHRS2	0.124	dehydrogenase/reductase 2
**Q9BTZ2**	DHRS4	0.256	dehydrogenase/reductase 4
**Q9Y394**	DHRS7	0.293	dehydrogenase/reductase 7
**O43143**	DHX15	0.161	DEAH-box helicase 15
**Q7L2E3-2**	DHX30	0.374	DExH-box helicase 30
**Q9H5Z1**	DHX35	0.317	DEAH-box helicase 35
**Q92620**	DHX38	0.156	DEAH-box helicase 38
**Q6P158-1**	DHX57	-0.44	DExH-box helicase 57
**Q08211**	DHX9	0.191	DExH-box helicase 9
**O60610-1**	DIAPH1	-0.268	diaphanous related formin 1
**Q9UPY3**	DICER1	-0.198	dicer 1, ribonuclease III
**Q9BTC0**	DIDO1	0.277	death inducer-obliterator 1
**Q9UNQ2**	DIMT1	-0.207	DIMT1 rRNA methyltransferase and ribosome maturation factor
**Q9P265**	DIP2B	-0.259	disco interacting protein 2 homolog B
**Q9Y2L1**	DIS3	0.151	DIS3 homolog, exosome endoribonuclease and 3'-5' exoribonuclease
**Q8IYB7**	DIS3L2	-0.305	DIS3 like 3'-5' exoribonuclease 2
**O60832**	DKC1	0.29	dyskerin pseudouridine synthase 1
**P10515**	DLAT	0.109	dihydrolipoamide S-acetyltransferase
**P09622**	DLD	0.363	dihydrolipoamide dehydrogenase
**Q9Y2H0**	DLGAP4	-0.173	DLG associated protein 4
**P36957**	DLST	0.289	dihydrolipoamide S-succinyltransferase
**Q9NPF5**	DMAP1	0.126	DNA methyltransferase 1 associated protein 1
**P11532-6**	DMD	-0.155	dystrophin
**Q08495-1**	DMTN	-0.268	dematin actin binding protein
**Q86Y56**	DNAAF5	-0.421	dynein axonemal assembly factor 5
**P31689**	DNAJA1	0.543	DnaJ heat shock protein family (Hsp40) member A1
**P25685**	DNAJB1	0.934	DnaJ heat shock protein family (Hsp40) member B1
**Q9UBS4**	DNAJB11	0.122	DnaJ heat shock protein family (Hsp40) member B11
**P25686-3**	DNAJB2	0.478	DnaJ heat shock protein family (Hsp40) member B2
**Q9UDY4**	DNAJB4	1.193	DnaJ heat shock protein family (Hsp40) member B4
**Q9NVH1-1**	DNAJC11	0.162	DnaJ heat shock protein family (Hsp40) member C11
**O75165**	DNAJC13	-0.112	DnaJ heat shock protein family (Hsp40) member C13
**Q9Y5T4**	DNAJC15	-0.583	DnaJ heat shock protein family (Hsp40) member C15
**Q9NVM6**	DNAJC17	-0.142	DnaJ heat shock protein family (Hsp40) member C17
**Q96DA6**	DNAJC19	0.398	DnaJ heat shock protein family (Hsp40) member C19
**Q99543-1**	DNAJC2	-0.288	DnaJ heat shock protein family (Hsp40) member C2
**Q9H3Z4**	DNAJC5	-0.693	DnaJ heat shock protein family (Hsp40) member C5
**Q99615-1**	DNAJC7	0.117	DnaJ heat shock protein family (Hsp40) member C7
**P50570**	DNM2	-0.364	dynamin 2
**Q9Y6K1**	DNMT3A	0.131	DNA methyltransferase 3 alpha
**Q9ULA0**	DNPEP	-0.282	aspartyl aminopeptidase
**O43598-1**	DNPH1	-0.462	2'-deoxynucleoside 5'-phosphate N-hydrolase 1
**Q5QJE6**	DNTTIP2	0.385	deoxynucleotidyltransferase terminal interacting protein 2
**Q8N1I0-3**	DOCK4	-0.25	dedicator of cytokinesis 4
**Q9H3H5**	DPAGT1	0.926	dolichyl-phosphate N-acetylglucosaminephosphotransferase 1

**Q9BZG8**	DPH1	-0.495	diphthamide biosynthesis 1
**Q9BQC3**	DPH2	-0.179	diphthamide biosynthesis 2
**Q9H2P9-5**	DPH5	-0.64	diphthamide biosynthesis 5
**O60762**	DPM1	0.106	dolichyl-phosphate mannosyltransferase subunit 1, catalytic
**Q9NY33**	DPP3	-0.2	dipeptidyl peptidase 3
**P27487**	DPP4	0.154	dipeptidyl peptidase 4
**Q86TI2-2**	DPP9	-0.188	dipeptidyl peptidase 9
**Q16555**	DPYSL2	-0.331	dihydropyrimidinase like 2
**Q9Y295**	DRG1	-0.187	developmentally regulated GTP binding protein 1
**Q9H410**	DSN1	-0.281	DSN1 component of MIS12 kinetochore complex
**P60981-1**	DSTN	0.165	destrin, actin depolymerizing factor
**Q8TEA8**	DTD1	-0.139	D-aminoacyl-tRNA deacylase 1
**P23919**	DTYMK	-0.136	deoxythymidylate kinase
**Q6P1R4**	DUS1L	-0.14	dihydrouridine synthase 1 like
**P28562**	DUSP1	1.431	dual specificity phosphatase 1
**O75319-1**	DUSP11	0.221	dual specificity phosphatase 11
**P33316-3**	DUT	-0.261	deoxyuridine triphosphatase
**O14641**	DVL2	-0.283	dishevelled segment polarity protein 2
**Q14204**	DYNC1H1	-0.261	dynein cytoplasmic 1 heavy chain 1
**Q9Y6G9**	DYNC1LI1	-0.17	dynein cytoplasmic 1 light intermediate chain 1
**O43237**	DYNC1LI2	-0.11	dynein cytoplasmic 1 light intermediate chain 2
**P63167**	DYNLL1	0.865	dynein light chain LC8-type 1
**Q96FJ2**	DYNLL2	0.539	dynein light chain LC8-type 2
**Q9NP97**	DYNLRB1	-0.13	dynein light chain roadblock-type 1
**Q16254**	E2F4	-0.272	E2F transcription factor 4
**Q99848**	EBNA1BP2	-0.218	EBNA1 binding protein 2
**O95905-3**	ECD	0.288	ecdysoneless cell cycle regulator
**P42892**	ECE1	-0.394	endothelin converting enzyme 1
**Q13011**	ECH1	0.287	enoyl-CoA hydratase 1
**Q9NTX5**	ECHDC1	0.219	ethylmalonyl-CoA decarboxylase 1
**P30084**	ECHS1	0.187	enoyl-CoA hydratase, short chain 1
**P42126**	ECI1	0.204	enoyl-CoA delta isomerase 1
**O75521**	ECI2	0.342	enoyl-CoA delta isomerase 2
**Q5VYK3**	ECPAS	-0.135	Ecm29 proteasome adaptor and scaffold
**Q9BQ95**	ECSIT	0.261	ECSIT signalling integrator
**Q9H8V3-3**	ECT2	0.27	epithelial cell transforming 2
**O60869**	EDF1	0.254	endothelial differentiation related factor 1
**P68104**	EEF1A1	0.127	eukaryotic translation elongation factor 1 alpha 1
**P26641-2**	EEF1G	-0.109	eukaryotic translation elongation factor 1 gamma
**P13639**	EEF2	-0.168	eukaryotic translation elongation factor 2
**O00418**	EEF2K	-0.476	eukaryotic elongation factor 2 kinase
**Q7Z2Z2**	EFL1	-0.355	elongation factor like GTPase 1
**Q8NDI1**	EHBP1	-0.436	EH domain binding protein 1
**Q9H4M9**	EHD1	-0.092	EH domain containing 1
**Q08426**	EHHADH	0.257	enoyl-CoA hydratase and 3-hydroxyacyl CoA dehydrogenase
**P41567**	EIF1	0.653	eukaryotic translation initiation factor 1
**P19525**	EIF2AK2	0.154	eukaryotic translation initiation factor 2 alpha kinase 2

**Q9NZJ5**	EIF2AK3	-0.305	eukaryotic translation initiation factor 2 alpha kinase 3
**Q9P2K8-1**	EIF2AK4	-0.218	eukaryotic translation initiation factor 2 alpha kinase 4
**Q14232**	EIF2B1	-0.347	eukaryotic translation initiation factor 2B subunit alpha
**Q9NR50-1**	EIF2B3	-0.138	eukaryotic translation initiation factor 2B subunit gamma
**Q13144**	EIF2B5	-0.219	eukaryotic translation initiation factor 2B subunit epsilon
**P41214**	EIF2D	-0.326	eukaryotic translation initiation factor 2D
**P05198**	EIF2S1	0.098	eukaryotic translation initiation factor 2 subunit alpha
**Q14152**	EIF3A	-0.196	eukaryotic translation initiation factor 3 subunit A
**P55884-2**	EIF3B	-0.115	eukaryotic translation initiation factor 3 subunit B
**Q99613**	EIF3C	-0.105	eukaryotic translation initiation factor 3 subunit C
**O75821**	EIF3G	-0.307	eukaryotic translation initiation factor 3 subunit G
**O15372**	EIF3H	-0.088	eukaryotic translation initiation factor 3 subunit H
**O75822**	EIF3J	-0.29	eukaryotic translation initiation factor 3 subunit J
**Q9UBQ5**	EIF3K	-0.48	eukaryotic translation initiation factor 3 subunit K
**Q7L2H7**	EIF3M	-0.261	eukaryotic translation initiation factor 3 subunit M
**Q14240-2**	EIF4A2	-0.214	eukaryotic translation initiation factor 4A2
**P38919**	EIF4A3	0.191	eukaryotic translation initiation factor 4A3
**P23588**	EIF4B	-0.299	eukaryotic translation initiation factor 4B
**P06730-2**	EIF4E	-0.178	eukaryotic translation initiation factor 4E
**O60573**	EIF4E2	-0.235	eukaryotic translation initiation factor 4E family member 2
**Q13541**	EIF4EBP1	-0.288	eukaryotic translation initiation factor 4E binding protein 1
**Q04637-9**	EIF4G1	-0.308	eukaryotic translation initiation factor 4 gamma 1
**O43432-3**	EIF4G3	-0.403	eukaryotic translation initiation factor 4 gamma 3
**Q15056-1**	EIF4H	-0.296	eukaryotic translation initiation factor 4H
**P55010**	EIF5	0.152	eukaryotic translation initiation factor 5
**P63241-2**	EIF5A	-0.198	eukaryotic translation initiation factor 5A
**Q9BQ52**	ELAC2	-0.108	elaC ribonuclease Z 2
**P32519**	ELF1	-0.173	E74 like ETS transcription factor 1
**P55199**	ELL	0.507	elongation factor for RNA polymerase II
**O00472**	ELL2	0.224	elongation factor for RNA polymerase II 2
**Q14241**	ELOA	0.496	elongin A
**Q15370-2**	ELOB	-0.249	elongin B
**O95163**	ELP1	-0.274	elongator complex protein 1
**Q6IA86-6**	ELP2	-0.197	elongator acetyltransferase complex subunit 2
**Q9H9T3**	ELP3	-0.366	elongator acetyltransferase complex subunit 3
**Q15006**	EMC2	0.214	ER membrane protein complex subunit 2
**Q9P0I2**	EMC3	0.212	ER membrane protein complex subunit 3
**Q9NPA0**	EMC7	0.134	ER membrane protein complex subunit 7
**O43402**	EMC8	0.187	ER membrane protein complex subunit 8
**P50402**	EMD	0.167	emerin
**Q92979**	EMG1	-0.136	EMG1 N1-specific pseudouridine methyltransferase
**Q9HC35**	EML4	-0.165	EMAP like 4
**Q8N8S7**	ENAH	-0.224	ENAH actin regulator
**O14682**	ENC1	0.674	ectodermal-neural cortex 1
**O94919**	ENDOD1	0.191	endonuclease domain containing 1
**Q14249**	ENDOG	-0.093	endonuclease G
**P06733-1**	ENO1	-0.259	enolase 1

**P09104**	ENO2	-0.153	enolase 2
**P13929-1**	ENO3	-0.486	enolase 3
**Q9UHY7**	ENOPH1	-0.362	enolase-phosphatase 1
**Q7L5Y1-5**	ENOSF1	-0.202	enolase superfamily member 1
**P22413**	ENPP1	-0.23	ectonucleotide pyrophosphatase/phosphodiesterase 1
**O43768-4**	ENSA	-0.527	endosulfine alpha
**Q5NDL2**	EOGT	-0.132	EGF domain specific O-linked N-acetylglucosamine transferase
**O43491-4**	EPB41L2	-0.244	erythrocyte membrane protein band 4.1 like 2
**P16422**	EPCAM	-0.177	epithelial cell adhesion molecule
**P29317**	EPHA2	0.832	EPH receptor A2
**P54760**	EPHB4	0.155	EPH receptor B4
**P07099**	EPHX1	0.445	epoxide hydrolase 1
**Q7L775**	EPM2AIP1	0.198	EPM2A interacting protein 1
**O95208**	EPN2	-0.569	epsin 2
**P42566**	EPS15	-0.236	epidermal growth factor receptor pathway substrate 15
**O75616**	ERAL1	0.303	Era like 12S mitochondrial rRNA chaperone 1
**Q9NZ08-2**	ERAP1	-0.116	endoplasmic reticulum aminopeptidase 1
**P04626**	ERBB2	-0.268	erb-b2 receptor tyrosine kinase 2
**P21860**	ERBB3	-0.316	erb-b2 receptor tyrosine kinase 3
**Q8IUD2-1**	ERC1	-0.353	ELKS/RAB6-interacting/CAST family member 1
**P18074-1**	ERCC2	-0.084	ERCC excision repair 2, TFIIH core complex helicase subunit
**P19447**	ERCC3	0.516	ERCC excision repair 3, TFIIH core complex helicase subunit
**Q969X5**	ERGIC1	0.102	endoplasmic reticulum-golgi intermediate compartment 1
**Q96RQ1**	ERGIC2	0.124	ERGIC and golgi 2
**Q9Y282-3**	ERGIC3	0.325	ERGIC and golgi 3
**P84090**	ERH	0.25	ERH mRNA splicing and mitosis factor
**O94905**	ERLIN2	0.185	ER lipid raft associated 2
**Q7Z2K6**	ERMP1	0.14	endoplasmic reticulum metallopeptidase 1
**P30040**	ERP29	0.186	endoplasmic reticulum protein 29
**Q96AP7**	ESAM	0.313	endothelial cell adhesion molecule
**P10768**	ESD	-0.117	esterase D
**B1AK53-1**	ESPN	-0.733	espin
**Q9H6T0**	ESRP2	-0.14	epithelial splicing regulatory protein 2
**Q96DF8**	ESS2	-0.326	ess-2 splicing factor homolog
**A0FGR8-2**	ESYT2	0.176	extended synaptotagmin 2
**P62495**	ETF1	-0.381	eukaryotic translation termination factor 1
**P13804-1**	ETFA	0.205	electron transfer flavoprotein subunit alpha
**P38117**	ETFB	0.328	electron transfer flavoprotein subunit beta
**Q01844-5**	EWSR1	-0.244	EWS RNA binding protein 1
**O60645**	EXOC3	-0.339	exocyst complex component 3
**Q9Y2D4**	EXOC6B	-0.355	exocyst complex component 6B
**Q9UPT5**	EXOC7	-0.094	exocyst complex component 7
**Q9Y2C4**	EXOG	0.165	exo/endonuclease G
**Q01780**	EXOSC10	0.253	exosome component 10
**Q9NQT5**	EXOSC3	0.197	exosome component 3
**Q96B26**	EXOSC8	-0.301	exosome component 8
**P00734**	F2	-0.322	coagulation factor II, thrombin

**P07148**	FABP1	-0.848	fatty acid binding protein 1
**O60427**	FADS1	-0.096	fatty acid desaturase 1
**O95864**	FADS2	-0.378	fatty acid desaturase 2
**Q9UNN5**	FAF1	-0.539	Fas associated factor 1
**Q96CS3**	FAF2	0.262	Fas associated factor family member 2
**P16930**	FAH	-0.37	fumarylacetoacetate hydrolase
**Q6P587-3**	FAHD1	0.105	fumarylacetoacetate hydrolase domain containing 1
**Q9H098-2**	FAM107B	-0.114	family with sequence similarity 107 member B
**Q8IWE2**	FAM114A1	-0.284	family with sequence similarity 114 member A1
**Q9NRY5**	FAM114A2	-0.319	family with sequence similarity 114 member A2
**Q9NZB2-6**	FAM120A	-0.172	family with sequence similarity 120A
**Q96E09**	FAM122A	-0.325	family with sequence similarity 122A
**Q96C01**	FAM136A	-0.237	family with sequence similarity 136 member A
**Q8N128-2**	FAM177A1	0.214	family with sequence similarity 177 member A1
**Q9GZU8**	FAM192A	-0.139	family with sequence similarity 192 member A
**Q9NSI2**	FAM207A	-0.423	family with sequence similarity 207 member A
**Q96ND0**	FAM210A	0.593	family with sequence similarity 210 member A
**Q96KR6**	FAM210B	0.433	family with sequence similarity 210 member B
**Q92520**	FAM3C	0.15	family with sequence similarity 3 member C
**Q9NUQ9**	FAM49B	-0.128	family with sequence similarity 49 member B
**Q9H4H8**	FAM83D	0.297	family with sequence similarity 83 member D
**Q52LJ0-2**	FAM98B	0.212	family with sequence similarity 98 member B
**Q9BXW9-1**	FANCD2	-0.197	FA complementation group D2
**Q9NVI1**	FANCI	-0.261	FA complementation group I
**Q9Y285**	FARSA	-0.185	phenylalanyl-tRNA synthetase subunit alpha
**Q9NSD9**	FARSB	-0.246	phenylalanyl-tRNA synthetase subunit beta
**P25445**	FAS	0.699	Fas cell surface death receptor
**P49327**	FASN	-0.353	fatty acid synthase
**Q9NYY8**	FASTKD2	0.185	FAST kinase domains 2
**Q7L8L6**	FASTKD5	0.862	FAST kinase domains 5
**P22087**	FBL	0.238	fibrillarin
**Q8WUP2-2**	FBLIM1	0.332	filamin binding LIM protein 1
**Q8N531-1**	FBXL6	0.358	F-box and leucine rich repeat protein 6
**Q8TB52**	FBXO30	0.368	F-box protein 30
**Q6PIJ6-1**	FBXO38	0.233	F-box protein 38
**O94868**	FCHSD2	-0.431	FCH and double SH3 domains 2
**P37268**	FDFT1	-0.246	farnesyl-diphosphate farnesyltransferase 1
**P14324**	FDPS	-0.216	farnesyl diphosphate synthase
**P22570-7**	FDXR	0.472	ferredoxin reductase
**P22830-2**	FECH	0.415	ferrochelatase
**P39748**	FEN1	0.086	flap structure-specific endonuclease 1
**P02671-1**	FGA	-0.65	fibrinogen alpha chain
**P02671-2**	FGA	-0.695	fibrinogen alpha chain
**P02675**	FGB	-0.242	fibrinogen beta chain
**O95684**	FGFR1OP	-0.248	FGFR1 oncogene partner
**P22455-1**	FGFR4	-0.606	fibroblast growth factor receptor 4
**P02679**	FGG	-0.429	fibrinogen gamma chain

**Q13643**	FHL3	0.709	four and a half LIM domains 3
**Q9Y613**	FHOD1	-0.236	formin homology 2 domain containing 1
**O43427**	FIBP	-0.404	FGF1 intracellular binding protein
**Q9NWM8**	FKBP14	-0.432	FKBP prolyl isomerase 14
**P62942**	FKBP1A	-0.33	FKBP prolyl isomerase 1A
**Q00688**	FKBP3	-0.161	FKBP prolyl isomerase 3
**Q02790**	FKBP4	0.201	FKBP prolyl isomerase 4
**Q14318-2**	FKBP8	0.118	FKBP prolyl isomerase 8
**O95302-3**	FKBP9	0.147	FKBP prolyl isomerase 9
**P21333**	FLNA	-0.164	filamin A
**O75369-8**	FLNB	-0.313	filamin B
**Q9Y5Y0**	FLVCR1	-0.403	feline leukemia virus subgroup C cellular receptor 1
**Q96CP2**	FLYWCH2	-0.36	FLYWCH family member 2
**Q96PY5-3**	FMNL2	-0.136	formin like 2
**P02751-15**	FN1	-0.635	fibronectin 1
**Q9H479**	FN3K	-0.129	fructosamine 3 kinase
**Q96RU3-1**	FNBP1	-0.564	formin binding protein 1
**Q5T0N5-1**	FNBP1L	-0.188	formin binding protein 1 like
**Q8N3X1-2**	FNBP4	-0.221	formin binding protein 4
**Q9Y2H6**	FNDC3A	0.242	fibronectin type III domain containing 3A
**Q53EP0**	FNDC3B	-0.504	fibronectin type III domain containing 3B
**P49354**	FNTA	-0.235	farnesyltransferase, CAAX box, alpha
**Q5VW36**	FOCAD	-0.32	focadhesin
**P15407**	FOSL1	1.38	FOS like 1, AP-1 transcription factor subunit
**P85037**	FOXK1	0.226	forkhead box K1
**Q8IWF2**	FOXRED2	-0.27	FAD dependent oxidoreductase domain containing 2
**Q86XX4-2**	FRAS1	-1.124	Fraser extracellular matrix complex subunit 1
**Q14331**	FRG1	0.187	FSHD region gene 1
**Q9BZ67**	FRMD8	0.215	FERM domain containing 8
**O95954-1**	FTCD	-0.285	formimidoyltransferase cyclodeaminase
**P02794**	FTH1	0.377	ferritin heavy chain 1
**P02792**	FTL	0.761	ferritin light chain
**Q9C0B1-1**	FTO	-0.463	FTO alpha-ketoglutarate dependent dioxygenase
**Q96AE4**	FUBP1	-0.217	far upstream element binding protein 1
**Q9BTY2**	FUCA2	0.092	alpha-L-fucosidase 2
**P09958**	FURIN	-0.4	furin, paired basic amino acid cleaving enzyme
**P51114**	FXR1	0.136	FMR1 autosomal homolog 1
**P51116**	FXR2	0.22	FMR1 autosomal homolog 2
**Q9BQS8-4**	FYCO1	-0.297	FYVE and coiled-coil domain containing 1
**Q13283**	G3BP1	-0.189	G3BP stress granule assembly factor 1
**P11413-2**	G6PD	-0.114	glucose-6-phosphate dehydrogenase
**O95166**	GABARAP	-0.254	GABA type A receptor-associated protein
**P60520**	GABARAPL2	0.181	GABA type A receptor associated protein like 2
**Q06546**	GABPA	-0.198	GA binding protein transcription factor subunit alpha
**Q06547**	GABPB1	0.247	GA binding protein transcription factor subunit beta 1
**O14976**	GAK	-0.177	cyclin G associated kinase
**Q14376**	GALE	-0.45	UDP-galactose-4-epimerase

**P51570-2**	GALK1	-0.147	galactokinase 1
**Q96C23**	GALM	-0.479	galactose mutarotase
**P34059**	GALNS	-0.134	galactosamine (N-acetyl)-6-sulfatase
**Q10472**	GALNT1	0.167	polypeptide N-acetylgalactosaminyltransferase 1
**P07902**	GALT	-0.206	galactose-1-phosphate uridylyltransferase
**Q14353**	GAMT	-0.258	guanidinoacetate N-methyltransferase
**P04406**	GAPDH	-0.187	glyceraldehyde-3-phosphate dehydrogenase
**Q9NY12**	GAR1	0.274	GAR1 ribonucleoprotein
**P22102**	GART	-0.167	phosphoribosylglycinamide formyltransferase, phosphoribosylglycinamide synthetase, ph[illegible]
**O43903**	GAS2	-0.215	growth arrest specific 2
**Q86YP4-3**	GATAD2A	0.102	GATA zinc finger domain containing 2A
**P04062**	GBA	0.145	glucosylceramidase beta
**Q04446**	GBE1	-0.171	1,4-alpha-glucan branching enzyme 1
**Q92538**	GBF1	-0.189	golgi brefeldin A resistant guanine nucleotide exchange factor 1
**P28676**	GCA	-0.352	grancalcin
**Q96CN9**	GCC1	0.324	GRIP and coiled-coil domain containing 1
**Q8IWJ2**	GCC2	0.131	GRIP and coiled-coil domain containing 2
**Q92947-1**	GCDH	0.183	glutaryl-CoA dehydrogenase
**P16383**	GCFC2	-0.104	GC-rich sequence DNA-binding factor 2
**P30793-1**	GCH1	0.365	GTP cyclohydrolase 1
**P48506**	GCLC	0.189	glutamate-cysteine ligase catalytic subunit
**P48507**	GCLM	1.098	glutamate-cysteine ligase modifier subunit
**Q92616**	GCN1	-0.167	GCN1 activator of EIF2AK4
**Q8TB36**	GDAP1	0.582	ganglioside induced differentiation associated protein 1
**Q9NZC3**	GDE1	0.326	glycerophosphodiester phosphodiesterase 1
**Q99988**	GDF15	1.155	growth differentiation factor 15
**P31150**	GDI1	0.167	GDP dissociation inhibitor 1
**P50395-1**	GDI2	-0.26	GDP dissociation inhibitor 2
**Q8TEQ6**	GEMIN5	-0.082	gem nuclear organelle associated protein 5
**Q7L5D6**	GET4	-0.294	guided entry of tail-anchored proteins factor 4
**Q969S9**	GFM2	0.185	G elongation factor mitochondrial 2
**Q06210**	GFPT1	-0.173	glutamine--fructose-6-phosphate transaminase 1
**Q9UJY5-6**	GGA1	-0.353	golgi associated, gamma adaptin ear containing, ARF binding protein 1
**Q9NZ52**	GGA3	-0.107	golgi associated, gamma adaptin ear containing, ARF binding protein 3
**O75223**	GGCT	-0.333	gamma-glutamylcyclotransferase
**P19440**	GGT1	-0.133	gamma-glutamyltransferase 1
**Q9UJ14**	GGT7	-0.257	gamma-glutamyltransferase 7
**Q9H3K2**	GHITM	0.369	growth hormone inducible transmembrane protein
**Q6Y7W6-1**	GIGYF2	-0.238	GRB10 interacting GYF protein 2
**Q9BRX5**	GINS3	-0.414	GINS complex subunit 3
**O14908-1**	GIPC1	-0.106	GIPC PDZ domain containing family member 1
**Q8TF65**	GIPC2	-0.109	GIPC PDZ domain containing family member 2
**Q9Y2X7-3**	GIT1	0.167	GIT ArfGAP 1
**Q14161-1**	GIT2	-0.166	GIT ArfGAP 2
**P08034**	GJB1	-0.803	gap junction protein beta 1
**P06280**	GLA	0.604	galactosidase alpha
**P23378**	GLDC	0.133	glycine decarboxylase

**Q53GS7-1**	GLE1	0.135	GLE1 RNA export mediator
**Q92896-2**	GLG1	-0.476	golgi glycoprotein 1
**Q92990**	GLMN	-0.189	glomulin, FKBP associated protein
**Q9HC38**	GLOD4	-0.219	glyoxalase domain containing 4
**O76003**	GLRX3	-0.2	glutaredoxin 3
**O94925**	GLS	1.065	glutaminase
**O94925-3**	GLS	0.451	glutaminase
**Q68CQ7-1**	GLT8D1	-0.139	glycosyltransferase 8 domain containing 1
**P00367**	GLUD1	0.267	glutamate dehydrogenase 1
**Q49A26**	GLYR1	0.264	glyoxylate reductase 1 homolog
**O60547**	GMDS	-0.309	GDP-mannose 4,6-dehydratase
**Q9UKD1**	GMEB2	0.239	glucocorticoid modulatory element binding protein 2
**P60983**	GMFB	-0.183	glia maturation factor beta
**O75496**	GMNN	0.18	geminin DNA replication inhibitor
**Q96IJ6**	GMPPA	-0.209	GDP-mannose pyrophosphorylase A
**Q9Y5P6-2**	GMPPB	-0.52	GDP-mannose pyrophosphorylase B
**Q9P2T1**	GMPR2	-0.19	guanosine monophosphate reductase 2
**P49915**	GMPS	-0.201	guanine monophosphate synthase
**P29992**	GNA11	0.14	G protein subunit alpha 11
**Q14344**	GNA13	0.464	G protein subunit alpha 13
**P04899**	GNAI2	0.275	G protein subunit alpha i2
**P08754**	GNAI3	0.187	G protein subunit alpha i3
**P50148**	GNAQ	-0.336	G protein subunit alpha q
**P62873**	GNB1	0.134	G protein subunit beta 1
**P36915**	GNL1	0.156	G protein nucleolar 1 (putative)
**Q9BVP2**	GNL3	-0.552	G protein nucleolar 3
**P46926**	GNPDA1	-0.093	glucosamine-6-phosphate deaminase 1
**Q96EK6**	GNPNAT1	-0.186	glucosamine-phosphate N-acetyltransferase 1
**Q08379**	GOLGA2	0.136	golgin A2
**Q08378**	GOLGA3	-0.21	golgin A3
**Q13439-5**	GOLGA4	-0.148	golgin A4
**Q7Z5G4**	GOLGA7	0.236	golgin A7
**Q9H4A6**	GOLPH3	-0.203	golgi phosphoprotein 3
**Q9H4A5**	GOLPH3L	-0.125	golgi phosphoprotein 3 like
**Q9Y3E0**	GOLT1B	0.481	golgi transport 1B
**Q9H8Y8**	GORASP2	-0.266	golgi reassembly stacking protein 2
**O14653-2**	GOSR2	0.254	golgi SNAP receptor complex member 2
**P00505**	GOT2	0.402	glutamic-oxaloacetic transaminase 2
**Q8IXQ4**	GPALPP1	-0.131	GPALPP motifs containing 1
**Q53EU6**	GPAT3	0.424	glycerol-3-phosphate acyltransferase 3
**Q5T3I0-3**	GPATCH4	-0.334	G-patch domain containing 4
**Q9UKJ3-1**	GPATCH8	-0.161	G-patch domain containing 8
**P35052**	GPC1	0.254	glypican 1
**P51654-1**	GPC3	-0.386	glypican 3
**Q9Y625**	GPC6	-0.582	glypican 6
**P21695**	GPD1	-0.384	glycerol-3-phosphate dehydrogenase 1
**Q9NQX3-2**	GPHN	-0.531	gephyrin

**P06744**	GPI	-0.22	glucose-6-phosphate isomerase
**Q9NQ84-2**	GPRC5C	0.15	G protein-coupled receptor class C group 5 member C
**P18283**	GPX2	-0.354	glutathione peroxidase 2
**P36969**	GPX4	-0.62	glutathione peroxidase 4
**Q14451-3**	GRB7	-0.106	growth factor receptor bound protein 7
**Q9UBQ7**	GRHPR	-0.14	glyoxylate and hydroxypyruvate reductase
**P25098**	GRK2	-0.121	G protein-coupled receptor kinase 2
**Q9HAV7**	GRPEL1	0.227	GrpE like 1, mitochondrial
**Q12849**	GRSF1	0.111	G-rich RNA sequence binding factor 1
**Q14687**	GSE1	-0.181	Gse1 coiled-coil protein
**P00390**	GSR	-0.092	glutathione-disulfide reductase
**P48637**	GSS	-0.216	glutathione synthetase
**P08263**	GSTA1	-0.468	glutathione S-transferase alpha 1
**Q9Y2Q3-1**	GSTK1	0.246	glutathione S-transferase kappa 1
**P21266**	GSTM3	-0.208	glutathione S-transferase mu 3
**P78417**	GSTO1	-0.414	glutathione S-transferase omega 1
**P30711**	GSTT1	-0.215	glutathione S-transferase theta 1
**Q00403**	GTF2B	0.415	general transcription factor IIB
**P35269**	GTF2F1	-0.259	general transcription factor IIF subunit 1
**P13984**	GTF2F2	-0.173	general transcription factor IIF subunit 2
**Q13889**	GTF2H3	0.487	general transcription factor IIH subunit 3
**Q92759**	GTF2H4	0.15	general transcription factor IIH subunit 4
**P78347**	GTF2I	-0.186	general transcription factor IIi
**Q12789**	GTF3C1	0.093	general transcription factor IIIC subunit 1
**Q9UKN8**	GTF3C4	0.091	general transcription factor IIIC subunit 4
**Q9Y5Q8-1**	GTF3C5	0.26	general transcription factor IIIC subunit 5
**A4D1E9**	GTPBP10	0.306	GTP binding protein 10
**Q969Y2-2**	GTPBP3	0.134	GTP binding protein 3, mitochondrial
**Q9NYZ3**	GTSE1	0.388	G2 and S-phase expressed 1
**Q8N442**	GUF1	0.281	GUF1 homolog, GTPase
**Q4G148**	GXYLT1	0.249	glucoside xylosyltransferase 1
**Q9H116**	GZF1	0.227	GDNF inducible zinc finger protein 1
**P07305**	H1-0	1.066	H1.0 linker histone
**P16401**	H1-5	0.876	H1.5 linker histone, cluster member
**P16104**	H2AX	0.377	H2A.X variant histone
**P0C0S5**	H2AZ1	0.771	H2A.Z variant histone 1
**O95479**	H6PD	-0.141	hexose-6-phosphate dehydrogenase/glucose 1-dehydrogenase
**P46952**	HAAO	-0.208	3-hydroxyanthranilate 3,4-dioxygenase
**Q5JVS0**	HABP4	-0.292	hyaluronan binding protein 4
**Q6Y1H2**	HACD2	0.395	3-hydroxyacyl-CoA dehydratase 2
**Q9P035**	HACD3	0.159	3-hydroxyacyl-CoA dehydratase 3
**Q9UJ83**	HACL1	0.135	2-hydroxyacyl-CoA lyase 1
**P40939**	HADHA	0.333	hydroxyacyl-CoA dehydrogenase trifunctional multienzyme complex subunit alpha
**P55084**	HADHB	0.319	hydroxyacyl-CoA dehydrogenase trifunctional multienzyme complex subunit beta
**P12081**	HARS1	-0.237	histidyl-tRNA synthetase 1
**O14929**	HAT1	-0.08	histone acetyltransferase 1
**O94927**	HAUS5	-0.202	HAUS augmin like complex subunit 5

**Q9Y450**	HBS1L	-0.162	HBS1 like translational GTPase
**P53701**	HCCS	0.261	holocytochrome c synthase
**Q92769**	HDAC2	0.122	histone deacetylase 2
**Q7Z4H3**	HDDC2	-0.206	HD domain containing 2
**Q8N4P3**	HDDC3	-0.249	HD domain containing 3
**P51858**	HDGF	-0.32	heparin binding growth factor
**Q9H0R4**	HDHD2	-0.163	haloacid dehalogenase like hydrolase domain containing 2
**Q00341**	HDLBP	-0.555	high density lipoprotein binding protein
**Q9H583**	HEATR1	0.202	HEAT repeat containing 1
**Q7Z4Q2**	HEATR3	-0.376	HEAT repeat containing 3
**Q86XA9**	HEATR5A	-0.35	HEAT repeat containing 5A
**Q9P2D3**	HEATR5B	-0.306	HEAT repeat containing 5B
**Q9Y5Z4**	HEBP2	-0.387	heme binding protein 2
**Q9BYK8**	HELZ2	0.264	helicase with zinc finger 2
**Q15011-1**	HERPUD1	-0.686	homocysteine inducible ER protein with ubiquitin like domain 1
**P07686**	HEXB	0.16	hexosaminidase subunit beta
**Q96MH2**	HEXIM2	-0.25	HEXIM P-TEFb complex subunit 2
**Q93099**	HGD	-0.177	homogentisate 1,2-dioxygenase
**Q9BTY7**	HGH1	-0.146	HGH1 homolog
**P31937**	HIBADH	0.275	3-hydroxyisobutyrate dehydrogenase
**Q6NVY1**	HIBCH	0.155	3-hydroxyisobutyryl-CoA hydrolase
**Q9NWT6**	HIF1AN	-0.281	hypoxia inducible factor 1 subunit alpha inhibitor
**P49773**	HINT1	-0.096	histidine triad nucleotide binding protein 1
**Q9BX68**	HINT2	0.42	histidine triad nucleotide binding protein 2
**Q9NQE9**	HINT3	-0.154	histidine triad nucleotide binding protein 3
**Q9BW71-1**	HIRIP3	-0.192	HIRA interacting protein 3
**P52789**	HK2	-0.147	hexokinase 2
**Q2TB90-1**	HKDC1	0.24	hexokinase domain containing 1
**P01892**	HLA-A	0.356	major histocompatibility complex, class I, A
**P05534**	HLA-A	0.389	major histocompatibility complex, class I, A
**Q14527**	HLTF	-0.356	helicase like transcription factor
**P08397**	HMBS	-0.298	hydroxymethylbilane synthase
**Q96FZ2**	HMCES	-0.221	5-hydroxymethylcytosine binding, ES cell specific
**P17096-1**	HMGA1	0.419	high mobility group AT-hook 1
**P52926-1**	HMGA2	0.401	high mobility group AT-hook 2
**P09429**	HMGB1	0.199	high mobility group box 1
**P35914**	HMGCL	0.218	3-hydroxy-3-methylglutaryl-CoA lyase
**Q01581**	HMGCS1	-0.494	3-hydroxy-3-methylglutaryl-CoA synthase 1
**P05114**	HMGN1	0.317	high mobility group nucleosome binding domain 1
**P05204**	HMGN2	0.991	high mobility group nucleosomal binding domain 2
**Q9UGU5**	HMGXB4	0.234	HMG-box containing 4
**O75330-3**	HMMR	0.437	hyaluronan mediated motility receptor
**P09601**	HMOX1	1.422	heme oxygenase 1
**P30519**	HMOX2	0.159	heme oxygenase 2
**P20823-7**	HNF1A	-0.236	HNF1 homeobox A
**P41235-4**	HNF4A	-0.58	hepatocyte nuclear factor 4 alpha
**P50135**	HNMT	-0.327	histamine N-methyltransferase

**Q13151**	HNRNPA0	0.226	heterogeneous nuclear ribonucleoprotein A0
**Q14103-3**	HNRNPD	-0.184	heterogeneous nuclear ribonucleoprotein D
**O14979-1**	HNRNPDL	-0.227	heterogeneous nuclear ribonucleoprotein D like
**P55795**	HNRNPH2	0.181	heterogeneous nuclear ribonucleoprotein H2
**P14866**	HNRNPL	0.16	heterogeneous nuclear ribonucleoprotein L
**Q00839**	HNRNPU	0.235	heterogeneous nuclear ribonucleoprotein U
**Q9NSC5**	HOMER3	-0.191	homer scaffold protein 3
**Q9UJC3**	HOOK1	-0.228	hook microtubule tethering protein 1
**P00738**	HP	-0.722	haptoglobin
**Q5SSJ5**	HP1BP3	0.474	heterochromatin protein 1 binding protein 3
**P37235**	HPCAL1	-0.325	hippocalcin like 1
**P32754-1**	HPD	-0.314	4-hydroxyphenylpyruvate dioxygenase
**Q9NWY4**	HPF1	-0.223	histone PARylation factor 1
**P00492**	HPRT1	-0.144	hypoxanthine phosphoribosyltransferase 1
**O75506**	HSBP1	-0.346	heat shock factor binding protein 1
**Q8IWL3**	HSCB	0.202	HscB mitochondrial iron-sulfur cluster cochaperone
**Q99714-1**	HSD17B10	0.18	hydroxysteroid 17-beta dehydrogenase 10
**Q53GQ0**	HSD17B12	0.158	hydroxysteroid 17-beta dehydrogenase 12
**P51659-1**	HSD17B4	0.232	hydroxysteroid 17-beta dehydrogenase 4
**Q3SXM5**	HSDL1	0.268	hydroxysteroid dehydrogenase like 1
**Q6YN16**	HSDL2	0.177	hydroxysteroid dehydrogenase like 2
**P07900**	HSP90AA1	0.197	heat shock protein 90 alpha family class A member 1
**P08238**	HSP90AB1	0.133	heat shock protein 90 alpha family class B member 1
**P14625**	HSP90B1	0.27	heat shock protein 90 beta family member 1
**P48723**	HSPA13	0.184	heat shock protein family A (Hsp70) member 13
**Q0VDF9**	HSPA14	-0.197	heat shock protein family A (Hsp70) member 14
**P0DMV9**	HSPA1A/HSPA1B	1.605	heat shock protein family A (Hsp70) member 1A
**O95757**	HSPA4L	0.137	heat shock protein family A (Hsp70) member 4 like
**P17066**	HSPA6	2.196	heat shock protein family A (Hsp70) member 6
**P38646**	HSPA9	0.294	heat shock protein family A (Hsp70) member 9
**P04792**	HSPB1	0.996	heat shock protein family B (small) member 1
**Q9NZL4-3**	HSPBP1	-0.252	HSPA (Hsp70) binding protein 1
**P10809**	HSPD1	0.364	heat shock protein family D (Hsp60) member 1
**P61604**	HSPE1	0.572	heat shock protein family E (Hsp10) member 1
**Q92598**	HSPH1	0.838	heat shock protein family H (Hsp110) member 1
**P42858**	HTT	-0.355	huntingtin
**Q7Z6Z7**	HUWE1	-0.246	HECT, UBA and WWE domain containing E3 ubiquitin protein ligase 1
**Q9NX55-2**	HYPK	-0.168	huntingtin interacting protein K
**Q2TAA2**	IAH1	-0.136	isoamyl acetate hydrolyzing esterase 1 (putative)
**P41252**	IARS1	-0.125	isoleucyl-tRNA synthetase 1
**Q9NSE4**	IARS2	0.349	isoleucyl-tRNA synthetase 2, mitochondrial
**Q5T440**	IBA57	0.341	iron-sulfur cluster assembly factor IBA57
**Q9P2D0**	IBTK	-0.227	inhibitor of Bruton tyrosine kinase
**P14735**	IDE	-0.192	insulin degrading enzyme
**O75874**	IDH1	-0.332	isocitrate dehydrogenase (NADP(+)) 1
**P48735**	IDH2	0.275	isocitrate dehydrogenase (NADP(+)) 2
**P50213**	IDH3A	0.202	isocitrate dehydrogenase (NAD(+)) 3 catalytic subunit alpha

**O43837**	IDH3B	0.232	isocitrate dehydrogenase (NAD(+)) 3 non-catalytic subunit beta
**P51553-1**	IDH3G	0.282	isocitrate dehydrogenase (NAD(+)) 3 non-catalytic subunit gamma
**Q13907**	IDI1	-0.17	isopentenyl-diphosphate delta isomerase 1
**Q9BYX4-1**	IFIH1	-0.344	interferon induced with helicase C domain 1
**O00425**	IGF2BP3	-0.098	insulin like growth factor 2 mRNA binding protein 3
**P11717**	IGF2R	-0.342	insulin like growth factor 2 receptor
**P08833**	IGFBP1	0.545	insulin like growth factor binding protein 1
**Q8N6C5-4**	IGSF1	-0.326	immunoglobulin superfamily member 1
**Q13123**	IK	0.171	IK cytokine
**Q70UQ0**	IKBIP	0.27	IKBKB interacting protein
**Q70UQ0-4**	IKBIP	0.365	IKBKB interacting protein
**Q9Y6K9-2**	IKBKG	0.139	inhibitor of nuclear factor kappa B kinase regulatory subunit gamma
**Q14116**	IL18	0.218	interleukin 18
**Q9NPH3**	IL1RAP	-0.207	interleukin 1 receptor accessory protein
**P40189**	IL6ST	-0.307	interleukin 6 signal transducer
**Q13418**	ILK	0.122	integrin linked kinase
**Q9H0C8**	ILKAP	-0.19	ILK associated serine/threonine phosphatase
**Q9NV31**	IMP3	0.182	IMP U3 small nucleolar ribonucleoprotein 3
**Q96G21**	IMP4	0.282	IMP U3 small nucleolar ribonucleoprotein 4
**Q9NX62**	IMPAD1	0.143	inositol monophosphatase domain containing 1
**Q9NQS7**	INCENP	0.477	inner centromere protein
**Q27J81**	INF2	-0.204	inverted formin, FH2 and WH2 domain containing
**Q9UK53-1**	ING1	0.307	inhibitor of growth family member 1
**Q92835**	INPP5D	-0.343	inositol polyphosphate-5-phosphatase D
**P06213**	INSR	-0.346	insulin receptor
**Q9NVM9**	INTS13	-0.189	integrator complex subunit 13
**Q96SY0**	INTS14	-0.195	integrator complex subunit 14
**Q96HW7**	INTS4	-0.092	integrator complex subunit 4
**Q9NV88-1**	INTS9	-0.121	integrator complex subunit 9
**Q9UI26-2**	IPO11	-0.272	importin 11
**Q8TEX9-2**	IPO4	-0.445	importin 4
**O00410-3**	IPO5	-0.466	importin 5
**O95373**	IPO7	-0.356	importin 7
**Q96P70**	IPO9	-0.299	importin 9
**P46940**	IQGAP1	-0.095	IQ motif containing GTPase activating protein 1
**Q13576-1**	IQGAP2	-0.253	IQ motif containing GTPase activating protein 2
**Q6DN90**	IQSEC1	-0.291	IQ motif and Sec7 domain ArfGEF 1
**P48200**	IREB2	-0.149	iron responsive element binding protein 2
**Q9H1B7**	IRF2BPL	0.164	interferon regulatory factor 2 binding protein like
**Q14653**	IRF3	-0.184	interferon regulatory factor 3
**Q8WZA9**	IRGQ	-0.151	immunity related GTPase Q
**Q86U28**	ISCA2	-0.228	iron-sulfur cluster assembly 2
**Q9H1K1**	ISCU	0.337	iron-sulfur cluster assembly enzyme
**P05161**	ISG15	-0.108	ISG15 ubiquitin like modifier
**Q9H9L3**	ISG20L2	-0.105	interferon stimulated exonuclease gene 20 like 2
**Q96CN7**	ISOC1	-0.127	isochorismatase domain containing 1
**Q96AB3-2**	ISOC2	0.198	isochorismatase domain containing 2

**P53990-1**	IST1	0.104	IST1 factor associated with ESCRT-III
**Q9ULR0-1**	ISY1	0.205	ISY1 splicing factor homolog
**P56199**	ITGA1	-0.131	integrin subunit alpha 1
**P23229-1**	ITGA6	-0.336	integrin subunit alpha 6
**P23229-6**	ITGA6	-0.426	integrin subunit alpha 6
**P19823**	ITIH2	-0.103	inter-alpha-trypsin inhibitor heavy chain 2
**Q9NQX7**	ITM2C	-0.166	integral membrane protein 2C
**Q9BY32**	ITPA	-0.283	inosine triphosphatase
**P28290**	ITPRID2	0.159	ITPR interacting domain containing 2
**P26440**	IVD	0.202	isovaleryl-CoA dehydrogenase
**Q96ST2**	IWS1	-0.209	interacts with SUPT6H, CTD assembly factor 1
**Q6IE81-1**	JADE1	-0.184	jade family PHD finger 1
**P78504**	JAG1	0.114	jagged canonical Notch ligand 1
**P23458**	JAK1	-0.342	Janus kinase 1
**Q9UK76**	JPT1	-0.184	Jupiter microtubule associated homolog 1
**Q9UK76-2**	JPT1	-0.353	Jupiter microtubule associated homolog 1
**P17275**	JUNB	0.907	JunB proto-oncogene, AP-1 transcription factor subunit
**P14923**	JUP	0.081	junction plakoglobin
**Q63ZY3-2**	KANK2	-0.353	KN motif and ankyrin repeat domains 2
**Q15046-2**	KARS1	-0.106	lysyl-tRNA synthetase 1
**Q9H7Z6-2**	KAT8	-0.175	lysine acetyltransferase 8
**Q86V97**	KBTBD6	0.195	kelch repeat and BTB domain containing 6
**Q9P0J7**	KCMF1	0.195	potassium channel modulatory factor 1
**Q9Y597**	KCTD3	-0.421	potassium channel tetramerization domain containing 3
**Q9NXV2**	KCTD5	0.349	potassium channel tetramerization domain containing 5
**Q7L273**	KCTD9	0.351	potassium channel tetramerization domain containing 9
**Q9Y2K7**	KDM2A	0.379	lysine demethylase 2A
**Q9Y4C1**	KDM3A	0.703	lysine demethylase 3A
**Q7LBC6-1**	KDM3B	-0.138	lysine demethylase 3B
**O75164**	KDM4A	-0.309	lysine demethylase 4A
**Q14145**	KEAP1	0.174	kelch like ECH associated protein 1
**Q7Z7F0-1**	KHDC4	-0.214	KH domain containing 4, pre-mRNA splicing factor
**P50053-2**	KHK	-0.445	ketohexokinase
**Q5T5P2-1**	KIAA1217	-0.183	KIAA1217
**Q9BY89-1**	KIAA1671	-0.313	KIAA1671
**Q9ULH0-1**	KIDINS220	-0.249	kinase D interacting substrate 220
**Q9NQT8**	KIF13B	-0.307	kinesin family member 13B
**Q9NS87**	KIF15	-0.205	kinesin family member 15
**Q96L93-2**	KIF16B	-0.296	kinesin family member 16B
**O60333-4**	KIF1B	-0.289	kinesin family member 1B
**O43896**	KIF1C	-0.168	kinesin family member 1C
**O95235**	KIF20A	0.264	kinesin family member 20A
**Q96Q89-2**	KIF20B	0.297	kinesin family member 20B
**Q14807**	KIF22	0.258	kinesin family member 22
**Q02241-1**	KIF23	0.175	kinesin family member 23
**O00139-2**	KIF2A	0.279	kinesin family member 2A
**Q99661**	KIF2C	0.306	kinesin family member 2C

**O95239-1**	KIF4A	-0.11	kinesin family member 4A
**P33176**	KIF5B	-0.144	kinesin family member 5B
**Q92845**	KIFAP3	-0.31	kinesin associated protein 3
**Q9BW19**	KIFC1	0.201	kinesin family member C1
**Q86Z14**	KLB	-0.392	klotho beta
**Q9NSK0-3**	KLC4	-0.248	kinesin light chain 4
**Q9BXK1**	KLF16	-0.213	Kruppel like factor 16
**Q9NVR0**	KLHL11	0.359	kelch like family member 11
**Q1ED39**	KNOP1	0.192	lysine rich nucleolar protein 1
**P50748**	KNTC1	-0.216	kinetochore associated 1
**P52292**	KPNA2	0.223	karyopherin subunit alpha 2
**O00505**	KPNA3	-0.174	karyopherin subunit alpha 3
**Q14974**	KPNB1	-0.336	karyopherin subunit beta 1
**Q8N9T8**	KRI1	0.144	KRI1 homolog
**Q13601**	KRR1	0.248	KRR1 small subunit processome component homolog
**P05783**	KRT18	0.181	keratin 18
**P08727**	KRT19	0.406	keratin 19
**P05787-2**	KRT8	0.202	keratin 8
**Q86UP2**	KTN1	0.129	kinectin 1
**Q16719**	KYNU	-0.088	kynureninase
**Q9H9P8-1**	L2HGDH	0.27	L-2-hydroxyglutarate dehydrogenase
**P83111-1**	LACTB	0.408	lactamase beta
**O15230**	LAMA5	-0.52	laminin subunit alpha 5
**P55268**	LAMB2	-0.381	laminin subunit beta 2
**P11047**	LAMC1	-0.139	laminin subunit gamma 1
**P13473-3**	LAMP2	-0.313	lysosomal associated membrane protein 2
**O43813**	LANCL1	-0.453	LanC like 1
**Q6PKG0-1**	LARP1	-0.146	La ribonucleoprotein domain family member 1
**Q659C4**	LARP1B	-0.324	La ribonucleoprotein domain family member 1B
**Q71RC2-4**	LARP4	0.108	La ribonucleoprotein domain family member 4
**Q92615**	LARP4B	0.134	La ribonucleoprotein domain family member 4B
**Q4G0J3-3**	LARP7	-0.148	La ribonucleoprotein domain family member 7
**Q15031**	LARS2	0.326	leucyl-tRNA synthetase 2, mitochondrial
**Q14847**	LASP1	-0.094	LIM and SH3 protein 1
**Q14739**	LBR	0.403	lamin B receptor
**Q96JN0-3**	LCOR	0.553	ligand dependent nuclear receptor corepressor
**Q9H6V9-2**	LDAH	-0.267	lipid droplet associated hydrolase
**P00338-3**	LDHA	-0.466	lactate dehydrogenase A
**P01130**	LDLR	-0.232	low density lipoprotein receptor
**Q9Y2U8**	LEMD3	0.32	LEM domain containing 3
**Q96PV6-2**	LENG8	0.203	leukocyte receptor cluster member 8
**O95202**	LETM1	0.297	leucine zipper and EF-hand containing transmembrane protein 1
**P09382**	LGALS1	0.147	galectin 1
**P17931**	LGALS3	-0.182	galectin 3
**O00214-2**	LGALS8	0.129	galectin 8
**Q99538**	LGMN	0.287	legumain
**P18858**	LIG1	-0.268	DNA ligase 1

**Q9NUP9**	LIN7C	-0.134	lin-7 homolog C, crumbs cell polarity complex component
**P38571**	LIPA	-0.315	lipase A, lysosomal acid type
**Q9BRT6**	LLPH	-0.514	LLP homolog, long-term synaptic facilitation factor
**Q9BU23-1**	LMF2	0.176	lipase maturation factor 2
**Q03252**	LMNB2	0.163	lamin B2
**Q9UIQ6**	LNPEP	-0.208	leucyl and cystinyl aminopeptidase
**P36776**	LONP1	0.282	lon peptidase 1, mitochondrial
**Q92604**	LPGAT1	0.155	lysophosphatidylglycerol acyltransferase 1
**Q93052**	LPP	-0.281	LIM domain containing preferred translocation partner in lipoma
**P50851-2**	LRBA	-0.276	LPS responsive beige-like anchor protein
**Q96II8-2**	LRCH3	-0.315	leucine rich repeats and calponin homology domain containing 3
**Q5T3J3**	LRIF1	0.487	ligand dependent nuclear receptor interacting factor 1
**Q7Z4F1**	LRP10	0.859	LDL receptor related protein 10
**P42704**	LRPPRC	0.329	leucine rich pentatricopeptide repeat containing
**Q9H9A6**	LRRC40	-0.181	leucine rich repeat containing 40
**Q15345-2**	LRRC41	0.275	leucine rich repeat containing 41
**Q8N1G4**	LRRC47	-0.336	leucine rich repeat containing 47
**Q96AG4**	LRRC59	0.275	leucine rich repeat containing 59
**Q8IWT6**	LRRC8A	0.404	leucine rich repeat containing 8 VRAC subunit A
**Q32MZ4-3**	LRRFIP1	-0.101	LRR binding FLII interacting protein 1
**Q9UFC0**	LRWD1	0.133	leucine rich repeats and WD repeat domain containing 1
**Q9H089**	LSG1	0.284	large 60S subunit nuclear export GTPase 1
**O15116**	LSM1	-0.103	LSM1 homolog, mRNA degradation associated
**Q9Y333**	LSM2	-0.159	LSM2 homolog, U6 small nuclear RNA and mRNA degradation associated
**Q9Y4Z0**	LSM4	0.231	LSM4 homolog, U6 small nuclear RNA and mRNA degradation associated
**Q9Y4Y9**	LSM5	-0.18	LSM5 homolog, U6 small nuclear RNA and mRNA degradation associated
**P48449**	LSS	-0.152	lanosterol synthase
**P09960-1**	LTA4H	-0.34	leukotriene A4 hydrolase
**O94822-3**	LTN1	-0.166	listerin E3 ubiquitin protein ligase 1
**Q96GA3**	LTV1	-0.245	LTV1 ribosome biogenesis factor
**Q9NQ29**	LUC7L	0.231	LUC7 like
**Q9Y383**	LUC7L2	0.144	LUC7 like 2, pre-mRNA splicing factor
**Q86V48**	LUZP1	0.295	leucine zipper protein 1
**O75608**	LYPLA1	-0.124	lysophospholipase 1
**O95372**	LYPLA2	-0.109	lysophospholipase 2
**Q9HD34**	LYRM4	0.259	LYR motif containing 4
**Q8WZA0-2**	LZIC	-0.371	leucine zipper and CTNNBIP1 domain containing
**Q9NQ48**	LZTFL1	-0.472	leucine zipper transcription factor like 1
**Q9UPN3**	MACF1	-0.287	microtubule actin crosslinking factor 1
**O75367**	MACROH2A1	0.552	macroH2A.1 histone
**Q9P0M6**	MACROH2A2	0.292	macroH2A.2 histone
**Q9Y6D9**	MAD1L1	-0.233	mitotic arrest deficient 1 like 1
**Q15013-3**	MAD2L1BP	0.509	MAD2L1 binding protein
**Q8WXG6-4**	MADD	0.288	MAP kinase activating death domain
**Q7L5Y9-1**	MAEA	0.132	macrophage erythroblast attacher
**Q9ULX9**	MAFF	1.243	MAF bZIP transcription factor F
**O15525**	MAFG	1.206	MAF bZIP transcription factor G

**Q9Y5V3-2**	MAGED1	-0.277	MAGE family member D1
**Q96QZ7**	MAGI1	-0.37	membrane associated guanylate kinase, WW and PDZ domain containing 1
**Q9H0U3**	MAGT1	0.329	magnesium transporter 1
**Q8WWC4**	MAIP1	0.344	matrix AAA peptidase interacting protein 1
**Q9BXY0**	MAK16	0.254	MAK16 homolog
**Q96EH3**	MALSU1	-0.124	mitochondrial assembly of ribosomal large subunit 1
**Q9UDY8**	MALT1	-0.25	MALT1 paracaspase
**P33908**	MAN1A1	-0.284	mannosidase alpha class 1A member 1
**O00754**	MAN2B1	0.133	mannosidase alpha class 2B member 1
**O00462**	MANBA	-0.363	mannosidase beta
**P55145**	MANF	-0.208	mesencephalic astrocyte derived neurotrophic factor
**P27338**	MAOB	0.229	monoamine oxidase B
**A6NCE7**	MAP1LC3B2	1.462	microtubule associated protein 1 light chain 3 beta 2
**Q66K74**	MAP1S	0.25	microtubule associated protein 1S
**Q02750**	MAP2K1	-0.321	mitogen-activated protein kinase kinase 1
**P36507**	MAP2K2	-0.128	mitogen-activated protein kinase kinase 2
**Q9NYL2**	MAP3K20	-0.383	mitogen-activated protein kinase kinase kinase 20
**Q9Y6R4-1**	MAP3K4	-0.399	mitogen-activated protein kinase kinase kinase 4
**P27816-1**	MAP4	-0.417	microtubule associated protein 4
**Q8IVH8**	MAP4K3	-0.15	mitogen-activated protein kinase kinase kinase kinase 3
**Q3KQU3**	MAP7D1	0.31	MAP7 domain containing 1
**P28482**	MAPK1	-0.349	mitogen-activated protein kinase 1
**P27361-1**	MAPK3	-0.156	mitogen-activated protein kinase 3
**Q16644**	MAPKAPK3	-0.18	MAPK activated protein kinase 3
**Q15691**	MAPRE1	0.147	microtubule associated protein RP/EB family member 1
**Q5VT66-2**	MARC1	-0.109	mitochondrial amidoxime reducing component 1
**P29966**	MARCKS	-0.271	myristoylated alanine rich protein kinase C substrate
**P49006**	MARCKSL1	0.178	MARCKS like 1
**Q96GW9**	MARS2	0.352	methionyl-tRNA synthetase 2, mitochondrial
**Q00266**	MAT1A	-0.323	methionine adenosyltransferase 1A
**Q9NZL9**	MAT2B	-0.201	methionine adenosyltransferase 2B
**Q7Z434**	MAVS	-0.112	mitochondrial antiviral signaling protein
**P61244**	MAX	-0.15	MYC associated factor X
**P56270-2**	MAZ	0.213	MYC associated zinc finger protein
**O95983-1**	MBD3	0.093	methyl-CpG binding domain protein 3
**Q96N66-1**	MBOAT7	0.318	membrane bound O-acyltransferase domain containing 7
**P43121**	MCAM	0.361	melanoma cell adhesion molecule
**Q8IVS2**	MCAT	0.223	malonyl-CoA-acyl carrier protein transacylase
**Q96RQ3**	MCCC1	0.159	methylcrotonoyl-CoA carboxylase 1
**Q9HCC0-1**	MCCC2	0.182	methylcrotonoyl-CoA carboxylase 2
**Q8NI22**	MCFD2	-0.742	multiple coagulation factor deficiency 2
**P49736**	MCM2	-0.266	minichromosome maintenance complex component 2
**P25205-2**	MCM3	-0.179	minichromosome maintenance complex component 3
**P33991**	MCM4	-0.19	minichromosome maintenance complex component 4
**P33992**	MCM5	-0.203	minichromosome maintenance complex component 5
**Q14566**	MCM6	-0.234	minichromosome maintenance complex component 6
**P33993-1**	MCM7	-0.075	minichromosome maintenance complex component 7

**Q9BTE3-1**	MCMBP	-0.198	minichromosome maintenance complex binding protein
**C9JLW8**	MCRIP1	-0.285	MAPK regulated corepressor interacting protein 1
**Q8NE86**	MCU	0.352	mitochondrial calcium uniporter
**P40925-3**	MDH1	-0.256	malate dehydrogenase 1
**P40926**	MDH2	0.306	malate dehydrogenase 2
**Q00987-11**	MDM2	0.55	MDM2 proto-oncogene
**P23368**	ME2	0.342	malic enzyme 2
**Q9HAF1-3**	MEAF6	0.442	MYST/Esa1 associated factor 6
**P51608-2**	MECP2	0.3	methyl-CpG binding protein 2
**Q93074-2**	MED12	0.15	mediator complex subunit 12
**O60244**	MED14	0.239	mediator complex subunit 14
**Q9NVC6**	MED17	0.125	mediator complex subunit 17
**Q71SY5-3**	MED25	0.183	mediator complex subunit 25
**Q96G25-2**	MED8	-0.232	mediator complex subunit 8
**Q9Y316-3**	MEMO1	-0.26	mediator of cell motility 1
**Q7L2J0-1**	MEPCE	0.219	methylphosphate capping enzyme
**Q14696**	MESD	-0.249	mesoderm development LRP chaperone
**P08581-1**	MET	-0.417	MET proto-oncogene, receptor tyrosine kinase
**P08581-2**	MET	-0.472	MET proto-oncogene, receptor tyrosine kinase
**P53582**	METAP1	-0.136	methionyl aminopeptidase 1
**Q9UBP6**	METTL1	-0.231	methyltransferase like 1
**Q86W50-1**	METTL16	-0.192	methyltransferase like 16
**Q9H7H0-3**	METTL17	0.169	methyltransferase like 17
**Q6P1Q9-1**	METTL2B	-0.142	methyltransferase like 2B
**Q86U44**	METTL3	-0.179	methyltransferase like 3
**Q9NRN9**	METTL5	0.193	methyltransferase like 5
**B3KW44**	METTL8	0.307	methyltransferase like 8
**Q8IWA4**	MFN1	0.138	mitofusin 1
**O95140**	MFN2	0.247	mitofusin 2
**Q8IWI9-4**	MGA	-0.221	MAX dimerization protein MGA
**P26572**	MGAT1	0.307	mannosyl (alpha-1,3-)-glycoprotein beta-1,2-N-acetylglucosaminyltransferase
**Q10469**	MGAT2	0.16	mannosyl (alpha-1,6-)-glycoprotein beta-1,2-N-acetylglucosaminyltransferase
**Q9UQ53-3**	MGAT4B	0.425	alpha-1,3-mannosyl-glycoprotein 4-beta-N-acetylglucosaminyltransferase B
**Q09328**	MGAT5	-0.405	alpha-1,6-mannosylglycoprotein 6-beta-N-acetylglucosaminyltransferase
**Q9BQP7**	MGME1	0.363	mitochondrial genome maintenance exonuclease 1
**P16455**	MGMT	-0.241	O-6-methylguanine-DNA methyltransferase
**O60291-2**	MGRN1	-0.329	mahogunin ring finger 1
**P10620**	MGST1	0.286	microsomal glutathione S-transferase 1
**Q99735**	MGST2	0.09	microsomal glutathione S-transferase 2
**Q7RTP6**	MICAL3	-0.399	microtubule associated monooxygenase, calponin and LIM domain containing 3
**Q8N3F8**	MICALL1	0.216	MICAL like 1
**Q9NPA3**	MID1IP1	-0.397	MID1 interacting protein 1
**L0R8F8**	MIEF1	-0.148	mitochondrial elongation factor 1
**Q9BRT3**	MIEN1	-0.612	migration and invasion enhancer 1
**Q8N108-12**	MIER1	-0.302	MIER1 transcriptional regulator
**Q9UNW1**	MINPP1	-0.129	multiple inositol-polyphosphate phosphatase 1
**Q9NXC5-1**	MIOS	-0.242	meiosis regulator for oocyte development

**Q99797**	MIPEP	0.226	mitochondrial intermediate peptidase
**Q8IVT2**	MISP	0.205	mitotic spindle positioning
**P46013**	MKI67	0.401	marker of proliferation Ki-67
**Q14165**	MLEC	0.262	malectin
**Q15773**	MLF2	0.418	myeloid leukemia factor 2
**P40692-1**	MLH1	-0.154	mutL homolog 1
**P51511**	MMP15	-0.544	matrix metallopeptidase 15
**Q96T76-8**	MMS19	-0.266	MMS19 homolog, cytosolic iron-sulfur assembly component
**P22033**	MMUT	0.197	methylmalonyl-CoA mutase
**P51948-1**	MNAT1	0.172	MNAT1 component of CDK activating kinase
**Q7L9L4-2**	MOB1B	0.094	MOB kinase activator 1B
**Q96EN8**	MOCOS	-0.152	molybdenum cofactor sulfurase
**O96007**	MOCS2	-0.221	molybdenum cofactor synthesis 2
**Q7Z3U7**	MON2	-0.311	MON2 homolog, regulator of endosome-to-Golgi trafficking
**Q9Y6X9**	MORC2	0.135	MORC family CW-type zinc finger 2
**Q15014**	MORF4L2	-0.243	mortality factor 4 like 2
**Q8NHP6**	MOSPD2	0.101	motile sperm domain containing 2
**Q9HCE1**	MOV10	-0.133	Mov10 RISC complex RNA helicase
**Q9Y5U8**	MPC1	0.555	mitochondrial pyruvate carrier 1
**O95563**	MPC2	0.213	mitochondrial pyruvate carrier 2
**Q99549-2**	MPHOSPH8	0.302	M-phase phosphoprotein 8
**P34949**	MPI	-0.206	mannose phosphate isomerase
**Q8N3R9-1**	MPP5	-0.183	membrane palmitoylated protein 5
**Q6WCQ1-2**	MPRIP	0.203	myosin phosphatase Rho interacting protein
**P25325-2**	MPST	-0.134	mercaptopyruvate sulfurtransferase
**Q9Y605**	MRFAP1	-0.345	Morf4 family associated protein 1
**Q9NV56**	MRGBP	0.181	MRG domain binding protein
**Q9HC36**	MRM3	0.451	mitochondrial rRNA methyltransferase 3
**Q9BYD6**	MRPL1	0.16	mitochondrial ribosomal protein L1
**Q9Y3B7-1**	MRPL11	0.159	mitochondrial ribosomal protein L11
**P52815**	MRPL12	0.214	mitochondrial ribosomal protein L12
**Q9BYD1**	MRPL13	0.293	mitochondrial ribosomal protein L13
**Q6P1L8**	MRPL14	0.363	mitochondrial ribosomal protein L14
**Q9P015**	MRPL15	0.362	mitochondrial ribosomal protein L15
**Q9NX20**	MRPL16	0.533	mitochondrial ribosomal protein L16
**Q9NRX2**	MRPL17	0.25	mitochondrial ribosomal protein L17
**Q9H0U6**	MRPL18	0.235	mitochondrial ribosomal protein L18
**P49406**	MRPL19	0.171	mitochondrial ribosomal protein L19
**Q5T653**	MRPL2	0.266	mitochondrial ribosomal protein L2
**Q9BYC9**	MRPL20	0.279	mitochondrial ribosomal protein L20
**Q7Z2W9**	MRPL21	0.434	mitochondrial ribosomal protein L21
**Q9NWU5**	MRPL22	0.22	mitochondrial ribosomal protein L22
**Q16540**	MRPL23	0.228	mitochondrial ribosomal protein L23
**Q96A35**	MRPL24	0.188	mitochondrial ribosomal protein L24
**Q9P0M9**	MRPL27	0.472	mitochondrial ribosomal protein L27
**Q13084**	MRPL28	0.289	mitochondrial ribosomal protein L28
**P09001**	MRPL3	0.244	mitochondrial ribosomal protein L3

**Q8TCC3-2**	MRPL30	0.41	mitochondrial ribosomal protein L30
**Q9BYC8**	MRPL32	-0.465	mitochondrial ribosomal protein L32
**Q9BZE1**	MRPL37	0.383	mitochondrial ribosomal protein L37
**Q96DV4**	MRPL38	0.363	mitochondrial ribosomal protein L38
**Q9NYK5**	MRPL39	0.269	mitochondrial ribosomal protein L39
**Q9BYD3**	MRPL4	0.258	mitochondrial ribosomal protein L4
**Q9H9J2**	MRPL44	0.423	mitochondrial ribosomal protein L44
**Q9BRJ2**	MRPL45	0.34	mitochondrial ribosomal protein L45
**Q13405**	MRPL49	0.23	mitochondrial ribosomal protein L49
**Q4U2R6**	MRPL51	0.433	mitochondrial ribosomal protein L51
**Q86TS9**	MRPL52	0.291	mitochondrial ribosomal protein L52
**Q7Z7F7-2**	MRPL55	0.238	mitochondrial ribosomal protein L55
**Q9BQC6**	MRPL57	0.386	mitochondrial ribosomal protein L57
**Q14197**	MRPL58	0.095	mitochondrial ribosomal protein L58
**Q9BYD2**	MRPL9	0.104	mitochondrial ribosomal protein L9
**P82912**	MRPS11	0.213	mitochondrial ribosomal protein S11
**O15235**	MRPS12	1.143	mitochondrial ribosomal protein S12
**O60783**	MRPS14	0.187	mitochondrial ribosomal protein S14
**P82914**	MRPS15	0.394	mitochondrial ribosomal protein S15
**Q9Y3D3**	MRPS16	0.162	mitochondrial ribosomal protein S16
**Q9NVS2**	MRPS18A	0.422	mitochondrial ribosomal protein S18A
**Q9Y399**	MRPS2	0.381	mitochondrial ribosomal protein S2
**P82921**	MRPS21	0.353	mitochondrial ribosomal protein S21
**P82650**	MRPS22	0.114	mitochondrial ribosomal protein S22
**Q96EL2**	MRPS24	0.622	mitochondrial ribosomal protein S24
**P82663**	MRPS25	0.213	mitochondrial ribosomal protein S25
**Q9BYN8**	MRPS26	0.255	mitochondrial ribosomal protein S26
**Q9NP92**	MRPS30	0.304	mitochondrial ribosomal protein S30
**Q92665**	MRPS31	0.171	mitochondrial ribosomal protein S31
**Q9Y291**	MRPS33	0.616	mitochondrial ribosomal protein S33
**P82930**	MRPS34	0.144	mitochondrial ribosomal protein S34
**P82673**	MRPS35	0.368	mitochondrial ribosomal protein S35
**P82909**	MRPS36	0.177	mitochondrial ribosomal protein S36
**P82675**	MRPS5	0.432	mitochondrial ribosomal protein S5
**P82932**	MRPS6	0.493	mitochondrial ribosomal protein S6
**Q9Y2R9**	MRPS7	0.312	mitochondrial ribosomal protein S7
**P82933**	MRPS9	0.437	mitochondrial ribosomal protein S9
**Q96E11**	MRRF	0.129	mitochondrial ribosome recycling factor
**Q9ULH7-5**	MRTFB	-0.339	myocardin related transcription factor B
**P43246**	MSH2	-0.274	mutS homolog 2
**P52701**	MSH6	-0.123	mutS homolog 6
**Q96DH6-1**	MSI2	-0.361	musashi RNA binding protein 2
**P03928**	MT-ATP8	0.35	ATP synthase F0 subunit 8
**P03915**	MT-ND5	0.527	NADH dehydrogenase, subunit 5 (complex I)
**P07438**	MT1B	2.98	metallothionein 1B
**O94776**	MTA2	0.396	metastasis associated 1 family member 2
**Q13126-1**	MTAP	-0.248	methylthioadenosine phosphorylase

**Q9NZJ7**	MTCH1	0.339	mitochondrial carrier 1
**Q96E29**	MTERF3	0.249	mitochondrial transcription termination factor 3
**Q9Y483**	MTF2	0.398	metal response element binding transcription factor 2
**Q9H019-1**	MTFR1L	-0.155	mitochondrial fission regulator 1 like
**P11586**	MTHFD1	-0.297	methylenetetrahydrofolate dehydrogenase, cyclohydrolase and formyltetrahydrofolate s
**P13995**	MTHFD2	0.344	methylenetetrahydrofolate dehydrogenase (NADP+ dependent) 2, methenyltetrahydrofo
**Q13496**	MTM1	-0.338	myotubularin 1
**Q13613**	MTMR1	-0.239	myotubularin related protein 1
**Q9C0I1**	MTMR12	-0.309	myotubularin related protein 12
**P42345**	MTOR	-0.098	mechanistic target of rapamycin kinase
**P58546**	MTPN	-0.246	myotrophin
**Q99707**	MTR	-0.405	5-methyltetrahydrofolate-homocysteine methyltransferase
**Q9UBK8-1**	MTRR	-0.28	5-methyltetrahydrofolate-homocysteine methyltransferase reductase
**Q765P7**	MTSS2	-0.53	MTSS I-BAR domain containing 2
**Q13505**	MTX1	0.228	metaxin 1
**Q5HYI7**	MTX3	0.359	metaxin 3
**Q969V5**	MUL1	0.262	mitochondrial E3 ubiquitin protein ligase 1
**P53602**	MVD	-0.215	mevalonate diphosphate decarboxylase
**Q03426**	MVK	-0.257	mevalonate kinase
**P84157-2**	MXRA7	0.177	matrix remodeling associated 7
**Q9BQG0-2**	MYBBP1A	-0.213	MYB binding protein 1a
**Q969H8**	MYDGF	0.265	myeloid derived growth factor
**Q7Z406-2**	MYH14	-0.178	myosin heavy chain 14
**P35579**	MYH9	0.089	myosin heavy chain 9
**O14950**	MYL12B	-0.155	myosin light chain 12B
**Q15746**	MYLK	-0.19	myosin light chain kinase
**Q96JP2**	MYO15B	-0.427	myosin XVB
**Q92614-1**	MYO18A	-0.211	myosin XVIIIA
**Q96H55-1**	MYO19	-0.216	myosin XIX
**O00159-3**	MYO1C	0.182	myosin IC
**Q12965**	MYO1E	-0.17	myosin IE
**Q6NSJ0**	MYORG	-0.445	myogenesis regulating glycosidase (putative)
**P41227**	NAA10	-0.355	N(alpha)-acetyltransferase 10, NatA catalytic subunit
**Q9BXJ9**	NAA15	-0.105	N(alpha)-acetyltransferase 15, NatA auxiliary subunit
**P61599**	NAA20	-0.321	N(alpha)-acetyltransferase 20, NatB catalytic subunit
**Q14CX7**	NAA25	-0.271	N(alpha)-acetyltransferase 25, NatB auxiliary subunit
**Q15742**	NAB2	0.25	NGFI-A binding protein 2
**Q4G0N4**	NADK2	0.173	NAD kinase 2, mitochondrial
**Q6IA69**	NADSYN1	-0.26	NAD synthetase 1
**Q13564-4**	NAE1	-0.324	NEDD8 activating enzyme E1 subunit 1
**Q9UJ70-2**	NAGK	-0.213	N-acetylglucosamine kinase
**P43490**	NAMPT	-0.079	nicotinamide phosphoribosyltransferase
**Q9NR45**	NANS	-0.221	N-acetylneuraminate synthase
**P55209**	NAP1L1	-0.124	nucleosome assembly protein 1 like 1
**Q99733-2**	NAP1L4	-0.126	nucleosome assembly protein 1 like 4
**O43776**	NARS1	-0.21	asparaginyl-tRNA synthetase 1
**Q96I59**	NARS2	0.363	asparaginyl-tRNA synthetase 2, mitochondrial

**P49321-3**	NASP	-0.435	nuclear autoantigenic sperm protein
**Q9H0A0**	NAT10	0.196	N-acetyltransferase 10
**Q8IVL1**	NAV2	-0.554	neuron navigator 2
**A2RRP1**	NBAS	-0.319	NBAS subunit of NRZ tethering complex
**Q15021**	NCAPD2	-0.263	non-SMC condensin I complex subunit D2
**Q9BPX3**	NCAPG	-0.22	non-SMC condensin I complex subunit G
**Q15003**	NCAPH	-0.237	non-SMC condensin I complex subunit H
**Q09161**	NCBP1	-0.237	nuclear cap binding protein subunit 1
**Q6PIU2-2**	NCEH1	0.451	neutral cholesterol ester hydrolase 1
**Q9NZQ3**	NCKIPSD	0.146	NCK interacting protein with SH3 domain
**Q9Y6Q9**	NCOA3	-0.277	nuclear receptor coactivator 3
**Q13772-4**	NCOA4	0.179	nuclear receptor coactivator 4
**Q9HCD5**	NCOA5	0.098	nuclear receptor coactivator 5
**O75376**	NCOR1	-0.216	nuclear receptor corepressor 1
**Q68D85**	NCR3LG1	-0.655	natural killer cell cytotoxicity receptor 3 ligand 1
**Q92542-1**	NCSTN	0.109	nicastrin
**O14777**	NDC80	-0.223	NDC80 kinetochore complex component
**Q9NXR1-1**	NDE1	-0.303	nudE neurodevelopment protein 1
**Q9UGV2**	NDRG3	-0.463	NDRG family member 3
**O95299**	NDUFA10	0.465	NADH:ubiquinone oxidoreductase subunit A10
**Q9UI09**	NDUFA12	0.292	NADH:ubiquinone oxidoreductase subunit A12
**Q9P0J0**	NDUFA13	0.437	NADH:ubiquinone oxidoreductase subunit A13
**O00483**	NDUFA4	-0.159	NDUFA4 mitochondrial complex associated
**P56556**	NDUFA6	0.456	NADH:ubiquinone oxidoreductase subunit A6
**O95182**	NDUFA7	0.29	NADH:ubiquinone oxidoreductase subunit A7
**P51970**	NDUFA8	0.29	NADH:ubiquinone oxidoreductase subunit A8
**Q16795**	NDUFA9	0.47	NADH:ubiquinone oxidoreductase subunit A9
**O14561**	NDUFAB1	0.385	NADH:ubiquinone oxidoreductase subunit AB1
**Q8N183**	NDUFAF2	0.188	NADH:ubiquinone oxidoreductase complex assembly factor 2
**Q9BU61-1**	NDUFAF3	-0.142	NADH:ubiquinone oxidoreductase complex assembly factor 3
**Q9P032**	NDUFAF4	0.172	NADH:ubiquinone oxidoreductase complex assembly factor 4
**Q7L592**	NDUFAF7	0.312	NADH:ubiquinone oxidoreductase complex assembly factor 7
**O96000**	NDUFB10	0.161	NADH:ubiquinone oxidoreductase subunit B10
**O43676**	NDUFB3	0.428	NADH:ubiquinone oxidoreductase subunit B3
**O95168**	NDUFB4	0.374	NADH:ubiquinone oxidoreductase subunit B4
**O43674-1**	NDUFB5	0.353	NADH:ubiquinone oxidoreductase subunit B5
**O95139**	NDUFB6	0.123	NADH:ubiquinone oxidoreductase subunit B6
**P17568**	NDUFB7	0.287	NADH:ubiquinone oxidoreductase subunit B7
**O95169**	NDUFB8	0.38	NADH:ubiquinone oxidoreductase subunit B8
**Q9Y6M9**	NDUFB9	0.44	NADH:ubiquinone oxidoreductase subunit B9
**O95298**	NDUFC2	0.4	NADH:ubiquinone oxidoreductase subunit C2
**P28331-2**	NDUFS1	0.194	NADH:ubiquinone oxidoreductase core subunit S1
**O75306**	NDUFS2	0.285	NADH:ubiquinone oxidoreductase core subunit S2
**O75489**	NDUFS3	0.338	NADH:ubiquinone oxidoreductase core subunit S3
**O43181**	NDUFS4	0.27	NADH:ubiquinone oxidoreductase subunit S4
**O43920**	NDUFS5	0.372	NADH:ubiquinone oxidoreductase subunit S5
**O75380**	NDUFS6	0.201	NADH:ubiquinone oxidoreductase subunit S6

**O75251**	NDUFS7	0.514	NADH:ubiquinone oxidoreductase core subunit S7
**O00217**	NDUFS8	0.188	NADH:ubiquinone oxidoreductase core subunit S8
**P49821**	NDUFV1	0.319	NADH:ubiquinone oxidoreductase core subunit V1
**P19404**	NDUFV2	-0.113	NADH:ubiquinone oxidoreductase core subunit V2
**Q92692-2**	NECTIN2	-0.167	nectin cell adhesion molecule 2
**Q8NHV4-3**	NEDD1	0.445	NEDD1 gamma-tubulin ring complex targeting factor
**P46934-4**	NEDD4	-0.269	NEDD4 E3 ubiquitin protein ligase
**Q15843**	NEDD8	-0.262	NEDD8 ubiquitin like modifier
**Q9H3P2**	NELFA	-0.16	negative elongation factor complex member A
**Q6NW34**	NEPRO	0.148	nucleolus and neural progenitor protein
**P48681**	NES	-0.226	nestin
**Q0ZGT2**	NEXN	0.434	nexilin F-actin binding protein
**O94916-4**	NFAT5	0.329	nuclear factor of activated T cells 5
**P19838-2**	NFKB1	-0.093	nuclear factor kappa B subunit 1
**Q9Y697**	NFS1	0.15	NFS1 cysteine desulfurase
**Q9UMS0**	NFU1	-0.271	NFU1 iron-sulfur cluster scaffold
**Q6ZNB6-1**	NFXL1	-0.59	nuclear transcription factor, X-box binding like 1
**Q8N5V2**	NGEF	-0.368	neuronal guanine nucleotide exchange factor
**Q96IV0**	NGLY1	-0.6	N-glycanase 1
**Q9NPE2-2**	NGRN	0.311	neugrin, neurite outgrowth associated
**Q9H9Q4-1**	NHEJ1	-0.502	non-homologous end joining factor 1
**Q9H9Q4-2**	NHEJ1	-0.382	non-homologous end joining factor 1
**Q5SYE7**	NHSL1	-0.362	NHS like 1
**P14543-1**	NID1	-0.436	nidogen 1
**Q9GZT8**	NIF3L1	-0.332	NGG1 interacting factor 3 like 1
**Q9BYG3**	NIFK	-0.155	nucleolar protein interacting with the FHA domain of MKI67
**Q92982**	NINJ1	0.21	ninjurin 1
**Q9Y221**	NIP7	0.192	nucleolar pre-rRNA processing protein NIP7
**Q9BPW8**	NIPSNAP1	0.383	nipsnap homolog 1
**O75323-1**	NIPSNAP2	0.372	nipsnap homolog 2
**Q9UFN0**	NIPSNAP3A	0.205	nipsnap homolog 3A
**Q9NQR4**	NIT2	-0.19	nitrilase family member 2
**Q8N5F7**	NKAP	0.125	NFKB activating protein
**Q969G9**	NKD1	-0.461	NKD inhibitor of WNT signaling pathway 1
**O15226-2**	NKRF	0.381	NFKB repressing factor
**P30414**	NKTR	0.292	natural killer cell triggering receptor
**Q9NVX2**	NLE1	-0.207	notchless homolog 1
**Q96D46**	NMD3	0.335	NMD3 ribosome export adaptor
**P22392-2**	NME2	-0.41	NME/NM23 nucleoside diphosphate kinase 2
**P30419**	NMT1	-0.346	N-myristoyltransferase 1
**Q13423**	NNT	0.393	nicotinamide nucleotide transhydrogenase
**Q8NC60**	NOA1	0.202	nitric oxide associated 1
**Q9ULX3**	NOB1	0.162	NIN1 (RPN12) binding protein 1 homolog
**Q9Y3T9**	NOC2L	0.335	NOC2 like nucleolar associated transcriptional repressor
**Q8WTT2**	NOC3L	0.498	NOC3 like DNA replication regulator
**Q9H8H0**	NOL11	0.116	nucleolar protein 11
**Q96MY1**	NOL4L	-0.317	nucleolar protein 4 like

**Q9UMY1**	NOL7	-0.375	nucleolar protein 7
**Q76FK4**	NOL8	0.145	nucleolar protein 8
**Q5C9Z4**	NOM1	0.336	nucleolar protein with MIF4G domain 1
**Q15233**	NONO	0.142	non-POU domain containing octamer binding
**Q9NPE3**	NOP10	0.258	NOP10 ribonucleoprotein
**Q9Y3C1**	NOP16	-0.471	NOP16 nucleolar protein
**P46087-4**	NOP2	0.495	NOP2 nucleolar protein
**Q9NZM5**	NOP53	-0.567	NOP53 ribosome biogenesis factor
**O00567**	NOP56	0.498	NOP56 ribonucleoprotein
**Q9Y2X3**	NOP58	0.414	NOP58 ribonucleoprotein
**O75052**	NOS1AP	-0.343	nitric oxide synthase 1 adaptor protein
**O15118**	NPC1	0.263	NPC intracellular cholesterol transporter 1
**P55786**	NPEPPS	-0.259	aminopeptidase puromycin sensitive
**P10588**	NR2F6	0.335	nuclear receptor subfamily 2 group F member 6
**O43847-2**	NRDC	-0.401	nardilysin convertase
**O95478**	NSA2	-0.65	NSA2 ribosome biogenesis factor
**O96028-1**	NSD2	-0.273	nuclear receptor binding SET domain protein 2
**Q15738**	NSDHL	0.088	NAD(P) dependent steroid dehydrogenase-like
**Q9UNZ2-5**	NSFL1C	-0.14	NSFL1 cofactor
**Q9H0G5**	NSRP1	-0.19	nuclear speckle splicing regulatory protein 1
**Q8TCD5**	NT5C	-0.221	5', 3'-nucleotidase, cytosolic
**P49902**	NT5C2	-0.148	5'-nucleotidase, cytosolic II
**Q5TFE4**	NT5DC1	-0.24	5'-nucleotidase domain containing 1
**Q9H857-2**	NT5DC2	0.446	5'-nucleotidase domain containing 2
**Q86UY8**	NT5DC3	0.377	5'-nucleotidase domain containing 3
**P21589-1**	NT5E	0.258	5'-nucleotidase ecto
**P78549**	NTHL1	-0.177	nth like DNA glycosylase 1
**Q9BSD7**	NTPCR	0.235	nucleoside-triphosphatase, cancer-related
**Q8TB37**	NUBPL	0.283	nucleotide binding protein like
**Q9H1E3**	NUCKS1	-0.479	nuclear casein kinase and cyclin dependent kinase substrate 1
**Q9Y266**	NUDC	-0.23	nuclear distribution C, dynein complex regulator
**Q96RS6**	NUDCD1	-0.123	NudC domain containing 1
**Q8WVJ2**	NUDCD2	-0.268	NudC domain containing 2
**Q8IVD9**	NUDCD3	-0.423	NudC domain containing 3
**Q9NV35**	NUDT15	-0.129	nudix hydrolase 15
**Q96DE0**	NUDT16	-0.115	nudix hydrolase 16
**Q9BRJ7-1**	NUDT16L1	0.33	nudix hydrolase 16 like 1
**A8MXV4**	NUDT19	0.494	nudix hydrolase 19
**P50583**	NUDT2	-0.411	nudix hydrolase 2
**Q9UKK9**	NUDT5	-0.327	nudix hydrolase 5
**Q7Z417**	NUFIP2	0.13	nuclear FMR1 interacting protein 2
**P49757**	NUMB	-0.209	NUMB endocytic adaptor protein
**Q8WUM0**	NUP133	0.095	nucleoporin 133
**P49790**	NUP153	0.311	nucleoporin 153
**Q5SRE5**	NUP188	0.094	nucleoporin 188
**Q8TEM1-1**	NUP210	0.272	nucleoporin 210
**Q8NFH4**	NUP37	0.192	nucleoporin 37

**Q9UKX7**	NUP50	0.13	nucleoporin 50
**P37198**	NUP62	-0.175	nucleoporin 62
**P61970**	NUTF2	-0.228	nuclear transport factor 2
**Q9UBU9-1**	NXF1	0.315	nuclear RNA export factor 1
**Q6DKJ4**	NXN	-0.226	nucleoredoxin
**Q9Y530**	OARD1	0.097	O-acyl-ADP-ribose deacylase 1
**P04181**	OAT	0.549	ornithine aminotransferase
**O75147**	OBSL1	-0.184	obscurin like cytoskeletal adaptor 1
**Q9NX40**	OCIAD1	0.155	OCIA domain containing 1
**Q16625**	OCLN	-0.215	occludin
**O60502**	OGA	0.096	O-GlcNAcase
**Q02218**	OGDH	0.365	oxoglutarate dehydrogenase
**Q6N063-1**	OGFOD2	-0.2	2-oxoglutarate and iron dependent oxygenase domain containing 2
**Q9NZT2**	OGFR	-0.186	opioid growth factor receptor
**O15294**	OGT	-0.166	O-linked N-acetylglucosamine (GlcNAc) transferase
**Q9NTK5-1**	OLA1	-0.3	Obg like ATPase 1
**Q9H6K4**	OPA3	0.499	outer mitochondrial membrane lipid metabolism regulator OPA3
**O14841**	OPLAH	-0.198	5-oxoprolinase, ATP-hydrolysing
**Q96CV9-1**	OPTN	-0.82	optineurin
**Q13415**	ORC1	0.744	origin recognition complex subunit 1
**Q13416**	ORC2	-0.126	origin recognition complex subunit 2
**Q9UBD5-2**	ORC3	0.179	origin recognition complex subunit 3
**O43929**	ORC4	0.098	origin recognition complex subunit 4
**Q9Y5N6**	ORC6	0.7	origin recognition complex subunit 6
**P19652**	ORM2	-0.376	orosomucoid 2
**Q9BZF1-1**	OSBPL8	0.122	oxysterol binding protein like 8
**Q92882**	OSTF1	-0.138	osteoclast stimulating factor 1
**Q96G74-1**	OTUD5	0.406	OTU deubiquitinase 5
**Q6GQQ9-1**	OTUD7B	0.188	OTU deubiquitinase 7B
**Q8N573-1**	OXR1	-0.48	oxidation resistance 1
**Q9NWU1-1**	OXSM	0.24	3-oxoacyl-ACP synthase, mitochondrial
**O95747**	OXSR1	-0.236	oxidative stress responsive kinase 1
**P07237**	P4HB	0.133	prolyl 4-hydroxylase subunit beta
**Q9BRP4**	PAAF1	-0.213	proteasomal ATPase associated factor 1
**Q6VY07-2**	PACS1	-0.388	phosphofurin acidic cluster sorting protein 1
**Q9UNF0**	PACSIN2	-0.204	protein kinase C and casein kinase substrate in neurons 2
**Q9UKS6**	PACSIN3	-0.189	protein kinase C and casein kinase substrate in neurons 3
**Q8N7H5**	PAF1	0.251	PAF1 homolog, Paf1/RNA polymerase II complex component
**P43034**	PAFAH1B1	-0.188	platelet activating factor acetylhydrolase 1b regulatory subunit 1
**Q15102**	PAFAH1B3	-0.268	platelet activating factor acetylhydrolase 1b catalytic subunit 3
**P00439**	PAH	-0.464	phenylalanine hydroxylase
**P22234-2**	PAICS	-0.269	phosphoribosylaminoimidazole carboxylase and phosphoribosylaminoimidazolesuccinoca
**Q9H074**	PAIP1	-0.258	poly(A) binding protein interacting protein 1
**Q9BPZ3**	PAIP2	0.346	poly(A) binding protein interacting protein 2
**Q9NWT1**	PAK1IP1	0.132	PAK1 interacting protein 1
**Q13177**	PAK2	-0.073	p21 (RAC1) activated kinase 2
**O96013**	PAK4	-0.153	p21 (RAC1) activated kinase 4

**A6NDB9**	PALM3	-0.433	paralemmin 3
**Q9NP74**	PALMD	-0.419	palmdelphin
**Q9BZ23**	PANK2	-0.113	pantothenate kinase 2
**Q9NVE7**	PANK4	-0.101	pantothenate kinase 4
**P51003**	PAPOLA	-0.123	poly(A) polymerase alpha
**O43252**	PAPSS1	-0.18	3'-phosphoadenosine 5'-phosphosulfate synthase 1
**Q9BYG5**	PARD6B	0.197	par-6 family cell polarity regulator beta
**Q86W56**	PARG	-0.135	poly(ADP-ribose) glycohydrolase
**O95453**	PARN	-0.195	poly(A)-specific ribonuclease
**P09874**	PARP1	0.339	poly(ADP-ribose) polymerase 1
**Q9UGN5-2**	PARP2	0.145	poly(ADP-ribose) polymerase 2
**Q7L3T8**	PARS2	0.188	prolyl-tRNA synthetase 2, mitochondrial
**Q9NVD7**	PARVA	-0.471	parvin alpha
**Q86TB9**	PATL1	0.161	PAT1 homolog 1, processing body mRNA decay factor
**Q9BVG4**	PBDC1	-0.144	polysaccharide biosynthesis domain containing 1
**P11498**	PC	0.197	pyruvate carboxylase
**P05165-1**	PCCA	0.19	propionyl-CoA carboxylase subunit alpha
**O94913**	PCF11	0.133	PCF11 cleavage and polyadenylation factor subunit
**P35227**	PCGF2	0.306	polycomb group ring finger 2
**Q9H4Z3**	PCIF1	-0.267	PDX1 C-terminal inhibiting factor 1
**Q16822**	PCK2	0.282	phosphoenolpyruvate carboxykinase 2, mitochondrial
**Q15004**	PCLAF	0.25	PCNA clamp associated factor
**Q15154-1**	PCM1	-0.208	pericentriolar material 1
**P12004**	PCNA	0.126	proliferating cell nuclear antigen
**Q8WW12**	PCNP	-0.274	PEST proteolytic signal containing nuclear protein
**Q9UKL6**	PCTP	-0.261	phosphatidylcholine transfer protein
**P49585**	PCYT1A	-0.198	phosphate cytidylyltransferase 1, choline, alpha
**Q99447**	PCYT2	-0.495	phosphate cytidylyltransferase 2, ethanolamine
**Q13442**	PDAP1	-0.127	PDGFA associated protein 1
**Q53EL6**	PDCD4	-0.23	programmed cell death 4
**O14737**	PDCD5	-0.276	programmed cell death 5
**Q8WUM4**	PDCD6IP	-0.184	programmed cell death 6 interacting protein
**Q13371**	PDCL	0.378	phosducin like
**Q9H2J4**	PDCL3	-0.26	phosducin like 3
**Q6L8Q7-1**	PDE12	0.137	phosphodiesterase 12
**Q13370**	PDE3B	-0.339	phosphodiesterase 3B
**Q9HBH1**	PDF	0.334	peptide deformylase, mitochondrial
**P08559-4**	PDHA1	0.097	pyruvate dehydrogenase E1 alpha 1 subunit
**P11177**	PDHB	0.185	pyruvate dehydrogenase E1 beta subunit
**O00330-1**	PDHX	0.227	pyruvate dehydrogenase complex component X
**P30101**	PDIA3	0.157	protein disulfide isomerase family A member 3
**Q15084-2**	PDIA6	0.117	protein disulfide isomerase family A member 6
**Q96JY6-5**	PDLIM2	-0.384	PDZ and LIM domain 2
**Q9NR12**	PDLIM7	0.43	PDZ and LIM domain 7
**Q9P0J1-2**	PDP1	0.424	pyruvate dehyrogenase phosphatase catalytic subunit 1
**Q8NCN5**	PDPR	0.367	pyruvate dehydrogenase phosphatase regulatory subunit
**Q6P996-1**	PDXDC1	-0.345	pyridoxal dependent decarboxylase domain containing 1

**Q5EBL8-2**	PDZD11	-0.121	PDZ domain containing 11
**Q5T2W1**	PDZK1	-0.181	PDZ domain containing 1
**Q15121-2**	PEA15	0.337	proliferation and apoptosis adaptor protein 15
**P30086**	PEBP1	-0.338	phosphatidylethanolamine binding protein 1
**Q9BY49-1**	PECR	0.298	peroxisomal trans-2-enoyl-CoA reductase
**Q9UBV8**	PEF1	-0.204	penta-EF-hand domain containing 1
**Q9GZU2**	PEG3	-0.226	paternally expressed 3
**Q9BRX2**	PELO	0.306	pelota mRNA surveillance and ribosome rescue factor
**P12955**	PEPD	-0.375	peptidase D
**O15067**	PFAS	-0.38	phosphoribosylformylglycinamidine synthase
**Q9UHV9**	PFDN2	-0.297	prefoldin subunit 2
**Q99471**	PFDN5	-0.264	prefoldin subunit 5
**O15212**	PFDN6	-0.259	prefoldin subunit 6
**O60825**	PFKFB2	0.141	6-phosphofructo-2-kinase/fructose-2,6-biphosphatase 2
**P17858-1**	PFKL	-0.18	phosphofructokinase, liver type
**Q01813**	PFKP	-0.376	phosphofructokinase, platelet
**P07737**	PFN1	-0.278	profilin 1
**P35080-2**	PFN2	-0.263	profilin 2
**P18669**	PGAM1	-0.154	phosphoglycerate mutase 1
**Q96HS1-1**	PGAM5	0.347	PGAM family member 5, mitochondrial serine/threonine protein phosphatase
**P00558**	PGK1	-0.125	phosphoglycerate kinase 1
**O95336**	PGLS	-0.29	6-phosphogluconolactonase
**P36871-1**	PGM1	-0.398	phosphoglucomutase 1
**Q96G03**	PGM2	-0.252	phosphoglucomutase 2
**O95394-4**	PGM3	-0.318	phosphoglucomutase 3
**A6NDG6**	PGP	-0.409	phosphoglycolate phosphatase
**Q9NXJ5**	PGPEP1	-0.3	pyroglutamyl-peptidase I
**O00264**	PGRMC1	0.446	progesterone receptor membrane component 1
**O75167-4**	PHACTR2	-0.401	phosphatase and actin regulator 2
**Q8IZ21-2**	PHACTR4	-0.29	phosphatase and actin regulator 4
**Q9H814**	PHAX	-0.284	phosphorylated adaptor for RNA export
**P35232**	PHB	0.251	prohibitin
**Q99623**	PHB2	0.374	prohibitin 2
**O94880**	PHF14	-0.146	PHD finger protein 14
**O75151**	PHF2	0.334	PHD finger protein 2
**A8MW92**	PHF20L1	-0.408	PHD finger protein 20 like 1
**Q9BUL5**	PHF23	0.235	PHD finger protein 23
**Q7RTV0**	PHF5A	0.243	PHD finger protein 5A
**Q8IWS0-1**	PHF6	0.113	PHD finger protein 6
**O43175**	PHGDH	-0.1	phosphoglycerate dehydrogenase
**Q8WWQ0**	PHIP	0.356	pleckstrin homology domain interacting protein
**P46019**	PHKA2	-0.136	phosphorylase kinase regulatory subunit alpha 2
**Q6NSJ2-1**	PHLDB3	0.302	pleckstrin homology like domain family B member 3
**Q96FC7**	PHYHIPL	-0.348	phytanoyl-CoA 2-hydroxylase interacting protein like
**Q9BTU6**	PI4K2A	0.167	phosphatidylinositol 4-kinase type 2 alpha
**O75925-2**	PIAS1	-0.528	protein inhibitor of activated STAT 1
**Q5H8A4-1**	PIGG	0.308	phosphatidylinositol glycan anchor biosynthesis class G

**Q92643**	PIGK	0.267	phosphatidylinositol glycan anchor biosynthesis class K
**Q96S52**	PIGS	0.13	phosphatidylinositol glycan anchor biosynthesis class S
**Q969N2**	PIGT	0.188	phosphatidylinositol glycan anchor biosynthesis class T
**Q9NWS0-1**	PIH1D1	-0.267	PIH1 domain containing 1
**Q9Y237-2**	PIN4	-0.203	peptidylprolyl cis/trans isomerase, NIMA-interacting 4
**Q96BK5**	PINX1	-0.263	PIN2 (TERF1) interacting telomerase inhibitor 1
**P48426**	PIP4K2A	-0.25	phosphatidylinositol-5-phosphate 4-kinase type 2 alpha
**P78356**	PIP4K2B	-0.144	phosphatidylinositol-5-phosphate 4-kinase type 2 beta
**Q99755**	PIP5K1A	0.171	phosphatidylinositol-4-phosphate 5-kinase type 1 alpha
**O00625**	PIR	-0.285	pirin
**Q9UG56-3**	PISD	-0.123	phosphatidylserine decarboxylase
**Q00169**	PITPNA	-0.18	phosphatidylinositol transfer protein alpha
**P48739-3**	PITPNB	-0.115	phosphatidylinositol transfer protein beta
**Q5JRX3-2**	PITRM1	0.371	pitrilysin metallopeptidase 1
**P30613**	PKLR	-0.451	pyruvate kinase L/R
**P14618**	PKM	-0.136	pyruvate kinase M1/2
**Q99640**	PKMYT1	0.198	protein kinase, membrane associated tyrosine/threonine 1
**Q16512-2**	PKN1	-0.162	protein kinase N1
**Q16513**	PKN2	-0.118	protein kinase N2
**Q99959-2**	PKP2	-0.094	plakophilin 2
**Q9Y263**	PLAA	-0.156	phospholipase A2 activating protein
**Q9NQ66-1**	PLCB1	-0.54	phospholipase C beta 1
**Q01970**	PLCB3	-0.351	phospholipase C beta 3
**Q13393-1**	PLD1	-0.201	phospholipase D1
**Q9Y2H5**	PLEKHA6	-0.248	pleckstrin homology domain containing A6
**Q6IQ23-2**	PLEKHA7	-0.437	pleckstrin homology domain containing A7
**Q9H8W4**	PLEKHF2	0.445	pleckstrin homology and FYVE domain containing 2
**Q8TD55**	PLEKHO2	0.317	pleckstrin homology domain containing O2
**Q99541**	PLIN2	0.59	perilipin 2
**O60664**	PLIN3	-0.126	perilipin 3
**Q02809-2**	PLOD1	-0.154	procollagen-lysine,2-oxoglutarate 5-dioxygenase 1
**O60568**	PLOD3	0.133	procollagen-lysine,2-oxoglutarate 5-dioxygenase 3
**O94903**	PLPBP	-0.164	pyridoxal phosphate binding protein
**O43660**	PLRG1	0.196	pleiotropic regulator 1
**Q14651**	PLS1	-0.302	plastin 1
**P13797**	PLS3	-0.285	plastin 3
**O43157**	PLXNB1	-0.227	plexin B1
**Q9Y4D7-1**	PLXND1	-0.397	plexin D1
**Q8IYS1**	PM20D2	-0.166	peptidase M20 domain containing 2
**Q6P1K2-2**	PMF1/PMF1-BGLAP	-0.4	polyamine modulated factor 1
**O15305**	PMM2	-0.443	phosphomannomutase 2
**Q10713**	PMPCA	0.247	peptidase, mitochondrial processing alpha subunit
**O75439**	PMPCB	0.26	peptidase, mitochondrial processing beta subunit
**Q8TF01**	PNISR	-0.246	PNN interacting serine and arginine rich protein
**Q8N490**	PNKD	-0.835	PNKD metallo-beta-lactamase domain containing
**Q96T60**	PNKP	0.142	polynucleotide kinase 3'-phosphatase
**Q9H307**	PNN	0.16	pinin, desmosome associated protein

**P41247**	PNPLA4	0.433	patatin like phospholipase domain containing 4
**Q9NVS9**	PNPO	-0.318	pyridoxamine 5'-phosphate oxidase
**Q8TCS8**	PNPT1	0.14	polyribonucleotide nucleotidyltransferase 1
**Q8NBL1**	POGLUT1	0.148	protein O-glucosyltransferase 1
**Q7Z3K3-1**	POGZ	0.156	pogo transposable element derived with ZNF domain
**P09884**	POLA1	-0.426	DNA polymerase alpha 1, catalytic subunit
**Q14181**	POLA2	-0.173	DNA polymerase alpha 2, accessory subunit
**P06746**	POLB	0.236	DNA polymerase beta
**P28340**	POLD1	-0.364	DNA polymerase delta 1, catalytic subunit
**P49005**	POLD2	-0.341	DNA polymerase delta 2, accessory subunit
**Q9Y2S7**	POLDIP2	0.141	DNA polymerase delta interacting protein 2
**Q07864**	POLE	-0.277	DNA polymerase epsilon, catalytic subunit
**Q9NRF9**	POLE3	-0.155	DNA polymerase epsilon 3, accessory subunit
**P54098**	POLG	0.348	DNA polymerase gamma, catalytic subunit
**O15160**	POLR1C	0.117	RNA polymerase I and III subunit C
**Q9GZS1-2**	POLR1E	0.322	RNA polymerase I subunit E
**P24928**	POLR2A	-0.174	RNA polymerase II subunit A
**P19388**	POLR2E	-0.158	RNA polymerase II subunit E
**P52434**	POLR2H	-0.403	RNA polymerase II subunit H
**P52435**	POLR2J	0.332	RNA polymerase II subunit J
**Q9BUI4**	POLR3C	0.209	RNA polymerase III subunit C
**Q9NVU0**	POLR3E	-0.182	RNA polymerase III subunit E
**A8CG34**	POM121/POM121C	0.606	POM121 transmembrane nucleoporin
**Q8WZA1-2**	POMGNT1	0.401	protein O-linked mannose N-acetylglucosaminyltransferase 1 (beta 1,2-)
**Q8NAT1**	POMGNT2	0.301	protein O-linked mannose N-acetylglucosaminyltransferase 2 (beta 1,4-)
**Q9H5K3**	POMK	0.335	protein O-mannose kinase
**Q9Y244**	POMP	-0.331	proteasome maturation protein
**O95707**	POP4	-0.144	POP4 homolog, ribonuclease P/MRP subunit
**P16435**	POR	0.238	cytochrome p450 oxidoreductase
**P14859-6**	POU2F1	-0.225	POU class 2 homeobox 1
**Q06203**	PPAT	-0.284	phosphoribosyl pyrophosphate amidotransferase
**Q9HAB8**	PPCS	-0.143	phosphopantothenoylcysteine synthetase
**P62937**	PPIA	-0.119	peptidylprolyl isomerase A
**P23284**	PPIB	0.136	peptidylprolyl isomerase B
**P45877**	PPIC	-0.143	peptidylprolyl isomerase C
**Q08752**	PPID	-0.188	peptidylprolyl isomerase D
**P30405**	PPIF	0.432	peptidylprolyl isomerase F
**Q9H2H8-1**	PPIL3	-0.185	peptidylprolyl isomerase like 3
**O15297**	PPM1D	0.543	protein phosphatase, Mg2+/Mn2+ dependent 1D
**P49593**	PPM1F	-0.23	protein phosphatase, Mg2+/Mn2+ dependent 1F
**O15355**	PPM1G	-0.125	protein phosphatase, Mg2+/Mn2+ dependent 1G
**Q9ULR3**	PPM1H	-0.359	protein phosphatase, Mg2+/Mn2+ dependent 1H
**Q96QC0**	PPP1R10	0.357	protein phosphatase 1 regulatory subunit 10
**Q96C90**	PPP1R14B	-0.594	protein phosphatase 1 regulatory inhibitor subunit 14B
**Q6NYC8**	PPP1R18	0.566	protein phosphatase 1 regulatory subunit 18
**Q6ZMI0-1**	PPP1R21	-0.3	protein phosphatase 1 regulatory subunit 21
**Q15435**	PPP1R7	-0.284	protein phosphatase 1 regulatory subunit 7

**Q9ULJ8-3**	PPP1R9A	-0.223	protein phosphatase 1 regulatory subunit 9A
**P30153**	PPP2R1A	-0.163	protein phosphatase 2 scaffold subunit Aalpha
**P30154**	PPP2R1B	-0.221	protein phosphatase 2 scaffold subunit Abeta
**Q15172-1**	PPP2R5A	-0.334	protein phosphatase 2 regulatory subunit B'alpha
**Q13362**	PPP2R5C	-0.193	protein phosphatase 2 regulatory subunit B'gamma
**Q16537**	PPP2R5E	-0.184	protein phosphatase 2 regulatory subunit B'epsilon
**Q08209**	PPP3CA	-0.321	protein phosphatase 3 catalytic subunit alpha
**P63098**	PPP3R1	-0.269	protein phosphatase 3 regulatory subunit B, alpha
**P60510**	PPP4C	-0.148	protein phosphatase 4 catalytic subunit
**Q8TF05**	PPP4R1	-0.301	protein phosphatase 4 regulatory subunit 1
**Q9NY27**	PPP4R2	-0.214	protein phosphatase 4 regulatory subunit 2
**P53041**	PPP5C	-0.206	protein phosphatase 5 catalytic subunit
**O00743-3**	PPP6C	-0.197	protein phosphatase 6 catalytic subunit
**O75170**	PPP6R2	-0.189	protein phosphatase 6 regulatory subunit 2
**Q5H9R7-5**	PPP6R3	-0.331	protein phosphatase 6 regulatory subunit 3
**O43663**	PRC1	0.438	protein regulator of cytokinesis 1
**Q92733**	PRCC	-0.123	proline rich mitotic checkpoint control factor
**P30048**	PRDX3	0.279	peroxiredoxin 3
**P30041**	PRDX6	-0.337	peroxiredoxin 6
**Q9HCU5**	PREB	0.207	prolactin regulatory element binding
**P48147**	PREP	-0.318	prolyl endopeptidase
**Q4J6C6**	PREPL	0.222	prolyl endopeptidase like
**P49642**	PRIM1	-0.196	DNA primase subunit 1
**P49643**	PRIM2	-0.403	DNA primase subunit 2
**P17612**	PRKACA	-0.197	protein kinase cAMP-activated catalytic subunit alpha
**P10644**	PRKAR1A	-0.285	protein kinase cAMP-dependent type I regulatory subunit alpha
**P31321**	PRKAR1B	-0.661	protein kinase cAMP-dependent type I regulatory subunit beta
**P17252**	PRKCA	-0.131	protein kinase C alpha
**Q05655**	PRKCD	0.1	protein kinase C delta
**O75569**	PRKRA	0.145	protein activator of interferon induced protein kinase EIF2AK2
**Q9H875**	PRKRIP1	-0.153	PRKR interacting protein 1
**Q99873**	PRMT1	-0.164	protein arginine methyltransferase 1
**O14744**	PRMT5	-0.159	protein arginine methyltransferase 5
**Q6P2P2**	PRMT9	-0.168	protein arginine methyltransferase 9
**P04070-2**	PROC	-0.428	protein C, inactivator of coagulation factors Va and VIIIa
**Q9UNN8**	PROCR	0.64	protein C receptor
**Q92786**	PROX1	0.153	prospero homeobox 1
**Q9UMS4**	PRPF19	0.094	pre-mRNA processing factor 19
**O43395**	PRPF3	0.122	pre-mRNA processing factor 3
**Q8NAV1-1**	PRPF38A	0.266	pre-mRNA processing factor 38A
**Q5VTL8**	PRPF38B	0.114	pre-mRNA processing factor 38B
**O43172**	PRPF4	0.08	pre-mRNA processing factor 4
**Q13523**	PRPF4B	0.245	pre-mRNA processing factor 4B
**P11908-2**	PRPS2	-0.157	phosphoribosyl pyrophosphate synthetase 2
**Q14558-2**	PRPSAP1	-0.191	phosphoribosyl pyrophosphate synthetase associated protein 1
**O60256-1**	PRPSAP2	-0.218	phosphoribosyl pyrophosphate synthetase associated protein 2
**Q5JSZ5-1**	PRRC2B	-0.165	proline rich coiled-coil 2B

**P07602-3**	PSAP	-0.404	prosaposin
**Q9Y617**	PSAT1	-0.255	phosphoserine aminotransferase 1
**P25786-2**	PSMA1	-0.152	proteasome subunit alpha 1
**P28066**	PSMA5	-0.151	proteasome subunit alpha 5
**P60900**	PSMA6	0.114	proteasome subunit alpha 6
**P28070**	PSMB4	0.126	proteasome subunit beta 4
**P28072**	PSMB6	-0.142	proteasome subunit beta 6
**Q99436**	PSMB7	-0.11	proteasome subunit beta 7
**Q99460**	PSMD1	-0.133	proteasome 26S subunit, non-ATPase 1
**Q9UNM6**	PSMD13	-0.141	proteasome 26S subunit, non-ATPase 13
**O00487**	PSMD14	0.115	proteasome 26S subunit, non-ATPase 14
**Q16401**	PSMD5	-0.297	proteasome 26S subunit, non-ATPase 5
**P48556**	PSMD8	-0.153	proteasome 26S subunit, non-ATPase 8
**O00233**	PSMD9	-0.121	proteasome 26S subunit, non-ATPase 9
**Q06323**	PSME1	-0.597	proteasome activator subunit 1
**Q9UL46**	PSME2	-0.322	proteasome activator subunit 2
**P61289-2**	PSME3	-0.161	proteasome activator subunit 3
**Q92530**	PSMF1	-0.314	proteasome inhibitor subunit 1
**O95456**	PSMG1	-0.358	proteasome assembly chaperone 1
**P78330**	PSPH	-0.421	phosphoserine phosphatase
**P26599-3**	PTBP1	-0.086	polypyrimidine tract binding protein 1
**O95758-4**	PTBP3	0.223	polypyrimidine tract binding protein 3
**O75127**	PTCD1	0.304	pentatricopeptide repeat domain 1
**Q96EY7-1**	PTCD3	0.3	pentatricopeptide repeat domain 3
**P48651**	PTDSS1	0.113	phosphatidylserine synthase 1
**Q9BVG9**	PTDSS2	-0.348	phosphatidylserine synthase 2
**Q14914**	PTGR1	0.326	prostaglandin reductase 1
**Q13308-6**	PTK7	-0.227	protein tyrosine kinase 7 (inactive)
**P06454**	PTMA	-0.314	prothymosin alpha
**P20962**	PTMS	-0.212	parathymosin
**Q15257**	PTPA	-0.364	protein phosphatase 2 phosphatase activator
**Q15257-2**	PTPA	-0.416	protein phosphatase 2 phosphatase activator
**Q06124**	PTPN11	-0.098	protein tyrosine phosphatase non-receptor type 11
**Q05209**	PTPN12	-0.217	protein tyrosine phosphatase non-receptor type 12
**P17706-1**	PTPN2	-0.253	protein tyrosine phosphatase non-receptor type 2
**Q9H3S7**	PTPN23	-0.181	protein tyrosine phosphatase non-receptor type 23
**P18433**	PTPRA	-0.251	protein tyrosine phosphatase receptor type A
**P10586-1**	PTPRF	-0.522	protein tyrosine phosphatase receptor type F
**Q15262-3**	PTPRK	-0.547	protein tyrosine phosphatase receptor type K
**Q15262-4**	PTPRK	-0.45	protein tyrosine phosphatase receptor type K
**Q86Y79**	PTRH1	0.27	peptidyl-tRNA hydrolase 1 homolog
**Q9Y3E5**	PTRH2	0.462	peptidyl-tRNA hydrolase 2
**Q00577**	PURA	-0.188	purine rich element binding protein A
**Q9Y606**	PUS1	0.147	pseudouridine synthase 1
**Q3MIT2**	PUS10	-0.402	pseudouridine synthase 10
**Q96PZ0**	PUS7	-0.251	pseudouridine synthase 7
**P15151-1**	PVR	0.339	PVR cell adhesion molecule

**P32322-3**	PYCR1	0.177	pyrroline-5-carboxylate reductase 1
**Q96C36**	PYCR2	0.41	pyrroline-5-carboxylate reductase 2
**P11216**	PYGB	-0.175	glycogen phosphorylase B
**P06737**	PYGL	-0.25	glycogen phosphorylase L
**Q9BRP8**	PYM1	-0.26	PYM homolog 1, exon junction complex associated factor
**Q96PU8**	QKI	-0.319	QKI, KH domain containing RNA binding
**Q15274**	QPRT	-0.223	quinolinate phosphoribosyltransferase
**Q6ZRP7**	QSOX2	-0.219	quiescin sulfhydryl oxidase 2
**Q9BXR0**	QTRT1	-0.129	queuine tRNA-ribosyltransferase catalytic subunit 1
**P61026**	RAB10	0.245	RAB10, member RAS oncogene family
**Q6IQ22**	RAB12	0.105	RAB12, member RAS oncogene family
**P61106**	RAB14	0.128	RAB14, member RAS oncogene family
**Q9NP72**	RAB18	0.194	RAB18, member RAS oncogene family
**P62820**	RAB1A	0.1	RAB1A, member RAS oncogene family
**Q9UL25**	RAB21	0.199	RAB21, member RAS oncogene family
**Q9UL26**	RAB22A	0.276	RAB22A, member RAS oncogene family
**P51159-1**	RAB27A	0.184	RAB27A, member RAS oncogene family
**P61019**	RAB2A	0.098	RAB2A, member RAS oncogene family
**Q8WUD1**	RAB2B	0.348	RAB2B, member RAS oncogene family
**Q13637**	RAB32	0.134	RAB32, member RAS oncogene family
**Q15286**	RAB35	0.15	RAB35, member RAS oncogene family
**O95716**	RAB3D	-0.209	RAB3D, member RAS oncogene family
**Q15042-1**	RAB3GAP1	-0.165	RAB3 GTPase activating protein catalytic subunit 1
**Q9H2M9**	RAB3GAP2	-0.186	RAB3 GTPase activating non-catalytic protein subunit 2
**Q86YS6**	RAB43	-0.104	RAB43, member RAS oncogene family
**P20338**	RAB4A	-0.182	RAB4A, member RAS oncogene family
**P51148-2**	RAB5C	0.092	RAB5C, member RAS oncogene family
**Q9BUV8-5**	RAB5IF	0.309	RAB5 interacting factor
**P61006**	RAB8A	0.177	RAB8A, member RAS oncogene family
**P51151**	RAB9A	0.141	RAB9A, member RAS oncogene family
**Q9UI14**	RABAC1	-0.223	Rab acceptor 1
**Q7Z6M1-1**	RABEPK	-0.341	Rab9 effector protein with kelch motifs
**Q9Y3P9**	RABGAP1	-0.47	RAB GTPase activating protein 1
**P53611**	RABGGTB	-0.2	Rab geranylgeranyltransferase subunit beta
**Q3YEC7-1**	RABL6	-0.174	RAB, member RAS oncogene family like 6
**P63000-2**	RAC1	0.267	Rac family small GTPase 1
**Q9H0H5**	RACGAP1	0.28	Rac GTPase activating protein 1
**P63244**	RACK1	-0.265	receptor for activated C kinase 1
**P54725-1**	RAD23A	-0.29	RAD23 homolog A, nucleotide excision repair protein
**P54725-3**	RAD23A	-0.437	RAD23 homolog A, nucleotide excision repair protein
**P54727**	RAD23B	-0.26	RAD23 homolog B, nucleotide excision repair protein
**Q96B01**	RAD51AP1	0.348	RAD51 associated protein 1
**P78406**	RAE1	0.331	ribonucleic acid export 1
**P11233**	RALA	0.13	RAS like proto-oncogene A
**P11234-2**	RALB	0.355	RAS like proto-oncogene B
**Q9UKM9**	RALY	0.24	RALY heterogeneous nuclear ribonucleoprotein
**P62826**	RAN	0.119	RAN, member RAS oncogene family

**P43487-2**	RANBP1	-0.779	RAN binding protein 1
**Q9H6Z4**	RANBP3	-0.331	RAN binding protein 3
**Q96S59-1**	RANBP9	0.128	RAN binding protein 9
**P46060**	RANGAP1	-0.149	Ran GTPase activating protein 1
**P52306-5**	RAP1GDS1	-0.494	Rap1 GTPase-GDP dissociation stimulator 1
**P61225**	RAP2B	0.213	RAP2B, member of RAS oncogene family
**Q8IY67-2**	RAVER1	-0.225	ribonucleoprotein, PTB binding 1
**P06400**	RB1	-0.183	RB transcriptional corepressor 1
**Q09028**	RBBP4	-0.149	RB binding protein 4, chromatin remodeling factor
**Q7Z6E9**	RBBP6	0.672	RB binding protein 6, ubiquitin ligase
**O75884**	RBBP9	-0.273	RB binding protein 9, serine hydrolase
**Q9BYM8**	RBCK1	-0.212	RANBP2-type and C3HC4-type zinc finger containing 1
**O43251-8**	RBFOX2	-0.623	RNA binding fox-1 homolog 2
**Q8N0T1**	RBIS	-0.955	ribosomal biogenesis factor
**Q9H477**	RBKS	-0.451	ribokinase
**Q8IXT5**	RBM12B	0.203	RNA binding motif protein 12B
**Q96PK6-1**	RBM14	0.147	RNA binding motif protein 14
**Q96T37**	RBM15	0.313	RNA binding motif protein 15
**Q96I25**	RBM17	0.166	RNA binding motif protein 17
**Q9Y4C8**	RBM19	-0.154	RNA binding motif protein 19
**Q9NW64**	RBM22	0.261	RNA binding motif protein 22
**P49756-1**	RBM25	0.169	RNA binding motif protein 25
**P98179**	RBM3	-0.211	RNA binding motif protein 3
**Q96EV2**	RBM33	0.176	RNA binding motif protein 33
**P42696**	RBM34	-0.288	RNA binding motif protein 34
**Q9BTD8**	RBM42	-0.145	RNA binding motif protein 42
**Q9BQ04**	RBM4B	0.376	RNA binding motif protein 4B
**P52756**	RBM5	0.345	RNA binding motif protein 5
**P38159-1**	RBMX	0.191	RNA binding motif protein X-linked
**Q9Y388**	RBMX2	0.207	RNA binding motif protein X-linked 2
**P02753**	RBP4	-0.302	retinol binding protein 4
**Q93062-3**	RBPMS	-0.253	RNA binding protein, mRNA processing factor
**Q5TC82-1**	RC3H1	-0.136	ring finger and CCCH-type domains 1
**P18754-2**	RCC1	0.086	regulator of chromosome condensation 1
**Q96I51**	RCC1L	0.195	RCC1 like
**Q9UKL0**	RCOR1	-0.175	REST corepressor 1
**Q8IZV5**	RDH10	0.163	retinol dehydrogenase 10
**Q8TC12**	RDH11	0.187	retinol dehydrogenase 11
**Q9HBH5**	RDH14	0.355	retinol dehydrogenase 14
**P35241-5**	RDX	0.132	radixin
**Q9H6H4**	REEP4	0.336	receptor accessory protein 4
**Q00765**	REEP5	0.339	receptor accessory protein 5
**Q04206**	RELA	-0.34	RELA proto-oncogene, NF-kB subunit
**Q9P260-2**	RELCH	-0.128	RAB11 binding and LisH domain, coiled-coil and HEAT repeat containing
**P78509**	RELN	-0.468	reelin
**Q9BWE0-4**	REPIN1	0.251	replication initiator 1
**Q8NC44**	RETREG2	-0.127	reticulophagy regulator family member 2

**Q86VR2**	RETREG3	-0.156	reticulophagy regulator family member 3
**Q6NUM9-1**	RETSAT	0.39	retinol saturase
**P35250-1**	RFC2	-0.233	replication factor C subunit 2
**P40937-1**	RFC5	-0.075	replication factor C subunit 5
**Q969G6**	RFK	0.422	riboflavin kinase
**Q6NTF9-2**	RHBDD2	0.347	rhomboid domain containing 2
**Q15382**	RHEB	0.149	Ras homolog, mTORC1 binding
**P84095**	RHOG	0.18	ras homolog family member G
**Q8IXI1**	RHOT2	0.176	ras homolog family member T2
**Q6R327-3**	RICTOR	-0.148	RPTOR independent companion of MTOR complex 2
**Q5UIP0**	RIF1	0.238	replication timing regulatory factor 1
**Q13671**	RIN1	0.667	Ras and Rab interactor 1
**Q6NUQ1**	RINT1	-0.09	RAD50 interactor 1
**Q9BRS2**	RIOK1	-0.238	RIO kinase 1
**Q9BVS4**	RIOK2	-0.204	RIO kinase 2
**O14730**	RIOK3	0.2	RIO kinase 3
**Q8IUF8**	RIOX2	-0.161	ribosomal oxygenase 2
**Q13546**	RIPK1	0.098	receptor interacting serine/threonine kinase 1
**Q96TC7**	RMDN3	0.296	regulator of microtubule dynamics 3
**Q9NWS8**	RMND1	0.398	required for meiotic nuclear division 1 homolog
**Q8TDP1**	RNASEH2C	-0.379	ribonuclease H2 subunit C
**Q9Y508**	RNF114	0.538	ring finger protein 114
**Q8NC42**	RNF149	-0.234	ring finger protein 149
**Q99496**	RNF2	0.091	ring finger protein 2
**Q63HN8-4**	RNF213	-0.194	ring finger protein 213
**Q8ND24**	RNF214	-0.088	ring finger protein 214
**O75150**	RNF40	0.118	ring finger protein 40
**O60942-1**	RNGTT	-0.239	RNA guanylyltransferase and 5'-phosphatase
**P13489**	RNH1	-0.247	ribonuclease/angiogenin inhibitor 1
**Q9H4A4**	RNPEP	-0.174	arginyl aminopeptidase
**Q15287-1**	RNPS1	0.224	RNA binding protein with serine rich domain 1
**Q9Y6N7-2**	ROBO1	-0.534	roundabout guidance receptor 1
**O75116**	ROCK2	-0.211	Rho associated coiled-coil containing protein kinase 2
**Q8TA86**	RP9	-0.353	RP9 pre-mRNA splicing factor
**Q9BWH6**	RPAP1	-0.164	RNA polymerase II associated protein 1
**Q9H6T3**	RPAP3	-0.234	RNA polymerase II associated protein 3
**Q96AT9**	RPE	-0.187	ribulose-5-phosphate-3-epimerase
**P49247**	RPIA	-0.126	ribose 5-phosphate isomerase A
**P30050**	RPL12	-0.24	ribosomal protein L12
**P50914**	RPL14	0.831	ribosomal protein L14
**Q07020**	RPL18	0.601	ribosomal protein L18
**P62829**	RPL23	-0.301	ribosomal protein L23
**P61254**	RPL26	0.273	ribosomal protein L26
**Q9UNX3**	RPL26L1	-0.532	ribosomal protein L26 like 1
**P46776**	RPL27A	0.197	ribosomal protein L27a
**P46779**	RPL28	0.442	ribosomal protein L28
**P62899**	RPL31	-0.343	ribosomal protein L31

**P18077**	RPL35A	0.318	ribosomal protein L35a
**Q9Y3U8**	RPL36	-0.163	ribosomal protein L36
**Q969Q0**	RPL36AL	0.848	ribosomal protein L36a like
**P05388**	RPLP0	-0.215	ribosomal protein lateral stalk subunit P0
**P04843**	RPN1	0.169	ribophorin I
**P04844-1**	RPN2	0.198	ribophorin II
**O75818**	RPP40	-0.076	ribonuclease P/MRP subunit p40
**Q96P16-1**	RPRD1A	-0.113	regulation of nuclear pre-mRNA domain containing 1A
**P62280**	RPS11	0.648	ribosomal protein S11
**P62841**	RPS15	-0.199	ribosomal protein S15
**P08708**	RPS17	-0.233	ribosomal protein S17
**P39019**	RPS19	-0.19	ribosomal protein S19
**Q86WX3**	RPS19BP1	-0.435	ribosomal protein S19 binding protein 1
**P15880**	RPS2	-0.167	ribosomal protein S2
**P60866**	RPS20	-0.137	ribosomal protein S20
**P63220**	RPS21	-0.385	ribosomal protein S21
**P42677**	RPS27	-0.214	ribosomal protein S27
**P62979**	RPS27A	0.682	ribosomal protein S27a
**Q71UM5**	RPS27L	0.225	ribosomal protein S27 like
**P62857**	RPS28	-0.172	ribosomal protein S28
**P62701**	RPS4X	0.16	ribosomal protein S4 X-linked
**P51812**	RPS6KA3	-0.323	ribosomal protein S6 kinase A3
**P46781**	RPS9	0.309	ribosomal protein S9
**P08865**	RPSA	-0.211	ribosomal protein SA
**Q8N122**	RPTOR	-0.332	regulatory associated protein of MTOR complex 1
**Q8IZ73**	RPUSD2	-0.169	RNA pseudouridine synthase domain containing 2
**P55042**	RRAD	2.84	RRAD, Ras related glycolysis inhibitor and calcium channel regulator
**Q7L523**	RRAGA	0.46	Ras related GTP binding A
**Q9HB90**	RRAGC	0.157	Ras related GTP binding C
**P62070-4**	RRAS2	0.363	RAS related 2
**Q9P2E9-1**	RRBP1	0.09	ribosome binding protein 1
**Q92766-2**	RREB1	-0.263	ras responsive element binding protein 1
**Q7LG56**	RRM2B	0.216	ribonucleotide reductase regulatory TP53 inducible subunit M2B
**Q7LG56-6**	RRM2B	0.497	ribonucleotide reductase regulatory TP53 inducible subunit M2B
**Q9NYV6**	RRN3	0.325	RRN3 homolog, RNA polymerase I transcription factor
**P56182**	RRP1	0.184	ribosomal RNA processing 1
**Q14684**	RRP1B	0.148	ribosomal RNA processing 1B
**Q96EU6**	RRP36	-0.412	ribosomal RNA processing 36
**O43159**	RRP8	0.154	ribosomal RNA processing 8
**Q15050**	RRS1	-0.405	ribosome biogenesis regulator 1 homolog
**Q9UHA3**	RSL24D1	-0.215	ribosomal L24 domain containing 1
**Q15404**	RSU1	-0.121	Ras suppressor protein 1
**Q9BY42**	RTF2	-0.122	replication termination factor 2
**Q9Y230**	RUVBL2	-0.143	RuvB like AAA ATPase 2
**Q6NW29**	RWDD4	-0.543	RWD domain containing 4
**P19793**	RXRA	-0.637	retinoid X receptor alpha
**P28702-3**	RXRB	-0.274	retinoid X receptor beta

**P23297**	S100A1	-0.67	S100 calcium binding protein A1
**P31949**	S100A11	0.833	S100 calcium binding protein A11
**Q99584**	S100A13	-0.097	S100 calcium binding protein A13
**Q96FQ6**	S100A16	0.221	S100 calcium binding protein A16
**P06703**	S100A6	0.154	S100 calcium binding protein A6
**P25815**	S100P	0.219	S100 calcium binding protein P
**Q96ER3**	SAAL1	-0.18	serum amyloid A like 1
**Q9UBE0**	SAE1	-0.24	SUMO1 activating enzyme subunit 1
**Q9Y3Z3**	SAMHD1	-0.154	SAM and HD domain containing deoxynucleoside triphosphate triphosphohydrolase 1
**O00422**	SAP18	0.305	Sin3A associated protein 18
**Q9Y6B6**	SAR1B	-0.226	secretion associated Ras related GTPase 1B
**P82979**	SARNP	0.253	SAP domain containing ribonucleoprotein
**Q9NP81**	SARS2	0.241	seryl-tRNA synthetase 2, mitochondrial
**O43290**	SART1	-0.1	spliceosome associated factor 1, recruiter of U4/U6.U5 tri-snRNP
**A3KN83**	SBNO1	0.221	strawberry notch homolog 1
**O15127**	SCAMP2	0.091	secretory carrier membrane protein 2
**Q8WTV0-2**	SCARB1	-0.126	scavenger receptor class B member 1
**Q8NBX0**	SCCPDH	0.204	saccharopine dehydrogenase (putative)
**Q8WU76-1**	SCFD2	-0.361	sec1 family domain containing 2
**O75880**	SCO1	0.333	SCO cytochrome c oxidase assembly protein 1
**O43819**	SCO2	0.14	SCO cytochrome c oxidase assembly protein 2
**P22307**	SCP2	0.235	sterol carrier protein 2
**Q9HB40**	SCPEP1	-0.226	serine carboxypeptidase 1
**Q12765-2**	SCRN1	-0.172	secernin 1
**P18827**	SDC1	-0.476	syndecan 1
**P34741**	SDC2	-1.046	syndecan 2
**O00560**	SDCBP	0.522	syndecan binding protein
**Q6IQ49-1**	SDE2	0.175	SDE2 telomere maintenance homolog
**Q99470**	SDF2	0.412	stromal cell derived factor 2
**P31040**	SDHA	0.33	succinate dehydrogenase complex flavoprotein subunit A
**P21912**	SDHB	0.164	succinate dehydrogenase complex iron sulfur subunit B
**Q9NRG7-2**	SDR39U1	0.246	short chain dehydrogenase/reductase family 39U member 1
**P67812-3**	SEC11A	0.29	SEC11 homolog A, signal peptidase complex subunit
**O75396**	SEC22B	0.204	SEC22 homolog B, vesicle trafficking protein (gene/pseudogene)
**Q15436**	SEC23A	-0.232	Sec23 homolog A, coat complex II component
**Q15437**	SEC23B	-0.246	SEC23 homolog B, coat complex II component
**Q9Y6Y8**	SEC23IP	-0.228	SEC23 interacting protein
**O95486**	SEC24A	-0.449	SEC24 homolog A, COPII coat complex component
**O95487-3**	SEC24B	-0.139	SEC24 homolog B, COPII coat complex component
**P53992**	SEC24C	-0.194	SEC24 homolog C, COPII coat complex component
**O94855-2**	SEC24D	-0.36	SEC24 homolog D, COPII coat complex component
**O94979-8**	SEC31A	-0.25	SEC31 homolog A, COPII coat complex component
**P61619**	SEC61A1	-0.132	SEC61 translocon alpha 1 subunit
**Q99442**	SEC62	0.154	SEC62 homolog, preprotein translocation factor
**Q13228-4**	SELENBP1	-0.334	selenium binding protein 1
**O60613**	SELENOF	-0.305	selenoprotein F
**Q9C0D9**	SELENOI	0.533	selenoprotein I

**P49903**	SEPHS1	-0.201	selenophosphate synthetase 1
**Q99611**	SEPHS2	-0.133	selenophosphate synthetase 2
**Q9NVA2-2**	SEPTIN11	-0.293	septin 11
**Q92599-1**	SEPTIN8	-0.23	septin 8
**Q9UHD8-1**	SEPTIN9	-0.346	septin 9
**Q8NC51-1**	SERBP1	-0.306	SERPINE1 mRNA binding protein 1
**P84101-1**	SERF2	-0.308	small EDRK-rich factor 2
**P05154**	SERPINA5	-0.349	serpin family A member 5
**P08185**	SERPINA6	-0.389	serpin family A member 6
**P05543**	SERPINA7	-0.253	serpin family A member 7
**P35237**	SERPINB6	-0.186	serpin family B member 6
**P50453**	SERPINB9	0.211	serpin family B member 9
**P01008**	SERPINC1	-0.637	serpin family C member 1
**P05546**	SERPIND1	-0.437	serpin family D member 1
**P05121**	SERPINE1	0.899	serpin family E member 1
**P08697-1**	SERPINF2	-0.493	serpin family F member 2
**P50454**	SERPINH1	0.611	serpin family H member 1
**P58004**	SESN2	1.277	sestrin 2
**Q01105**	SET	-0.172	SET nuclear proto-oncogene
**Q9BYW2**	SETD2	-0.275	SET domain containing 2, histone lysine methyltransferase
**Q86TU7**	SETD3	-0.305	SET domain containing 3, actin histidine methyltransferase
**Q15637-5**	SF1	-0.27	splicing factor 1
**Q15428**	SF3A2	0.239	splicing factor 3a subunit 2
**Q9Y3B4**	SF3B6	0.187	splicing factor 3b subunit 6
**P31947**	SFN	0.41	stratifin
**Q587I9**	SFT2D3	0.262	SFT2 domain containing 3
**Q9H9B4**	SFXN1	0.268	sideroflexin 1
**A0A0A0MS41**	SFXN3	0.338	sideroflexin 3
**Q8TD22**	SFXN5	0.295	sideroflexin 5
**Q562F6**	SGO2	0.415	shugoshin 2
**O95470**	SGPL1	0.148	sphingosine-1-phosphate lyase 1
**Q96EQ0**	SGTB	-0.326	small glutamine rich tetratricopeptide repeat containing beta
**Q9Y3L3-1**	SH3BP1	-0.222	SH3 domain binding protein 1
**Q9P0V3**	SH3BP4	0.436	SH3 domain binding protein 4
**Q9NR46-2**	SH3GLB2	-0.364	SH3 domain containing GRB2 like, endophilin B2
**Q8NEM2**	SHCBP1	0.617	SHC binding and spindle associated 1
**P34896**	SHMT1	-0.442	serine hydroxymethyltransferase 1
**P34897-1**	SHMT2	0.304	serine hydroxymethyltransferase 2
**Q9UQ13**	SHOC2	0.491	SHOC2 leucine rich repeat scaffold protein
**A0A0B4J2A0**	SHPK	-0.305	sedoheptulokinase
**Q2M3G4-1**	SHROOM1	-0.383	shroom family member 1
**A0MZ66-3**	SHTN1	-0.125	shootin 1
**Q99720**	SIGMAR1	0.371	sigma non-opioid intracellular receptor 1
**Q96EB6**	SIRT1	0.37	sirtuin 1
**Q9NTG7**	SIRT3	0.241	sirtuin 3
**Q9NXA8-1**	SIRT5	-0.115	sirtuin 5
**Q9NRC8**	SIRT7	0.272	sirtuin 7

**Q15477**	SKIV2L	-0.13	Ski2 like RNA helicase
**Q9UP95**	SLC12A4	0.148	solute carrier family 12 member 4
**Q9UHW9-2**	SLC12A6	-0.279	solute carrier family 12 member 6
**Q9Y666**	SLC12A7	-0.232	solute carrier family 12 member 7
**P53985**	SLC16A1	0.272	solute carrier family 16 member 1
**O15427**	SLC16A3	-0.205	solute carrier family 16 member 3
**P43007**	SLC1A4	-0.291	solute carrier family 1 member 4
**Q15758**	SLC1A5	0.101	solute carrier family 1 member 5
**Q8WUM9**	SLC20A1	-0.424	solute carrier family 20 member 1
**Q08357**	SLC20A2	0.719	solute carrier family 20 member 2
**Q9UGH3**	SLC23A2	-0.318	solute carrier family 23 member 2
**P53007**	SLC25A1	0.17	solute carrier family 25 member 1
**Q9UBX3-2**	SLC25A10	0.308	solute carrier family 25 member 10
**O75746**	SLC25A12	0.192	solute carrier family 25 member 12
**Q9UJS0-2**	SLC25A13	0.346	solute carrier family 25 member 13
**Q9Y619**	SLC25A15	0.284	solute carrier family 25 member 15
**Q6NUK1**	SLC25A24	0.22	solute carrier family 25 member 24
**Q00325-1**	SLC25A3	0.608	solute carrier family 25 member 3
**P05141**	SLC25A5	0.549	solute carrier family 25 member 5
**P12236**	SLC25A6	0.675	solute carrier family 25 member 6
**O14975**	SLC27A2	-0.184	solute carrier family 27 member 2
**Q6P1M0**	SLC27A4	-0.162	solute carrier family 27 member 4
**P11166**	SLC2A1	-0.16	solute carrier family 2 member 1
**Q9Y6M5**	SLC30A1	0.977	solute carrier family 30 member 1
**Q9HBR0**	SLC38A10	-0.224	solute carrier family 38 member 10
**Q99624**	SLC38A3	-0.234	solute carrier family 38 member 3
**Q15043**	SLC39A14	-0.379	solute carrier family 39 member 14
**P08195-4**	SLC3A2	0.359	solute carrier family 3 member 2
**Q8WWI5**	SLC44A1	-0.334	solute carrier family 44 member 1
**Q9UPY5**	SLC7A11	0.696	solute carrier family 7 member 11
**Q01650**	SLC7A5	0.255	solute carrier family 7 member 5
**O14745**	SLC9A3R1	-0.376	SLC9A3 regulator 1
**Q15599**	SLC9A3R2	-0.297	SLC9A3 regulator 2
**O95391**	SLU7	0.398	SLU7 homolog, splicing factor
**Q99717**	SMAD5	0.532	SMAD family member 5
**P51532-1**	SMARCA4	0.13	SWI/SNF related, matrix associated, actin dependent regulator of chromatin, subfamily a,
**O60264**	SMARCA5	0.298	SWI/SNF related, matrix associated, actin dependent regulator of chromatin, subfamily a,
**Q92925**	SMARCD2	-0.231	SWI/SNF related, matrix associated, actin dependent regulator of chromatin, subfamily d
**Q9NTJ3-1**	SMC4	0.074	structural maintenance of chromosomes 4
**Q8IY18**	SMC5	0.147	structural maintenance of chromosomes 5
**A6NHR9-1**	SMCHD1	0.139	structural maintenance of chromosomes flexible hinge domain containing 1
**Q96Q15-1**	SMG1	-0.272	SMG1 nonsense mediated mRNA decay associated PI3K related kinase
**Q86US8-1**	SMG6	-0.547	SMG6 nonsense mediated mRNA decay factor
**Q8ND04-2**	SMG8	0.231	SMG8 nonsense mediated mRNA decay factor
**Q8N5G0-2**	SMIM20	0.555	small integral membrane protein 20
**O75940**	SMNDC1	-0.216	survival motor neuron domain containing 1
**Q9NWM0**	SMOX	0.462	spermine oxidase

**P52788**	SMS	-0.228	spermine synthase
**P53814-6**	SMTN	0.989	smoothelin
**Q2TAY7**	SMU1	0.248	SMU1 DNA replication regulator and spliceosomal factor
**Q9NRG4**	SMYD2	-0.364	SET and MYND domain containing 2
**Q6GMV2**	SMYD5	-0.355	SMYD family member 5
**O00161**	SNAP23	-0.148	synaptosome associated protein 23
**Q7KZF4**	SND1	-0.232	staphylococcal nuclease and tudor domain containing 1
**Q96H20**	SNF8	-0.213	SNF8 subunit of ESCRT-II
**Q8TAD8**	SNIP1	0.341	Smad nuclear interacting protein 1
**O75643-1**	SNRNP200	0.187	small nuclear ribonucleoprotein U5 subunit 200
**P62314**	SNRPD1	0.284	small nuclear ribonucleoprotein D1 polypeptide
**P62316**	SNRPD2	-0.147	small nuclear ribonucleoprotein D2 polypeptide
**P62304**	SNRPE	0.115	small nuclear ribonucleoprotein polypeptide E
**Q13425-1**	SNTB2	0.217	syntrophin beta 2
**P55769**	SNU13	-0.152	small nuclear ribonucleoprotein 13
**Q13596**	SNX1	-0.173	sorting nexin 1
**Q9UMY4**	SNX12	-0.31	sorting nexin 12
**Q9NRS6**	SNX15	-0.363	sorting nexin 15
**Q15036-1**	SNX17	-0.228	sorting nexin 17
**O60749**	SNX2	-0.173	sorting nexin 2
**Q96L92**	SNX27	-0.168	sorting nexin 27
**Q5VWJ9**	SNX30	-0.265	sorting nexin family member 30
**O95219**	SNX4	-0.244	sorting nexin 4
**Q9Y5X3**	SNX5	-0.178	sorting nexin 5
**Q9UNH6-3**	SNX7	-0.363	sorting nexin 7
**Q9Y5X2**	SNX8	-0.389	sorting nexin 8
**P04179**	SOD2	0.419	superoxide dismutase 2
**O94964-2**	SOGA1	-0.124	suppressor of glucose, autophagy associated 1
**O94875-11**	SORBS2	-0.371	sorbin and SH3 domain containing 2
**Q00796**	SORD	-0.414	sorbitol dehydrogenase
**Q92673**	SORL1	-0.946	sortilin related receptor 1
**Q99523**	SORT1	-0.378	sortilin 1
**P48436**	SOX9	-0.291	SRY-box transcription factor 9
**Q07617**	SPAG1	0.343	sperm associated antigen 1
**Q96R06**	SPAG5	0.148	sperm associated antigen 5
**O75391**	SPAG7	-0.69	sperm associated antigen 7
**O60271**	SPAG9	-0.211	sperm associated antigen 9
**Q8TB22-2**	SPATA20	0.238	spermatogenesis associated 20
**Q8NB90-1**	SPATA5	-0.443	spermatogenesis associated 5
**Q9HBM1**	SPC25	-0.197	SPC25 component of NDC80 kinetochore complex
**Q15005**	SPCS2	0.232	signal peptidase complex subunit 2
**P61009**	SPCS3	0.729	signal peptidase complex subunit 3
**Q96EA4**	SPDL1	0.479	spindle apparatus coiled-coil protein 1
**Q9H2V7**	SPNS1	0.11	sphingolipid transporter 1 (putative)
**P10451**	SPP1	-0.308	secreted phosphoprotein 1
**P35270**	SPR	-0.104	sepiapterin reductase
**Q5W111**	SPRYD7	0.115	SPRY domain containing 7

**Q01082-3**	SPTBN1	0.677	spectrin beta, non-erythrocytic 1
**Q9NUV7**	SPTLC3	-0.412	serine palmitoyltransferase long chain base subunit 3
**Q68D10**	SPTY2D1	0.805	SPT2 chromatin protein domain containing 1
**Q14534**	SQLE	-0.153	squalene epoxidase
**Q9Y6N5**	SQOR	0.5	sulfide quinone oxidoreductase
**Q13501-1**	SQSTM1	0.323	sequestosome 1
**Q9HD15**	SRA1	-0.222	steroid receptor RNA activator 1
**P12931-2**	SRC	0.16	SRC proto-oncogene, non-receptor tyrosine kinase
**Q6ZRS2**	SRCAP	0.298	Snf2 related CREBBP activator protein
**O75044**	SRGAP2	-0.265	SLIT-ROBO Rho GTPase activating protein 2
**P19623**	SRM	-0.114	spermidine synthase
**Q9UHB9**	SRP68	-0.108	signal recognition particle 68
**O76094**	SRP72	-0.12	signal recognition particle 72
**P49458**	SRP9	-0.188	signal recognition particle 9
**Q96SB4**	SRPK1	-0.249	SRSF protein kinase 1
**Q9BXP5**	SRRT	0.157	serrate, RNA effector molecule
**Q01130**	SRSF2	-0.296	serine and arginine rich splicing factor 2
**Q13247**	SRSF6	0.239	serine and arginine rich splicing factor 6
**Q16629**	SRSF7	0.448	serine and arginine rich splicing factor 7
**Q9BYN0**	SRXN1	1.178	sulfiredoxin 1
**Q04837**	SSBP1	0.3	single stranded DNA binding protein 1
**P43307**	SSR1	0.14	signal sequence receptor subunit 1
**Q9UNL2-2**	SSR3	0.169	signal sequence receptor subunit 3
**P51571**	SSR4	0.19	signal sequence receptor subunit 4
**Q9NP77**	SSU72	-0.197	SSU72 homolog, RNA polymerase II CTD phosphatase
**P50502**	ST13	-0.196	ST13 Hsp70 interacting protein
**P15907**	ST6GAL1	-0.426	ST6 beta-galactoside alpha-2,6-sialyltransferase 1
**O95630**	STAMBP	-0.232	STAM binding protein
**Q9Y365**	STARD10	-0.184	StAR related lipid transfer domain containing 10
**Q9NUL3**	STAU2	-0.289	staufen double-stranded RNA binding protein 2
**Q658P3-2**	STEAP3	-0.877	STEAP3 metalloreductase
**Q9Y6E0**	STK24	-0.303	serine/threonine kinase 24
**Q13188-2**	STK3	-0.721	serine/threonine kinase 3
**Q8TDR2**	STK35	0.214	serine/threonine kinase 35
**Q9UEW8**	STK39	-0.46	serine/threonine kinase 39
**Q13043-1**	STK4	-0.095	serine/threonine kinase 4
**P16949-1**	STMN1	-0.308	stathmin 1
**P27105**	STOM	0.609	stomatin
**Q9UJZ1**	STOML2	0.256	stomatin like 2
**O43815**	STRN	-0.161	striatin
**P46977**	STT3A	0.144	STT3 oligosaccharyltransferase complex catalytic subunit A
**Q8TCJ2**	STT3B	0.137	STT3 oligosaccharyltransferase complex catalytic subunit B
**Q9UNE7-1**	STUB1	-0.19	STIP1 homology and U-box containing protein 1
**Q86Y82**	STX12	0.137	syntaxin 12
**Q9P2W9**	STX18	-0.165	syntaxin 18
**Q13277**	STX3	0.455	syntaxin 3
**Q12846**	STX4	0.153	syntaxin 4

**O43752**	STX6	0.307	syntaxin 6
**Q9UNK0**	STX8	-0.416	syntaxin 8
**O00186**	STXBP3	0.189	syntaxin binding protein 3
**P53999**	SUB1	0.211	SUB1 regulator of transcription
**Q9P2R7**	SUCLA2	0.314	succinate-CoA ligase ADP-forming beta subunit
**P53597**	SUCLG1	0.396	succinate-CoA ligase alpha subunit
**Q96I99**	SUCLG2	0.195	succinate-CoA ligase GDP-forming beta subunit
**P50225**	SULT1A1	-0.21	sulfotransferase family 1A member 1
**P0DMM9**	SULT1A3/SULT1A4	-0.241	sulfotransferase family 1A member 3
**Q06520**	SULT2A1	-0.357	sulfotransferase family 2A member 1
**O94901-9**	SUN1	0.323	Sad1 and UNC84 domain containing 1
**Q9Y5B9**	SUPT16H	0.143	SPT16 homolog, facilitates chromatin remodeling subunit
**Q8IYB8**	SUPV3L1	0.35	Suv3 like RNA helicase
**O15260**	SURF4	0.101	surfeit 4
**O75683**	SURF6	-0.855	surfeit 6
**Q9UH65**	SWAP70	0.099	switching B cell complex subunit SWAP70
**Q96A49**	SYAP1	-0.283	synapse associated protein 1
**O95926**	SYF2	-0.279	SYF2 pre-mRNA splicing factor
**Q9UMZ2**	SYNRG	-0.393	synergin gamma
**Q86TM6**	SYVN1	0.625	synoviolin 1
**Q15750-1**	TAB1	-0.217	TGF-beta activated kinase 1 (MAP3K7) binding protein 1
**O75410-2**	TACC1	-0.176	transforming acidic coiled-coil containing protein 1
**O95359**	TACC2	-0.187	transforming acidic coiled-coil containing protein 2
**Q9BSH4**	TACO1	0.161	translational activator of cytochrome c oxidase I
**O75528**	TADA3	0.172	transcriptional adaptor 3
**P49848-3**	TAF6	-0.152	TATA-box binding protein associated factor 6
**Q15545**	TAF7	0.138	TATA-box binding protein associated factor 7
**Q7Z7C8-4**	TAF8	0.117	TATA-box binding protein associated factor 8
**Q01995**	TAGLN	-0.316	transgelin
**P37802-2**	TAGLN2	0.225	transgelin 2
**Q96BW9-3**	TAMM41	0.483	TAM41 mitochondrial translocator assembly and maintenance homolog
**Q9C0B7**	TANGO6	-0.331	transport and golgi organization 6 homolog
**Q03518**	TAP1	0.416	transporter 1, ATP binding cassette subfamily B member
**O15533-3**	TAPBP	0.265	TAP binding protein
**P26639-2**	TARS1	-0.164	threonyl-tRNA synthetase 1
**Q9BW92**	TARS2	0.232	threonyl-tRNA synthetase 2, mitochondrial
**O14907**	TAX1BP3	0.156	Tax1 binding protein 3
**Q9BXI6-2**	TBC1D10A	0.304	TBC1 domain family member 10A
**Q9NVG8**	TBC1D13	-0.308	TBC1 domain family member 13
**Q96BZ9-3**	TBC1D20	0.423	TBC1 domain family member 20
**Q8WUA7**	TBC1D22A	-0.335	TBC1 domain family member 22A
**O60343-1**	TBC1D4	-0.235	TBC1 domain family member 4
**Q92609-2**	TBC1D5	-0.231	TBC1 domain family member 5
**O75347**	TBCA	-0.28	tubulin folding cofactor A
**Q99426**	TBCB	-0.203	tubulin folding cofactor B
**Q9BTW9-4**	TBCD	-0.214	tubulin folding cofactor D
**Q15813-2**	TBCE	-0.131	tubulin folding cofactor E

**Q9BZK7**	TBL1XR1	-0.209	transducin beta like 1 X-linked receptor 1
**Q9Y4P3**	TBL2	0.143	transducin beta like 2
**Q969Z0**	TBRG4	0.418	transforming growth factor beta regulator 4
**O75764**	TCEA3	-0.161	transcription elongation factor A3
**Q15170-2**	TCEAL1	-0.269	transcription elongation factor A like 1
**Q9NUW8**	TDP1	-0.217	tyrosyl-DNA phosphodiesterase 1
**Q9NZ01-1**	TECR	0.237	trans-2,3-enoyl-CoA reductase
**Q96QE5**	TEFM	0.477	transcription elongation factor, mitochondrial
**Q9Y4R8**	TELO2	-0.144	telomere maintenance 2
**Q15554**	TERF2	0.157	telomeric repeat binding factor 2
**Q9NYB0**	TERF2IP	0.443	TERF2 interacting protein
**Q9UGI8**	TES	0.253	testin LIM domain protein
**Q96BS2**	TESC	-0.371	tescalcin
**Q9NXF1**	TEX10	0.188	testis expressed 10
**Q8IWB9-2**	TEX2	-0.196	testis expressed 2
**P02787**	TF	-0.282	transferrin
**Q00059**	TFAM	0.939	transcription factor A, mitochondrial
**Q9H5Q4**	TFB2M	0.51	transcription factor B2, mitochondrial
**Q14186**	TFDP1	-0.17	transcription factor Dp-1
**Q92734**	TFG	-0.131	trafficking from ER to golgi regulator
**P10646**	TFPI	-0.24	tissue factor pathway inhibitor
**P01137**	TGFB1	0.56	transforming growth factor beta 1
**P37173-2**	TGFBR2	-0.47	transforming growth factor beta receptor 2
**Q03167**	TGFBR3	-0.652	transforming growth factor beta receptor 3
**P21980**	TGM2	-0.121	transglutaminase 2
**O43493-1**	TGOLN2	-0.229	trans-golgi network protein 2
**P07996**	THBS1	0.377	thrombospondin 1
**Q96FV9**	THOC1	0.139	THO complex 1
**Q13769**	THOC5	0.108	THO complex 5
**Q6I9Y2**	THOC7	-0.193	THO complex 7
**P52888**	THOP1	-0.279	thimet oligopeptidase 1
**Q9NXG2**	THUMPD1	0.132	THUMP domain containing 1
**Q9BV44**	THUMPD3	-0.128	THUMP domain containing 3
**Q9P016**	THYN1	-0.168	thymocyte nuclear protein 1
**Q01085-2**	TIAL1	-0.215	TIA1 cytotoxic granule associated RNA binding protein like 1
**Q9NQ88**	TIGAR	0.3	TP53 induced glycolysis regulatory phosphatase
**P62072**	TIMM10	0.097	translocase of inner mitochondrial membrane 10
**Q9Y5J6**	TIMM10B	-0.123	translocase of inner mitochondrial membrane 10B
**Q9Y5L4**	TIMM13	-0.253	translocase of inner mitochondrial membrane 13
**Q99595**	TIMM17A	-0.465	translocase of inner mitochondrial membrane 17A
**Q9BVV7**	TIMM21	0.302	translocase of inner mitochondrial membrane 21
**O14925**	TIMM23	-0.138	translocase of inner mitochondrial membrane 23
**Q9BSF4**	TIMM29	-0.571	translocase of inner mitochondrial membrane 29
**O43615**	TIMM44	0.505	translocase of inner mitochondrial membrane 44
**Q3ZCQ8-2**	TIMM50	0.21	translocase of inner mitochondrial membrane 50
**Q9BVW5**	TIPIN	-0.268	TIMELESS interacting protein
**Q5JTD0**	TJAP1	-0.125	tight junction associated protein 1

**Q9UDY2**	TJP2	-0.326	tight junction protein 2
**P04183**	TK1	0.44	thymidine kinase 1
**Q3LXA3**	TKFC	-0.415	triokinase and FMN cyclase
**P29401-2**	TKT	-0.086	transketolase
**Q99805**	TM9SF2	-0.196	transmembrane 9 superfamily member 2
**Q9HD45**	TM9SF3	0.298	transmembrane 9 superfamily member 3
**Q96EY4**	TMA16	0.17	translation machinery associated 16 homolog
**Q9Y2S6**	TMA7	0.177	translation machinery associated 7 homolog
**Q9UM00**	TMCO1	0.393	transmembrane and coiled-coil domains 1
**P49755**	TMED10	0.158	transmembrane p24 trafficking protein 10
**Q9Y3A6**	TMED5	0.378	transmembrane p24 trafficking protein 5
**Q9NUM4**	TMEM106B	-0.143	transmembrane protein 106B
**P17152**	TMEM11	0.332	transmembrane protein 11
**Q9H061**	TMEM126A	0.401	transmembrane protein 126A
**Q9P0S9**	TMEM14C	0.32	transmembrane protein 14C
**Q9HC07**	TMEM165	0.082	transmembrane protein 165
**Q8TBQ9**	TMEM167A	0.354	transmembrane protein 167A
**Q53S58**	TMEM177	0.359	transmembrane protein 177
**Q8N511**	TMEM199	0.298	transmembrane protein 199
**Q5SNT2-1**	TMEM201	0.573	transmembrane protein 201
**Q96SK2**	TMEM209	0.298	transmembrane protein 209
**Q96A57-2**	TMEM230	0.354	transmembrane protein 230
**Q8WUH6**	TMEM263	0.188	transmembrane protein 263
**P57088**	TMEM33	0.397	transmembrane protein 33
**Q9BTV4**	TMEM43	0.164	transmembrane protein 43
**Q9BXS4**	TMEM59	-0.213	transmembrane protein 59
**Q9BUB7**	TMEM70	0.551	transmembrane protein 70
**P42166**	TMPO	0.218	thymopoietin
**P42167**	TMPO	0.341	thymopoietin
**P63313**	TMSB10/TMSB4X	0.433	thymosin beta 4 X-linked
**Q9H3N1**	TMX1	0.177	thioredoxin related transmembrane protein 1
**Q9Y320**	TMX2	0.246	thioredoxin related transmembrane protein 2
**Q9H1E5**	TMX4	0.277	thioredoxin related transmembrane protein 4
**O00220**	TNFRSF10A	0.421	TNF receptor superfamily member 10a
**O14763**	TNFRSF10B	0.168	TNF receptor superfamily member 10b
**Q9C0C2**	TNKS1BP1	-0.123	tankyrase 1 binding protein 1
**O14787-2**	TNPO2	-0.231	transportin 2
**Q8NDV7**	TNRC6A	-0.137	trinucleotide repeat containing adaptor 6A
**Q68CZ2-1**	TNS3	-0.333	tensin 3
**O60784**	TOM1	-0.327	target of myb1 membrane trafficking protein
**Q9NS69**	TOMM22	0.156	translocase of outer mitochondrial membrane 22
**Q15785**	TOMM34	0.269	translocase of outer mitochondrial membrane 34
**O96008**	TOMM40	0.134	translocase of outer mitochondrial membrane 40
**Q8N4H5**	TOMM5	0.233	translocase of outer mitochondrial membrane 5
**O94826**	TOMM70	0.196	translocase of outer mitochondrial membrane 70
**P11387**	TOP1	0.629	DNA topoisomerase I
**P11388-4**	TOP2A	0.69	DNA topoisomerase II alpha

**Q02880**	TOP2B	0.436	DNA topoisomerase II beta
**O14656**	TOR1A	0.175	torsin family 1 member A
**Q5JTV8**	TOR1AIP1	0.402	torsin 1A interacting protein 1
**Q8NFQ8**	TOR1AIP2	0.293	torsin 1A interacting protein 2
**O14657**	TOR1B	0.131	torsin family 1 member B
**O94842**	TOX4	0.229	TOX high mobility group box family member 4
**P04637**	TP53	1.04	tumor protein p53
**Q53FA7**	TP53I3	0.428	tumor protein p53 inducible protein 3
**P55327-3**	TPD52	-0.403	tumor protein D52
**P60174**	TPI1	-0.186	triosephosphate isomerase 1
**P09493-2**	TPM1	-0.375	tropomyosin 1
**P67936**	TPM4	-0.179	tropomyosin 4
**Q9ULW0**	TPX2	0.802	TPX2 microtubule nucleation factor
**Q13595**	TRA2A	0.183	transformer 2 alpha homolog
**P62995**	TRA2B	0.267	transformer 2 beta homolog
**Q9H4I3-1**	TRABD	0.215	TraB domain containing
**Q12931**	TRAP1	0.295	TNF receptor associated protein 1
**O43617-1**	TRAPPC3	-0.167	trafficking protein particle complex 3
**Q9Y296**	TRAPPC4	0.182	trafficking protein particle complex 4
**Q8IUR0**	TRAPPC5	0.105	trafficking protein particle complex 5
**Q86SZ2**	TRAPPC6B	-0.207	trafficking protein particle complex 6B
**O15164**	TRIM24	-0.26	tripartite motif containing 24
**Q14258**	TRIM25	-0.121	tripartite motif containing 25
**Q12899**	TRIM26	0.191	tripartite motif containing 26
**P14373**	TRIM27	-0.166	tripartite motif containing 27
**Q13263**	TRIM28	0.274	tripartite motif containing 28
**Q9UPN9-1**	TRIM33	-0.319	tripartite motif containing 33
**O75962-1**	TRIO	-0.798	trio Rho guanine nucleotide exchange factor
**Q9H2D6**	TRIOBP	0.195	TRIO and F-actin binding protein
**Q15642**	TRIP10	-0.181	thyroid hormone receptor interactor 10
**Q15643**	TRIP11	0.188	thyroid hormone receptor interactor 11
**Q15645**	TRIP13	-0.176	thyroid hormone receptor interactor 13
**Q15650**	TRIP4	-0.424	thyroid hormone receptor interactor 4
**Q9BQ61**	TRIR	-0.399	telomerase RNA component interacting RNase
**Q7L0Y3**	TRMT10C	0.559	tRNA methyltransferase 10C, mitochondrial RNase P subunit
**Q8IZ69**	TRMT2A	-0.296	tRNA methyltransferase 2 homolog A
**O75648**	TRMU	0.242	tRNA 5-methylaminomethyl-2-thiouridylate methyltransferase
**Q8TEL6**	TRPC4AP	-0.364	transient receptor potential cation channel subfamily C member 4 associated protein
**O95900**	TRUB2	0.173	TruB pseudouridine synthase family member 2
**Q15631-1**	TSN	-0.205	translin
**Q99598**	TSNAX	-0.087	translin associated factor X
**Q9H0U9**	TSPYL1	0.46	TSPY like 1
**Q9H2G4**	TSPYL2	0.961	TSPY like 2
**Q16762**	TST	0.394	thiosulfate sulfurtransferase
**Q13630**	TSTA3	-0.208	tissue specific transplantation antigen P35B
**Q99614**	TTC1	-0.23	tetratricopeptide repeat domain 1
**Q96AE7-2**	TTC17	-0.18	tetratricopeptide repeat domain 17

**Q6P3X3**	TTC27	-0.474	tetratricopeptide repeat domain 27
**Q5R3I4**	TTC38	-0.486	tetratricopeptide repeat domain 38
**Q14166**	TTLL12	-0.235	tubulin tyrosine ligase like 12
**Q9BTX7**	TTPAL	-0.371	alpha tocopherol transfer protein like
**P68366**	TUBA4A	0.394	tubulin alpha 4a
**P07437**	TUBB	-0.074	tubulin beta class I
**Q13885**	TUBB2A	0.38	tubulin beta 2A class IIa
**Q13509**	TUBB3	0.24	tubulin beta 3 class III
**Q9BUF5**	TUBB6	0.498	tubulin beta 6 class V
**Q9BSJ2-4**	TUBGCP2	-0.09	tubulin gamma complex associated protein 2
**Q96CW5**	TUBGCP3	-0.123	tubulin gamma complex associated protein 3
**P49411**	TUFM	0.308	Tu translation elongation factor, mitochondrial
**O75896**	TUSC2	0.175	tumor suppressor 2, mitochondrial calcium regulator
**Q5TAX3**	TUT4	-0.251	terminal uridylyl transferase 4
**Q96RR1**	TWNK	0.314	twinkle mtDNA helicase
**P10599-1**	TXN	-0.227	thioredoxin
**Q99757**	TXN2	0.878	thioredoxin 2
**Q96J42-1**	TXNDC15	-0.205	thioredoxin domain containing 15
**Q9BRA2**	TXNDC17	-0.183	thioredoxin domain containing 17
**O14530**	TXNDC9	0.232	thioredoxin domain containing 9
**O43396**	TXNL1	-0.114	thioredoxin like 1
**P83876**	TXNL4A	-0.376	thioredoxin like 4A
**Q9NX01**	TXNL4B	0.38	thioredoxin like 4B
**P19971-2**	TYMP	-0.478	thymidine phosphorylase
**P04818**	TYMS	0.172	thymidylate synthetase
**Q2T9J0**	TYSND1	-0.401	trypsin domain containing 1
**Q01081**	U2AF1/U2AF1L5	0.155	U2 small nuclear RNA auxiliary factor 1
**P26368**	U2AF2	-0.099	U2 small nuclear RNA auxiliary factor 2
**Q9BZF9**	UACA	-0.226	uveal autoantigen with coiled-coil domains and ankyrin repeats
**Q3KQV9**	UAP1L1	-0.255	UDP-N-acetylglucosamine pyrophosphorylase 1 like 1
**P22314**	UBA1	-0.169	ubiquitin like modifier activating enzyme 1
**Q9UBT2**	UBA2	-0.174	ubiquitin like modifier activating enzyme 2
**Q8TBC4-1**	UBA3	-0.12	ubiquitin like modifier activating enzyme 3
**Q9GZZ9-1**	UBA5	-0.212	ubiquitin like modifier activating enzyme 5
**A0AVT1-1**	UBA6	-0.125	ubiquitin like modifier activating enzyme 6
**Q9BSL1**	UBAC1	-0.364	UBA domain containing 1
**Q5T6F2**	UBAP2	-0.159	ubiquitin associated protein 2
**O00762**	UBE2C	0.083	ubiquitin conjugating enzyme E2 C
**P51965-1**	UBE2E1	-0.395	ubiquitin conjugating enzyme E2 E1
**Q969T4**	UBE2E3	-0.449	ubiquitin conjugating enzyme E2 E3
**P62253**	UBE2G1	-0.199	ubiquitin conjugating enzyme E2 G1
**P63279**	UBE2I	-0.189	ubiquitin conjugating enzyme E2 I
**P61086**	UBE2K	-0.348	ubiquitin conjugating enzyme E2 K
**P68036**	UBE2L3	-0.295	ubiquitin conjugating enzyme E2 L3
**P61081**	UBE2M	-0.205	ubiquitin conjugating enzyme E2 M
**P61088**	UBE2N	-0.321	ubiquitin conjugating enzyme E2 N
**Q9C0C9**	UBE2O	-0.194	ubiquitin conjugating enzyme E2 O

**Q712K3**	UBE2R2	-0.359	ubiquitin conjugating enzyme E2 R2
**Q16763**	UBE2S	0.859	ubiquitin conjugating enzyme E2 S
**Q9NPD8**	UBE2T	-0.152	ubiquitin conjugating enzyme E2 T
**Q13404-1**	UBE2V1	-0.299	ubiquitin conjugating enzyme E2 V1
**Q15386-1**	UBE3C	-0.23	ubiquitin protein ligase E3C
**Q14139-2**	UBE4A	-0.212	ubiquitination factor E4A
**O95155-4**	UBE4B	-0.309	ubiquitination factor E4B
**P11441**	UBL4A	-0.147	ubiquitin like 4A
**Q96S82**	UBL7	-0.316	ubiquitin like 7
**Q8WVY7**	UBLCP1	-0.315	ubiquitin like domain containing CTD phosphatase 1
**Q9UMX0-1**	UBQLN1	-0.139	ubiquilin 1
**Q9NRR5**	UBQLN4	-0.276	ubiquilin 4
**Q8IWV7**	UBR1	-0.304	ubiquitin protein ligase E3 component n-recognin 1
**Q8IWV8**	UBR2	-0.2	ubiquitin protein ligase E3 component n-recognin 2
**Q5T4S7-2**	UBR4	-0.264	ubiquitin protein ligase E3 component n-recognin 4
**Q8N806**	UBR7	-0.203	ubiquitin protein ligase E3 component n-recognin 7 (putative)
**Q04323-1**	UBXN1	-0.261	UBX domain protein 1
**Q92575**	UBXN4	0.183	UBX domain protein 4
**Q9BZX2-1**	UCK2	-0.13	uridine-cytidine kinase 2
**Q9Y3C8**	UFC1	-0.191	ubiquitin-fold modifier conjugating enzyme 1
**Q9NUQ7**	UFSP2	0.187	UFM1 specific peptidase 2
**O60701**	UGDH	-0.092	UDP-glucose 6-dehydrogenase
**Q16851**	UGP2	-0.128	UDP-glucose pyrophosphorylase 2
**P16662**	UGT2B7	0.581	UDP glucuronosyltransferase family 2 member B7
**Q96T88-2**	UHRF1	-0.445	ubiquitin like with PHD and ring finger domains 1
**Q6BDS2**	UHRF1BP1	-0.181	UHRF1 binding protein 1
**A0JNW5**	UHRF1BP1L	-0.263	UHRF1 binding protein 1 like
**Q96RL1**	UIMC1	-0.151	ubiquitin interaction motif containing 1
**Q70J99-3**	UNC13D	-0.262	unc-13 homolog D
**Q92900**	UPF1	-0.119	UPF1 RNA helicase and ATPase
**Q9HAU5**	UPF2	-0.178	UPF2 regulator of nonsense mediated mRNA decay
**Q9BZI7-1**	UPF3B	0.227	UPF3B regulator of nonsense mediated mRNA decay
**Q9BRT2**	UQCC2	-0.132	ubiquinol-cytochrome c reductase complex assembly factor 2
**P31930**	UQCRC1	0.276	ubiquinol-cytochrome c reductase core protein 1
**P47985**	UQCRFS1	0.18	ubiquinol-cytochrome c reductase, Rieske iron-sulfur polypeptide 1
**O14949**	UQCRQ	0.301	ubiquinol-cytochrome c reductase complex III subunit VII
**Q8TCY9**	URGCP	0.151	upregulator of cell proliferation
**P06132**	UROD	-0.322	uroporphyrinogen decarboxylase
**O60763-1**	USO1	-0.175	USO1 vesicle transport factor
**O60763-2**	USO1	-0.3	USO1 vesicle transport factor
**Q14694-2**	USP10	-0.178	ubiquitin specific peptidase 10
**Q92995**	USP13	-0.364	ubiquitin specific peptidase 13
**P54578**	USP14	-0.134	ubiquitin specific peptidase 14
**O94966-5**	USP19	0.144	ubiquitin specific peptidase 19
**Q9UPU5**	USP24	-0.155	ubiquitin specific peptidase 24
**Q96RU2-2**	USP28	-0.191	ubiquitin specific peptidase 28
**Q9Y6I4**	USP3	0.121	ubiquitin specific peptidase 3

**Q8NFA0**	USP32	-0.421	ubiquitin specific peptidase 32
**Q8TEY7**	USP33	-0.413	ubiquitin specific peptidase 33
**Q70CQ2**	USP34	-0.298	ubiquitin specific peptidase 34
**Q96K76**	USP47	-0.166	ubiquitin specific peptidase 47
**Q86UV5-8**	USP48	-0.358	ubiquitin specific peptidase 48
**P45974-1**	USP5	-0.159	ubiquitin specific peptidase 5
**Q93009**	USP7	-0.123	ubiquitin specific peptidase 7
**P40818**	USP8	-0.088	ubiquitin specific peptidase 8
**Q93008**	USP9X	-0.247	ubiquitin specific peptidase 9 X-linked
**Q9BVJ6-1**	UTP14A	-0.347	UTP14A small subunit processome component
**Q8TED0**	UTP15	0.206	UTP15 small subunit processome component
**Q68CQ4**	UTP25	0.157	UTP25 small subunit processor component
**Q9NQZ2**	UTP3	0.501	UTP3 small subunit processome component
**Q969X6**	UTP4	0.314	UTP4 small subunit processome component
**P46939**	UTRN	-0.103	utrophin
**Q08AM6**	VAC14	-0.337	VAC14 component of PIKFYVE complex
**P51809**	VAMP7	0.172	vesicle associated membrane protein 7
**Q9BV40**	VAMP8	0.185	vesicle associated membrane protein 8
**Q9P0L0-2**	VAPA	0.401	VAMP associated protein A
**O95292**	VAPB	0.191	VAMP associated protein B and C
**P26640**	VARS	-0.179	valyl-tRNA synthetase 1
**Q6EMK4**	VASN	-0.405	vasorin
**Q99536**	VAT1	-0.17	vesicle amine transport 1
**P52735**	VAV2	-0.362	vav guanine nucleotide exchange factor 2
**P52735-2**	VAV2	-0.421	vav guanine nucleotide exchange factor 2
**P61758**	VBP1	-0.212	VHL binding protein 1
**P18206**	VCL	-0.386	vinculin
**P21796**	VDAC1	0.319	voltage dependent anion channel 1
**P45880-1**	VDAC2	0.532	voltage dependent anion channel 2
**Q9Y277-2**	VDAC3	0.289	voltage dependent anion channel 3
**Q9H9C1-1**	VIPAS39	-0.427	VPS33B interacting protein, apical-basolateral polarity regulator, spe-39 homolog
**Q69YN4**	VIRMA	0.199	vir like m6A methyltransferase associated
**Q9BRG1**	VPS25	-0.217	vacuolar protein sorting 25 homolog
**O75436**	VPS26A	-0.075	VPS26, retromer complex component A
**Q4G0F5**	VPS26B	-0.181	VPS26, retromer complex component B
**Q9UK41-1**	VPS28	-0.228	VPS28 subunit of ESCRT-I
**Q9UBQ0**	VPS29	-0.285	VPS29 retromer complex component
**Q96QK1**	VPS35	-0.303	VPS35 retromer complex component
**Q86VN1**	VPS36	-0.14	vacuolar protein sorting 36 homolog
**Q96JG6**	VPS50	-0.364	VPS50 subunit of EARP/GARPII complex
**Q9UID3-1**	VPS51	-0.102	VPS51 subunit of GARP complex
**Q5VIR6-4**	VPS53	-0.36	VPS53 subunit of GARP complex
**P62760**	VSNL1	-0.467	visinin like 1
**P23381**	WARS1	-0.375	tryptophanyl-tRNA synthetase 1
**Q9UGM6**	WARS2	0.285	tryptophanyl tRNA synthetase 2, mitochondrial
**Q9Y6W5**	WASF2	-0.097	WASP family member 2
**Q2M389**	WASHC4	-0.25	WASH complex subunit 4

**Q12768**	WASHC5	-0.357	WASH complex subunit 5
**Q9H6R7**	WDCP	-0.17	WD repeat and coiled coil containing
**O75717**	WDHD1	-0.174	WD repeat and HMG-box DNA binding protein 1
**Q9BZH6**	WDR11	-0.192	WD repeat domain 11
**Q9GZL7**	WDR12	-0.252	WD repeat domain 12
**Q9H7D7**	WDR26	0.102	WD repeat domain 26
**Q9UNX4**	WDR3	0.263	WD repeat domain 3
**Q9C0J8**	WDR33	-0.078	WD repeat domain 33
**Q8NI36**	WDR36	0.185	WD repeat domain 36
**Q9Y2I8-2**	WDR37	-0.232	WD repeat domain 37
**Q15061**	WDR43	0.171	WD repeat domain 43
**Q8TAF3-1**	WDR48	-0.105	WD repeat domain 48
**P61964**	WDR5	0.177	WD repeat domain 5
**Q9NW82**	WDR70	-0.257	WD repeat domain 70
**Q8IWA0**	WDR75	0.138	WD repeat domain 75
**Q9BQA1**	WDR77	-0.148	WD repeat domain 77
**Q562E7**	WDR81	-0.099	WD repeat domain 81
**A4D1P6**	WDR91	-0.205	WD repeat domain 91
**Q8N9V3**	WDSUB1	-0.355	WD repeat, sterile alpha motif and U-box domain containing 1
**O76024**	WFS1	-0.14	wolframin ER transmembrane glycoprotein
**Q9Y3S1**	WNK2	-1.031	WNK lysine deficient protein kinase 2
**Q9BUR4**	WRAP53	-0.155	WD repeat containing antisense to TP53
**Q15007**	WTAP	0.275	WT1 associated protein
**O00308**	WWP2	-0.601	WW domain containing E3 ubiquitin protein ligase 2
**Q9HCS7**	XAB2	0.195	XPA binding protein 2
**P98170**	XIAP	-0.504	X-linked inhibitor of apoptosis
**Q01831**	XPC	0.374	XPC complex subunit, DNA damage recognition and repair factor
**Q9NQW7-3**	XPNPEP1	-0.13	X-prolyl aminopeptidase 1
**O14980**	XPO1	-0.17	exportin 1
**Q9C0E2**	XPO4	-0.439	exportin 4
**Q9HAV4**	XPO5	-0.192	exportin 5
**Q9UIA9**	XPO7	-0.175	exportin 7
**O43592**	XPOT	-0.234	exportin for tRNA
**Q13426**	XRCC4	-0.371	X-ray repair cross complementing 4
**P13010**	XRCC5	-0.089	X-ray repair cross complementing 5
**O75191**	XYLB	-0.322	xylulokinase
**P46937-9**	YAP1	-0.142	Yes associated protein 1
**P54577**	YARS1	-0.275	tyrosyl-tRNA synthetase 1
**Q9Y2Z4**	YARS2	0.392	tyrosyl-tRNA synthetase 2
**P67809**	YBX1	-0.557	Y-box binding protein 1
**P16989**	YBX3	-0.325	Y-box binding protein 3
**O95619**	YEATS4	-0.182	YEATS domain containing 4
**P07947**	YES1	0.139	YES proto-oncogene 1, Src family tyrosine kinase
**Q5BJH7**	YIF1B	0.254	Yip1 interacting factor homolog B, membrane trafficking protein
**Q9BW85**	YJU2	-0.142	YJU2 splicing factor homolog
**O15498**	YKT6	0.343	YKT6 v-SNARE homolog
**Q96TA2-1**	YME1L1	0.275	YME1 like 1 ATPase

**Q86U90**	YRDC	0.34	yrdC N6-threonylcarbamoyltransferase domain containing
**Q96MU7-1**	YTHDC1	0.237	YTH domain containing 1
**Q9H6S0**	YTHDC2	-0.199	YTH domain containing 2
**Q9Y5A9**	YTHDF2	-0.343	YTH N6-methyladenosine RNA binding protein 2
**P62258**	YWHAE	-0.284	tyrosine 3-monooxygenase/tryptophan 5-monooxygenase activation protein epsilon
**P25490**	YY1	0.272	YY1 transcription factor
**Q8N4Q0**	ZADH2	0.298	zinc binding alcohol dehydrogenase domain containing 2
**O96006**	ZBED1	-0.527	zinc finger BED-type containing 1
**O95625**	ZBTB11	0.427	zinc finger and BTB domain containing 11
**O43829**	ZBTB14	-0.397	zinc finger and BTB domain containing 14
**Q96GY0**	ZC2HC1A	0.335	zinc finger C2HC-type containing 1A
**O75152**	ZC3H11A	0.428	zinc finger CCCH-type containing 11A
**Q8WU90**	ZC3H15	-0.339	zinc finger CCCH-type containing 15
**Q86VM9**	ZC3H18	0.095	zinc finger CCCH-type containing 18
**Q7Z2W4**	ZC3HAV1	0.318	zinc finger CCCH-type containing, antiviral 1
**Q86WB0**	ZC3HC1	-0.309	zinc finger C3HC-type containing 1
**Q9C0B9**	ZCCHC2	-0.253	zinc finger CCHC-type containing 2
**Q9C0B5**	ZDHHC5	0.159	zinc finger DHHC-type containing 5
**Q15911-1**	ZFHX3	-0.343	zinc finger homeobox 3
**Q07352**	ZFP36L1	0.778	ZFP36 ring finger protein like 1
**Q96KR1**	ZFR	-0.097	zinc finger RNA binding protein
**Q8N5A5**	ZGPAT	0.278	zinc finger CCCH-type and G-patch domain containing
**Q9Y6X8**	ZHX2	-0.238	zinc fingers and homeoboxes 2
**P17029**	ZKSCAN1	0.322	zinc finger with KRAB and SCAN domains 1
**O75844**	ZMPSTE24	0.205	zinc metallopeptidase STE24
**Q14202**	ZMYM3	-0.416	zinc finger MYM-type containing 3
**O75362**	ZNF217	0.086	zinc finger protein 217
**P17028-1**	ZNF24	0.241	zinc finger protein 24
**Q8ND82**	ZNF280C	0.335	zinc finger protein 280C
**Q9Y2X9**	ZNF281	0.244	zinc finger protein 281
**Q5BKZ1**	ZNF326	0.255	zinc finger protein 326
**Q9Y3S2**	ZNF330	0.398	zinc finger protein 330
**Q8TF68-2**	ZNF384	0.308	zinc finger protein 384
**Q92610**	ZNF592	0.133	zinc finger protein 592
**Q86UK7**	ZNF598	-0.178	zinc finger protein 598
**Q969S3**	ZNF622	0.48	zinc finger protein 622
**Q14966**	ZNF638	0.24	zinc finger protein 638
**Q9H582**	ZNF644	-0.223	zinc finger protein 644
**Q8N1G0**	ZNF687	0.346	zinc finger protein 687
**Q2TB10**	ZNF800	0.972	zinc finger protein 800
**Q9P2E3**	ZNFX1	0.337	zinc finger NFX1-type containing 1
**O75312**	ZPR1	-0.172	ZPR1 zinc finger
**O95218-1**	ZRANB2	-0.364	zinc finger RANBP2-type containing 2
**Q9H900-1**	ZWILCH	-0.19	zwilch kinetochore protein
**Q9C0D3**	ZYG11B	-0.153	zyg-11 family member B, cell cycle regulator
**O43149**	ZZEF1	-0.338	zinc finger ZZ-type and EF-hand domain containing 1
**F8W031**		0.189	

**H0YIV9**		-0.188	
**P00761**		0.493	
**P04908**		1.037	
**P30042**		0.233	
**P62158**		-0.362	
**P62805**		0.789	
**P69905**		1.653	
**P84243**		0.835	
**Q15269**		0.233	
**Q16637**		0.289	
**Q6FI13**		0.768	
**Q6NSW5**		-0.474	
**Q96JG8-4**		0.189	
**Q9BV94-1**		-0.396	
