# Supplementary material for: Elucidating the cellular response of silver nanoparticles as a potential combinatorial agent for cisplatin chemotherapy
Source: J Nanobiotechnology. 2020 Nov 10;18:164. doi: 10.1186/s12951-020-00719-x (PMC7654574; doi:10.1186/s12951-020-00719-x)
Supplement: Supplementary file 3 — Addiional file 3. List of proteins deregulated in THLE2 cells after 24 h exposure to CDDP and AgNP/CDDP. [file 12951_2020_719_MOESM3_ESM.pdf]

Elucidating the cellular response of silver nanoparticles as a potential combinatorial agent for cisplatin chemotherapy

Additional File 3

List of proteins deregulated in THLE2 cells after 24h exposure to CDDP 10 µM………………… pages 1 – 18
List of proteins deregulated in THLE2 cells after 24h exposure to AgNP 3.5 ug/mL + CDDP 10 µM ………………… pages 19 – 48

List of proteins deregulated in THLE2 cells after 24h exposure to CDDP 10 mM

ID	Symbol	Expr Log Ratio	Entrez Gene Name
**Q2M2I8**	AAK1	-0.214	AP2 associated kinase 1
**Q9NRN7**	AASDHPPT	0.23	aminoadipate-semialdehyde dehydrogenase-phosphopantetheinyl transferase
**P33897**	ABCD1	0.27	ATP binding cassette subfamily D member 1
**Q9NUJ1**	ABHD10	0.203	abhydrolase domain containing 10
**Q12979**	ABR	-0.213	ABR activator of RhoGEF and GTPase
**Q9ULW3**	ABT1	-0.231	activator of basal transcription 1
**P42765**	ACAA2	0.189	acetyl-CoA acyltransferase 2
**Q13085-4**	ACACA	-0.263	acetyl-CoA carboxylase alpha
**Q9UKV3-5**	ACIN1	0.152	apoptotic chromatin condensation inducer 1
**Q86TX2**	ACOT1	0.259	acyl-CoA thioesterase 1
**P11117**	ACP2	0.198	acid phosphatase 2, lysosomal
**P33121**	ACSL1	0.153	acyl-CoA synthetase long chain family member 1
**O43707**	ACTN4	-0.235	actinin alpha 4
**O14672**	ADAM10	-0.219	ADAM metallopeptidase domain 10
**P11766**	ADH5	0.196	alcohol dehydrogenase 5 (class III), chi polypeptide
**Q16186**	ADRM1	0.187	adhesion regulating molecule 1
**Q8N556-2**	AFAP1	-0.311	actin filament associated protein 1
**Q9UHB7-1**	AFF4	0.185	AF4/FMR2 family member 4
**O00468**	AGRN	-0.381	agrin
**Q9BRQ8**	AIFM2	0.274	apoptosis inducing factor mitochondria associated 2
**Q9UIJ7-1**	AK3	0.223	adenylate kinase 3
**P27144**	AK4	0.129	adenylate kinase 4
**Q9UKA4**	AKAP11	-0.233	A-kinase anchoring protein 11
**Q02952-1**	AKAP12	-0.274	A-kinase anchoring protein 12
**O43488**	AKR7A2	0.182	aldo-keto reductase family 7 member A2
**Q13740**	ALCAM	-0.225	activated leukocyte cell adhesion molecule
**P30837**	ALDH1B1	0.178	aldehyde dehydrogenase 1 family member B1
**P49189**	ALDH9A1	0.192	aldehyde dehydrogenase 9 family member A1
**Q86SJ2**	AMIGO2	-0.162	adhesion molecule with Ig like domain 2
**Q9BY76**	ANGPTL4	-0.664	angiopoietin like 4
**Q8IWZ3-6**	ANKHD1/ANKHD1-EIF4EBP3	-0.183	ankyrin repeat and KH domain containing 1
**Q9NQW6**	ANLN	0.349	anillin actin binding protein
**P58335-1**	ANTXR2	-0.688	ANTXR cell adhesion molecule 2
**P07355-2**	ANXA2	-0.233	annexin A2
**Q06481**	APLP2	-0.285	amyloid beta precursor like protein 2
**Q9NRW3**	APOBEC3C	0.41	apolipoprotein B mRNA editing enzyme catalytic subunit 3C
**P05067**	APP	-0.644	amyloid beta precursor protein
**Q96P48-6**	ARAP1	-0.184	ArfGAP with RhoGAP domain, ankyrin repeat and PH domain 1

**A1A4S6**	ARHGAP10	-0.387	Rho GTPase activating protein 10
**Q8N392**	ARHGAP18	-0.247	Rho GTPase activating protein 18
**O15013**	ARHGEF10	-0.257	Rho guanine nucleotide exchange factor 10
**Q9NZN5**	ARHGEF12	-0.198	Rho guanine nucleotide exchange factor 12
**Q8NFD5-3**	ARID1B	-0.481	AT-rich interaction domain 1B
**O43150-1**	ASAP2	-0.225	ArfGAP with SH3 domain, ankyrin repeat and PH domain 2
**Q8N9N2-2**	ASCC1	-0.605	activating signal cointegrator 1 complex subunit 1
**Q9H1I8-1**	ASCC2	-0.496	activating signal cointegrator 1 complex subunit 2
**Q8N3C0**	ASCC3	-0.55	activating signal cointegrator 1 complex subunit 3
**Q9NVP2**	ASF1B	0.261	anti-silencing function 1B histone chaperone
**O95352**	ATG7	-0.276	autophagy related 7
**O00244**	ATOX1	0.312	antioxidant 1 copper chaperone
**P05026**	ATP1B1	0.163	ATPase Na+/K+ transporting subunit beta 1
**P23634-1**	ATP2B4	-0.22	ATPase plasma membrane Ca2+ transporting 4
**P56381**	ATP5F1E	0.327	ATP synthase F1 subunit epsilon
**Q9UII2**	ATP5IF1	0.443	ATP synthase inhibitory factor subunit 1
**P0C7T5**	ATXN1L	0.311	ataxin 1 like
**Q96GD4-5**	AURKB	0.266	aurora kinase B
**P30530**	AXL	-0.357	AXL receptor tyrosine kinase
**P61769**	B2M	0.254	beta-2-microglobulin
**Q9NXR7-2**	BABAM2	-0.197	BRISC and BRCA1 A complex member 2
**O95816**	BAG2	0.175	BCL2 associated athanogene 2
**Q8WY36**	BBX	-0.39	BBX high mobility group box domain containing
**P51572-2**	BCAP31	0.162	B cell receptor associated protein 31
**O75815-1**	BCAR3	-0.739	BCAR3 adaptor protein, NSP family member
**O75934**	BCAS2	0.19	BCAS2 pre-mRNA processing factor
**Q9NYF8-1**	BCLAF1	0.283	BCL2 associated transcription factor 1
**Q9H694**	BICC1	-0.491	BicC family RNA binding protein 1
**Q9NR09**	BIRC6	-0.359	baculoviral IAP repeat containing 6
**Q14692**	BMS1	-0.248	BMS1 ribosome biogenesis factor
**Q7KYR7**	BTN2A1	0.406	butyrophilin subfamily 2 member A1
**Q8N5I9**	C12orf45	0.424	chromosome 12 open reading frame 45
**Q8N3J3**	C17orf53	0.361	chromosome 17 open reading frame 53
**Q9GZN8-2**	C20orf27	-0.191	chromosome 20 open reading frame 27
**Q9BVC5**	C2orf49	0.22	chromosome 2 open reading frame 49
**P01024**	C3	-0.342	complement C3
**P0C0L4-1**	C4A/C4B	-0.431	complement C4A (Rodgers blood group)
**Q49AR2**	C5orf22	0.417	chromosome 5 open reading frame 22
**Q9BRJ6**	C7orf50	-0.442	chromosome 7 open reading frame 50
**P40123**	CAP2	-0.132	cyclase associated actin cytoskeleton regulatory protein 2
**Q5VZK9**	CARMIL1	-0.329	capping protein regulator and myosin 1 linker 1
**O14936**	CASK	-0.213	calcium/calmodulin dependent serine protein kinase
**P29466**	CASP1	0.151	caspase 1
**P42575**	CASP2	0.283	caspase 2
**P42574**	CASP3	0.221	caspase 3
**P04040**	CAT	0.203	catalase
**O95810**	CAVIN2	0.278	caveolae associated protein 2

**P45973**	CBX5	0.258	chromobox 5
**Q8N163-1**	CCAR2	0.145	cell cycle and apoptosis regulator 2
**Q6PK04**	CCDC137	-0.395	coiled-coil domain containing 137
**Q7Z3E2**	CCDC186	0.233	coiled-coil domain containing 186
**Q9Y6R9**	CCDC61	-0.954	coiled-coil domain containing 61
**Q76M96-2**	CCDC80	-0.846	coiled-coil domain containing 80
**O00622**	CCN1	-0.881	cellular communication network factor 1
**P29279**	CCN2	-0.658	cellular communication network factor 2
**P20248**	CCNA2	0.279	cyclin A2
**P24385**	CCND1	-0.896	cyclin D1
**O95273-1**	CCNDBP1	0.766	cyclin D1 binding protein 1
**O60563**	CCNT1	0.369	cyclin T1
**O14618**	CCS	0.245	copper chaperone for superoxide dismutase
**P48643**	CCT5	0.148	chaperonin containing TCP1 subunit 5
**P40227**	CCT6A	0.163	chaperonin containing TCP1 subunit 6A
**Q6YHK3**	CD109	-0.188	CD109 molecule
**O95400**	CD2BP2	0.257	CD2 cytoplasmic tail binding protein 2
**P21926**	CD9	0.201	CD9 molecule
**Q12834**	CDC20	-0.196	cell division cycle 20
**O75419-3**	CDC45	0.35	cell division cycle 45
**O00311**	CDC7	0.35	cell division cycle 7
**Q96FF9**	CDCA5	0.806	cell division cycle associated 5
**P19022**	CDH2	-0.172	cadherin 2
**P06493**	CDK1	0.339	cyclin dependent kinase 1
**Q96SN8**	CDK5RAP2	-0.41	CDK5 regulatory subunit associated protein 2
**Q00534**	CDK6	-0.174	cyclin dependent kinase 6
**Q5VV42**	CDKAL1	-0.593	CDK5 regulatory subunit associated protein 1 like 1
**P46527**	CDKN1B	-0.701	cyclin dependent kinase inhibitor 1B
**Q8N726**	CDKN2A	-0.384	cyclin dependent kinase inhibitor 2A
**Q86X02**	CDR2L	0.453	cerebellar degeneration related protein 2 like
**Q02224**	CENPE	-0.279	centromere protein E
**Q7Z7K6-3**	CENPV	0.235	centromere protein V
**Q8TEP8-3**	CEP192	-0.303	centrosomal protein 192
**Q9Y5P4-3**	CERT1	-0.241	ceramide transporter 1
**O00748**	CES2	0.416	carboxylesterase 2
**P41208**	CETN2	0.231	centrin 2
**Q13112**	CHAF1B	0.38	chromatin assembly factor 1 subunit B
**Q96JM3**	CHAMP1	0.251	chromosome alignment maintaining phosphoprotein 1
**Q7LBR1**	CHMP1B	0.291	charged multivesicular body protein 1B
**Q9UHD1**	CHORDC1	0.249	cysteine and histidine rich domain containing 1
**P0CG13-1**	CHTF8	0.34	chromosome transmission fidelity factor 8
**Q9NZ45**	CISD1	0.25	CDGSH iron sulfur domain 1
**O14578-4**	CIT	-0.172	citron rho-interacting serine/threonine kinase
**Q8WWK9-5**	CKAP2	0.727	cytoskeleton associated protein 2
**Q8IYA6**	CKAP2L	0.175	cytoskeleton associated protein 2 like
**Q07065**	CKAP4	0.156	cytoskeleton associated protein 4
**P61024**	CKS1B	0.394	CDC28 protein kinase regulatory subunit 1B

**Q7Z460-4**	CLASP1	-0.22	cytoplasmic linker associated protein 1
**P49759-3**	CLK1	0.309	CDC like kinase 1
**Q9H6B4**	CLMP	-0.457	CXADR like membrane protein
**O75503**	CLN5	0.174	CLN5 intracellular trafficking protein
**Q92989**	CLP1	0.389	cleavage and polyadenylation factor I subunit 1
**Q9HAW4**	CLSPN	0.325	claspin
**O94985-1**	CLSTN1	-0.381	calsyntenin 1
**O75153**	CLUH	-0.169	clustered mitochondria homolog
**Q8NFW8**	CMAS	0.331	cytidine monophosphate N-acetylneuraminic acid synthetase
**Q96DG6**	CMBL	0.41	carboxymethylenebutenolidase homolog
**Q9BQ75**	CMSS1	-0.352	cms1 ribosomal small subunit homolog
**P09543-1**	CNP	0.327	2',3'-cyclic nucleotide 3' phosphodiesterase
**Q9NYJ1-2**	COA4	-0.199	cytochrome c oxidase assembly factor 4 homolog
**Q96BR5**	COA7	0.199	cytochrome c oxidase assembly factor 7 (putative)
**Q9UP83-2**	COG5	-0.338	component of oligomeric golgi complex 5
**P38432**	COIL	0.339	coilin
**P02452**	COL1A1	-0.554	collagen type I alpha 1 chain
**P02462**	COL4A1	-0.352	collagen type IV alpha 1 chain
**P08572**	COL4A2	-0.497	collagen type IV alpha 2 chain
**Q8NI60-1**	COQ8A	0.361	coenzyme Q8A
**P13073**	COX4I1	0.216	cytochrome c oxidase subunit 4I1
**P14406**	COX7A2	0.298	cytochrome c oxidase subunit 7A2
**P15954**	COX7C	0.214	cytochrome c oxidase subunit 7C
**O75976**	CPD	-0.149	carboxypeptidase D
**Q99829**	CPNE1	0.191	copine 1
**P31327-3**	CPS1	-0.246	carbamoyl-phosphate synthase 1
**Q8N684-3**	CPSF7	0.161	cleavage and polyadenylation specific factor 7
**P23786**	CPT2	0.296	carnitine palmitoyltransferase 2
**P43155**	CRAT	0.211	carnitine O-acetyltransferase
**O75629**	CREG1	0.189	cellular repressor of E1A stimulated genes 1
**Q9NZV1**	CRIM1	-0.512	cysteine rich transmembrane BMP regulator 1
**P52943**	CRIP2	0.257	cysteine rich protein 2
**O75534-4**	CSDE1	-0.199	cold shock domain containing E1
**Q16527**	CSRP2	0.286	cysteine and glycine rich protein 2
**Q05048**	CSTF1	0.163	cleavage stimulation factor subunit 1
**O43310-2**	CTIF	-0.418	cap binding complex dependent translation initiation factor
**P35222**	CTNNB1	-0.215	catenin beta 1
**P07339**	CTSD	0.269	cathepsin D
**P42830**	CXCL5	-0.647	C-X-C motif chemokine ligand 5
**Q9UHQ9**	CYB5R1	0.213	cytochrome b5 reductase 1
**Q9NV06**	DCAF13	-0.28	DDB1 and CUL4 associated factor 13
**Q58WW2**	DCAF6	-0.653	DDB1 and CUL4 associated factor 6
**Q96PD2-2**	DCBLD2	-0.313	discoidin, CUB and LCCL domain containing 2
**Q7Z4W1**	DCXR	0.19	dicarbonyl and L-xylulose reductase
**Q92466**	DDB2	0.489	damage specific DNA binding protein 2
**Q13206**	DDX10	-0.262	DEAD-box helicase 10
**Q92841**	DDX17	-0.151	DEAD-box helicase 17

**Q9H8H2**	DDX31	-0.276	DEAD-box helicase 31
**Q9UJV9**	DDX41	0.167	DEAD-box helicase 41
**Q9Y6V7**	DDX49	-0.271	DEAD-box helicase 49
**P17844**	DDX5	-0.156	DEAD-box helicase 5
**Q9NY93**	DDX56	-0.522	DEAD-box helicase 56
**O43583**	DENR	0.321	density regulated re-initiation and release factor
**Q8WYQ5-1**	DGCR8	0.336	DGCR8 microprocessor complex subunit
**Q6UWP2**	DHRS11	0.291	dehydrogenase/reductase 11
**Q8IY37**	DHX37	-0.144	DEAH-box helicase 37
**Q6P158-1**	DHX57	-0.233	DExH-box helicase 57
**O60879**	DIAPH2	-0.343	diaphanous related formin 2
**Q9NSV4**	DIAPH3	-0.507	diaphanous related formin 3
**Q9UNQ2**	DIMT1	-0.232	DIMT1 rRNA methyltransferase and ribosome maturation factor
**Q9P265**	DIP2B	-0.268	disco interacting protein 2 homolog B
**Q8IYB7**	DIS3L2	-0.256	DIS3 like 3'-5' exoribonuclease 2
**O94907**	DKK1	-0.405	dickkopf WNT signaling pathway inhibitor 1
**P09622**	DLD	0.228	dihydrolipoamide dehydrogenase
**Q86Y56**	DNAAF5	-0.234	dynein axonemal assembly factor 5
**P31689**	DNAJA1	0.182	DnaJ heat shock protein family (Hsp40) member A1
**P25685**	DNAJB1	0.23	DnaJ heat shock protein family (Hsp40) member B1
**Q96KC8**	DNAJC1	-0.177	DnaJ heat shock protein family (Hsp40) member C1
**Q96DA6**	DNAJC19	0.3	DnaJ heat shock protein family (Hsp40) member C19
**Q8WXX5**	DNAJC9	0.24	DnaJ heat shock protein family (Hsp40) member C9
**Q6XZF7**	DNMBP	-0.258	dynamin binding protein
**P26358-1**	DNMT1	-0.218	DNA methyltransferase 1
**Q8N1I0-3**	DOCK4	-0.363	dedicator of cytokinesis 4
**Q96N67**	DOCK7	-0.187	dedicator of cytokinesis 7
**Q9BZ29-4**	DOCK9	-0.258	dedicator of cytokinesis 9
**Q14919-2**	DRAP1	0.212	DR1 associated protein 1
**P15924-1**	DSP	-0.202	desmoplakin
**Q03001-7**	DST	-0.363	dystonin
**Q9BVJ7**	DUSP23	-0.155	dual specificity phosphatase 23
**Q92997**	DVL3	0.256	dishevelled segment polarity protein 3
**O75923-13**	DYSF	-0.316	dysferlin
**P42892**	ECE1	-0.175	endothelin converting enzyme 1
**Q13011**	ECH1	0.222	enoyl-CoA hydratase 1
**O75521**	ECI2	0.229	enoyl-CoA delta isomerase 2
**Q9BZQ6**	EDEM3	-0.217	ER degradation enhancing alpha-mannosidase like protein 3
**P29692-2**	EEF1D	0.256	eukaryotic translation elongation factor 1 delta
**O00418**	EEF2K	-0.577	eukaryotic elongation factor 2 kinase
**P57772**	EEFSEC	-0.2	eukaryotic elongation factor, selenocysteine-tRNA specific
**Q12805**	EFEMP1	-0.478	EGF containing fibulin extracellular matrix protein 1
**P98172**	EFNB1	0.271	ephrin B1
**P52799**	EFNB2	-0.247	ephrin B2
**P00533-1**	EGFR	-0.531	epidermal growth factor receptor
**Q8N3D4**	EHBP1L1	-0.196	EH domain binding protein 1 like 1
**Q9H9B1**	EHMT1	-0.328	euchromatic histone lysine methyltransferase 1

**P19525**	EIF2AK2	0.163	eukaryotic translation initiation factor 2 alpha kinase 2
**P05198**	EIF2S1	0.143	eukaryotic translation initiation factor 2 subunit alpha
**P38919**	EIF4A3	0.245	eukaryotic translation initiation factor 4A3
**O43432-3**	EIF4G3	-0.218	eukaryotic translation initiation factor 4 gamma 3
**P11171-1**	EPB41	-0.31	erythrocyte membrane protein band 4.1
**O43491**	EPB41L2	-0.126	erythrocyte membrane protein band 4.1 like 2
**Q9UM22**	EPDR1	0.518	ependymin related 1
**Q9HCE0-1**	EPG5	-0.218	ectopic P-granules autophagy protein 5 homolog
**P29317**	EPHA2	0.259	EPH receptor A2
**P29323-3**	EPHB2	-0.397	EPH receptor B2
**P07099**	EPHX1	0.249	epoxide hydrolase 1
**Q8IUD2-1**	ERC1	-0.372	ELKS/RAB6-interacting/CAST family member 1
**O60447-2**	EVI5	-0.328	ecotropic viral integration site 5
**Q96A65**	EXOC4	-0.383	exocyst complex component 4
**Q9Y2D4**	EXOC6B	-0.376	exocyst complex component 6B
**Q9NQT5**	EXOSC3	0.203	exosome component 3
**Q06265-2**	EXOSC9	0.167	exosome component 9
**Q93063-3**	EXT2	-0.23	exostosin glycosyltransferase 2
**P00734**	F2	-0.848	coagulation factor II, thrombin
**P13726**	F3	-0.386	coagulation factor III, tissue factor
**O95864**	FADS2	-0.314	fatty acid desaturase 2
**Q9UNN5**	FAF1	-0.473	Fas associated factor 1
**Q6SJ93**	FAM111B	0.559	family with sequence similarity 111 member B
**Q9NSI2**	FAM207A	-0.323	family with sequence similarity 207 member A
**Q9H4H8**	FAM83D	0.347	family with sequence similarity 83 member D
**Q8WVX9**	FAR1	-0.287	fatty acyl-CoA reductase 1
**O95363**	FARS2	-0.26	phenylalanyl-tRNA synthetase 2, mitochondrial
**P25445**	FAS	0.527	Fas cell surface death receptor
**P49327**	FASN	-0.17	fatty acid synthase
**P23142**	FBLN1	-0.43	fibulin 1
**P35555**	FBN1	-0.584	fibrillin 1
**Q8N531-1**	FBXL6	0.541	F-box and leucine rich repeat protein 6
**Q8NEZ5**	FBXO22	0.199	F-box protein 22
**Q9Y324**	FCF1	-0.292	FCF1 rRNA-processing protein
**O94868**	FCHSD2	-0.638	FCH and double SH3 domains 2
**P37268**	FDFT1	-0.364	farnesyl-diphosphate farnesyltransferase 1
**P22570-3**	FDXR	0.47	ferredoxin reductase
**P22830-2**	FECH	0.219	ferrochelatase
**P39748**	FEN1	0.327	flap structure-specific endonuclease 1
**Q9UHY8-2**	FEZ2	-0.439	fasciculation and elongation protein zeta 2
**P02679**	FGG	-0.665	fibrinogen gamma chain
**Q2V2M9-4**	FHOD3	-0.386	formin homology 2 domain containing 3
**Q4L180**	FILIP1L	0.63	filamin A interacting protein 1 like
**Q14254**	FLOT2	0.208	flotillin 2
**Q96CP2**	FLYWCH2	0.236	FLYWCH family member 2
**Q96PY5-3**	FMNL2	-0.642	formin like 2
**P02751**	FN1	-0.763	fibronectin 1

**Q96RU3-1**	FNBP1	-0.347	formin binding protein 1
**Q53EP0**	FNDC3B	-0.563	fibronectin type III domain containing 3B
**Q5VW36**	FOCAD	-0.155	focadhesin
**P85037**	FOXK1	-0.2	forkhead box K1
**Q9P2Q2**	FRMD4A	-0.37	FERM domain containing 4A
**Q9BZ67**	FRMD8	0.262	FERM domain containing 8
**Q12841**	FSTL1	-0.425	follistatin like 1
**P02792**	FTL	0.467	ferritin light chain
**Q9C0B1-1**	FTO	-0.293	FTO alpha-ketoglutarate dependent dioxygenase
**P04066**	FUCA1	0.319	alpha-L-fucosidase 1
**Q9BTY2**	FUCA2	0.235	alpha-L-fucosidase 2
**Q9BYC5**	FUT8	-0.217	fucosyltransferase 8
**Q9BQS8-4**	FYCO1	-0.393	FYVE and coiled-coil domain containing 1
**P10253**	GAA	0.226	glucosidase alpha, acid
**Q86SR1**	GALNT10	-0.255	polypeptide N-acetylgalactosaminyltransferase 10
**Q10471**	GALNT2	-0.158	polypeptide N-acetylgalactosaminyltransferase 2
**Q14435**	GALNT3	-0.211	polypeptide N-acetylgalactosaminyltransferase 3
**Q86SF2**	GALNT7	-0.198	polypeptide N-acetylgalactosaminyltransferase 7
**Q92538**	GBF1	-0.252	golgi brefeldin A resistant guanine nucleotide exchange factor 1
**Q99988**	GDF15	0.73	growth differentiation factor 15
**P17302**	GJA1	-0.278	gap junction protein alpha 1
**P32189-3**	GK	0.145	glycerol kinase
**P06280**	GLA	0.316	galactosidase alpha
**Q92896-2**	GLG1	-0.203	golgi glycoprotein 1
**Q04760-1**	GLO1	0.231	glyoxalase I
**O76003**	GLRX3	-0.289	glutaredoxin 3
**P00367**	GLUD1	0.155	glutamate dehydrogenase 1
**P15104**	GLUL	0.415	glutamate-ammonia ligase
**P17900**	GM2A	0.338	GM2 ganglioside activator
**O60547**	GMDS	-0.275	GDP-mannose 4,6-dehydratase
**O75496**	GMNN	0.485	geminin DNA replication inhibitor
**Q14344**	GNA13	0.233	G protein subunit alpha 13
**P50148**	GNAQ	-0.253	G protein subunit alpha q
**P62879**	GNB2	0.206	G protein subunit beta 2
**P61952**	GNG11	0.725	G protein subunit gamma 11
**P63218**	GNG5	0.357	G protein subunit gamma 5
**Q13823**	GNL2	-0.157	G protein nucleolar 2
**Q9BVP2**	GNL3	-0.324	G protein nucleolar 3
**P15586**	GNS	0.172	glucosamine (N-acetyl)-6-sulfatase
**P35052**	GPC1	0.506	glypican 1
**Q9Y625**	GPC6	-0.686	glypican 6
**Q9NQX3-2**	GPHN	-0.311	gephyrin
**Q8NFJ5**	GPRC5A	0.327	G protein-coupled receptor class C group 5 member A
**Q13322-1**	GRB10	-0.526	growth factor receptor bound protein 10
**P62993**	GRB2	-0.339	growth factor receptor bound protein 2
**O60565**	GREM1	-0.526	gremlin 1, DAN family BMP antagonist
**Q8NEC7-1**	GSTCD	-0.251	glutathione S-transferase C-terminal domain containing

**P28161**	GSTM2	0.198	glutathione S-transferase mu 2
**P78417**	GSTO1	0.194	glutathione S-transferase omega 1
**P09211**	GSTP1	0.222	glutathione S-transferase pi 1
**P13984**	GTF2F2	-0.285	general transcription factor IIF subunit 2
**Q9BZE4**	GTPBP4	-0.212	GTP binding protein 4
**Q9NYZ3**	GTSE1	0.301	G2 and S-phase expressed 1
**P0C0S5**	H2AZ1	0.208	H2A.Z variant histone 1
**P40939**	HADHA	0.219	hydroxyacyl-CoA dehydrogenase trifunctional multienzyme complex subunit alpha
**P55084**	HADHB	0.169	hydroxyacyl-CoA dehydrogenase trifunctional multienzyme complex subunit beta
**O14929**	HAT1	0.197	histone acetyltransferase 1
**Q00341**	HDLBP	-0.217	high density lipoprotein binding protein
**Q5T447**	HECTD3	-0.171	HECT domain E3 ubiquitin protein ligase 3
**P07686**	HEXB	0.217	hexosaminidase subunit beta
**O94992**	HEXIM1	0.569	HEXIM P-TEFb complex subunit 1
**P04439**	HLA-A	0.188	major histocompatibility complex, class I, A
**P01889**	HLA-B	0.289	major histocompatibility complex, class I, B
**P30481**	HLA-B	0.331	major histocompatibility complex, class I, B
**P20039**	HLA-DRB1	0.239	major histocompatibility complex, class II, DR beta 1
**Q14527**	HLTF	-0.337	helicase like transcription factor
**P09429**	HMGB1	0.278	high mobility group box 1
**P35914**	HMGCL	0.228	3-hydroxy-3-methylglutaryl-CoA lyase
**P05204**	HMGN2	0.302	high mobility group nucleosomal binding domain 2
**O00479**	HMGN4	0.308	high mobility group nucleosomal binding domain 4
**O75330-3**	HMMR	0.215	hyaluronan mediated motility receptor
**P09601**	HMOX1	-0.177	heme oxygenase 1
**Q1KMD3**	HNRNPUL2	0.195	heterogeneous nuclear ribonucleoprotein U like 2
**P0DMV9**	HSPA1A/HSPA1B	0.236	heat shock protein family A (Hsp70) member 1A
**P54652**	HSPA2	0.287	heat shock protein family A (Hsp70) member 2
**O95757**	HSPA4L	0.24	heat shock protein family A (Hsp70) member 4 like
**P61604**	HSPE1	0.351	heat shock protein family E (Hsp10) member 1
**P98160**	HSPG2	-0.419	heparan sulfate proteoglycan 2
**Q9BUP3-3**	HTATIP2	0.269	HIV-1 Tat interactive protein 2
**Q92743**	HTRA1	-0.527	HtrA serine peptidase 1
**Q7Z6Z7**	HUWE1	-0.248	HECT, UBA and WWE domain containing E3 ubiquitin protein ligase 1
**P50213**	IDH3A	0.148	isocitrate dehydrogenase (NAD(+)) 3 catalytic subunit alpha
**Q16666-1**	IFI16	0.205	interferon gamma inducible protein 16
**P80217-2**	IFI35	0.23	interferon induced protein 35
**P09914**	IFIT1	0.243	interferon induced protein with tetratricopeptide repeats 1
**P09913**	IFIT2	0.446	interferon induced protein with tetratricopeptide repeats 2
**O14879**	IFIT3	0.536	interferon induced protein with tetratricopeptide repeats 3
**Q9NZI8**	IGF2BP1	-0.205	insulin like growth factor 2 mRNA binding protein 1
**Q9Y6M1**	IGF2BP2	-0.238	insulin like growth factor 2 mRNA binding protein 2
**P11717**	IGF2R	-0.278	insulin like growth factor 2 receptor
**P40189**	IL6ST	-0.208	interleukin 6 signal transducer
**P29218-3**	IMPA1	0.226	inositol monophosphatase 1
**Q9NQS7**	INCENP	0.2	inner centromere protein
**Q6DN90**	IQSEC1	-0.34	IQ motif and Sec7 domain ArfGEF 1

**Q9Y616**	IRAK3	-0.265	interleukin 1 receptor associated kinase 3
**Q8IU81**	IRF2BP1	0.184	interferon regulatory factor 2 binding protein 1
**Q9H1B7**	IRF2BPL	0.394	interferon regulatory factor 2 binding protein like
**P35568**	IRS1	-0.203	insulin receptor substrate 1
**Q9BUE6-2**	ISCA1	0.297	iron-sulfur cluster assembly 1
**Q9H9L3**	ISG20L2	-0.46	interferon stimulated exonuclease gene 20 like 2
**Q96J02**	ITCH	-0.24	itchy E3 ubiquitin protein ligase
**P26006**	ITGA3	-0.236	integrin subunit alpha 3
**P18084**	ITGB5	-0.186	integrin subunit beta 5
**P19827**	ITIH1	-0.674	inter-alpha-trypsin inhibitor heavy chain 1
**Q14571**	ITPR2	-0.325	inositol 1,4,5-trisphosphate receptor type 2
**Q9Y6Y0**	IVNS1ABP	-0.252	influenza virus NS1A binding protein
**P23458**	JAK1	-0.309	Janus kinase 1
**O95251-1**	KAT7	0.278	lysine acetyltransferase 7
**Q9Y2K7**	KDM2A	0.527	lysine demethylase 2A
**Q92945**	KHSRP	0.137	KH-type splicing regulatory protein
**Q5T5P2-1**	KIAA1217	-0.189	KIAA1217
**Q9BY89-1**	KIAA1671	-0.559	KIAA1671
**Q9ULH0-1**	KIDINS220	-0.263	kinase D interacting substrate 220
**P52732**	KIF11	0.32	kinesin family member 11
**Q9H1H9**	KIF13A	-0.299	kinesin family member 13A
**Q9NQT8**	KIF13B	-0.262	kinesin family member 13B
**O95235**	KIF20A	0.163	kinesin family member 20A
**Q14807**	KIF22	0.313	kinesin family member 22
**Q99661**	KIF2C	0.315	kinesin family member 2C
**Q96EK5**	KIFBP	0.165	kinesin family binding protein
**Q9BW19**	KIFC1	0.232	kinesin family member C1
**Q9NSK0-3**	KLC4	0.201	kinesin light chain 4
**P50748**	KNTC1	-0.163	kinetochore associated 1
**Q14533**	KRT81	-0.703	keratin 81
**O15230**	LAMA5	-0.617	laminin subunit alpha 5
**P07942**	LAMB1	-0.345	laminin subunit beta 1
**P11047**	LAMC1	-0.35	laminin subunit gamma 1
**Q13753**	LAMC2	-0.341	laminin subunit gamma 2
**Q6IAA8**	LAMTOR1	0.137	late endosomal/lysosomal adaptor, MAPK and MTOR activator 1
**P01130**	LDLR	-0.526	low density lipoprotein receptor
**Q99538**	LGMN	0.156	legumain
**Q9UPQ0**	LIMCH1	-0.459	LIM and calponin homology domains 1
**Q86WA8**	LONP2	-0.277	lon peptidase 2, peroxisomal
**Q9Y4K0**	LOXL2	-0.585	lysyl oxidase like 2
**Q93052**	LPP	-0.31	LIM domain containing preferred translocation partner in lipoma
**P50851-2**	LRBA	-0.327	LPS responsive beige-like anchor protein
**Q9Y2L9-3**	LRCH1	-0.218	leucine rich repeats and calponin homology domain containing 1
**Q96II8-2**	LRCH3	-0.187	leucine rich repeats and calponin homology domain containing 3
**Q07954**	LRP1	-0.366	LDL receptor related protein 1
**Q6UWE0**	LRSAM1	-0.249	leucine rich repeat and sterile alpha motif containing 1
**Q3MHD2-2**	LSM12	0.164	LSM12 homolog

**Q9BX40**	LSM14B	0.309	LSM family member 14B
**Q14766-4**	LTBP1	-0.705	latent transforming growth factor beta binding protein 1
**Q9Y383**	LUC7L2	0.247	LUC7 like 2, pre-mRNA splicing factor
**O95232**	LUC7L3	0.333	LUC7 like 3 pre-mRNA splicing factor
**P07948-1**	LYN	-0.255	LYN proto-oncogene, Src family tyrosine kinase
**O95372**	LYPLA2	0.219	lysophospholipase 2
**Q9UPN3**	MACF1	-0.307	microtubule actin crosslinking factor 1
**O75367**	MACROH2A1	0.167	macroH2A.1 histone
**Q9Y6D9**	MAD1L1	-0.247	mitotic arrest deficient 1 like 1
**Q8WXG6-4**	MADD	0.172	MAP kinase activating death domain
**Q9ULX9**	MAFF	0.617	MAF bZIP transcription factor F
**P46821**	MAP1B	-0.172	microtubule associated protein 1B
**Q9Y6R4-1**	MAP3K4	-0.2	mitogen-activated protein kinase kinase kinase 4
**P27816-1**	MAP4	-0.207	microtubule associated protein 4
**Q8IVH8**	MAP4K3	-0.232	mitogen-activated protein kinase kinase kinase kinase 3
**Q16644**	MAPKAPK3	-0.169	MAPK activated protein kinase 3
**P49006**	MARCKSL1	0.352	MARCKS like 1
**Q96GX5**	MASTL	0.427	microtubule associated serine/threonine kinase like
**Q9NZL9**	MAT2B	0.195	methionine adenosyltransferase 2B
**P43121**	MCAM	0.175	melanoma cell adhesion molecule
**P40926**	MDH2	0.249	malate dehydrogenase 2
**Q9NU22**	MDN1	-0.149	midasin AAA ATPase 1
**Q9H944**	MED20	0.237	mediator complex subunit 20
**Q7L2J0-1**	MEPCE	0.227	methylphosphate capping enzyme
**Q86W50-1**	METTL16	-0.181	methyltransferase like 16
**Q9H8H3**	METTL7A	0.168	methyltransferase like 7A
**Q99685**	MGLL	-0.368	monoglyceride lipase
**Q9BQP7**	MGME1	0.479	mitochondrial genome maintenance exonuclease 1
**O94851-4**	MICAL2	-0.314	microtubule associated monooxygenase, calponin and LIM domain containing 2
**Q7RTP6**	MICAL3	-0.277	microtubule associated monooxygenase, calponin and LIM domain containing 3
**Q9BPX6-3**	MICU1	-0.187	mitochondrial calcium uptake 1
**Q99797**	MIPEP	-0.219	mitochondrial intermediate peptidase
**Q9UL63-1**	MKLN1	-0.217	muskelin 1
**Q13015**	MLLT11	0.238	MLLT11 transcription factor 7 cofactor
**P50281**	MMP14	-0.212	matrix metallopeptidase 14
**Q7Z3U7**	MON2	-0.182	MON2 homolog, regulator of endosome-to-Golgi trafficking
**Q14149**	MORC3	0.165	MORC family CW-type zinc finger 3
**O00566**	MPHOSPH10	0.152	M-phase phosphoprotein 10
**Q8N3R9-1**	MPP5	-0.172	membrane palmitoylated protein 5
**Q9UBG0**	MRC2	-0.186	mannose receptor C type 2
**Q9HC36**	MRM3	0.213	mitochondrial rRNA methyltransferase 3
**Q86TS9**	MRPL52	0.197	mitochondrial ribosomal protein L52
**P82912**	MRPS11	0.198	mitochondrial ribosomal protein S11
**O15235**	MRPS12	0.275	mitochondrial ribosomal protein S12
**P82932**	MRPS6	0.165	mitochondrial ribosomal protein S6
**Q6UB35-1**	MTHFD1L	-0.257	methylenetetrahydrofolate dehydrogenase (NADP+ dependent) 1 like
**P42345**	MTOR	-0.17	mechanistic target of rapamycin kinase

**Q9BQG0-2**	MYBBP1A	-0.266	MYB binding protein 1a
**O75592-1**	MYCBP2	-0.404	MYC binding protein 2
**P24844-1**	MYL9	-0.559	myosin light chain 9
**Q12965**	MYO1E	-0.253	myosin IE
**Q13459**	MYO9B	-0.25	myosin IXB
**Q9NZM1**	MYOF	-0.174	myoferlin
**Q9Y2G1**	MYRF	-0.458	myelin regulatory factor
**E9PAV3**	NACA	-0.187	nascent polypeptide associated complex subunit alpha
**Q8NEY1**	NAV1	-0.46	neuron navigator 1
**A2RRP1**	NBAS	-0.292	NBAS subunit of NRZ tethering complex
**Q9UGV2**	NDRG3	-0.265	NDRG family member 3
**O95299**	NDUFA10	0.164	NADH:ubiquinone oxidoreductase subunit A10
**Q16718**	NDUFA5	0.154	NADH:ubiquinone oxidoreductase subunit A5
**P56556**	NDUFA6	0.189	NADH:ubiquinone oxidoreductase subunit A6
**O95182**	NDUFA7	0.163	NADH:ubiquinone oxidoreductase subunit A7
**Q9Y6M9**	NDUFB9	0.185	NADH:ubiquinone oxidoreductase subunit B9
**P49821**	NDUFV1	0.138	NADH:ubiquinone oxidoreductase core subunit V1
**P46934-4**	NEDD4	-0.241	NEDD4 E3 ubiquitin protein ligase
**Q96PU5**	NEDD4L	-0.254	NEDD4 like E3 ubiquitin protein ligase
**Q8IXH7**	NELFCD	0.161	negative elongation factor complex member C/D
**P18615**	NELFE	0.161	negative elongation factor complex member E
**Q96IV0**	NGLY1	-0.271	N-glycanase 1
**Q9BZQ8**	NIBAN1	-0.216	niban apoptosis regulator 1
**Q9BYG3**	NIFK	-0.298	nucleolar protein interacting with the FHA domain of MKI67
**Q9BPW8**	NIPSNAP1	0.228	nipsnap homolog 1
**Q9UFN0**	NIPSNAP3A	0.162	nipsnap homolog 3A
**Q96D46**	NMD3	0.155	NMD3 ribosome export adaptor
**P30419**	NMT1	0.142	N-myristoyltransferase 1
**Q9BVI4**	NOC4L	-0.191	nucleolar complex associated 4 homolog
**Q9H6R4-1**	NOL6	-0.181	nucleolar protein 6
**P78316**	NOP14	-0.182	NOP14 nucleolar protein
**Q9Y3C1**	NOP16	-0.254	NOP16 nucleolar protein
**Q9NZM5**	NOP53	-0.585	NOP53 ribosome biogenesis factor
**P15559-1**	NQO1	0.201	NAD(P)H quinone dehydrogenase 1
**P16083**	NQO2	0.225	N-ribosyldihydronicotinamide:quinone reductase 2
**O14786-1**	NRP1	-0.465	neuropilin 1
**O95478**	NSA2	-0.595	NSA2 ribosome biogenesis factor
**Q9UNZ2**	NSFL1C	0.157	NSFL1 cofactor
**P49757**	NUMB	-0.299	NUMB endocytic adaptor protein
**Q9BXS6**	NUSAP1	-0.318	nucleolar and spindle associated protein 1
**Q9UBU9-1**	NXF1	0.291	nuclear RNA export factor 1
**P04181**	OAT	0.162	ornithine aminotransferase
**Q8N543**	OGFOD1	0.23	2-oxoglutarate and iron dependent oxygenase domain containing 1
**Q9NZT2**	OGFR	0.167	opioid growth factor receptor
**Q9BZF3-5**	OSBPL6	-0.264	oxysterol binding protein like 6
**P55809**	OXCT1	0.146	3-oxoacid CoA-transferase 1
**Q8N573-1**	OXR1	-0.282	oxidation resistance 1

**Q8IVL5**	P3H2	-0.423	prolyl 3-hydroxylase 2
**Q6VY07**	PACS1	-0.27	phosphofurin acidic cluster sorting protein 1
**Q8N7H5**	PAF1	0.233	PAF1 homolog, Paf1/RNA polymerase II complex component
**Q8TEW0**	PARD3	-0.386	par-3 family cell polarity regulator
**O95453**	PARN	-0.303	poly(A)-specific ribonuclease
**Q9UKK3**	PARP4	-0.129	poly(ADP-ribose) polymerase family member 4
**Q8IXQ6**	PARP9	0.194	poly(ADP-ribose) polymerase family member 9
**Q9HBI1**	PARVB	-0.278	parvin beta
**Q9Y5B6**	PAXBP1	0.301	PAX3 and PAX7 binding protein 1
**Q15004**	PCLAF	0.391	PCNA clamp associated factor
**P12004**	PCNA	0.219	proliferating cell nuclear antigen
**Q14690**	PDCD11	-0.262	programmed cell death 11
**Q53EL6**	PDCD4	0.339	programmed cell death 4
**Q96HC4**	PDLIM5	-0.205	PDZ and LIM domain 5
**Q9BRX2**	PELO	0.188	pelota mRNA surveillance and ribosome rescue factor
**Q9UHV9**	PFDN2	0.181	prefoldin subunit 2
**Q9NQP4**	PFDN4	0.213	prefoldin subunit 4
**Q99471**	PFDN5	0.22	prefoldin subunit 5
**P00558**	PGK1	0.154	phosphoglycerate kinase 1
**P35232**	PHB	0.214	prohibitin
**Q9BTU6**	PI4K2A	0.178	phosphatidylinositol 4-kinase type 2 alpha
**P42356**	PI4KA	-0.174	phosphatidylinositol 4-kinase alpha
**O43164**	PJA2	-0.259	praja ring finger ubiquitin ligase 2
**Q99640**	PKMYT1	0.453	protein kinase, membrane associated tyrosine/threonine 1
**P47712**	PLA2G4A	-0.254	phospholipase A2 group IVA
**Q13393-1**	PLD1	-0.259	phospholipase D1
**Q9HAU0-6**	PLEKHA5	-0.222	pleckstrin homology domain containing A5
**P00747**	PLG	-0.357	plasminogen
**Q99541**	PLIN2	-0.155	perilipin 2
**O75051**	PLXNA2	-0.459	plexin A2
**Q9NRX1**	PNO1	-0.508	partner of NOB1 homolog
**Q9H488**	POFUT1	0.146	protein O-fucosyltransferase 1
**Q8NBL1**	POGLUT1	0.138	protein O-glucosyltransferase 1
**P09884**	POLA1	-0.275	DNA polymerase alpha 1, catalytic subunit
**P36954**	POLR2I	-0.358	RNA polymerase II subunit I
**Q9UNP9**	PPIE	0.293	peptidylprolyl isomerase E
**P30405**	PPIF	0.335	peptidylprolyl isomerase F
**Q96QC0**	PPP1R10	0.165	protein phosphatase 1 regulatory subunit 10
**Q12972**	PPP1R8	0.276	protein phosphatase 1 regulatory subunit 8
**Q08209**	PPP3CA	-0.301	protein phosphatase 3 catalytic subunit alpha
**Q9NY27**	PPP4R2	0.136	protein phosphatase 4 regulatory subunit 2
**P50897**	PPT1	0.267	palmitoyl-protein thioesterase 1
**O60828**	PQBP1	0.242	polyglutamine binding protein 1
**O43663**	PRC1	0.278	protein regulator of cytokinesis 1
**Q06830**	PRDX1	0.24	peroxiredoxin 1
**P49642**	PRIM1	-0.216	DNA primase subunit 1
**P49643**	PRIM2	-0.268	DNA primase subunit 2

**Q13131-2**	PRKAA1	0.206	protein kinase AMP-activated catalytic subunit alpha 1
**Q9Y478**	PRKAB1	0.379	protein kinase AMP-activated non-catalytic subunit beta 1
**P17612**	PRKACA	-0.217	protein kinase cAMP-activated catalytic subunit alpha
**P22694**	PRKACB	-0.546	protein kinase cAMP-activated catalytic subunit beta
**P10644**	PRKAR1A	-0.292	protein kinase cAMP-dependent type I regulatory subunit alpha
**O15091**	PRORP	-0.295	protein only RNase P catalytic subunit
**Q9UMS4**	PRPF19	0.194	pre-mRNA processing factor 19
**O43172**	PRPF4	0.157	pre-mRNA processing factor 4
**P25788**	PSMA3	0.223	proteasome subunit alpha 3
**P25789**	PSMA4	0.171	proteasome subunit alpha 4
**P28066**	PSMA5	0.145	proteasome subunit alpha 5
**O14818-1**	PSMA7	0.152	proteasome subunit alpha 7
**P28074-1**	PSMB5	0.171	proteasome subunit beta 5
**Q99436**	PSMB7	0.157	proteasome subunit beta 7
**P35998**	PSMC2	0.217	proteasome 26S subunit, ATPase 2
**P17980**	PSMC3	0.183	proteasome 26S subunit, ATPase 3
**P43686**	PSMC4	0.167	proteasome 26S subunit, ATPase 4
**P62333**	PSMC6	0.233	proteasome 26S subunit, ATPase 6
**O43242**	PSMD3	0.156	proteasome 26S subunit, non-ATPase 3
**Q9H939-1**	PSTPIP2	0.223	proline-serine-threonine phosphatase interacting protein 2
**Q15185**	PTGES3	-0.228	prostaglandin E synthase 3
**Q14914**	PTGR1	0.271	prostaglandin reductase 1
**Q05397-5**	PTK2	-0.324	protein tyrosine kinase 2
**Q15678**	PTPN14	-0.225	protein tyrosine phosphatase non-receptor type 14
**P10586-1**	PTPRF	-0.398	protein tyrosine phosphatase receptor type F
**Q15262-3**	PTPRK	-0.711	protein tyrosine phosphatase receptor type K
**Q86Y79**	PTRH1	-0.185	peptidyl-tRNA hydrolase 1 homolog
**Q9Y3E5**	PTRH2	0.219	peptidyl-tRNA hydrolase 2
**Q15397**	PUM3	-0.455	pumilio RNA binding family member 3
**Q00577**	PURA	0.275	purine rich element binding protein A
**Q92626**	PXDN	-0.393	peroxidasin
**Q96PU8**	QKI	-0.203	QKI, KH domain containing RNA binding
**P20337**	RAB3B	-0.164	RAB3B, member RAS oncogene family
**Q9BUV8-5**	RAB5IF	0.351	RAB5 interacting factor
**P61006**	RAB8A	0.201	RAB8A, member RAS oncogene family
**P51151**	RAB9A	0.182	RAB9A, member RAS oncogene family
**P78406**	RAE1	0.194	ribonucleic acid export 1
**P62826**	RAN	0.15	RAN, member RAS oncogene family
**P52306-5**	RAP1GDS1	-0.297	Rap1 GTPase-GDP dissociation stimulator 1
**Q8TEU7-4**	RAPGEF6	-0.363	Rap guanine nucleotide exchange factor 6
**P20936-1**	RASA1	-0.176	RAS p21 protein activator 1
**Q14644**	RASA3	-0.316	RAS p21 protein activator 3
**Q9UJF2-2**	RASAL2	-0.466	RAS protein activator like 2
**Q7Z6E9**	RBBP6	0.286	RB binding protein 6, ubiquitin ligase
**Q9Y4C8**	RBM19	-0.327	RNA binding motif protein 19
**P52756**	RBM5	0.39	RNA binding motif protein 5
**Q9Y388**	RBMX2	0.736	RNA binding motif protein X-linked 2

**Q93062-3**	RBPMS	-0.298	RNA binding protein, mRNA processing factor
**P18754-2**	RCC1	0.156	regulator of chromosome condensation 1
**P46063**	RECQL	0.168	RecQ like helicase
**Q6NUM9-1**	RETSAT	0.253	retinol saturase
**P40938**	RFC3	0.204	replication factor C subunit 3
**P35249**	RFC4	0.222	replication factor C subunit 4
**P62745**	RHOB	0.26	ras homolog family member B
**Q8IXI1**	RHOT2	0.167	ras homolog family member T2
**O14730**	RIOK3	0.392	RIO kinase 3
**Q8NC42**	RNF149	-0.232	ring finger protein 149
**Q5VTR2**	RNF20	0.25	ring finger protein 20
**Q63HN8-4**	RNF213	-0.191	ring finger protein 213
**Q15287-1**	RNPS1	0.203	RNA binding protein with serine rich domain 1
**Q9H9Y2**	RPF1	-0.487	ribosome production factor 1 homolog
**Q9H7B2**	RPF2	-0.267	ribosome production factor 2 homolog
**Q6P5R6**	RPL22L1	-0.69	ribosomal protein L22 like 1
**P62829**	RPL23	-0.189	ribosomal protein L23
**Q6DKI1**	RPL7L1	-0.382	ribosomal protein L7 like 1
**Q5VT52**	RPRD2	0.179	regulation of nuclear pre-mRNA domain containing 2
**Q86WX3**	RPS19BP1	-0.475	ribosomal protein S19 binding protein 1
**P62857**	RPS28	-0.168	ribosomal protein S28
**P22090**	RPS4Y1	-0.325	ribosomal protein S4 Y-linked 1
**Q8N122**	RPTOR	-0.354	regulatory associated protein of MTOR complex 1
**P55042**	RRAD	1.297	RRAD, Ras related glycolysis inhibitor and calcium channel regulator
**P31350-2**	RRM2	0.61	ribonucleotide reductase regulatory subunit M2
**Q7LG56-6**	RRM2B	0.572	ribonucleotide reductase regulatory TP53 inducible subunit M2B
**Q5JTH9**	RRP12	-0.172	ribosomal RNA processing 12 homolog
**O76021**	RSL1D1	-0.167	ribosomal L1 domain containing 1
**Q7L4I2**	RSRC2	0.233	arginine and serine rich coiled-coil 2
**P0DJI8**	SAA1	-0.15	serum amyloid A1
**Q9NSI8-1**	SAMSN1	-0.328	SAM domain, SH3 domain and nuclear localization signals 1
**O00422**	SAP18	0.177	Sin3A associated protein 18
**Q9UHR5-1**	SAP30BP	0.169	SAP30 binding protein
**O43819**	SCO2	0.229	SCO cytochrome c oxidase assembly protein 2
**P22307**	SCP2	0.178	sterol carrier protein 2
**P31040**	SDHA	0.222	succinate dehydrogenase complex flavoprotein subunit A
**O60613**	SELENOF	-0.296	selenoprotein F
**O75326-1**	SEMA7A	-0.532	semaphorin 7A (John Milton Hagen blood group)
**P30740**	SERPINB1	0.257	serpin family B member 1
**P05121**	SERPINE1	-0.737	serpin family E member 1
**P36955**	SERPINF1	-0.65	serpin family F member 1
**Q01105**	SET	0.308	SET nuclear proto-oncogene
**Q9BYW2**	SETD2	-0.401	SET domain containing 2, histone lysine methyltransferase
**Q15459**	SF3A1	0.197	splicing factor 3a subunit 1
**Q15428**	SF3A2	0.25	splicing factor 3a subunit 2
**Q12874**	SF3A3	0.187	splicing factor 3a subunit 3
**Q13435**	SF3B2	0.163	splicing factor 3b subunit 2

**Q9H9B4**	SFXN1	0.168	sideroflexin 1
**Q7Z6J0**	SH3RF1	-0.662	SH3 domain containing ring finger 1
**Q8NEM2**	SHCBP1	0.332	SHC binding and spindle associated 1
**Q9H173**	SIL1	-0.568	SIL1 nucleotide exchange factor
**O43166-2**	SIPA1L1	-0.311	signal induced proliferation associated 1 like 1
**O60292**	SIPA1L3	-0.504	signal induced proliferation associated 1 like 3
**Q96EB6**	SIRT1	0.366	sirtuin 1
**Q9P270**	SLAIN2	0.233	SLAIN motif family member 2
**Q8WUM9**	SLC20A1	-0.331	solute carrier family 20 member 1
**P05141**	SLC25A5	0.227	solute carrier family 25 member 5
**P11169**	SLC2A3	0.188	solute carrier family 2 member 3
**Q9Y6M5**	SLC30A1	0.255	solute carrier family 30 member 1
**Q96QD8**	SLC38A2	-0.446	solute carrier family 38 member 2
**Q8WUX1-1**	SLC38A5	-0.206	solute carrier family 38 member 5
**Q8IWA5-2**	SLC44A2	0.301	solute carrier family 44 member 2
**P04920**	SLC4A2	-0.147	solute carrier family 4 member 2
**Q9Y6M7-7**	SLC4A7	-0.202	solute carrier family 4 member 7
**P30825**	SLC7A1	-0.165	solute carrier family 7 member 1
**P52569-3**	SLC7A2	-0.328	solute carrier family 7 member 2
**Q01650**	SLC7A5	0.163	solute carrier family 7 member 5
**Q08AF3-1**	SLFN5	0.285	schlafen family member 5
**O95391**	SLU7	0.275	SLU7 homolog, splicing factor
**P84022**	SMAD3	-0.271	SMAD family member 3
**Q92922**	SMARCC1	-0.294	SWI/SNF related, matrix associated, actin dependent regulator of chromatin subfamily
**Q96Q15-1**	SMG1	-0.17	SMG1 nonsense mediated mRNA decay associated PI3K related kinase
**Q8ND04-2**	SMG8	0.206	SMG8 nonsense mediated mRNA decay factor
**P53814-5**	SMTN	0.413	smoothelin
**Q9HAU4**	SMURF2	-0.341	SMAD specific E3 ubiquitin protein ligase 2
**Q9H7B4**	SMYD3	-0.534	SET and MYND domain containing 3
**Q7KZF4**	SND1	-0.257	staphylococcal nuclease and tudor domain containing 1
**P62308**	SNRPG	0.301	small nuclear ribonucleoprotein polypeptide G
**Q15036-1**	SNX17	-0.201	sorting nexin 17
**Q9UNH7-1**	SNX6	0.141	sorting nexin 6
**P04179**	SOD2	0.284	superoxide dismutase 2
**Q99523**	SORT1	-0.851	sortilin 1
**Q02447-1**	SP3	0.294	Sp3 transcription factor
**Q96R06**	SPAG5	0.183	sperm associated antigen 5
**P09486**	SPARC	-0.202	secreted protein acidic and cysteine rich
**Q8NB90-1**	SPATA5	-0.292	spermatogenesis associated 5
**Q86XZ4**	SPATS2	-0.279	spermatogenesis associated serine rich 2
**Q08AE8**	SPIRE1	-0.213	spire type actin nucleation factor 1
**P35270**	SPR	0.231	sepiapterin reductase
**Q01082-1**	SPTBN1	-0.168	spectrin beta, non-erythrocytic 1
**Q14534**	SQLE	-0.194	squalene epoxidase
**Q8NEF9**	SRFBP1	-0.182	serum response factor binding protein 1
**Q7Z6B7**	SRGAP1	-0.432	SLIT-ROBO Rho GTPase activating protein 1
**O75044**	SRGAP2	-0.25	SLIT-ROBO Rho GTPase activating protein 2

**P30626**	SRI	0.196	sorcin
**P49458**	SRP9	0.277	signal recognition particle 9
**Q9BXP5**	SRRT	0.239	serrate, RNA effector molecule
**Q05519**	SRSF11	0.185	serine and arginine rich splicing factor 11
**Q08170**	SRSF4	0.285	serine and arginine rich splicing factor 4
**Q13247**	SRSF6	0.209	serine and arginine rich splicing factor 6
**P05455**	SSB	0.2	small RNA binding exonuclease protection factor La
**Q04837**	SSBP1	0.277	single stranded DNA binding protein 1
**Q9UNL2-2**	SSR3	0.192	signal sequence receptor subunit 3
**Q8WVM7**	STAG1	-0.255	stromal antigen 1
**O75886**	STAM2	-0.212	signal transducing adaptor molecule 2
**O76061**	STC2	-0.764	stanniocalcin 2
**Q13586**	STIM1	-0.234	stromal interaction molecule 1
**Q13188-2**	STK3	-0.479	serine/threonine kinase 3
**P16949-1**	STMN1	0.295	stathmin 1
**P27105**	STOM	0.316	stomatin
**Q9UNK0**	STX8	-0.44	syntaxin 8
**O00186**	STXBP3	0.208	syntaxin binding protein 3
**Q5T5C0-1**	STXBP5	-0.238	syntaxin binding protein 5
**P53999**	SUB1	0.411	SUB1 regulator of transcription
**P53597**	SUCLG1	0.273	succinate-CoA ligase alpha subunit
**Q9Y5B9**	SUPT16H	0.221	SPT16 homolog, facilitates chromatin remodeling subunit
**Q7KZ85**	SUPT6H	0.155	SPT6 homolog, histone chaperone and transcription elongation factor
**O94864-1**	SUPT7L	0.273	SPT7 like, STAGA complex gamma subunit
**O75683**	SURF6	-0.258	surfeit 6
**Q8NF91**	SYNE1	-0.458	spectrin repeat containing nuclear envelope protein 1
**Q8WXH0-2**	SYNE2	-0.313	spectrin repeat containing nuclear envelope protein 2
**Q8N3V7-2**	SYNPO	0.252	synaptopodin
**Q9Y6A5**	TACC3	0.267	transforming acidic coiled-coil containing protein 3
**Q9BSH4**	TACO1	0.25	translational activator of cytochrome c oxidase I
**P09758**	TACSTD2	0.259	tumor associated calcium signal transducer 2
**Q86TJ2**	TADA2B	0.314	transcriptional adaptor 2B
**Q9C0D5**	TANC1	-0.315	tetratricopeptide repeat, ankyrin repeat and coiled-coil containing 1
**Q9HCD6-2**	TANC2	-0.465	tetratricopeptide repeat, ankyrin repeat and coiled-coil containing 2
**Q9H2K8**	TAOK3	-0.209	TAO kinase 3
**Q03518**	TAP1	0.249	transporter 1, ATP binding cassette subfamily B member
**Q03519-1**	TAP2	0.245	transporter 2, ATP binding cassette subfamily B member
**Q92609-2**	TBC1D5	-0.303	TBC1 domain family member 5
**O14776**	TCERG1	0.13	transcription elongation regulator 1
**P17987**	TCP1	0.128	t-complex 1
**Q00059**	TFAM	0.343	transcription factor A, mitochondrial
**Q92734**	TFG	0.206	trafficking from ER to golgi regulator
**P61812-2**	TGFB2	-0.695	transforming growth factor beta 2
**Q15582**	TGFBI	-0.692	transforming growth factor beta induced
**P37173-2**	TGFBR2	-0.762	transforming growth factor beta receptor 2
**Q6YHU6**	THADA	-0.321	THADA armadillo repeat containing
**P07996**	THBS1	-0.99	thrombospondin 1

**Q9NWX6**	THG1L	0.287	tRNA-histidine guanylyltransferase 1 like
**Q9NQ88**	TIGAR	0.27	TP53 induced glycolysis regulatory phosphatase
**O43615**	TIMM44	0.195	translocase of inner mitochondrial membrane 44
**P16035**	TIMP2	-0.35	TIMP metallopeptidase inhibitor 2
**P04183**	TK1	0.873	thymidine kinase 1
**O60603**	TLR2	-0.409	toll like receptor 2
**Q9UM00**	TMCO1	0.179	transmembrane and coiled-coil domains 1
**Q96MH6**	TMEM68	-0.984	transmembrane protein 68
**P42166**	TMPO	0.204	thymopoietin
**P24821**	TNC	-0.587	tenascin C
**Q03169**	TNFAIP2	0.376	TNF alpha induced protein 2
**O14763**	TNFRSF10B	0.205	TNF receptor superfamily member 10b
**Q9NP84**	TNFRSF12A	-0.436	TNF receptor superfamily member 12A
**Q15025**	TNIP1	-0.427	TNFAIP3 interacting protein 1
**Q9HBL0**	TNS1	-0.188	tensin 1
**Q68CZ2-1**	TNS3	-0.226	tensin 3
**Q8IZW8**	TNS4	-0.315	tensin 4
**P11388-4**	TOP2A	0.274	DNA topoisomerase II alpha
**P04637**	TP53	0.179	tumor protein p53
**Q53FA7**	TP53I3	0.361	tumor protein p53 inducible protein 3
**O43399-7**	TPD52L2	0.148	TPD52 like 2
**P60174**	TPI1	0.37	triosephosphate isomerase 1
**P07951**	TPM2	-0.433	tropomyosin 2
**P07951-2**	TPM2	-0.157	tropomyosin 2
**Q9ULW0**	TPX2	0.252	TPX2 microtubule nucleation factor
**O43715**	TRIAP1	0.29	TP53 regulated inhibitor of apoptosis 1
**P19474**	TRIM21	0.372	tripartite motif containing 21
**Q8IYM9-1**	TRIM22	0.515	tripartite motif containing 22
**Q13263**	TRIM28	0.294	tripartite motif containing 28
**Q13049**	TRIM32	0.208	tripartite motif containing 32
**O75962-1**	TRIO	-0.308	trio Rho guanine nucleotide exchange factor
**Q15650**	TRIP4	-0.421	thyroid hormone receptor interactor 4
**Q7L0Y3**	TRMT10C	0.215	tRNA methyltransferase 10C, mitochondrial RNase P subunit
**Q96Q11**	TRNT1	0.178	tRNA nucleotidyl transferase 1
**Q15631-1**	TSN	0.239	translin
**Q16762**	TST	0.258	thiosulfate sulfurtransferase
**Q6P3X3**	TTC27	-0.268	tetratricopeptide repeat domain 27
**Q9ULT0-4**	TTC7A	-0.199	tetratricopeptide repeat domain 7A
**P68366**	TUBA4A	0.202	tubulin alpha 4a
**Q13509**	TUBB3	0.324	tubulin beta 3 class III
**P49411**	TUFM	0.151	Tu translation elongation factor, mitochondrial
**P40222**	TXLNA	0.194	taxilin alpha
**O14530**	TXNDC9	0.217	thioredoxin domain containing 9
**P83876**	TXNL4A	-0.182	thioredoxin like 4A
**Q9NV66**	TYW1	-0.436	tRNA-yW synthesizing protein 1 homolog
**Q16763**	UBE2S	0.299	ubiquitin conjugating enzyme E2 S
**Q9NPD8**	UBE2T	0.235	ubiquitin conjugating enzyme E2 T

**O95155-4**	UBE4B	-0.185	ubiquitination factor E4B
**O94874**	UFL1	0.155	UFM1 specific ligase 1
**Q16739**	UGCG	-0.32	UDP-glucose ceramide glucosyltransferase
**Q96T88-2**	UHRF1	-0.319	ubiquitin like with PHD and ring finger domains 1
**Q96RL1**	UIMC1	-0.297	ubiquitin interaction motif containing 1
**Q9UDW1**	UQCR10	0.345	ubiquinol-cytochrome c reductase, complex III subunit X
**O94763**	URI1	-0.218	URI1 prefoldin like chaperone
**P51784**	USP11	0.168	ubiquitin specific peptidase 11
**Q8NFA0**	USP32	-0.255	ubiquitin specific peptidase 32
**Q9P275-2**	USP36	-0.197	ubiquitin specific peptidase 36
**Q93008**	USP9X	-0.206	ubiquitin specific peptidase 9 X-linked
**Q9BVJ6-1**	UTP14A	-0.433	UTP14A small subunit processome component
**Q9Y5J1**	UTP18	-0.162	UTP18 small subunit processome component
**O75691**	UTP20	-0.307	UTP20 small subunit processome component
**Q9NQZ2**	UTP3	0.215	UTP3 small subunit processome component
**Q9NYH9**	UTP6	-0.214	UTP6 small subunit processome component
**P46939**	UTRN	-0.376	utrophin
**P52735**	VAV2	-0.337	vav guanine nucleotide exchange factor 2
**P19320**	VCAM1	-0.665	vascular cell adhesion molecule 1
**P18206**	VCL	-0.211	vinculin
**P45880-1**	VDAC2	0.198	voltage dependent anion channel 2
**Q9Y277**	VDAC3	0.297	voltage dependent anion channel 3
**Q709C8**	VPS13C	-0.159	vacuolar protein sorting 13 homolog C
**O14972**	VPS26C	-0.157	VPS26 endosomal protein sorting factor C
**Q8NEZ2**	VPS37A	0.297	VPS37A subunit of ESCRT-I
**P49754**	VPS41	-0.238	VPS41 subunit of HOPS complex
**Q96AJ9**	VTI1A	-0.433	vesicle transport through interaction with t-SNAREs 1A
**A3KMH1**	VWA8	-0.146	von Willebrand factor A domain containing 8
**Q9Y2W2**	WBP11	0.29	WW domain binding protein 11
**Q969T9**	WBP2	-0.276	WW domain binding protein 2
**O15213**	WDR46	-0.212	WD repeat domain 46
**Q9NW82**	WDR70	-0.289	WD repeat domain 70
**Q6RFH5**	WDR74	-0.169	WD repeat domain 74
**Q6UXN9**	WDR82	0.196	WD repeat domain 82
**Q5T9L3-2**	WLS	-0.344	Wnt ligand secretion mediator
**Q96S55**	WRNIP1	0.153	WRN helicase interacting protein 1
**O00308**	WWP2	-0.551	WW domain containing E3 ubiquitin protein ligase 2
**Q01831**	XPC	0.515	XPC complex subunit, DNA damage recognition and repair factor
**Q9C0E2**	XPO4	-0.179	exportin 4
**Q96QU8**	XPO6	-0.236	exportin 6
**Q9Y5A9**	YTHDF2	-0.242	YTH N6-methyladenosine RNA binding protein 2
**O96006**	ZBED1	0.323	zinc finger BED-type containing 1
**O75152**	ZC3H11A	0.204	zinc finger CCCH-type containing 11A
**Q8IWR0**	ZC3H7A	-0.132	zinc finger CCCH-type containing 7A
**Q8TF68-2**	ZNF384	0.696	zinc finger protein 384
**O43149**	ZZEF1	-0.257	zinc finger ZZ-type and EF-hand domain containing 1
**P00374**		0.229	

**P84243**		0.167	

List of proteins deregulated in THLE2 cells after 24h exposure to AgNP 3.5 ug/mL + CDDP 10 mM

ID	Symbol	Expr Log Ratio	Entrez Gene Name
**Q86V21-1**	AACS	-0.213	acetoacetyl-CoA synthetase
**Q6PD74**	AAGAB	-0.379	alpha and gamma adaptin binding protein
**Q2M2I8**	AAK1	-0.204	AP2 associated kinase 1
**Q9Y312**	AAR2	0.229	AAR2 splicing factor
**Q5JTZ9**	AARS2	0.178	alanyl-tRNA synthetase 2, mitochondrial
**Q9UDR5**	AASS	0.186	aminoadipate-semialdehyde synthase
**P33897**	ABCD1	0.39	ATP binding cassette subfamily D member 1
**Q9NUJ1**	ABHD10	0.254	abhydrolase domain containing 10
**Q8NFV4**	ABHD11	0.206	abhydrolase domain containing 11
**O95870**	ABHD16A	0.367	abhydrolase domain containing 16A
**Q12979**	ABR	-0.263	ABR activator of RhoGEF and GTPase
**Q9P1F3**	ABRACL	-0.498	ABRA C-terminal like
**P09110-1**	ACAA1	0.181	acetyl-CoA acyltransferase 1
**P42765**	ACAA2	0.277	acetyl-CoA acyltransferase 2
**Q13085-4**	ACACA	-0.31	acetyl-CoA carboxylase alpha
**Q9H845**	ACAD9	0.201	acyl-CoA dehydrogenase family member 9
**P11310-2**	ACADM	0.231	acyl-CoA dehydrogenase medium chain
**P24752**	ACAT1	0.187	acetyl-CoA acetyltransferase 1
**Q5T8D3-3**	ACBD5	0.206	acyl-CoA binding domain containing 5
**Q9BR61**	ACBD6	-0.337	acyl-CoA binding domain containing 6
**E7EQT4**	ACIN1	0.605	apoptotic chromatin condensation inducer 1
**Q9UKV3-5**	ACIN1	0.205	apoptotic chromatin condensation inducer 1
**P21399**	ACO1	-0.239	aconitase 1
**Q86TX2**	ACOT1	0.364	acyl-CoA thioesterase 1
**P11117**	ACP2	0.187	acid phosphatase 2, lysosomal
**Q4G176**	ACSF3	0.254	acyl-CoA synthetase family member 3
**P33121**	ACSL1	0.21	acyl-CoA synthetase long chain family member 1
**P12814-3**	ACTN1	-0.415	actinin alpha 1
**O43707**	ACTN4	-0.363	actinin alpha 4
**P00813**	ADA	0.289	adenosine deaminase
**P35611-1**	ADD1	-0.262	adducin 1
**Q9UEY8-2**	ADD3	-0.212	adducin 3
**P55263**	ADK	-0.164	adenosine kinase
**Q9H2P0**	ADNP	0.215	activity dependent neuroprotector homeobox
**Q9BRR6-1**	ADPGK	0.17	ADP dependent glucokinase
**Q8N556-2**	AFAP1	-0.213	actin filament associated protein 1
**Q9UHB7-1**	AFF4	0.235	AF4/FMR2 family member 4
**Q9Y4W6**	AFG3L2	0.169	AFG3 like matrix AAA peptidase subunit 2
**Q9NUQ2**	AGPAT5	0.23	1-acylglycerol-3-phosphate O-acyltransferase 5
**O00468**	AGRN	-0.267	agrin

**O95831-1**	AIFM1	0.148	apoptosis inducing factor mitochondria associated 1
**Q9BRQ8**	AIFM2	0.374	apoptosis inducing factor mitochondria associated 2
**P54819**	AK2	0.263	adenylate kinase 2
**Q9UIJ7-1**	AK3	0.3	adenylate kinase 3
**P27144**	AK4	0.218	adenylate kinase 4
**Q9UKA4**	AKAP11	-0.348	A-kinase anchoring protein 11
**Q02952-1**	AKAP12	-0.323	A-kinase anchoring protein 12
**Q9ULX6**	AKAP8L	0.267	A-kinase anchoring protein 8 like
**Q13740**	ALCAM	-0.197	activated leukocyte cell adhesion molecule
**P30837**	ALDH1B1	0.195	aldehyde dehydrogenase 1 family member B1
**P51648-2**	ALDH3A2	0.197	aldehyde dehydrogenase 3 family member A2
**Q9UJX6**	ANAPC2	-0.195	anaphase promoting complex subunit 2
**Q9BY76**	ANGPTL4	-0.75	angiopoietin like 4
**Q9P2R3-4**	ANKFY1	-0.205	ankyrin repeat and FYVE domain containing 1
**Q8IWZ3-6**	ANKHD1/ANKHD1-EIF4EBP3	-0.181	ankyrin repeat and KH domain containing 1
**Q9NQW6**	ANLN	0.31	anillin actin binding protein
**P39687**	ANP32A	-0.168	acidic nuclear phosphoprotein 32 family member A
**Q92688**	ANP32B	-0.388	acidic nuclear phosphoprotein 32 family member B
**P58335-1**	ANTXR2	-0.57	ANTXR cell adhesion molecule 2
**P07355-2**	ANXA2	-0.387	annexin A2
**P12429**	ANXA3	-0.212	annexin A3
**P09525**	ANXA4	-0.274	annexin A4
**P08133**	ANXA6	-0.205	annexin A6
**P20073**	ANXA7	-0.309	annexin A7
**P53680**	AP2S1	0.247	adaptor related protein complex 2 subunit sigma 1
**O14617-5**	AP3D1	-0.148	adaptor related protein complex 3 subunit delta 1
**Q92572**	AP3S1	-0.179	adaptor related protein complex 3 subunit sigma 1
**Q7Z5R6**	APBB1IP	-0.319	amyloid beta precursor protein binding family B member 1 interacting protein
**Q06481**	APLP2	-0.207	amyloid beta precursor like protein 2
**P04114**	APOB	-0.458	apolipoprotein B
**Q9NRW3**	APOBEC3C	0.352	apolipoprotein B mRNA editing enzyme catalytic subunit 3C
**P05067**	APP	-0.61	amyloid beta precursor protein
**O60306**	AQR	0.141	aquarius intron-binding spliceosomal factor
**Q96P48-6**	ARAP1	-0.2	ArfGAP with RhoGAP domain, ankyrin repeat and PH domain 1
**P15514**	AREG	-0.127	amphiregulin
**Q9NP61**	ARFGAP3	-0.213	ADP ribosylation factor GTPase activating protein 3
**Q9Y6D6**	ARFGEF1	-0.215	ADP ribosylation factor guanine nucleotide exchange factor 1
**Q8N392**	ARHGAP18	-0.274	Rho GTPase activating protein 18
**Q13017-1**	ARHGAP5	-0.142	Rho GTPase activating protein 5
**O15013**	ARHGEF10	-0.261	Rho guanine nucleotide exchange factor 10
**Q9NZN5**	ARHGEF12	-0.119	Rho guanine nucleotide exchange factor 12
**Q8NFD5-3**	ARID1B	-0.367	AT-rich interaction domain 1B
**Q9NVJ2**	ARL8B	0.192	ADP ribosylation factor like GTPase 8B
**Q7Z3E5**	ARMC9	-0.641	armadillo repeat containing 9
**P15289**	ARSA	0.204	arylsulfatase A
**Q9HBK9**	AS3MT	-0.588	arsenite methyltransferase
**Q13510-2**	ASAH1	0.179	N-acylsphingosine amidohydrolase 1

**O43150-1**	ASAP2	-0.266	ArfGAP with SH3 domain, ankyrin repeat and PH domain 2
**Q8N9N2**	ASCC1	-0.567	activating signal cointegrator 1 complex subunit 1
**Q9H1I8-1**	ASCC2	-0.509	activating signal cointegrator 1 complex subunit 2
**Q8N3C0**	ASCC3	-0.534	activating signal cointegrator 1 complex subunit 3
**Q9NVP2**	ASF1B	0.221	anti-silencing function 1B histone chaperone
**Q8NBU5**	ATAD1	0.171	ATPase family AAA domain containing 1
**Q9NVI7-2**	ATAD3A	0.222	ATPase family AAA domain containing 3A
**O95352**	ATG7	-0.409	autophagy related 7
**Q6DD88**	ATL3	0.15	atlastin GTPase 3
**P54259**	ATN1	0.371	atrophin 1
**O00244**	ATOX1	0.201	antioxidant 1 copper chaperone
**P05026**	ATP1B1	0.213	ATPase Na+/K+ transporting subunit beta 1
**P23634-1**	ATP2B4	-0.198	ATPase plasma membrane Ca2+ transporting 4
**P06576**	ATP5F1B	0.202	ATP synthase F1 subunit beta
**P56381**	ATP5F1E	0.443	ATP synthase F1 subunit epsilon
**Q9UII2**	ATP5IF1	0.581	ATP synthase inhibitory factor subunit 1
**O75947-1**	ATP5PD	0.218	ATP synthase peripheral stalk subunit d
**P48047**	ATP5PO	0.212	ATP synthase peripheral stalk subunit OSCP
**Q9UBB4**	ATXN10	-0.259	ataxin 10
**P0C7T5**	ATXN1L	0.403	ataxin 1 like
**Q9Y679-1**	AUP1	0.234	AUP1 lipid droplet regulating VLDL assembly factor
**Q96GD4-5**	AURKB	0.291	aurora kinase B
**Q9NQS1**	AVEN	-0.285	apoptosis and caspase activation inhibitor
**P30530**	AXL	-0.374	AXL receptor tyrosine kinase
**P61769**	B2M	0.214	beta-2-microglobulin
**Q9NXR7-2**	BABAM2	-0.261	BRISC and BRCA1 A complex member 2
**O95816**	BAG2	0.145	BCL2 associated athanogene 2
**O95429**	BAG4	-0.246	BCL2 associated athanogene 4
**Q9UHR4**	BAIAP2L1	-0.17	BAR/IMD domain containing adaptor protein 2 like 1
**O75531**	BANF1	0.239	barrier to autointegration factor 1
**Q8WY36**	BBX	-0.34	BBX high mobility group box domain containing
**P51572-2**	BCAP31	0.248	B cell receptor associated protein 31
**O75815-1**	BCAR3	-0.681	BCAR3 adaptor protein, NSP family member
**O75934**	BCAS2	0.2	BCAS2 pre-mRNA processing factor
**O15382**	BCAT2	0.176	branched chain amino acid transaminase 2
**Q9NYF8-1**	BCLAF1	0.397	BCL2 associated transcription factor 1
**P11274-1**	BCR	-0.172	BCR activator of RhoGEF and GTPase
**Q9H694**	BICC1	-0.476	BicC family RNA binding protein 1
**O15392**	BIRC5	0.256	baculoviral IAP repeat containing 5
**Q9NR09**	BIRC6	-0.358	baculoviral IAP repeat containing 6
**Q14692**	BMS1	-0.165	BMS1 ribosome biogenesis factor
**Q8NFC6**	BOD1L1	0.162	biorientation of chromosomes in cell division 1 like 1
**Q12830**	BPTF	-0.195	bromodomain PHD finger transcription factor
**P20290**	BTF3	-0.191	basic transcription factor 3
**Q7KYR7**	BTN2A1	0.436	butyrophilin subfamily 2 member A1
**Q9H0W9**	C11orf54	-0.259	chromosome 11 open reading frame 54
**Q9H3H3-3**	C11orf68	-0.411	chromosome 11 open reading frame 68

**Q8N5I9**	C12orf45	0.509	chromosome 12 open reading frame 45
**Q99622**	C12orf57	-0.291	chromosome 12 open reading frame 57
**Q9UFG5**	C19orf25	0.297	chromosome 19 open reading frame 25
**Q9BXJ4-3**	C1QTNF3	-0.627	C1q and TNF related 3
**Q9GZN8-2**	C20orf27	-0.301	chromosome 20 open reading frame 27
**Q9BVC5**	C2orf49	0.256	chromosome 2 open reading frame 49
**P01024**	C3	-0.308	complement C3
**P0C0L4-1**	C4A/C4B	-0.347	complement C4A (Rodgers blood group)
**Q49AR2**	C5orf22	0.404	chromosome 5 open reading frame 22
**Q7Z6I8**	C5orf24	0.346	chromosome 5 open reading frame 24
**Q9BRJ6**	C7orf50	-0.444	chromosome 7 open reading frame 50
**Q5T6V5**	C9orf64	-0.243	chromosome 9 open reading frame 64
**Q9Y376**	CAB39	-0.506	calcium binding protein 39
**P27708**	CAD	-0.199	carbamoyl-phosphate synthetase 2, aspartate transcarbamylase, and dihydroorotase
**P27824-2**	CANX	0.131	calnexin
**P40123**	CAP2	-0.213	cyclase associated actin cytoskeleton regulatory protein 2
**Q14444**	CAPRIN1	-0.227	cell cycle associated protein 1
**Q9Y2V2**	CARHSP1	-0.172	calcium regulated heat stable protein 1
**Q5VZK9**	CARMIL1	-0.389	capping protein regulator and myosin 1 linker 1
**P49589-3**	CARS1	-0.136	cysteinyl-tRNA synthetase 1
**O14936**	CASK	-0.185	calcium/calmodulin dependent serine protein kinase
**P49662**	CASP4	0.238	caspase 4
**P04040**	CAT	0.259	catalase
**Q6NZI2**	CAVIN1	0.178	caveolae associated protein 1
**O95810**	CAVIN2	0.292	caveolae associated protein 2
**P45973**	CBX5	0.347	chromobox 5
**Q9HC52**	CBX8	0.371	chromobox 8
**Q6P1N0**	CC2D1A	-0.245	coiled-coil and C2 domain containing 1A
**Q8N163-1**	CCAR2	0.178	cell cycle and apoptosis regulator 2
**Q96BQ5**	CCDC127	0.395	coiled-coil domain containing 127
**Q6PK04**	CCDC137	-0.32	coiled-coil domain containing 137
**Q7Z3E2**	CCDC186	0.266	coiled-coil domain containing 186
**Q86WR0**	CCDC25	-0.443	coiled-coil domain containing 25
**Q4VC31**	CCDC58	0.309	coiled-coil domain containing 58
**Q9Y6R9**	CCDC61	-1.012	coiled-coil domain containing 61
**Q76M96-2**	CCDC80	-0.922	coiled-coil domain containing 80
**A6NKD9**	CCDC85C	-0.343	coiled-coil domain containing 85C
**O00622**	CCN1	-0.999	cellular communication network factor 1
**P29279**	CCN2	-0.588	cellular communication network factor 2
**P20248**	CCNA2	0.411	cyclin A2
**P24385**	CCND1	-0.971	cyclin D1
**O60563**	CCNT1	0.488	cyclin T1
**Q9ULG6-5**	CCPG1	0.193	cell cycle progression 1
**O14618**	CCS	0.243	copper chaperone for superoxide dismutase
**Q6YHK3**	CD109	-0.278	CD109 molecule
**P08571**	CD14	-0.248	CD14 molecule
**Q9Y5K6**	CD2AP	-0.243	CD2 associated protein

**O95400**	CD2BP2	0.29	CD2 cytoplasmic tail binding protein 2
**P16070**	CD44	0.206	CD44 molecule (Indian blood group)
**P08174-7**	CD55	0.408	CD55 molecule (Cromer blood group)
**P13987**	CD59	0.334	CD59 molecule (CD59 blood group)
**P21926**	CD9	0.18	CD9 molecule
**Q12834**	CDC20	-0.241	cell division cycle 20
**Q9UJX2-1**	CDC23	0.288	cell division cycle 23
**Q9H3Q1**	CDC42EP4	0.254	CDC42 effector protein 4
**O75419-3**	CDC45	0.394	cell division cycle 45
**Q99459**	CDC5L	0.204	cell division cycle 5 like
**O00311**	CDC7	0.268	cell division cycle 7
**Q69YH5-1**	CDCA2	0.21	cell division cycle associated 2
**Q96FF9**	CDCA5	0.925	cell division cycle associated 5
**Q9H5V8**	CDCP1	-0.131	CUB domain containing protein 1
**P19022**	CDH2	-0.203	cadherin 2
**P06493**	CDK1	0.298	cyclin dependent kinase 1
**P24941**	CDK2	0.188	cyclin dependent kinase 2
**Q96SN8**	CDK5RAP2	-0.378	CDK5 regulatory subunit associated protein 2
**Q00534**	CDK6	-0.226	cyclin dependent kinase 6
**Q5VV42**	CDKAL1	-0.652	CDK5 regulatory subunit associated protein 1 like 1
**P42771-1**	CDKN2A	-0.18	cyclin dependent kinase inhibitor 2A
**Q8N726**	CDKN2A	-0.303	cyclin dependent kinase inhibitor 2A
**Q86X02**	CDR2L	0.348	cerebellar degeneration related protein 2 like
**Q03701**	CEBPZ	0.184	CCAAT enhancer binding protein zeta
**Q02224**	CENPE	-0.264	centromere protein E
**Q9H3R5**	CENPH	0.328	centromere protein H
**Q7Z7K6-3**	CENPV	0.321	centromere protein V
**Q8TEP8-3**	CEP192	-0.394	centrosomal protein 192
**Q9Y5P4-3**	CERT1	-0.312	ceramide transporter 1
**O00748**	CES2	0.448	carboxylesterase 2
**P41208**	CETN2	0.21	centrin 2
**O15182**	CETN3	-0.384	centrin 3
**Q8N884**	CGAS	0.368	cyclic GMP-AMP synthase
**Q13112**	CHAF1B	0.411	chromatin assembly factor 1 subunit B
**Q96JM3**	CHAMP1	0.31	chromosome alignment maintaining phosphoprotein 1
**Q8N4Q1-2**	CHCHD4	0.374	coiled-coil-helix-coiled-coil-helix domain containing 4
**Q9HCK8-1**	CHD8	0.154	chromodomain helicase DNA binding protein 8
**Q7LBR1**	CHMP1B	0.19	charged multivesicular body protein 1B
**Q9P2E5-1**	CHPF2	0.134	chondroitin polymerizing factor 2
**P0CG13-1**	CHTF8	0.288	chromosome transmission fidelity factor 8
**Q9NZ45**	CISD1	0.342	CDGSH iron sulfur domain 1
**Q8N5K1**	CISD2	0.276	CDGSH iron sulfur domain 2
**O14578-4**	CIT	-0.175	citron rho-interacting serine/threonine kinase
**Q8WWK9-5**	CKAP2	0.656	cytoskeleton associated protein 2
**Q07065**	CKAP4	0.253	cytoskeleton associated protein 4
**P61024**	CKS1B	0.314	CDC28 protein kinase regulatory subunit 1B
**Q7Z460-4**	CLASP1	-0.238	cytoplasmic linker associated protein 1

**O75122-3**	CLASP2	-0.145	cytoplasmic linker associated protein 2
**O14967**	CLGN	0.289	calmegin
**Q9Y696**	CLIC4	-0.276	chloride intracellular channel 4
**P49759-3**	CLK1	0.333	CDC like kinase 1
**P49761**	CLK3	0.285	CDC like kinase 3
**Q9H6B4**	CLMP	-0.416	CXADR like membrane protein
**O75503**	CLN5	0.3	CLN5 intracellular trafficking protein
**Q92989**	CLP1	0.385	cleavage and polyadenylation factor I subunit 1
**O76031**	CLPX	0.191	caseinolytic mitochondrial matrix peptidase chaperone subunit
**O94985-1**	CLSTN1	-0.371	calsyntenin 1
**O75153**	CLUH	-0.239	clustered mitochondria homolog
**Q8NFW8**	CMAS	0.475	cytidine monophosphate N-acetylneuraminic acid synthetase
**Q96DG6**	CMBL	0.376	carboxymethylenebutenolidase homolog
**Q9BQ75**	CMSS1	-0.293	cms1 ribosomal small subunit homolog
**Q8N1G2**	CMTR1	0.248	cap methyltransferase 1
**P62633**	CNBP	0.533	CCHC-type zinc finger nucleic acid binding protein
**Q99439**	CNN2	-0.221	calponin 2
**P09543-1**	CNP	0.416	2',3'-cyclic nucleotide 3' phosphodiesterase
**Q9Y2B0**	CNPY2	0.223	canopy FGF signaling regulator 2
**Q9Y2R0**	COA3	0.189	cytochrome c oxidase assembly factor 3
**Q96BR5**	COA7	0.177	cytochrome c oxidase assembly factor 7 (putative)
**Q9UP83-2**	COG5	-0.271	component of oligomeric golgi complex 5
**P38432**	COIL	0.404	coilin
**P02452**	COL1A1	-0.584	collagen type I alpha 1 chain
**P08123**	COL1A2	-0.666	collagen type I alpha 2 chain
**P02462**	COL4A1	-0.239	collagen type IV alpha 1 chain
**P08572**	COL4A2	-0.429	collagen type IV alpha 2 chain
**Q86VU5**	COMTD1	0.176	catechol-O-methyltransferase domain containing 1
**O14579-1**	COPE	-0.328	coatomer protein complex subunit epsilon
**Q9UBF2**	COPG2	-0.161	coatomer protein complex subunit gamma 2
**Q9Y2Z9**	COQ6	0.216	coenzyme Q6, monooxygenase
**Q8NI60-1**	COQ8A	0.474	coenzyme Q8A
**O75208**	COQ9	0.201	coenzyme Q9
**Q14019**	COTL1	-0.469	coactosin like F-actin binding protein 1
**P13073**	COX4I1	0.267	cytochrome c oxidase subunit 4I1
**O14548**	COX7A2L	-0.397	cytochrome c oxidase subunit 7A2 like
**P15954**	COX7C	0.2	cytochrome c oxidase subunit 7C
**P36551**	CPOX	0.223	coproporphyrinogen oxidase
**Q9BRF8-1**	CPPED1	-0.441	calcineurin like phosphoesterase domain containing 1
**P31327-3**	CPS1	-0.251	carbamoyl-phosphate synthase 1
**Q9P2I0**	CPSF2	0.161	cleavage and polyadenylation specific factor 2
**Q8N684-3**	CPSF7	0.174	cleavage and polyadenylation specific factor 7
**P23786**	CPT2	0.358	carnitine palmitoyltransferase 2
**P43155**	CRAT	0.233	carnitine O-acetyltransferase
**O75629**	CREG1	0.326	cellular repressor of E1A stimulated genes 1
**Q9NZV1**	CRIM1	-0.459	cysteine rich transmembrane BMP regulator 1
**P52943**	CRIP2	0.272	cysteine rich protein 2

**Q9BZJ0**	CRNKL1	0.214	crooked neck pre-mRNA splicing factor 1
**Q9UKG9-3**	CROT	0.204	carnitine O-octanoyltransferase
**Q68DQ2**	CRYBG3	-0.28	crystallin beta-gamma domain containing 3
**O75390**	CS	0.139	citrate synthase
**O75534-4**	CSDE1	-0.328	cold shock domain containing E1
**P68400**	CSNK2A1	0.147	casein kinase 2 alpha 1
**P19784**	CSNK2A2	0.177	casein kinase 2 alpha 2
**Q16527**	CSRP2	0.251	cysteine and glycine rich protein 2
**Q05048**	CSTF1	0.183	cleavage stimulation factor subunit 1
**Q9Y5B0**	CTDP1	-0.194	CTD phosphatase subunit 1
**O43310-2**	CTIF	-0.496	cap binding complex dependent translation initiation factor
**P35221**	CTNNA1	-0.208	catenin alpha 1
**Q6PD62**	CTR9	0.175	CTR9 homolog, Paf1/RNA polymerase II complex component
**P10619**	CTSA	0.224	cathepsin A
**P53634**	CTSC	0.137	cathepsin C
**P07339**	CTSD	0.28	cathepsin D
**P07711**	CTSL	0.194	cathepsin L
**P42830**	CXCL5	-0.695	C-X-C motif chemokine ligand 5
**Q9UHQ9**	CYB5R1	0.293	cytochrome b5 reductase 1
**P99999**	CYCS	0.262	cytochrome c, somatic
**Q96EP5**	DAZAP1	-0.403	DAZ associated protein 1
**P11182**	DBT	0.199	dihydrolipoamide branched chain transacylase E2
**Q8TEB1**	DCAF11	0.264	DDB1 and CUL4 associated factor 11
**Q9NV06**	DCAF13	-0.249	DDB1 and CUL4 associated factor 13
**Q96PD2-2**	DCBLD2	-0.297	discoidin, CUB and LCCL domain containing 2
**Q9H773**	DCTPP1	-0.235	dCTP pyrophosphatase 1
**Q7Z4W1**	DCXR	0.249	dicarbonyl and L-xylulose reductase
**Q92466**	DDB2	0.49	damage specific DNA binding protein 2
**Q96HY6**	DDRGK1	0.165	DDRGK domain containing 1
**P30046**	DDT	-0.393	D-dopachrome tautomerase
**Q92841**	DDX17	-0.134	DEAD-box helicase 17
**Q9NVP1**	DDX18	0.149	DEAD-box helicase 18
**Q9H8H2**	DDX31	-0.208	DEAD-box helicase 31
**Q9UJV9**	DDX41	0.198	DEAD-box helicase 41
**Q9Y6V7**	DDX49	-0.243	DEAD-box helicase 49
**Q9Y2R4**	DDX52	-0.182	DExD-box helicase 52
**Q9NY93**	DDX56	-0.414	DEAD-box helicase 56
**Q16698**	DECR1	0.201	2,4-dienoyl-CoA reductase 1
**Q9NUI1**	DECR2	0.192	2,4-dienoyl-CoA reductase 2
**Q8WYQ5-1**	DGCR8	0.236	DGCR8 microprocessor complex subunit
**Q6IAN0**	DHRS7B	0.22	dehydrogenase/reductase 7B
**Q92620**	DHX38	0.139	DEAH-box helicase 38
**Q6P158-1**	DHX57	-0.245	DExH-box helicase 57
**O60879**	DIAPH2	-0.38	diaphanous related formin 2
**Q9NSV4**	DIAPH3	-0.448	diaphanous related formin 3
**Q9BTC0**	DIDO1	0.245	death inducer-obliterator 1
**Q9P265**	DIP2B	-0.315	disco interacting protein 2 homolog B

**Q8IYB7**	DIS3L2	-0.248	DIS3 like 3'-5' exoribonuclease 2
**O60832**	DKC1	0.168	dyskerin pseudouridine synthase 1
**O94907**	DKK1	-0.299	dickkopf WNT signaling pathway inhibitor 1
**P10515**	DLAT	0.19	dihydrolipoamide S-acetyltransferase
**P09622**	DLD	0.319	dihydrolipoamide dehydrogenase
**Q9Y2H0**	DLGAP4	-0.451	DLG associated protein 4
**P36957**	DLST	0.285	dihydrolipoamide S-succinyltransferase
**Q9NPF5**	DMAP1	0.18	DNA methyltransferase 1 associated protein 1
**Q86Y56**	DNAAF5	-0.214	dynein axonemal assembly factor 5
**P25685**	DNAJB1	0.28	DnaJ heat shock protein family (Hsp40) member B1
**O75190**	DNAJB6	-0.283	DnaJ heat shock protein family (Hsp40) member B6
**Q96KC8**	DNAJC1	-0.113	DnaJ heat shock protein family (Hsp40) member C1
**Q96DA6**	DNAJC19	0.384	DnaJ heat shock protein family (Hsp40) member C19
**Q05193**	DNM1	-0.207	dynamin 1
**Q6XZF7**	DNMBP	-0.236	dynamin binding protein
**P26358-1**	DNMT1	-0.194	DNA methyltransferase 1
**Q96N67**	DOCK7	-0.149	dedicator of cytokinesis 7
**Q9BZ29-4**	DOCK9	-0.228	dedicator of cytokinesis 9
**Q9NYP3**	DONSON	0.302	downstream neighbor of SON
**Q86TI2-2**	DPP9	-0.143	dipeptidyl peptidase 9
**Q14919-2**	DRAP1	0.166	DR1 associated protein 1
**Q14126**	DSG2	0.122	desmoglein 2
**P15924-1**	DSP	-0.176	desmoplakin
**Q03001-7**	DST	-0.381	dystonin
**P60981-1**	DSTN	-0.192	destrin, actin depolymerizing factor
**Q8TDB6**	DTX3L	0.178	deltex E3 ubiquitin ligase 3L
**Q96G46**	DUS3L	0.224	dihydrouridine synthase 3 like
**Q9BVJ7**	DUSP23	-0.173	dual specificity phosphatase 23
**Q92997**	DVL3	0.278	dishevelled segment polarity protein 3
**Q14204**	DYNC1H1	-0.159	dynein cytoplasmic 1 heavy chain 1
**Q13409-2**	DYNC1I2	-0.185	dynein cytoplasmic 1 intermediate chain 2
**P63172**	DYNLT1	-0.549	dynein light chain Tctex-type 1
**O75923-13**	DYSF	-0.237	dysferlin
**Q13011**	ECH1	0.304	enoyl-CoA hydratase 1
**O75521**	ECI2	0.321	enoyl-CoA delta isomerase 2
**Q9BQ95**	ECSIT	0.209	ECSIT signalling integrator
**Q9H8V3-3**	ECT2	0.198	epithelial cell transforming 2
**Q3B7T1**	EDRF1	-0.547	erythroid differentiation regulatory factor 1
**Q05639**	EEF1A2	-0.316	eukaryotic translation elongation factor 1 alpha 2
**P29692-2**	EEF1D	0.326	eukaryotic translation elongation factor 1 delta
**O00418**	EEF2K	-0.432	eukaryotic elongation factor 2 kinase
**P57772**	EEFSEC	-0.2	eukaryotic elongation factor, selenocysteine-tRNA specific
**Q12805**	EFEMP1	-0.384	EGF containing fibulin extracellular matrix protein 1
**Q7Z2Z2**	EFL1	-0.159	elongation factor like GTPase 1
**P98172**	EFNB1	0.287	ephrin B1
**P52799**	EFNB2	-0.301	ephrin B2
**P00533-1**	EGFR	-0.411	epidermal growth factor receptor

**Q8N3D4**	EHBP1L1	-0.218	EH domain binding protein 1 like 1
**Q9H223**	EHD4	-0.132	EH domain containing 4
**Q9H9B1**	EHMT1	-0.217	euchromatic histone lysine methyltransferase 1
**Q9P2K8-1**	EIF2AK4	-0.199	eukaryotic translation initiation factor 2 alpha kinase 4
**Q9NR50-1**	EIF2B3	-0.169	eukaryotic translation initiation factor 2B subunit gamma
**Q9UBQ5**	EIF3K	-0.249	eukaryotic translation initiation factor 3 subunit K
**P38919**	EIF4A3	0.288	eukaryotic translation initiation factor 4A3
**O43432-3**	EIF4G3	-0.273	eukaryotic translation initiation factor 4 gamma 3
**P55199**	ELL	0.319	elongation factor for RNA polymerase II
**Q14241**	ELOA	0.244	elongin A
**Q6IA86-6**	ELP2	-0.187	elongator acetyltransferase complex subunit 2
**Q9H9T3**	ELP3	-0.286	elongator acetyltransferase complex subunit 3
**Q96EB1-2**	ELP4	-0.377	elongator acetyltransferase complex subunit 4
**Q9P0I2**	EMC3	0.145	ER membrane protein complex subunit 3
**Q8N8S7-2**	ENAH	-0.334	ENAH actin regulator
**P17813**	ENG	-0.598	endoglin
**Q9UM22**	EPDR1	0.672	ependymin related 1
**Q9HCE0-1**	EPG5	-0.241	ectopic P-granules autophagy protein 5 homolog
**P29317**	EPHA2	0.303	EPH receptor A2
**P29323-3**	EPHB2	-0.373	EPH receptor B2
**P07099**	EPHX1	0.333	epoxide hydrolase 1
**O95208**	EPN2	0.616	epsin 2
**Q8IUD2-1**	ERC1	-0.36	ELKS/RAB6-interacting/CAST family member 1
**P19447**	ERCC3	0.22	ERCC excision repair 3, TFIIH core complex helicase subunit
**O43414-1**	ERI3	-0.402	ERI1 exoribonuclease family member 3
**A0FGR8-2**	ESYT2	0.155	extended synaptotagmin 2
**P62495**	ETF1	-0.271	eukaryotic translation termination factor 1
**P13804-1**	ETFA	0.203	electron transfer flavoprotein subunit alpha
**P38117**	ETFB	0.163	electron transfer flavoprotein subunit beta
**O60447-2**	EVI5	-0.398	ecotropic viral integration site 5
**Q96A65**	EXOC4	-0.454	exocyst complex component 4
**Q9Y2D4**	EXOC6B	-0.355	exocyst complex component 6B
**Q9NQT5**	EXOSC3	0.217	exosome component 3
**Q5RKV6**	EXOSC6	0.237	exosome component 6
**Q93063-3**	EXT2	-0.254	exostosin glycosyltransferase 2
**P13726**	F3	-0.28	coagulation factor III, tissue factor
**Q01469**	FABP5	-0.462	fatty acid binding protein 5
**O95864**	FADS2	-0.243	fatty acid desaturase 2
**Q9UNN5**	FAF1	-0.606	Fas associated factor 1
**Q6SJ93**	FAM111B	0.524	family with sequence similarity 111 member B
**Q8IWE2**	FAM114A1	-0.173	family with sequence similarity 114 member A1
**Q9NX05-1**	FAM120C	-0.235	family with sequence similarity 120C
**Q96A26**	FAM162A	0.229	family with sequence similarity 162 member A
**Q9NSI2**	FAM207A	-0.299	family with sequence similarity 207 member A
**O75063**	FAM20B	0.151	FAM20B glycosaminoglycan xylosylkinase
**Q92520**	FAM3C	0.193	family with sequence similarity 3 member C
**Q9H4H8**	FAM83D	0.365	family with sequence similarity 83 member D

**O95363**	FARS2	-0.279	phenylalanyl-tRNA synthetase 2, mitochondrial
**P25445**	FAS	0.371	Fas cell surface death receptor
**P49327**	FASN	-0.237	fatty acid synthase
**Q7L8L6**	FASTKD5	0.223	FAST kinase domains 5
**P23142**	FBLN1	-0.414	fibulin 1
**P35555**	FBN1	-0.478	fibrillin 1
**Q8N531-1**	FBXL6	0.619	F-box and leucine rich repeat protein 6
**Q9UKB1**	FBXW11	-0.15	F-box and WD repeat domain containing 11
**Q0JRZ9-1**	FCHO2	-0.328	FCH and mu domain containing endocytic adaptor 2
**O94868**	FCHSD2	-0.622	FCH and double SH3 domains 2
**Q8N0W3-2**	FCSK	-0.177	fucose kinase
**P37268**	FDFT1	-0.318	farnesyl-diphosphate farnesyltransferase 1
**P22570-3**	FDXR	0.539	ferredoxin reductase
**P22830-2**	FECH	0.296	ferrochelatase
**P39748**	FEN1	0.244	flap structure-specific endonuclease 1
**Q9UHY8-2**	FEZ2	-0.44	fasciculation and elongation protein zeta 2
**P02679**	FGG	-0.732	fibrinogen gamma chain
**Q2V2M9-4**	FHOD3	-0.406	formin homology 2 domain containing 3
**Q4L180**	FILIP1L	0.505	filamin A interacting protein 1 like
**P26885**	FKBP2	0.352	FKBP prolyl isomerase 2
**Q14254**	FLOT2	0.258	flotillin 2
**Q96CP2**	FLYWCH2	0.211	FLYWCH family member 2
**Q96PY5-3**	FMNL2	-0.531	formin like 2
**Q8IVF7**	FMNL3	-0.266	formin like 3
**P02751**	FN1	-0.919	fibronectin 1
**Q96RU3-1**	FNBP1	-0.409	formin binding protein 1
**Q53EP0**	FNDC3B	-0.503	fibronectin type III domain containing 3B
**Q5VW36**	FOCAD	-0.161	focadhesin
**P85037**	FOXK1	-0.256	forkhead box K1
**Q9BZ67**	FRMD8	0.388	FERM domain containing 8
**O94915-1**	FRYL	-0.235	FRY like transcription coactivator
**Q12841**	FSTL1	-0.42	follistatin like 1
**P02794**	FTH1	0.368	ferritin heavy chain 1
**P02792**	FTL	0.604	ferritin light chain
**Q9C0B1-1**	FTO	-0.411	FTO alpha-ketoglutarate dependent dioxygenase
**Q9UET6**	FTSJ1	-0.17	FtsJ RNA 2'-O-methyltransferase 1
**Q8IY81**	FTSJ3	0.117	FtsJ RNA 2'-O-methyltransferase 3
**P04066**	FUCA1	0.304	alpha-L-fucosidase 1
**Q9BTY2**	FUCA2	0.365	alpha-L-fucosidase 2
**Q13283**	G3BP1	-0.188	G3BP stress granule assembly factor 1
**Q9UN86**	G3BP2	-0.212	G3BP stress granule assembly factor 2
**P10253**	GAA	0.268	glucosidase alpha, acid
**O14976**	GAK	-0.151	cyclin G associated kinase
**Q86SR1**	GALNT10	-0.169	polypeptide N-acetylgalactosaminyltransferase 10
**Q14435**	GALNT3	-0.224	polypeptide N-acetylgalactosaminyltransferase 3
**Q8N4A0**	GALNT4	0.168	polypeptide N-acetylgalactosaminyltransferase 4
**Q86SF2**	GALNT7	-0.131	polypeptide N-acetylgalactosaminyltransferase 7

**Q9NY12**	GAR1	0.331	GAR1 ribonucleoprotein
**Q92538**	GBF1	-0.258	golgi brefeldin A resistant guanine nucleotide exchange factor 1
**Q92947-1**	GCDH	0.134	glutaryl-CoA dehydrogenase
**Q92616**	GCN1	-0.182	GCN1 activator of EIF2AK4
**P23434**	GCSH	0.258	glycine cleavage system protein H
**Q99988**	GDF15	0.849	growth differentiation factor 15
**O75223**	GGCT	-0.271	gamma-glutamylcyclotransferase
**Q9H3K2**	GHITM	0.276	growth hormone inducible transmembrane protein
**P17302**	GJA1	-0.251	gap junction protein alpha 1
**P32189-3**	GK	0.201	glycerol kinase
**P06280**	GLA	0.396	galactosidase alpha
**Q92896-2**	GLG1	-0.136	golgi glycoprotein 1
**P35754**	GLRX	-0.245	glutaredoxin
**O76003**	GLRX3	-0.447	glutaredoxin 3
**P00367**	GLUD1	0.232	glutamate dehydrogenase 1
**P15104**	GLUL	0.336	glutamate-ammonia ligase
**P17900**	GM2A	0.357	GM2 ganglioside activator
**O60547**	GMDS	-0.27	GDP-mannose 4,6-dehydratase
**P60983**	GMFB	-0.334	glia maturation factor beta
**O75496**	GMNN	0.463	geminin DNA replication inhibitor
**Q14344**	GNA13	0.279	G protein subunit alpha 13
**P63096-1**	GNAI1	0.15	G protein subunit alpha i1
**P63092-4**	GNAS	0.186	GNAS complex locus
**P62879**	GNB2	0.199	G protein subunit beta 2
**P61952**	GNG11	0.667	G protein subunit gamma 11
**P63218**	GNG5	0.343	G protein subunit gamma 5
**Q9BVP2**	GNL3	-0.249	G protein nucleolar 3
**P15586**	GNS	0.295	glucosamine (N-acetyl)-6-sulfatase
**Q9HD26**	GOPC	-0.151	golgi associated PDZ and coiled-coil motif containing
**P35052**	GPC1	0.443	glypican 1
**Q9Y625**	GPC6	-0.942	glypican 6
**Q9NQX3-2**	GPHN	-0.38	gephyrin
**Q8NFJ5**	GPRC5A	0.295	G protein-coupled receptor class C group 5 member A
**Q96CP6**	GRAMD1A	0.177	GRAM domain containing 1A
**Q13322-1**	GRB10	-0.539	growth factor receptor bound protein 10
**P62993**	GRB2	-0.527	growth factor receptor bound protein 2
**O60565**	GREM1	-0.5	gremlin 1, DAN family BMP antagonist
**Q8NEC7-1**	GSTCD	-0.379	glutathione S-transferase C-terminal domain containing
**P30711**	GSTT1	-0.149	glutathione S-transferase theta 1
**P13984**	GTF2F2	-0.415	general transcription factor IIF subunit 2
**P32780**	GTF2H1	0.239	general transcription factor IIH subunit 1
**A4D1E9**	GTPBP10	0.187	GTP binding protein 10
**Q9BZE4**	GTPBP4	-0.161	GTP binding protein 4
**Q9NYZ3**	GTSE1	0.23	G2 and S-phase expressed 1
**Q8N442**	GUF1	0.243	GUF1 homolog, GTPase
**P46976**	GYG1	0.319	glycogenin 1
**P16401**	H1-5	0.385	H1.5 linker histone, cluster member

**P0C0S5**	H2AZ1	0.234	H2A.Z variant histone 1
**Q16836-2**	HADH	0.136	hydroxyacyl-CoA dehydrogenase
**P40939**	HADHA	0.318	hydroxyacyl-CoA dehydrogenase trifunctional multienzyme complex subunit alpha
**P55084**	HADHB	0.263	hydroxyacyl-CoA dehydrogenase trifunctional multienzyme complex subunit beta
**P49590**	HARS2	0.161	histidyl-tRNA synthetase 2, mitochondrial
**O14929**	HAT1	0.165	histone acetyltransferase 1
**P53701**	HCCS	0.18	holocytochrome c synthase
**Q8WUI4-5**	HDAC7	-0.549	histone deacetylase 7
**Q9BXW7**	HDHD5	0.112	haloacid dehalogenase like hydrolase domain containing 5
**Q00341**	HDLBP	-0.289	high density lipoprotein binding protein
**Q6AI08**	HEATR6	-0.147	HEAT repeat containing 6
**Q9Y5Z4**	HEBP2	-0.493	heme binding protein 2
**Q5T447**	HECTD3	-0.185	HECT domain E3 ubiquitin protein ligase 3
**P07686**	HEXB	0.26	hexosaminidase subunit beta
**O94992**	HEXIM1	0.571	HEXIM P-TEFb complex subunit 1
**Q9BX68**	HINT2	0.203	histidine triad nucleotide binding protein 2
**P54198**	HIRA	-0.242	histone cell cycle regulator
**P04439**	HLA-A	0.208	major histocompatibility complex, class I, A
**P05534**	HLA-A	0.2	major histocompatibility complex, class I, A
**P01889**	HLA-B	0.336	major histocompatibility complex, class I, B
**P30481**	HLA-B	0.288	major histocompatibility complex, class I, B
**A0A140T9H5**	HLA-C	0.316	major histocompatibility complex, class I, C
**P20036**	HLA-DPA1	0.148	major histocompatibility complex, class II, DP alpha 1
**A0A182DWE9**	HLA-DPB1	0.246	major histocompatibility complex, class II, DP beta 1
**P20039**	HLA-DRB1	0.331	major histocompatibility complex, class II, DR beta 1
**Q14527**	HLTF	-0.311	helicase like transcription factor
**P09429**	HMGB1	0.182	high mobility group box 1
**P35914**	HMGCL	0.252	3-hydroxy-3-methylglutaryl-CoA lyase
**Q01581**	HMGCS1	-0.249	3-hydroxy-3-methylglutaryl-CoA synthase 1
**P05204**	HMGN2	0.236	high mobility group nucleosomal binding domain 2
**O00479**	HMGN4	0.442	high mobility group nucleosomal binding domain 4
**O75330-3**	HMMR	0.205	hyaluronan mediated motility receptor
**P09601**	HMOX1	0.236	heme oxygenase 1
**P30519**	HMOX2	0.171	heme oxygenase 2
**Q13151**	HNRNPA0	0.23	heterogeneous nuclear ribonucleoprotein A0
**P51991**	HNRNPA3	-0.256	heterogeneous nuclear ribonucleoprotein A3
**O14979-1**	HNRNPDL	-0.301	heterogeneous nuclear ribonucleoprotein D like
**P31943**	HNRNPH1	-0.174	heterogeneous nuclear ribonucleoprotein H1
**P52272**	HNRNPM	0.156	heterogeneous nuclear ribonucleoprotein M
**Q9BUJ2-1**	HNRNPUL1	0.126	heterogeneous nuclear ribonucleoprotein U like 1
**Q1KMD3**	HNRNPUL2	0.284	heterogeneous nuclear ribonucleoprotein U like 2
**Q86VS8**	HOOK3	-0.343	hook microtubule tethering protein 3
**Q5SSJ5**	HP1BP3	0.318	heterochromatin protein 1 binding protein 3
**P37235**	HPCAL1	-0.325	hippocalcin like 1
**O75506**	HSBP1	-0.3	heat shock factor binding protein 1
**P28845**	HSD11B1	0.556	hydroxysteroid 11-beta dehydrogenase 1
**P51659-1**	HSD17B4	0.172	hydroxysteroid 17-beta dehydrogenase 4

**Q3SXM5**	HSDL1	0.265	hydroxysteroid dehydrogenase like 1
**Q6YN16**	HSDL2	0.153	hydroxysteroid dehydrogenase like 2
**Q0VDF9**	HSPA14	-0.187	heat shock protein family A (Hsp70) member 14
**P0DMV9**	HSPA1A/HSPA1B	0.213	heat shock protein family A (Hsp70) member 1A
**P54652**	HSPA2	0.237	heat shock protein family A (Hsp70) member 2
**O95757**	HSPA4L	0.204	heat shock protein family A (Hsp70) member 4 like
**P38646**	HSPA9	0.184	heat shock protein family A (Hsp70) member 9
**Q9Y547**	HSPB11	-0.71	heat shock protein family B (small) member 11
**Q9UJY1**	HSPB8	0.377	heat shock protein family B (small) member 8
**P10809**	HSPD1	0.21	heat shock protein family D (Hsp60) member 1
**P61604**	HSPE1	0.552	heat shock protein family E (Hsp10) member 1
**P98160**	HSPG2	-0.461	heparan sulfate proteoglycan 2
**Q9BUP3-3**	HTATIP2	0.399	HIV-1 Tat interactive protein 2
**Q92743**	HTRA1	-0.554	HtrA serine peptidase 1
**Q7Z6Z7**	HUWE1	-0.288	HECT, UBA and WWE domain containing E3 ubiquitin protein ligase 1
**Q9NSE4**	IARS2	0.165	isoleucyl-tRNA synthetase 2, mitochondrial
**P05362**	ICAM1	0.144	intercellular adhesion molecule 1
**P48735**	IDH2	0.184	isocitrate dehydrogenase (NADP(+)) 2
**P50213**	IDH3A	0.195	isocitrate dehydrogenase (NAD(+)) 3 catalytic subunit alpha
**O43837**	IDH3B	0.246	isocitrate dehydrogenase (NAD(+)) 3 non-catalytic subunit beta
**Q16666-1**	IFI16	0.232	interferon gamma inducible protein 16
**P80217-2**	IFI35	0.195	interferon induced protein 35
**P09914**	IFIT1	0.175	interferon induced protein with tetratricopeptide repeats 1
**P09913**	IFIT2	0.318	interferon induced protein with tetratricopeptide repeats 2
**O14879**	IFIT3	0.382	interferon induced protein with tetratricopeptide repeats 3
**Q9NZI8**	IGF2BP1	-0.266	insulin like growth factor 2 mRNA binding protein 1
**Q9Y6M1**	IGF2BP2	-0.285	insulin like growth factor 2 mRNA binding protein 2
**O00425**	IGF2BP3	-0.256	insulin like growth factor 2 mRNA binding protein 3
**P11717**	IGF2R	-0.245	insulin like growth factor 2 receptor
**Q13123**	IK	0.174	IK cytokine
**Q70UQ0-4**	IKBIP	0.148	IKBKB interacting protein
**P01584**	IL1B	0.31	interleukin 1 beta
**Q9NQS7**	INCENP	0.358	inner centromere protein
**O15327**	INPP4B	-0.541	inositol polyphosphate-4-phosphatase type II B
**Q9Y2H2-1**	INPP5F	-0.224	inositol polyphosphate-5-phosphatase F
**Q8TEX9-2**	IPO4	-0.208	importin 4
**O95373**	IPO7	-0.265	importin 7
**Q5JU85-2**	IQSEC2	-0.164	IQ motif and Sec7 domain ArfGEF 2
**Q9Y616**	IRAK3	-0.42	interleukin 1 receptor associated kinase 3
**Q9H1B7**	IRF2BPL	0.306	interferon regulatory factor 2 binding protein like
**P35568**	IRS1	-0.186	insulin receptor substrate 1
**Q9BUE6-2**	ISCA1	0.354	iron-sulfur cluster assembly 1
**Q96J02**	ITCH	-0.306	itchy E3 ubiquitin protein ligase
**P26006**	ITGA3	-0.209	integrin subunit alpha 3
**P19827**	ITIH1	-0.703	inter-alpha-trypsin inhibitor heavy chain 1
**Q14624-1**	ITIH4	-0.751	inter-alpha-trypsin inhibitor heavy chain 4
**Q14571**	ITPR2	-0.26	inositol 1,4,5-trisphosphate receptor type 2

**Q8IWB1**	ITPRIP	0.253	inositol 1,4,5-trisphosphate receptor interacting protein
**Q15811**	ITSN1	-0.384	intersectin 1
**P26440**	IVD	0.153	isovaleryl-CoA dehydrogenase
**Q9Y6Y0**	IVNS1ABP	-0.306	influenza virus NS1A binding protein
**P23458**	JAK1	-0.214	Janus kinase 1
**Q9UK76-2**	JPT1	-0.441	Jupiter microtubule associated homolog 1
**P17275**	JUNB	0.276	JunB proto-oncogene, AP-1 transcription factor subunit
**O95251-1**	KAT7	0.419	lysine acetyltransferase 7
**Q9P0J7**	KCMF1	-0.215	potassium channel modulatory factor 1
**Q9Y2K7**	KDM2A	0.634	lysine demethylase 2A
**O15037**	KHNYN	0.244	KH and NYN domain containing
**Q92945**	KHSRP	0.145	KH-type splicing regulatory protein
**Q9BY89-1**	KIAA1671	-0.499	KIAA1671
**P52732**	KIF11	0.227	kinesin family member 11
**Q9H1H9**	KIF13A	-0.283	kinesin family member 13A
**Q9NQT8**	KIF13B	-0.236	kinesin family member 13B
**Q14807**	KIF22	0.398	kinesin family member 22
**Q99661**	KIF2C	0.269	kinesin family member 2C
**Q9BW19**	KIFC1	0.248	kinesin family member C1
**Q8TBB5**	KLHDC4	-0.43	kelch domain containing 4
**Q03164-3**	KMT2A	0.182	lysine methyltransferase 2A
**Q8NG31-1**	KNL1	0.185	kinetochore scaffold 1
**Q1ED39**	KNOP1	0.3	lysine rich nucleolar protein 1
**P50748**	KNTC1	-0.248	kinetochore associated 1
**P52294**	KPNA1	-0.241	karyopherin subunit alpha 1
**O00505**	KPNA3	-0.373	karyopherin subunit alpha 3
**O00629**	KPNA4	-0.314	karyopherin subunit alpha 4
**O60684**	KPNA6	-0.244	karyopherin subunit alpha 6
**Q14974**	KPNB1	-0.26	karyopherin subunit beta 1
**Q8N9T8**	KRI1	0.183	KRI1 homolog
**Q14533**	KRT81	-0.766	keratin 81
**Q9BQD3**	KXD1	-0.204	KxDL motif containing 1
**Q9H9P8-1**	L2HGDH	0.2	L-2-hydroxyglutarate dehydrogenase
**Q96EM0**	L3HYPDH	-0.199	trans-L-3-hydroxyproline dehydratase
**O15230**	LAMA5	-0.378	laminin subunit alpha 5
**P07942**	LAMB1	-0.278	laminin subunit beta 1
**P11047**	LAMC1	-0.319	laminin subunit gamma 1
**Q13753**	LAMC2	-0.271	laminin subunit gamma 2
**Q6IAA8**	LAMTOR1	0.191	late endosomal/lysosomal adaptor, MAPK and MTOR activator 1
**P28838-2**	LAP3	0.139	leucine aminopeptidase 3
**Q659C4**	LARP1B	-0.351	La ribonucleoprotein domain family member 1B
**P01130**	LDLR	-0.436	low density lipoprotein receptor
**Q8NC56-1**	LEMD2	0.316	LEM domain containing 2
**Q8WVC0**	LEO1	0.217	LEO1 homolog, Paf1/RNA polymerase II complex component
**O95202**	LETM1	0.14	leucine zipper and EF-hand containing transmembrane protein 1
**Q08380**	LGALS3BP	0.154	galectin 3 binding protein
**Q99538**	LGMN	0.19	legumain

**Q9UPQ0**	LIMCH1	-0.487	LIM and calponin homology domains 1
**Q03252**	LMNB2	0.167	lamin B2
**Q9Y4K0**	LOXL2	-0.435	lysyl oxidase like 2
**Q93052**	LPP	-0.314	LIM domain containing preferred translocation partner in lipoma
**P50851-2**	LRBA	-0.397	LPS responsive beige-like anchor protein
**O75427**	LRCH4	0.188	leucine rich repeats and calponin homology domain containing 4
**Q07954**	LRP1	-0.289	LDL receptor related protein 1
**P30533**	LRPAP1	0.178	LDL receptor related protein associated protein 1
**Q96AG4**	LRRC59	0.252	leucine rich repeat containing 59
**Q8IWT6**	LRRC8A	0.175	leucine rich repeat containing 8 VRAC subunit A
**Q6NSJ5**	LRRC8E	0.183	leucine rich repeat containing 8 VRAC subunit E
**Q6UWE0**	LRSAM1	-0.361	leucine rich repeat and sterile alpha motif containing 1
**Q9UFC0**	LRWD1	0.158	leucine rich repeats and WD repeat domain containing 1
**Q9BX40**	LSM14B	0.364	LSM family member 14B
**Q9Y4Z0**	LSM4	0.189	LSM4 homolog, U6 small nuclear RNA and mRNA degradation associated
**Q9UK45**	LSM7	0.216	LSM7 homolog, U6 small nuclear RNA and mRNA degradation associated
**Q14766-4**	LTBP1	-0.565	latent transforming growth factor beta binding protein 1
**O94822-3**	LTN1	-0.19	listerin E3 ubiquitin protein ligase 1
**Q96GA3**	LTV1	-0.257	LTV1 ribosome biogenesis factor
**Q9NQ29**	LUC7L	0.24	LUC7 like
**Q9Y383**	LUC7L2	0.271	LUC7 like 2, pre-mRNA splicing factor
**O95232**	LUC7L3	0.321	LUC7 like 3 pre-mRNA splicing factor
**Q9NX58**	LYAR	0.173	Ly1 antibody reactive
**Q5U5X0**	LYRM7	0.319	LYR motif containing 7
**Q8WZA0-2**	LZIC	-0.379	leucine zipper and CTNNBIP1 domain containing
**Q9UPN3**	MACF1	-0.34	microtubule actin crosslinking factor 1
**Q9UPN3-5**	MACF1	-0.255	microtubule actin crosslinking factor 1
**O75367**	MACROH2A1	0.252	macroH2A.1 histone
**Q9Y6D9**	MAD1L1	-0.186	mitotic arrest deficient 1 like 1
**Q8WXG6-4**	MADD	0.209	MAP kinase activating death domain
**Q9ULX9**	MAFF	0.885	MAF bZIP transcription factor F
**O00754**	MAN2B1	0.141	mannosidase alpha class 2B member 1
**O00462**	MANBA	-0.209	mannosidase beta
**P46821**	MAP1B	-0.221	microtubule associated protein 1B
**P11137**	MAP2	-0.164	microtubule associated protein 2
**Q9Y6R4-1**	MAP3K4	-0.284	mitogen-activated protein kinase kinase kinase 4
**P27816-1**	MAP4	-0.278	microtubule associated protein 4
**Q16644**	MAPKAPK3	-0.201	MAPK activated protein kinase 3
**Q969Z3-1**	MARC2	0.194	mitochondrial amidoxime reducing component 2
**P49006**	MARCKSL1	0.336	MARCKS like 1
**Q96GX5**	MASTL	0.323	microtubule associated serine/threonine kinase like
**Q9NZL9**	MAT2B	0.152	methionine adenosyltransferase 2B
**A0A0R4J2E8**	MATR3	0.149	matrin 3
**P43121**	MCAM	0.212	melanoma cell adhesion molecule
**P23508-2**	MCC	-0.536	MCC regulator of WNT signaling pathway
**Q96RQ3**	MCCC1	0.124	methylcrotonoyl-CoA carboxylase 1
**Q96EZ8-2**	MCRS1	-0.563	microspherule protein 1

**P40926**	MDH2	0.276	malate dehydrogenase 2
**P48163**	ME1	-0.181	malic enzyme 1
**P23368**	ME2	0.22	malic enzyme 2
**P51608-2**	MECP2	0.222	methyl-CpG binding protein 2
**Q15648**	MED1	0.305	mediator complex subunit 1
**Q9H944**	MED20	0.28	mediator complex subunit 20
**Q9Y316**	MEMO1	-0.359	mediator of cell motility 1
**Q7L2J0-1**	MEPCE	0.368	methylphosphate capping enzyme
**Q9H8H3**	METTL7A	0.26	methyltransferase like 7A
**P55081**	MFAP1	0.259	microfibril associated protein 1
**O95140**	MFN2	0.175	mitofusin 2
**P26572**	MGAT1	0.235	mannosyl (alpha-1,3-)-glycoprotein beta-1,2-N-acetylglucosaminyltransferase
**Q99685**	MGLL	-0.479	monoglyceride lipase
**Q9BQP7**	MGME1	0.512	mitochondrial genome maintenance exonuclease 1
**Q96PC5**	MIA2	0.176	MIA SH3 domain ER export factor 2
**O94851-4**	MICAL2	-0.294	microtubule associated monooxygenase, calponin and LIM domain containing 2
**Q7RTP6**	MICAL3	-0.212	microtubule associated monooxygenase, calponin and LIM domain containing 3
**Q99797**	MIPEP	-0.182	mitochondrial intermediate peptidase
**P46013**	MKI67	0.247	marker of proliferation Ki-67
**Q14165**	MLEC	0.194	malectin
**Q8NB16**	MLKL	-0.174	mixed lineage kinase domain like pseudokinase
**Q13015**	MLLT11	0.298	MLLT11 transcription factor 7 cofactor
**Q9Y4U1**	MMACHC	-0.521	metabolism of cobalamin associated C
**Q8N4V1**	MMGT1	0.68	membrane magnesium transporter 1
**P50281**	MMP14	-0.127	matrix metallopeptidase 14
**Q96T76-8**	MMS19	-0.208	MMS19 homolog, cytosolic iron-sulfur assembly component
**Q7Z3U7**	MON2	-0.259	MON2 homolog, regulator of endosome-to-Golgi trafficking
**Q14149**	MORC3	0.143	MORC family CW-type zinc finger 3
**Q15014**	MORF4L2	0.163	mortality factor 4 like 2
**Q8NHP6**	MOSPD2	0.453	motile sperm domain containing 2
**O00566**	MPHOSPH10	0.251	M-phase phosphoprotein 10
**Q99547**	MPHOSPH6	0.422	M-phase phosphoprotein 6
**Q99549**	MPHOSPH8	0.156	M-phase phosphoprotein 8
**P39210**	MPV17	0.252	mitochondrial inner membrane protein MPV17
**Q9UBG0**	MRC2	-0.132	mannose receptor C type 2
**P49959-3**	MRE11	0.196	MRE11 homolog, double strand break repair nuclease
**Q9HC36**	MRM3	0.341	mitochondrial rRNA methyltransferase 3
**Q9Y3B7-1**	MRPL11	0.147	mitochondrial ribosomal protein L11
**Q9NX20**	MRPL16	0.207	mitochondrial ribosomal protein L16
**Q9NRX2**	MRPL17	0.19	mitochondrial ribosomal protein L17
**P49406**	MRPL19	0.255	mitochondrial ribosomal protein L19
**P09001**	MRPL3	0.261	mitochondrial ribosomal protein L3
**Q9NYK5**	MRPL39	0.145	mitochondrial ribosomal protein L39
**Q9BRJ2**	MRPL45	0.217	mitochondrial ribosomal protein L45
**Q4U2R6**	MRPL51	0.244	mitochondrial ribosomal protein L51
**Q86TS9**	MRPL52	0.333	mitochondrial ribosomal protein L52
**Q6P161**	MRPL54	-0.136	mitochondrial ribosomal protein L54

**Q9BQC6**	MRPL57	0.32	mitochondrial ribosomal protein L57
**P82912**	MRPS11	0.256	mitochondrial ribosomal protein S11
**O15235**	MRPS12	0.488	mitochondrial ribosomal protein S12
**Q9Y3D5**	MRPS18C	0.362	mitochondrial ribosomal protein S18C
**Q9BYN8**	MRPS26	0.183	mitochondrial ribosomal protein S26
**Q9NP92**	MRPS30	0.213	mitochondrial ribosomal protein S30
**P82909**	MRPS36	0.271	mitochondrial ribosomal protein S36
**P82675**	MRPS5	0.164	mitochondrial ribosomal protein S5
**P82932**	MRPS6	0.277	mitochondrial ribosomal protein S6
**Q96E11**	MRRF	0.121	mitochondrial ribosome recycling factor
**P04731**	MT1A	1.035	metallothionein 1A
**O94776**	MTA2	0.237	metastasis associated 1 family member 2
**Q9NZJ7**	MTCH1	0.137	mitochondrial carrier 1
**Q6UB35-1**	MTHFD1L	-0.212	methylenetetrahydrofolate dehydrogenase (NADP+ dependent) 1 like
**P13995**	MTHFD2	0.311	methylenetetrahydrofolate dehydrogenase (NADP+ dependent) 2, methenyltetrahydrofola
**P42345**	MTOR	-0.184	mechanistic target of rapamycin kinase
**P58546**	MTPN	-0.451	myotrophin
**Q99707**	MTR	-0.241	5-methyltetrahydrofolate-homocysteine methyltransferase
**Q13505**	MTX1	0.15	metaxin 1
**O75431**	MTX2	0.162	metaxin 2
**Q5HYI7**	MTX3	0.391	metaxin 3
**Q03426**	MVK	-0.309	mevalonate kinase
**Q9BQG0-2**	MYBBP1A	-0.189	MYB binding protein 1a
**O75592-1**	MYCBP2	-0.362	MYC binding protein 2
**Q969H8**	MYDGF	0.265	myeloid derived growth factor
**P35579**	MYH9	-0.225	myosin heavy chain 9
**P24844-1**	MYL9	-0.613	myosin light chain 9
**Q12965**	MYO1E	-0.29	myosin IE
**Q13459**	MYO9B	-0.275	myosin IXB
**Q9NZM1**	MYOF	-0.139	myoferlin
**Q9Y2G1**	MYRF	-0.443	myelin regulatory factor
**Q14CX7**	NAA25	-0.192	N(alpha)-acetyltransferase 25, NatB auxiliary subunit
**Q9GZZ1-1**	NAA50	-0.2	N(alpha)-acetyltransferase 50, NatE catalytic subunit
**E9PAV3**	NACA	-0.449	nascent polypeptide associated complex subunit alpha
**P54920**	NAPA	-0.22	NSF attachment protein alpha
**Q8NEY1**	NAV1	-0.526	neuron navigator 1
**A2RRP1**	NBAS	-0.244	NBAS subunit of NRZ tethering complex
**Q53F19**	NCBP3	0.234	nuclear cap binding subunit 3
**Q9UBB6-3**	NCDN	-0.156	neurochondrin
**O43639**	NCK2	-0.225	NCK adaptor protein 2
**Q9NXR1-1**	NDE1	-0.208	nudE neurodevelopment protein 1
**Q9UGV2**	NDRG3	-0.28	NDRG family member 3
**O95299**	NDUFA10	0.197	NADH:ubiquinone oxidoreductase subunit A10
**O43678**	NDUFA2	0.18	NADH:ubiquinone oxidoreductase subunit A2
**Q16718**	NDUFA5	0.178	NADH:ubiquinone oxidoreductase subunit A5
**P56556**	NDUFA6	0.279	NADH:ubiquinone oxidoreductase subunit A6
**O95182**	NDUFA7	0.236	NADH:ubiquinone oxidoreductase subunit A7

**O75438**	NDUFB1	0.331	NADH:ubiquinone oxidoreductase subunit B1
**O96000**	NDUFB10	0.239	NADH:ubiquinone oxidoreductase subunit B10
**Q9NX14-2**	NDUFB11	0.188	NADH:ubiquinone oxidoreductase subunit B11
**O43674-1**	NDUFB5	0.231	NADH:ubiquinone oxidoreductase subunit B5
**O95139**	NDUFB6	0.136	NADH:ubiquinone oxidoreductase subunit B6
**O95169**	NDUFB8	0.175	NADH:ubiquinone oxidoreductase subunit B8
**Q9Y6M9**	NDUFB9	0.285	NADH:ubiquinone oxidoreductase subunit B9
**O95298**	NDUFC2	0.202	NADH:ubiquinone oxidoreductase subunit C2
**O43920**	NDUFS5	0.151	NADH:ubiquinone oxidoreductase subunit S5
**O75380**	NDUFS6	0.257	NADH:ubiquinone oxidoreductase subunit S6
**O75251**	NDUFS7	0.19	NADH:ubiquinone oxidoreductase core subunit S7
**O00217**	NDUFS8	0.245	NADH:ubiquinone oxidoreductase core subunit S8
**P49821**	NDUFV1	0.21	NADH:ubiquinone oxidoreductase core subunit V1
**P46934-4**	NEDD4	-0.273	NEDD4 E3 ubiquitin protein ligase
**Q96PU5**	NEDD4L	-0.361	NEDD4 like E3 ubiquitin protein ligase
**Q8IXH7**	NELFCD	0.188	negative elongation factor complex member C/D
**P18615**	NELFE	0.155	negative elongation factor complex member E
**O60524**	NEMF	-0.181	nuclear export mediator factor
**P19838-2**	NFKB1	-0.157	nuclear factor kappa B subunit 1
**Q96IV0**	NGLY1	-0.399	N-glycanase 1
**Q8NBF2**	NHLRC2	-0.18	NHL repeat containing 2
**Q9BZQ8**	NIBAN1	-0.264	niban apoptosis regulator 1
**Q96TA1**	NIBAN2	-0.208	niban apoptosis regulator 2
**Q9BPW8**	NIPSNAP1	0.286	nipsnap homolog 1
**O75323-1**	NIPSNAP2	0.534	nipsnap homolog 2
**Q9UFN0**	NIPSNAP3A	0.164	nipsnap homolog 3A
**Q9Y2I1**	NISCH	0.225	nischarin
**Q86X76-3**	NIT1	0.862	nitrilase 1
**O15226-2**	NKRF	0.218	NFKB repressing factor
**P30414**	NKTR	0.376	natural killer cell triggering receptor
**Q13423**	NNT	0.165	nicotinamide nucleotide transhydrogenase
**Q8WTT2**	NOC3L	0.218	NOC3 like DNA replication regulator
**Q9BVI4**	NOC4L	-0.15	nucleolar complex associated 4 homolog
**Q15233**	NONO	0.157	non-POU domain containing octamer binding
**Q9NZM5**	NOP53	-0.601	NOP53 ribosome biogenesis factor
**O00567**	NOP56	0.16	NOP56 ribonucleoprotein
**Q9Y2X3**	NOP58	0.135	NOP58 ribonucleoprotein
**O15118**	NPC1	0.169	NPC intracellular cholesterol transporter 1
**P06748-2**	NPM1	0.842	nucleophosmin 1
**Q12980**	NPRL3	-0.343	NPR3 like, GATOR1 complex subunit
**P15559-1**	NQO1	0.217	NAD(P)H quinone dehydrogenase 1
**P13056-1**	NR2C1	-0.657	nuclear receptor subfamily 2 group C member 1
**P04150-3**	NR3C1	0.225	nuclear receptor subfamily 3 group C member 1
**O14786-1**	NRP1	-0.369	neuropilin 1
**O95478**	NSA2	-0.454	NSA2 ribosome biogenesis factor
**O96028-1**	NSD2	-0.189	nuclear receptor binding SET domain protein 2
**Q9H857-2**	NT5DC2	0.137	5'-nucleotidase domain containing 2

**Q9Y266**	NUDC	-0.285	nuclear distribution C, dynein complex regulator
**Q96RS6**	NUDCD1	-0.171	NudC domain containing 1
**A8MXV4**	NUDT19	0.266	nudix hydrolase 19
**O43809**	NUDT21	0.221	nudix hydrolase 21
**Q7Z417**	NUFIP2	0.293	nuclear FMR1 interacting protein 2
**P49757**	NUMB	-0.194	NUMB endocytic adaptor protein
**Q8NFH3**	NUP43	0.167	nucleoporin 43
**Q9UKX7**	NUP50	0.17	nucleoporin 50
**Q9UBU9-1**	NXF1	0.351	nuclear RNA export factor 1
**Q9Y530**	OARD1	-0.636	O-acyl-ADP-ribose deacylase 1
**P04181**	OAT	0.22	ornithine aminotransferase
**Q56VL3**	OCIAD2	0.209	OCIA domain containing 2
**Q8N543**	OGFOD1	0.154	2-oxoglutarate and iron dependent oxygenase domain containing 1
**O15294**	OGT	-0.133	O-linked N-acetylglucosamine (GlcNAc) transferase
**Q9NTK5-1**	OLA1	-0.234	Obg like ATPase 1
**O60313-2**	OPA1	0.119	OPA1 mitochondrial dynamin like GTPase
**Q9BXB4**	OSBPL11	0.146	oxysterol binding protein like 11
**Q92882**	OSTF1	-0.464	osteoclast stimulating factor 1
**Q96FW1**	OTUB1	-0.236	OTU deubiquitinase, ubiquitin aldehyde binding 1
**P55809**	OXCT1	0.222	3-oxoacid CoA-transferase 1
**Q8N573-1**	OXR1	-0.273	oxidation resistance 1
**O95747**	OXSR1	-0.175	oxidative stress responsive kinase 1
**Q8IVL5**	P3H2	-0.319	prolyl 3-hydroxylase 2
**P11940**	PABPC1	-0.154	poly(A) binding protein cytoplasmic 1
**Q6VY07**	PACS1	-0.312	phosphofurin acidic cluster sorting protein 1
**Q9UKS6**	PACSIN3	0.211	protein kinase C and casein kinase substrate in neurons 3
**Q8N7H5**	PAF1	0.28	PAF1 homolog, Paf1/RNA polymerase II complex component
**P68402**	PAFAH1B2	-0.182	platelet activating factor acetylhydrolase 1b catalytic subunit 2
**Q9H074**	PAIP1	-0.209	poly(A) binding protein interacting protein 1
**Q8WX93-1**	PALLD	-0.208	palladin, cytoskeletal associated protein
**Q96RD7-1**	PANX1	0.193	pannexin 1
**Q8TEW0**	PARD3	-0.379	par-3 family cell polarity regulator
**O95453**	PARN	-0.285	poly(A)-specific ribonuclease
**P09874**	PARP1	0.139	poly(ADP-ribose) polymerase 1
**Q9UKK3**	PARP4	-0.151	poly(ADP-ribose) polymerase family member 4
**Q7L3T8**	PARS2	0.172	prolyl-tRNA synthetase 2, mitochondrial
**Q9HBI1**	PARVB	-0.254	parvin beta
**Q96IZ0**	PAWR	-0.276	pro-apoptotic WT1 regulator
**Q9Y5B6**	PAXBP1	0.419	PAX3 and PAX7 binding protein 1
**Q96AQ6**	PBXIP1	0.149	PBX homeobox interacting protein 1
**Q16822**	PCK2	0.185	phosphoenolpyruvate carboxykinase 2, mitochondrial
**Q15004**	PCLAF	0.453	PCNA clamp associated factor
**Q8NBM8**	PCYOX1L	0.29	prenylcysteine oxidase 1 like
**Q99447-3**	PCYT2	-0.216	phosphate cytidylyltransferase 2, ethanolamine
**Q14690**	PDCD11	-0.136	programmed cell death 11
**Q53EL6**	PDCD4	0.304	programmed cell death 4
**Q9H2J4**	PDCL3	-0.159	phosducin like 3

**P08559-4**	PDHA1	0.252	pyruvate dehydrogenase E1 alpha 1 subunit
**P11177**	PDHB	0.191	pyruvate dehydrogenase E1 beta subunit
**P30101**	PDIA3	0.193	protein disulfide isomerase family A member 3
**Q14554**	PDIA5	0.155	protein disulfide isomerase family A member 5
**Q96HC4**	PDLIM5	-0.208	PDZ and LIM domain 5
**Q9P0J1-2**	PDP1	0.29	pyruvate dehyrogenase phosphatase catalytic subunit 1
**Q5EBL8-2**	PDZD11	-0.243	PDZ domain containing 11
**Q9BY49-1**	PECR	0.525	peroxisomal trans-2-enoyl-CoA reductase
**Q9BRX2**	PELO	0.152	pelota mRNA surveillance and ribosome rescue factor
**P07737**	PFN1	-0.488	profilin 1
**P35080-2**	PFN2	-0.288	profilin 2
**Q96HS1-1**	PGAM5	0.175	PGAM family member 5, mitochondrial serine/threonine protein phosphatase
**O75167-4**	PHACTR2	-0.365	phosphatase and actin regulator 2
**P35232**	PHB	0.308	prohibitin
**Q99623**	PHB2	0.223	prohibitin 2
**O43189**	PHF1	0.729	PHD finger protein 1
**Q8WWQ0**	PHIP	0.312	pleckstrin homology domain interacting protein
**Q9BTU6**	PI4K2A	0.286	phosphatidylinositol 4-kinase type 2 alpha
**P42356**	PI4KA	-0.143	phosphatidylinositol 4-kinase alpha
**Q5H8A4-1**	PIGG	0.227	phosphatidylinositol glycan anchor biosynthesis class G
**O43164**	PJA2	-0.208	praja ring finger ubiquitin ligase 2
**P14618-2**	PKM	-0.157	pyruvate kinase M1/2
**Q99640**	PKMYT1	0.509	protein kinase, membrane associated tyrosine/threonine 1
**Q16512-2**	PKN1	-0.145	protein kinase N1
**Q16513**	PKN2	-0.196	protein kinase N2
**Q8NCC3**	PLA2G15	0.191	phospholipase A2 group XV
**P47712**	PLA2G4A	-0.248	phospholipase A2 group IVA
**Q8NHP8**	PLBD2	0.378	phospholipase B domain containing 2
**Q8N3E9**	PLCD3	0.262	phospholipase C delta 3
**Q8IV08**	PLD3	0.144	phospholipase D family member 3
**Q9HAU0-6**	PLEKHA5	-0.157	pleckstrin homology domain containing A5
**P00747**	PLG	-0.241	plasminogen
**Q9HBL7**	PLGRKT	0.28	plasminogen receptor with a C-terminal lysine
**O60568**	PLOD3	0.158	procollagen-lysine,2-oxoglutarate 5-dioxygenase 3
**Q14651**	PLS1	-0.268	plastin 1
**P13797**	PLS3	-0.206	plastin 3
**O75051**	PLXNA2	-0.445	plexin A2
**O15305**	PMM2	-0.284	phosphomannomutase 2
**Q10713**	PMPCA	0.207	peptidase, mitochondrial processing alpha subunit
**O75439**	PMPCB	0.202	peptidase, mitochondrial processing beta subunit
**Q15126**	PMVK	-0.186	phosphomevalonate kinase
**Q9H307**	PNN	0.135	pinin, desmosome associated protein
**Q9NRX1**	PNO1	-0.541	partner of NOB1 homolog
**Q9H488**	POFUT1	0.205	protein O-fucosyltransferase 1
**Q8NBL1**	POGLUT1	0.272	protein O-glucosyltransferase 1
**Q6UW63**	POGLUT2	0.182	protein O-glucosyltransferase 2
**Q7Z4H8**	POGLUT3	0.168	protein O-glucosyltransferase 3

**P09884**	POLA1	-0.228	DNA polymerase alpha 1, catalytic subunit
**P06746**	POLB	0.224	DNA polymerase beta
**P52434**	POLR2H	-0.346	RNA polymerase II subunit H
**P36954**	POLR2I	-0.317	RNA polymerase II subunit I
**P05423**	POLR3D	-0.171	RNA polymerase III subunit D
**Q96HA1**	POM121/POM121C	0.253	POM121 transmembrane nucleoporin
**Q9H2U2-2**	PPA2	0.177	inorganic pyrophosphatase 2
**P62937**	PPIA	-0.281	peptidylprolyl isomerase A
**P23284**	PPIB	0.18	peptidylprolyl isomerase B
**Q9UNP9**	PPIE	0.344	peptidylprolyl isomerase E
**P30405**	PPIF	0.462	peptidylprolyl isomerase F
**O15355**	PPM1G	0.148	protein phosphatase, Mg2+/Mn2+ dependent 1G
**Q96QC0**	PPP1R10	0.221	protein phosphatase 1 regulatory subunit 10
**Q8WUF5**	PPP1R13L	-0.228	protein phosphatase 1 regulatory subunit 13 like
**Q96C90**	PPP1R14B	-0.319	protein phosphatase 1 regulatory inhibitor subunit 14B
**Q6NYC8**	PPP1R18	0.186	protein phosphatase 1 regulatory subunit 18
**Q12972**	PPP1R8	0.262	protein phosphatase 1 regulatory subunit 8
**P30153**	PPP2R1A	-0.166	protein phosphatase 2 scaffold subunit Aalpha
**Q08209**	PPP3CA	-0.427	protein phosphatase 3 catalytic subunit alpha
**Q9NY27**	PPP4R2	0.147	protein phosphatase 4 regulatory subunit 2
**Q5H9R7-5**	PPP6R3	-0.3	protein phosphatase 6 regulatory subunit 3
**P50897**	PPT1	0.299	palmitoyl-protein thioesterase 1
**O60828**	PQBP1	0.344	polyglutamine binding protein 1
**Q86YV5**	PRAG1	-0.848	PEAK1 related, kinase-activating pseudokinase 1
**O43663**	PRC1	0.29	protein regulator of cytokinesis 1
**Q06830**	PRDX1	0.153	peroxiredoxin 1
**P48147**	PREP	-0.213	prolyl endopeptidase
**P49642**	PRIM1	-0.184	DNA primase subunit 1
**P49643**	PRIM2	-0.219	DNA primase subunit 2
**P17612**	PRKACA	-0.247	protein kinase cAMP-activated catalytic subunit alpha
**P22694**	PRKACB	-0.506	protein kinase cAMP-activated catalytic subunit beta
**P10644**	PRKAR1A	-0.316	protein kinase cAMP-dependent type I regulatory subunit alpha
**P17252**	PRKCA	-0.27	protein kinase C alpha
**O15091**	PRORP	-0.281	protein only RNase P catalytic subunit
**Q9UMS4**	PRPF19	0.2	pre-mRNA processing factor 19
**O43395**	PRPF3	0.214	pre-mRNA processing factor 3
**O43172**	PRPF4	0.178	pre-mRNA processing factor 4
**Q13523**	PRPF4B	0.249	pre-mRNA processing factor 4B
**P25788**	PSMA3	0.143	proteasome subunit alpha 3
**O14818-1**	PSMA7	0.172	proteasome subunit alpha 7
**P28074-1**	PSMB5	0.125	proteasome subunit beta 5
**P28065**	PSMB9	-0.185	proteasome subunit beta 9
**P35998**	PSMC2	0.153	proteasome 26S subunit, ATPase 2
**P17980**	PSMC3	0.141	proteasome 26S subunit, ATPase 3
**P43686**	PSMC4	0.122	proteasome 26S subunit, ATPase 4
**P62333**	PSMC6	0.224	proteasome 26S subunit, ATPase 6
**O43242**	PSMD3	0.18	proteasome 26S subunit, non-ATPase 3

**Q16401**	PSMD5	-0.276	proteasome 26S subunit, non-ATPase 5
**Q9H939-1**	PSTPIP2	0.16	proline-serine-threonine phosphatase interacting protein 2
**O95758-4**	PTBP3	0.208	polypyrimidine tract binding protein 3
**Q15185**	PTGES3	-0.489	prostaglandin E synthase 3
**Q14914**	PTGR1	0.158	prostaglandin reductase 1
**Q05397-5**	PTK2	-0.336	protein tyrosine kinase 2
**Q05209**	PTPN12	-0.257	protein tyrosine phosphatase non-receptor type 12
**Q15678**	PTPN14	-0.215	protein tyrosine phosphatase non-receptor type 14
**P10586-1**	PTPRF	-0.325	protein tyrosine phosphatase receptor type F
**Q15262-3**	PTPRK	-0.712	protein tyrosine phosphatase receptor type K
**Q9Y3E5**	PTRH2	0.279	peptidyl-tRNA hydrolase 2
**Q6GMV3**	PTRHD1	0.263	peptidyl-tRNA hydrolase domain containing 1
**Q15397**	PUM3	-0.371	pumilio RNA binding family member 3
**Q00577**	PURA	0.234	purine rich element binding protein A
**Q92626**	PXDN	-0.309	peroxidasin
**Q96C36**	PYCR2	0.277	pyrroline-5-carboxylate reductase 2
**Q9UL25**	RAB21	0.202	RAB21, member RAS oncogene family
**P20337**	RAB3B	-0.203	RAB3B, member RAS oncogene family
**Q9BUV8-5**	RAB5IF	0.431	RAB5 interacting factor
**P61006**	RAB8A	0.233	RAB8A, member RAS oncogene family
**P51151**	RAB9A	0.264	RAB9A, member RAS oncogene family
**Q9Y3P9**	RABGAP1	-0.193	RAB GTPase activating protein 1
**Q5R372-3**	RABGAP1L	-0.45	RAB GTPase activating protein 1 like
**P54727**	RAD23B	-0.425	RAD23 homolog B, nucleotide excision repair protein
**O43502**	RAD51C	0.669	RAD51 paralog C
**P78406**	RAE1	0.229	ribonucleic acid export 1
**Q6VN20**	RANBP10	0.237	RAN binding protein 10
**P52306-5**	RAP1GDS1	-0.509	Rap1 GTPase-GDP dissociation stimulator 1
**Q8TEU7-4**	RAPGEF6	-0.296	Rap guanine nucleotide exchange factor 6
**P20936-1**	RASA1	-0.239	RAS p21 protein activator 1
**Q14644**	RASA3	-0.297	RAS p21 protein activator 3
**Q9UJF2-2**	RASAL2	-0.411	RAS protein activator like 2
**Q09028**	RBBP4	0.202	RB binding protein 4, chromatin remodeling factor
**Q7Z6E9**	RBBP6	0.413	RB binding protein 6, ubiquitin ligase
**Q16576-1**	RBBP7	0.237	RB binding protein 7, chromatin remodeling factor
**O43251-8**	RBFOX2	-0.208	RNA binding fox-1 homolog 2
**Q9NTZ6**	RBM12	-0.315	RNA binding motif protein 12
**Q96T37**	RBM15	0.162	RNA binding motif protein 15
**Q9NW64**	RBM22	0.206	RNA binding motif protein 22
**Q5T8P6-2**	RBM26	0.329	RNA binding motif protein 26
**P52756**	RBM5	0.455	RNA binding motif protein 5
**Q9Y388**	RBMX2	0.595	RNA binding motif protein X-linked 2
**Q93062-3**	RBPMS	-0.335	RNA binding protein, mRNA processing factor
**P18754-2**	RCC1	0.268	regulator of chromosome condensation 1
**Q96I51**	RCC1L	0.224	RCC1 like
**Q14257**	RCN2	0.27	reticulocalbin 2
**Q6NUK4**	REEP3	0.253	receptor accessory protein 3

**Q00765**	REEP5	0.177	receptor accessory protein 5
**Q6NUM9-1**	RETSAT	0.28	retinol saturase
**P40938**	RFC3	0.273	replication factor C subunit 3
**P35249**	RFC4	0.249	replication factor C subunit 4
**P62745**	RHOB	0.248	ras homolog family member B
**P84095**	RHOG	0.165	ras homolog family member G
**Q8IXI1**	RHOT2	0.293	ras homolog family member T2
**Q5EBL4-1**	RILPL1	-0.381	Rab interacting lysosomal protein like 1
**Q06587**	RING1	0.162	ring finger protein 1
**Q9BVS4**	RIOK2	-0.183	RIO kinase 2
**O14730**	RIOK3	0.385	RIO kinase 3
**Q96TC7**	RMDN3	0.168	regulator of microtubule dynamics 3
**Q96GF1**	RNF185	0.27	ring finger protein 185
**Q5VTR2**	RNF20	0.271	ring finger protein 20
**Q63HN8-4**	RNF213	-0.249	ring finger protein 213
**O75150**	RNF40	0.218	ring finger protein 40
**O60942-1**	RNGTT	-0.312	RNA guanylyltransferase and 5'-phosphatase
**O43148-2**	RNMT	0.169	RNA guanine-7 methyltransferase
**Q15287-1**	RNPS1	0.285	RNA binding protein with serine rich domain 1
**Q13464**	ROCK1	-0.175	Rho associated coiled-coil containing protein kinase 1
**O75116**	ROCK2	-0.22	Rho associated coiled-coil containing protein kinase 2
**Q9H9Y2**	RPF1	-0.41	ribosome production factor 1 homolog
**Q9H7B2**	RPF2	-0.17	ribosome production factor 2 homolog
**Q6P5R6**	RPL22L1	-0.746	ribosomal protein L22 like 1
**P62829**	RPL23	-0.208	ribosomal protein L23
**P62899**	RPL31	-0.302	ribosomal protein L31
**P63173**	RPL38	-0.233	ribosomal protein L38
**Q6DKI1**	RPL7L1	-0.302	ribosomal protein L7 like 1
**P04843**	RPN1	0.154	ribophorin I
**Q9BUL9**	RPP25	0.25	ribonuclease P and MRP subunit p25
**P78345**	RPP38	0.368	ribonuclease P/MRP subunit p38
**Q5VT52**	RPRD2	0.3	regulation of nuclear pre-mRNA domain containing 2
**P62263**	RPS14	-0.213	ribosomal protein S14
**P62841**	RPS15	-0.152	ribosomal protein S15
**Q86WX3**	RPS19BP1	-0.253	ribosomal protein S19 binding protein 1
**P62854**	RPS26	-0.147	ribosomal protein S26
**P42677**	RPS27	-0.167	ribosomal protein S27
**P62857**	RPS28	-0.175	ribosomal protein S28
**P22090**	RPS4Y1	-0.345	ribosomal protein S4 Y-linked 1
**P08865**	RPSA	-0.179	ribosomal protein SA
**Q8N122**	RPTOR	-0.181	regulatory associated protein of MTOR complex 1
**P55042**	RRAD	1.152	RRAD, Ras related glycolysis inhibitor and calcium channel regulator
**P31350-2**	RRM2	0.498	ribonucleotide reductase regulatory subunit M2
**Q7LG56-6**	RRM2B	0.429	ribonucleotide reductase regulatory TP53 inducible subunit M2B
**Q9NYV6**	RRN3	0.308	RRN3 homolog, RNA polymerase I transcription factor
**O43818**	RRP9	0.116	ribosomal RNA processing 9, U3 small nucleolar RNA binding protein
**Q7L4I2**	RSRC2	0.234	arginine and serine rich coiled-coil 2

**Q15404**	RSU1	-0.17	Ras suppressor protein 1
**P60903**	S100A10	-0.285	S100 calcium binding protein A10
**Q15424-3**	SAFB	0.213	scaffold attachment factor B
**Q5K651**	SAMD9	0.197	sterile alpha motif domain containing 9
**Q9NSI8-1**	SAMSN1	-0.382	SAM domain, SH3 domain and nuclear localization signals 1
**O00422**	SAP18	0.27	Sin3A associated protein 18
**Q9UHR5-1**	SAP30BP	0.227	SAP30 binding protein
**O43290**	SART1	0.21	spliceosome associated factor 1, recruiter of U4/U6.U5 tri-snRNP
**Q8NBX0**	SCCPDH	0.159	saccharopine dehydrogenase (putative)
**Q86SK9**	SCD5	-0.581	stearoyl-CoA desaturase 5
**O43819**	SCO2	0.264	SCO cytochrome c oxidase assembly protein 2
**P22307**	SCP2	0.167	sterol carrier protein 2
**Q9HB40**	SCPEP1	0.19	serine carboxypeptidase 1
**Q14160-3**	SCRIB	0.141	scribble planar cell polarity protein
**Q99470**	SDF2	0.223	stromal cell derived factor 2
**P31040**	SDHA	0.3	succinate dehydrogenase complex flavoprotein subunit A
**P21912**	SDHB	0.206	succinate dehydrogenase complex iron sulfur subunit B
**P55735-3**	SEC13	-0.325	SEC13 homolog, nuclear pore and COPII coat complex component
**O60613**	SELENOF	-0.404	selenoprotein F
**Q9BQE4**	SELENOS	-0.302	selenoprotein S
**O75326-1**	SEMA7A	-0.549	semaphorin 7A (John Milton Hagen blood group)
**Q9UHD8-1**	SEPTIN9	-0.132	septin 9
**P05543**	SERPINA7	-0.492	serpin family A member 7
**P05121**	SERPINE1	-0.73	serpin family E member 1
**P36955**	SERPINF1	-0.687	serpin family F member 1
**Q01105**	SET	0.329	SET nuclear proto-oncogene
**Q15459**	SF3A1	0.22	splicing factor 3a subunit 1
**Q15428**	SF3A2	0.28	splicing factor 3a subunit 2
**Q12874**	SF3A3	0.261	splicing factor 3a subunit 3
**Q13435**	SF3B2	0.241	splicing factor 3b subunit 2
**P23246**	SFPQ	0.177	splicing factor proline and glutamine rich
**Q9H9B4**	SFXN1	0.235	sideroflexin 1
**A0A0A0MS41**	SFXN3	0.187	sideroflexin 3
**O95470**	SGPL1	0.213	sphingosine-1-phosphate lyase 1
**Q9H788**	SH2D4A	-0.283	SH2 domain containing 4A
**Q9Y371**	SH3GLB1	-0.234	SH3 domain containing GRB2 like, endophilin B1
**Q7Z6J0**	SH3RF1	-0.762	SH3 domain containing ring finger 1
**Q8NEM2**	SHCBP1	0.313	SHC binding and spindle associated 1
**P34897-1**	SHMT2	0.226	serine hydroxymethyltransferase 2
**Q9H173**	SIL1	-0.577	SIL1 nucleotide exchange factor
**O43166-2**	SIPA1L1	-0.23	signal induced proliferation associated 1 like 1
**O60292**	SIPA1L3	-0.416	signal induced proliferation associated 1 like 3
**Q96EB6**	SIRT1	0.328	sirtuin 1
**Q8IXJ6**	SIRT2	-0.156	sirtuin 2
**Q14493**	SLBP	0.331	stem-loop binding protein
**Q8WUM9**	SLC20A1	-0.313	solute carrier family 20 member 1
**P53007**	SLC25A1	0.248	solute carrier family 25 member 1

**Q00325-2**	SLC25A3	0.323	solute carrier family 25 member 3
**P05141**	SLC25A5	0.339	solute carrier family 25 member 5
**P12236**	SLC25A6	0.236	solute carrier family 25 member 6
**P11169**	SLC2A3	0.241	solute carrier family 2 member 3
**Q9Y6M5**	SLC30A1	0.589	solute carrier family 30 member 1
**Q8TB61**	SLC35B2	0.131	solute carrier family 35 member B2
**Q9H2H9**	SLC38A1	-0.352	solute carrier family 38 member 1
**Q96QD8**	SLC38A2	-0.368	solute carrier family 38 member 2
**Q8WUX1-1**	SLC38A5	-0.169	solute carrier family 38 member 5
**Q8IWA5-2**	SLC44A2	0.357	solute carrier family 44 member 2
**Q9Y6M7-7**	SLC4A7	-0.144	solute carrier family 4 member 7
**P30825**	SLC7A1	-0.143	solute carrier family 7 member 1
**P52569-3**	SLC7A2	-0.319	solute carrier family 7 member 2
**O14745**	SLC9A3R1	-0.153	SLC9A3 regulator 1
**Q15599-2**	SLC9A3R2	0.124	SLC9A3 regulator 2
**Q08AF3-1**	SLFN5	0.271	schlafen family member 5
**Q9NWH9-1**	SLTM	0.159	SAFB like transcription modulator
**O95391**	SLU7	0.328	SLU7 homolog, splicing factor
**P84022**	SMAD3	-0.273	SMAD family member 3
**Q92922**	SMARCC1	-0.276	SWI/SNF related, matrix associated, actin dependent regulator of chromatin subfamily c me
**P17405-1**	SMPD1	0.197	sphingomyelin phosphodiesterase 1
**P52788**	SMS	-0.156	spermine synthase
**P53814-5**	SMTN	0.457	smoothelin
**Q2TAY7**	SMU1	0.188	SMU1 DNA replication regulator and spliceosomal factor
**Q9HAU4**	SMURF2	-0.313	SMAD specific E3 ubiquitin protein ligase 2
**Q9H7B4**	SMYD3	-0.534	SET and MYND domain containing 3
**Q7KZF4**	SND1	-0.277	staphylococcal nuclease and tudor domain containing 1
**Q8TAD8**	SNIP1	0.241	Smad nuclear interacting protein 1
**P62306**	SNRPF	0.448	small nuclear ribonucleoprotein polypeptide F
**P62308**	SNRPG	0.414	small nuclear ribonucleoprotein polypeptide G
**Q13573**	SNW1	0.226	SNW domain containing 1
**Q15036-1**	SNX17	-0.257	sorting nexin 17
**Q96L92**	SNX27	-0.244	sorting nexin 27
**P04179**	SOD2	0.358	superoxide dismutase 2
**P18583-5**	SON	0.241	SON DNA binding protein
**Q99523**	SORT1	-0.847	sortilin 1
**Q53LP3**	SOWAHC	0.195	sosondowah ankyrin repeat domain family member C
**Q02447-1**	SP3	0.443	Sp3 transcription factor
**P09486**	SPARC	-0.166	secreted protein acidic and cysteine rich
**Q8NB90-1**	SPATA5	-0.279	spermatogenesis associated 5
**Q86XZ4**	SPATS2	-0.244	spermatogenesis associated serine rich 2
**Q9HBM1**	SPC25	-0.232	SPC25 component of NDC80 kinetochore complex
**Q96T58**	SPEN	0.16	spen family transcriptional repressor
**Q9UQ90**	SPG7	0.151	SPG7 matrix AAA peptidase subunit, paraplegin
**Q08AE8**	SPIRE1	-0.198	spire type actin nucleation factor 1
**P35270**	SPR	0.178	sepiapterin reductase
**Q13813**	SPTAN1	-0.304	spectrin alpha, non-erythrocytic 1

**Q01082-1**	SPTBN1	-0.179	spectrin beta, non-erythrocytic 1
**Q68D10**	SPTY2D1	0.472	SPT2 chromatin protein domain containing 1
**Q8WXA9-2**	SREK1	0.163	splicing regulatory glutamic acid and lysine rich protein 1
**Q7Z6B7**	SRGAP1	-0.431	SLIT-ROBO Rho GTPase activating protein 1
**O75044**	SRGAP2	-0.287	SLIT-ROBO Rho GTPase activating protein 2
**P49458**	SRP9	0.318	signal recognition particle 9
**Q96SB4-3**	SRPK1	-0.277	SRSF protein kinase 1
**Q9Y5M8**	SRPRB	0.172	SRP receptor subunit beta
**Q9BXP5**	SRRT	0.288	serrate, RNA effector molecule
**Q05519**	SRSF11	0.188	serine and arginine rich splicing factor 11
**Q08170**	SRSF4	0.324	serine and arginine rich splicing factor 4
**Q13243**	SRSF5	0.195	serine and arginine rich splicing factor 5
**Q13247**	SRSF6	0.245	serine and arginine rich splicing factor 6
**P05455**	SSB	0.235	small RNA binding exonuclease protection factor La
**Q04837**	SSBP1	0.34	single stranded DNA binding protein 1
**Q9UNL2-2**	SSR3	0.39	signal sequence receptor subunit 3
**O75886**	STAM2	-0.191	signal transducing adaptor molecule 2
**P51692**	STAT5B	-0.331	signal transducer and activator of transcription 5B
**O76061**	STC2	-0.617	stanniocalcin 2
**P31948-2**	STIP1	-0.179	stress induced phosphoprotein 1
**O94804**	STK10	-0.18	serine/threonine kinase 10
**Q13188-2**	STK3	-0.452	serine/threonine kinase 3
**Q9UEW8**	STK39	-0.345	serine/threonine kinase 39
**P27105**	STOM	0.312	stomatin
**O43752**	STX6	0.165	syntaxin 6
**Q9UNK0**	STX8	-0.471	syntaxin 8
**O00186**	STXBP3	0.249	syntaxin binding protein 3
**Q5T5C0-1**	STXBP5	-0.224	syntaxin binding protein 5
**P53999**	SUB1	0.438	SUB1 regulator of transcription
**P53597**	SUCLG1	0.278	succinate-CoA ligase alpha subunit
**Q9Y5B9**	SUPT16H	0.177	SPT16 homolog, facilitates chromatin remodeling subunit
**O00267-2**	SUPT5H	0.21	SPT5 homolog, DSIF elongation factor subunit
**Q7KZ85**	SUPT6H	0.21	SPT6 homolog, histone chaperone and transcription elongation factor
**O94864-1**	SUPT7L	0.36	SPT7 like, STAGA complex gamma subunit
**Q8IYB8**	SUPV3L1	0.148	Suv3 like RNA helicase
**O75683**	SURF6	-0.214	surfeit 6
**O95926**	SYF2	0.519	SYF2 pre-mRNA splicing factor
**Q8NF91**	SYNE1	-0.282	spectrin repeat containing nuclear envelope protein 1
**Q8WXH0-2**	SYNE2	-0.363	spectrin repeat containing nuclear envelope protein 2
**Q8N3V7-2**	SYNPO	0.326	synaptopodin
**Q9Y6A5**	TACC3	0.24	transforming acidic coiled-coil containing protein 3
**Q9BSH4**	TACO1	0.37	translational activator of cytochrome c oxidase I
**P09758**	TACSTD2	0.373	tumor associated calcium signal transducer 2
**Q86TJ2**	TADA2B	0.345	transcriptional adaptor 2B
**Q01995**	TAGLN	-0.289	transgelin
**Q96BW9-3**	TAMM41	1.028	TAM41 mitochondrial translocator assembly and maintenance homolog
**Q9C0D5**	TANC1	-0.293	tetratricopeptide repeat, ankyrin repeat and coiled-coil containing 1

**Q9HCD6-2**	TANC2	-0.434	tetratricopeptide repeat, ankyrin repeat and coiled-coil containing 2
**Q9H2K8**	TAOK3	-0.195	TAO kinase 3
**Q03518**	TAP1	0.317	transporter 1, ATP binding cassette subfamily B member
**Q03519-1**	TAP2	0.306	transporter 2, ATP binding cassette subfamily B member
**O15533-3**	TAPBP	0.216	TAP binding protein
**O14907**	TAX1BP3	-0.227	Tax1 binding protein 3
**Q9BYX2**	TBC1D2	-0.164	TBC1 domain family member 2
**Q92609-2**	TBC1D5	-0.355	TBC1 domain family member 5
**Q66K14**	TBC1D9B	-0.239	TBC1 domain family member 9B
**Q9BTW9**	TBCD	-0.284	tubulin folding cofactor D
**Q15813**	TBCE	-0.327	tubulin folding cofactor E
**Q9Y4P3**	TBL2	0.15	transducin beta like 2
**Q96EI5-2**	TCEAL4	-0.194	transcription elongation factor A like 4
**O14776**	TCERG1	0.185	transcription elongation regulator 1
**Q13428-3**	TCOF1	0.261	treacle ribosome biogenesis factor 1
**Q15554**	TERF2	0.249	telomeric repeat binding factor 2
**Q9NYB0**	TERF2IP	0.358	TERF2 interacting protein
**Q9Y6I9**	TEX264	0.254	testis expressed 264, ER-phagy receptor
**P02787**	TF	-0.224	transferrin
**Q00059**	TFAM	0.398	transcription factor A, mitochondrial
**Q9H5Q4**	TFB2M	0.124	transcription factor B2, mitochondrial
**Q92734**	TFG	0.203	trafficking from ER to golgi regulator
**P02786**	TFRC	-0.266	transferrin receptor
**P61812-2**	TGFB2	-0.654	transforming growth factor beta 2
**Q15582**	TGFBI	-0.548	transforming growth factor beta induced
**P37173-2**	TGFBR2	-0.752	transforming growth factor beta receptor 2
**P21980**	TGM2	-0.323	transglutaminase 2
**Q6YHU6**	THADA	-0.444	THADA armadillo repeat containing
**P07996**	THBS1	-0.938	thrombospondin 1
**P52888**	THOP1	-0.211	thimet oligopeptidase 1
**Q9Y2W1**	THRAP3	0.21	thyroid hormone receptor associated protein 3
**Q9NQ88**	TIGAR	0.173	TP53 induced glycolysis regulatory phosphatase
**Q9BVV7**	TIMM21	0.248	translocase of inner mitochondrial membrane 21
**O43615**	TIMM44	0.244	translocase of inner mitochondrial membrane 44
**P04183**	TK1	0.78	thymidine kinase 1
**Q9Y490**	TLN1	-0.253	talin 1
**O60603**	TLR2	-0.277	toll like receptor 2
**Q9UM00**	TMCO1	0.269	transmembrane and coiled-coil domains 1
**P17152**	TMEM11	0.224	transmembrane protein 11
**Q9H330-2**	TMEM245	0.34	transmembrane protein 245
**Q96MH6**	TMEM68	-0.668	transmembrane protein 68
**P42166**	TMPO	0.211	thymopoietin
**P42167**	TMPO	0.31	thymopoietin
**P62328**	TMSB10/TMSB4X	-0.693	thymosin beta 4 X-linked
**P63313**	TMSB10/TMSB4X	-0.387	thymosin beta 4 X-linked
**P24821**	TNC	-0.768	tenascin C
**Q03169**	TNFAIP2	0.345	TNF alpha induced protein 2

**O00220**	TNFRSF10A	0.201	TNF receptor superfamily member 10a
**O14763**	TNFRSF10B	0.313	TNF receptor superfamily member 10b
**Q9NP84**	TNFRSF12A	-0.278	TNF receptor superfamily member 12A
**Q15025**	TNIP1	-0.494	TNFAIP3 interacting protein 1
**Q9HBL0**	TNS1	-0.242	tensin 1
**Q68CZ2-1**	TNS3	-0.234	tensin 3
**Q8IZW8**	TNS4	-0.399	tensin 4
**P11388-4**	TOP2A	0.358	DNA topoisomerase II alpha
**Q92547**	TOPBP1	0.227	DNA topoisomerase II binding protein 1
**Q5JTV8**	TOR1AIP1	0.181	torsin 1A interacting protein 1
**Q8NFQ8**	TOR1AIP2	0.151	torsin 1A interacting protein 2
**Q9NXH8**	TOR4A	0.209	torsin family 4 member A
**P04637**	TP53	0.171	tumor protein p53
**Q16890**	TPD52L1	-0.203	TPD52 like 1
**P60174**	TPI1	0.234	triosephosphate isomerase 1
**P07951**	TPM2	-0.585	tropomyosin 2
**P07951-2**	TPM2	-0.227	tropomyosin 2
**P67936**	TPM4	-0.166	tropomyosin 4
**P12270**	TPR	0.178	translocated promoter region, nuclear basket protein
**Q9ULW0**	TPX2	0.309	TPX2 microtubule nucleation factor
**Q13595**	TRA2A	0.242	transformer 2 alpha homolog
**O43715**	TRIAP1	0.359	TP53 regulated inhibitor of apoptosis 1
**P19474**	TRIM21	0.314	tripartite motif containing 21
**Q8IYM9-1**	TRIM22	0.55	tripartite motif containing 22
**Q13263**	TRIM28	0.325	tripartite motif containing 28
**O75962-1**	TRIO	-0.262	trio Rho guanine nucleotide exchange factor
**Q15650**	TRIP4	-0.361	thyroid hormone receptor interactor 4
**Q7L0Y3**	TRMT10C	0.223	tRNA methyltransferase 10C, mitochondrial RNase P subunit
**Q96Q11**	TRNT1	0.15	tRNA nucleotidyl transferase 1
**Q16762**	TST	0.307	thiosulfate sulfurtransferase
**Q6P3X3**	TTC27	-0.374	tetratricopeptide repeat domain 27
**Q9C0H2-4**	TTYH3	0.454	tweety family member 3
**Q13509**	TUBB3	0.198	tubulin beta 3 class III
**P49411**	TUFM	0.223	Tu translation elongation factor, mitochondrial
**Q9H6E5**	TUT1	0.219	terminal uridylyl transferase 1, U6 snRNA-specific
**Q12792**	TWF1	-0.189	twinfilin actin binding protein 1
**P40222**	TXLNA	0.142	taxilin alpha
**Q8NBS9-1**	TXNDC5	0.28	thioredoxin domain containing 5
**P83876**	TXNL4A	-0.277	thioredoxin like 4A
**Q9NNW7**	TXNRD2	0.317	thioredoxin reductase 2
**Q2T9J0**	TYSND1	0.382	trypsin domain containing 1
**Q9NV66**	TYW1	-0.445	tRNA-yW synthesizing protein 1 homolog
**Q16222-1**	UAP1	-0.238	UDP-N-acetylglucosamine pyrophosphorylase 1
**P49459**	UBE2A	-0.234	ubiquitin conjugating enzyme E2 A
**P61086**	UBE2K	-0.469	ubiquitin conjugating enzyme E2 K
**P68036**	UBE2L3	-0.378	ubiquitin conjugating enzyme E2 L3
**Q9C0C9**	UBE2O	-0.235	ubiquitin conjugating enzyme E2 O

**Q7Z7E8**	UBE2Q1	0.191	ubiquitin conjugating enzyme E2 Q1
**Q16763**	UBE2S	0.2	ubiquitin conjugating enzyme E2 S
**Q9NPD8**	UBE2T	0.231	ubiquitin conjugating enzyme E2 T
**Q13404-1**	UBE2V1	-0.394	ubiquitin conjugating enzyme E2 V1
**Q15819**	UBE2V2	-0.478	ubiquitin conjugating enzyme E2 V2
**O95155-4**	UBE4B	-0.271	ubiquitination factor E4B
**P11441**	UBL4A	0.167	ubiquitin like 4A
**Q9BZL1**	UBL5	-0.406	ubiquitin like 5
**Q5T4S7-2**	UBR4	-0.191	ubiquitin protein ligase E3 component n-recognin 4
**P09936**	UCHL1	-0.181	ubiquitin C-terminal hydrolase L1
**P15374**	UCHL3	-0.191	ubiquitin C-terminal hydrolase L3
**Q8IX04**	UEVLD	-0.239	UEV and lactate/malate dehyrogenase domains
**O94874**	UFL1	0.205	UFM1 specific ligase 1
**Q9NUQ7**	UFSP2	0.155	UFM1 specific peptidase 2
**Q16739**	UGCG	-0.31	UDP-glucose ceramide glucosyltransferase
**Q96T88-2**	UHRF1	-0.292	ubiquitin like with PHD and ring finger domains 1
**Q96RL1**	UIMC1	-0.257	ubiquitin interaction motif containing 1
**O14795-2**	UNC13B	-0.786	unc-13 homolog B
**Q70J99-3**	UNC13D	-0.271	unc-13 homolog D
**Q9UDW1**	UQCR10	0.544	ubiquinol-cytochrome c reductase, complex III subunit X
**P14927**	UQCRB	0.315	ubiquinol-cytochrome c reductase binding protein
**P31930**	UQCRC1	0.126	ubiquinol-cytochrome c reductase core protein 1
**P22695**	UQCRC2	0.234	ubiquinol-cytochrome c reductase core protein 2
**P47985**	UQCRFS1	0.144	ubiquinol-cytochrome c reductase, Rieske iron-sulfur polypeptide 1
**P07919**	UQCRH	0.28	ubiquinol-cytochrome c reductase hinge protein
**O94763**	URI1	-0.219	URI1 prefoldin like chaperone
**Q9UPU5**	USP24	-0.196	ubiquitin specific peptidase 24
**Q8NFA0**	USP32	-0.298	ubiquitin specific peptidase 32
**Q13107-1**	USP4	-0.169	ubiquitin specific peptidase 4
**P40818**	USP8	-0.141	ubiquitin specific peptidase 8
**Q93008**	USP9X	-0.302	ubiquitin specific peptidase 9 X-linked
**Q9BVJ6-1**	UTP14A	-0.326	UTP14A small subunit processome component
**O75691**	UTP20	-0.26	UTP20 small subunit processome component
**Q9NQZ2**	UTP3	0.262	UTP3 small subunit processome component
**P46939**	UTRN	-0.318	utrophin
**Q5ST30-4**	VARS2	0.257	valyl-tRNA synthetase 2, mitochondrial
**P52735**	VAV2	-0.422	vav guanine nucleotide exchange factor 2
**P19320**	VCAM1	-0.786	vascular cell adhesion molecule 1
**P13611**	VCAN	-0.454	versican
**P18206**	VCL	-0.387	vinculin
**P21796**	VDAC1	0.164	voltage dependent anion channel 1
**P45880-1**	VDAC2	0.244	voltage dependent anion channel 2
**Q9Y277**	VDAC3	0.332	voltage dependent anion channel 3
**Q9HBM0-1**	VEZT	-0.548	vezatin, adherens junctions transmembrane protein
**Q96GC9**	VMP1	0.162	vacuole membrane protein 1
**Q709C8**	VPS13C	-0.237	vacuolar protein sorting 13 homolog C
**Q7Z3J2-1**	VPS35L	-0.165	VPS35 endosomal protein sorting factor like

**P49754**	VPS41	-0.244	VPS41 subunit of HOPS complex
**Q5VIR6-4**	VPS53	-0.267	VPS53 subunit of GARP complex
**Q96AJ9**	VTI1A	-0.406	vesicle transport through interaction with t-SNAREs 1A
**P04004**	VTN	-0.84	vitronectin
**O00401**	WASL	-0.173	WASP like actin nucleation promoting factor
**Q9Y2W2**	WBP11	0.388	WW domain binding protein 11
**Q969T9**	WBP2	-0.275	WW domain binding protein 2
**Q9NW82**	WDR70	-0.265	WD repeat domain 70
**Q6UXN9**	WDR82	0.253	WD repeat domain 82
**Q5T9L3-2**	WLS	-0.225	Wnt ligand secretion mediator
**Q96S55**	WRNIP1	0.18	WRN helicase interacting protein 1
**O00308**	WWP2	-0.592	WW domain containing E3 ubiquitin protein ligase 2
**Q9HCS7**	XAB2	0.195	XPA binding protein 2
**P98170**	XIAP	-0.363	X-linked inhibitor of apoptosis
**Q01831**	XPC	0.652	XPC complex subunit, DNA damage recognition and repair factor
**Q9C0E2**	XPO4	-0.218	exportin 4
**Q96QU8**	XPO6	-0.213	exportin 6
**Q9Y2Z4**	YARS2	0.147	tyrosyl-tRNA synthetase 2
**Q5BJH7**	YIF1B	0.202	Yip1 interacting factor homolog B, membrane trafficking protein
**Q9Y5A9**	YTHDF2	-0.209	YTH N6-methyladenosine RNA binding protein 2
**P61981**	YWHAG	-0.243	tyrosine 3-monooxygenase/tryptophan 5-monooxygenase activation protein gamma
**P63104**	YWHAZ	-0.18	tyrosine 3-monooxygenase/tryptophan 5-monooxygenase activation protein zeta
**Q8N4Q0**	ZADH2	0.166	zinc binding alcohol dehydrogenase domain containing 2
**O96006**	ZBED1	0.352	zinc finger BED-type containing 1
**O75152**	ZC3H11A	0.325	zinc finger CCCH-type containing 11A
**Q86VM9**	ZC3H18	0.173	zinc finger CCCH-type containing 18
**Q8IUH4**	ZDHHC13	0.249	zinc finger DHHC-type containing 13
**O60293**	ZFC3H1	0.187	zinc finger C3H1-type containing
**P17028-1**	ZNF24	0.351	zinc finger protein 24
**Q9Y2X9**	ZNF281	0.258	zinc finger protein 281
**Q8TF68-2**	ZNF384	0.888	zinc finger protein 384
**Q96NB3**	ZNF830	0.296	zinc finger protein 830
**O75312**	ZPR1	-0.197	ZPR1 zinc finger
**O43149**	ZZEF1	-0.273	zinc finger ZZ-type and EF-hand domain containing 1
**P62805**		0.268	
**P69905**		-0.552	
**P84243**		0.19	
